# Reactant-induced photoactivation of in situ generated organogold intermediates leading to alkynylated indoles via C*sp*^2^-C*sp* cross-coupling

**DOI:** 10.1038/s41467-022-29982-2

**Published:** 2022-04-28

**Authors:** Fen Zhao, Mehdi Abdellaoui, Wided Hagui, Maria Ballarin-Marion, Jérôme Berthet, Vincent Corcé, Stéphanie Delbaere, Héloïse Dossmann, Agathe Espagne, Jérémy Forté, Ludovic Jullien, Thomas Le Saux, Virginie Mouriès-Mansuy, Cyril Ollivier, Louis Fensterbank

**Affiliations:** 1 Sorbonne Université, CNRS, Institut Parisien de Chimie Moléculaire, 4 Place Jussieu, 75005 Paris, France; 2 Univ Lille, INSERM, CHU Lille, UMR-S 1172, Lille Neuroscience and Cognition Research Center, 59000 Lille, France; 3 PASTEUR, Département de Chimie, École Normale Supérieure, PSL University, Sorbonne Université, CNRS, 24, Rue Lhomond, 75005 Paris, France

**Keywords:** Reaction mechanisms, Photocatalysis

## Abstract

Photosensitization of organogold intermediates is an emerging field in catalysis. In this context, an access to 2,3-disubstituted indoles from *o*-alkynyl aniline and iodoalkyne derivatives via a gold-catalyzed sequence under visible-light irradiation and in the absence of an exogenous photocatalyst was uncovered. A wide scope of the process is observed. Of note, 2-iodo-ynamides can be used as electrophiles in this cross-coupling reaction. The resulting *N*-alkynyl indoles lend themselves to post-functionalization affording valuable scaffolds, notably benzo[a]carbazoles. Mechanistic studies converge on the fact that a potassium sulfonyl amide generates emissive aggregates in the reaction medium. Static quenching of these aggregates by a vinylgold(I) intermediate yields to an excited state of the latter, which can react with an electrophile via oxidative addition and reductive elimination to forge the key C-C bond. This reactant-induced photoactivation of an organogold intermediate opens rich perspectives in the field of cross-coupling reactions.

## Abstract (web summary)

Dual photo- and metal-catalysis is generally not well-understood when the metal catalyst is a gold complex. Here the authors show that a variation of a known metallaphotoredox annulation is possible without an added photocatalyst, proceeding putatively through a gold–intermediate photosensitization by the aggregate of a potassium amide reactant, and yielding indole derivatives.

## Introduction

In the quest of synthetic efficiency featuring notably step economy, dual catalysis through the merger of transition metal-catalysis and photocatalysis has constituted undoubtedly a major advance^1–3^. As early as in 2007, Osawa observed an acceleration of a copper-free palladium-catalyzed Sonogashira coupling in the presence of Ru(bpy)_3_(PF_6_)_2_ ^4^. It was claimed that the latter facilitates the oxidative addition step but no mechanistic evidence was provided. In 2011, the Sanford group highlighted that the photoreduction of an aryldiazononium salt via single electron transfer (SET) could be coupled to a Pd(II)/Pd(IV) manifold involving a directed C–H activation to provide bis-aryl products. The design of these transformations relied on the intertwining of a photoredox cycle with the palladium cycle, which ensures the adequate electronic shuttle^5^. This seminal report launched the field of metallaphotocatalysis^1^ which found valuable applications, notably in gold(I) catalysis a fertile field of investigations over the last two decades^6,7^. Indeed, in order to access to higher molecular complexity^8,9^, the frequently encountered protodeauration step has to be circumvented and the post-functionalization of organogold intermediates^10^ via C–C bond formation appears highly desirable. Nevertheless, in contrast to the commonly used metals in catalysis and due to the high redox potential of the Au(I)/Au(III) couple^11^, the oxidative addition at gold(I) is difficult. Initial reports have focused on the use of a stoichiometric oxidant^12,13^ and there has been a need to devise new pathways to promote it^14–17^. While ligand design for gold(I) has emerged as a possibility^18^, versatile catalytic cross-coupling reactions, notably thanks to the use of hemilabile (P,N) ligands, have been recently worked out^19^. In addition, Au(I)/Au(III) cycles have also been sustained via photoredox catalysis using aryl-diazonium or iodonium partners as SET partners^13,20–23^. Synthetically relevant sequences have resulted but the question to know how those actually work is still pending, notably because some of these reactions proceed in photocatalyst-free conditions^24–26^, or even under irradiation-free conditions in the simple presence of a mineral base^27^.

**Fig. 1:**
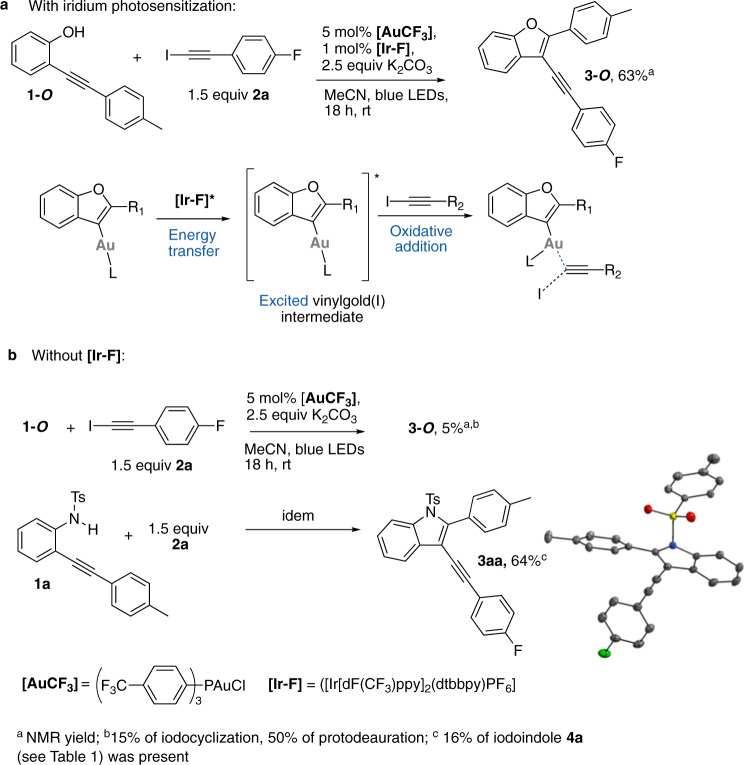
Gold(I) catalysis under visible-light. **a** Previous work^28^: alkynylative cyclization of *o*-alkynylphenols via phosensitized oxidative addition. **b** Contrasting reactivity between the phenol and the sulfonamide precursors **1-*O*** and **1a**.

Recently, we have reported a new reactivity paradigm by providing the first example of an energy transfer promoted oxidative addition at gold(I)^28,29^. Upon visible-light irradiation, an iridium photocatalyst triggers via triplet sensitization the oxidative addition of an alkynyliodide onto a vinylgold(I) intermediate to deliver Cs*p*^2^-C*sp* coupling products after reductive elimination. Mechanistic and modeling studies suggest that no redox pathway is involved but that an energy transfer^30–32^ takes place to generate a triplet state of the vinylgold intermediate. The latter can engage in an oxidative addition with an alkynyl or a vinyl iodide. This mode of activation in gold homogenous catalysis was applied in several dual catalytic processes. For instance, alkynylbenzofuran derivatives like **3-*O*** could be obtained from *o*-alkynylphenol **1-*O*** and iodoalkyne **2a** in the presence of a catalytic mixture of a gold(I) (**[AuCF**_**3**_**]**: ([(*p*-CF_3_Ph)_3_PAuCl]) and an iridium(III) complex ([Ir[dF(CF_3_)ppy]_2_(dtbbpy)PF_6_]) under blue LEDs irradiation (Fig. 1a).

While we were studying possible synthetic extensions of this chemistry, we made some puzzling observations when comparing the reactivity of *o*-alkynylphenol precursor **1-*O*** vs. *o*-alkynyl *N*-tosylamide **1a**. In iridium-free conditions, but in the presence of 5 mol% **[AuCF**_**3**_**]** and K_2_CO_3_ under blue LEDs irradiation, only a very little amount of **3-*O*** (5%) was observed (Fig. 1b). In sharp contrast, these iridium-free reaction conditions proved to be perfectly suited for the formation of indole **3aa** (64% yield) from NTs amide **1a**. Structures of **1a** and **3aa** were confirmed by X-ray diffraction (XRD) analysis.

In this work, the generality of this reaction is examined since indoles are key motifs in many relevant natural products and are also privileged scaffolds in medicinal chemistry, agrochemical industry, personal cares as well as dyes^33–35^. It is also the occasion to investigate the mechanism and notably how the heteroatomic nucleophilic component (*N*- vs. *O*-nucleophile) alters the photochemical activation process in these exogenous photocatalyst-free transformations.

## Results

### Optimization of the conditions

**Fig. 2:**
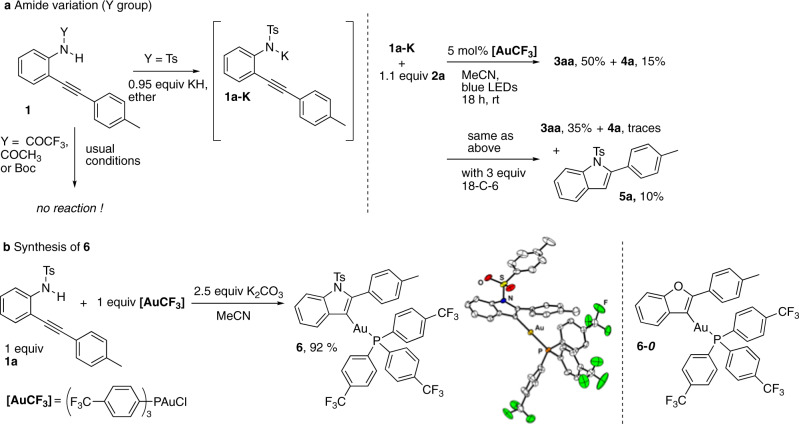
Preparation of putative intermediates and scope. **a** Preforming the potassic salt **1a-K** is possible to obtain the cross-coupling reaction. Of note, whatever the reaction conditions, no product was observed with an amide or a carbamate precursor. **b** Synthesis of **6** and its XRD structure.

We screened some variations on the reaction parameters in order to check if we were working in the most favorable set of conditions (Table 1). We first checked that the use of PPh_3_AuCl (entry 1) instead of **[AuCF**_**3**_**]** was detrimental for the reaction since only 50% of conversion was observed and only 8 % of **3aa** was detected on the crude ^1^H NMR spectrum, accompanied by 10% of iodoindole **4a** as iodocyclization by-product. The addition of 1 mol% **[Ir-F]** was productive in **3aa** but not as much as in photosensitizer free conditions (38% vs. 64%, entries 2 and 4) and the selectivity **3aa** vs. **4a** was lower. The addition of 10 mol% benzophenone as photosensitizer proved to have no effect in terms of yield and selectivity for **3aa** (entry 3). In order to reduce the quantity of **4a**, we diminished the source of iodine (iodoalkyne **2a**) in the reaction conditions. Engaging a 1:1.1 ratio of **1a** and **2a** (entry 5) proved to be as yielding as previous conditions (1:1.5 ratio, entry 4) but more selective toward **3aa**. Variation of the counter ion gave contrasted results and confirmed the supremacy of the potassium cation over sodium (entry 6), cesium (entry 7) and calcium (entry 8), all three resulting in poor or no conversion. Addition of 18-C-6 caused a consequent yield decrease (only 14% of **3aa**, entry 9). While we showed that the reaction could be run by using a preformed potassic salt **1a-K** resulting from the deprotonation with KH of **1a** (50% of **3aa**, Fig. 2a), addition of 18-C-6 was also deleterious in that case (35% of **3aa** accompanied by 10% of protodeauration product **5a**). All attempts with an amide (acetamide, trifluoroacetamide) or a carbamate (Boc) precursor failed to give any product. Of note also, no productive reaction leading to **3aa** was obtained from the corresponding bromoalkyne **2a-Br** and the chloroalkyne **2a-Cl**. Overall, this series of findings confirmed us in using a sulfonamide nucleophile and a potassium base in MeCN (see also Supplementary Table 1) for further developments.

**Table 1:** Optimization of the formation of indole 3aa.


Entry	1a:2a Ratio	Base	Additives^c^	3aa, yield (%)^a^	4a, yield (%)^a^
1^b^	1:1.5	K_2_CO_3_	–	8	10
2^c^	1:1.5	K_2_CO_3_	1 mol% **[Ir]**	38	18
3	1:1.5	K_2_CO_3_	10 mol% PhCOPh	61	16
4	1:1.5	K_2_CO_3_	–	64	16
5	1:1.1	K_2_CO_3_	–	62	10
6	1:1.1	Na_2_CO_3_	–	4	10
7	1:1.1	Cs_2_CO_3_	–	10	6
8	1:1.1	CaCO_3_	–	0	0
9	1:1.1	K_2_CO_3_	1 equiv 18-C-6	14	16

^a^NMR yield using 1,3,5-trimethoxybenzene as internal standard; all reactions run on a 0.1 mmol except for entries 3 and 4 (0.3 mmol).

^b^5 mol% PPh_3_AuCl was used instead of 5 mol% **[AuCF**_**3**_**]**.

^c^**[Ir]** = [Ir[dF(CF_3_)ppy]_2_(dtbbpy)PF_6_].

### Mechanistic studies

In order to extend the scope of possible substrates, we wished to get some insight into the mechanism of this transformation. The fact that a photo-triggered event occurs without any photocatalyst logically led us to consider the optical properties of the different partners present in the reaction medium. UV–vis absorption spectra were first recorded (Supplementary Figs. 1 and 2) and evidenced that the starting materials **1a** and **2a** as well as the cross-coupling product **3aa** at 50 μM were unable to absorb the light of the blue LED used (*λ*_max_ = 450 nm). Species presumably formed in situ were also studied such as vinylgold(I) intermediate **6**. The latter was smoothly obtained in 92% yield by reacting an equimolar mixture of **1a** and **[AuCF**_**3**_**]** in the presence of K_2_CO_3_ in MeCN (Fig. 2b). Crystals were collected and allowed a XRD analysis. Tosylamide anion **1a-K**, supposedly originating from the deprotonation of **1a** (pKa ≈ 10) in the basic medium was also examined.

**Fig. 3:**
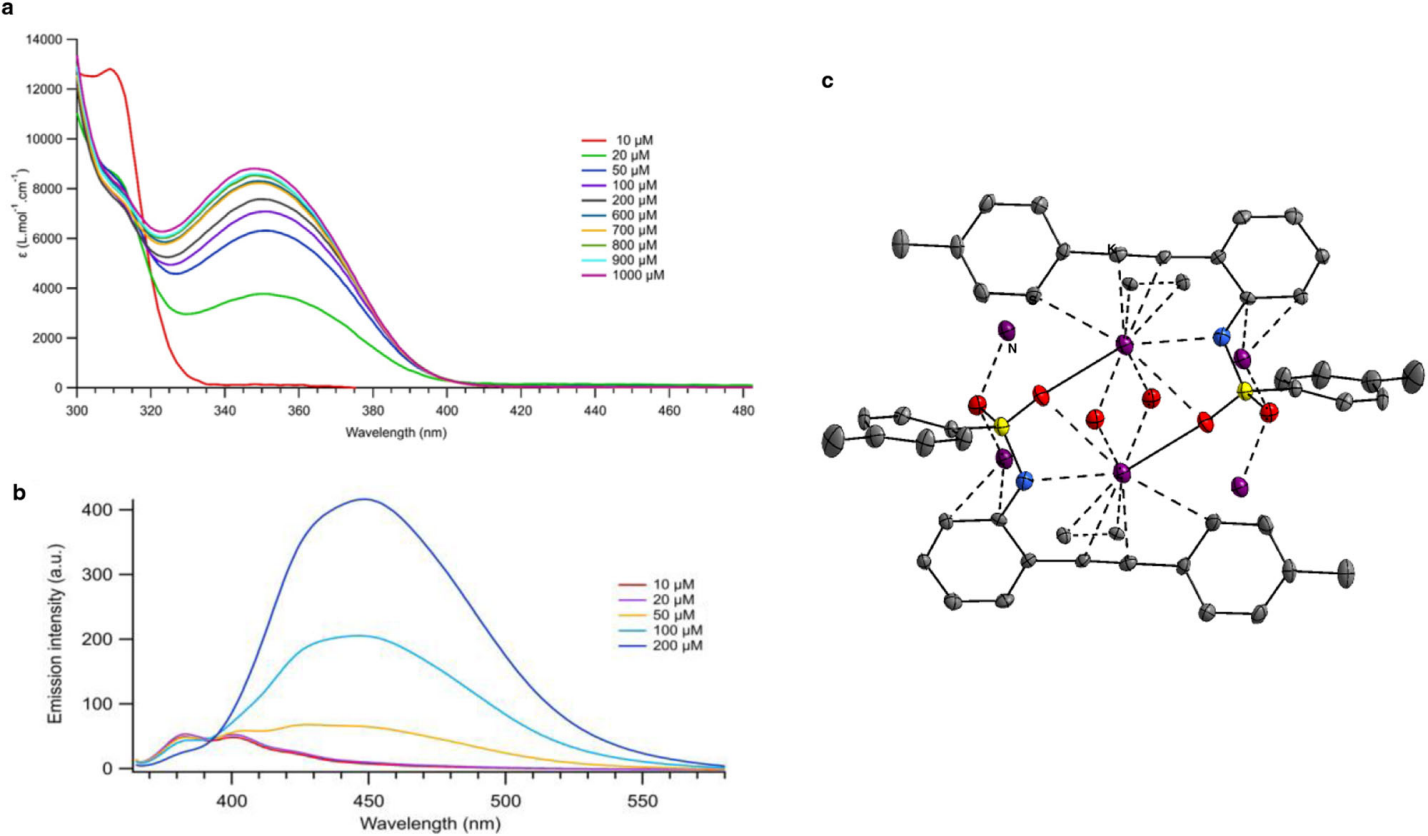
Characterization of 1a-K. Dependence from the concentration of **1a-K** in MeCN at 298 K. **a** On the molar absorption coefficient. **b** On the luminescence spectrum. **c** XRD structure of **1a-K**. Red atom: O, yellow atom: S, blue atom: N, purple atom: K.

Complex **6** did not exhibit absorption above 400 nm at 50 μM and comparison between **6** and **6-*O*** showed both vinylgold(I) intermediates exhibit a similar absorption profile (Supplementary Fig. 3). In contrast, vinylgold **6** proved much more emissive than **6-*O***, exhibiting a wide emission band at 400 nm (Supplementary Fig. 4). Thorough analysis of the photophysical properties of **1a-K** provided important information. First, we observed a dependence of the absorption properties on the concentration of **1a-K**. Indeed, by increasing the latter from 10 µM to 1 mM (Fig. 3a and Supplementary Fig. 5), the narrow absorption band at 310 nm collapsed and the appearance of a new wide band centered at 350 nm was observed. Second, upon increasing **1a-K** concentration, the emission band at 383 nm dropped at the benefit of a wide emission band centered at 450 nm (Fig. 3b and Supplementary Fig. 8). All these findings are in line with the formation of aggregates of **1a-K**, which could account for chromatic alteration and excimer formation in a regime of strong coupling between the chromophores^36^. In contrast, no such phenomenon was found with a potassic phenolate salt (Supplementary Fig. 6).

XRD analysis of **1a-K** strengthened both the idea of the formation of aggregates and the crucial role of the potassium counterion since it revealed that each potassium cation is octacoordinated (Fig. 3c). Besides the electrostatic interaction with the N atom, each potassium connects to four oxygen atoms of four distinct sulfonamide anions and also to two alkyne moieties through *η*^2^-slipped complexes featuring 3.0973–3.2589 Å and 3.3117–3.1062 Å distances. Such *η*^2^-slipped complexes remain rare^37,38^, but have been invoked in some reactions^39^. The last interaction consists in a *η*^1^-coordination between a carbon of the tolyl ring and the potassium cation with a distance of 3.3074 Å^37^. Conversely, one can understand the detrimental effect of 18-C-6 that would prevent the formation of this aggregate.

Liquid-state NMR experiments were conducted to monitor the aggregation of **1a-K** in solution. First, the ^1^H NMR spectra at concentrations ranging from 10 to 0.05 mM were recorded (Supplementary Fig. 44). It was observed that upon decreasing the concentration, the four proton signals of the di-*ortho* substituted aniline ring broaden and shift. At 0.05 mM, these broadened signals are at the same chemical shifts than those in pure **1a**. These observations are consistent with monomeric **1a-K** at that low concentration. Hydrogen (in **1a**) and potassium (in **1a-K**) atoms being similarly bounded to nitrogen, quadrupolar potassium (spin 3/2) alone generates signal broadening. The improvement of the signal resolution by increasing the concentration suggests that the molecules adopt progressively a different spatial organization. This is confirmed by acquiring NOE-1D experiments with 0.05, 0.5 and 10 mM solutions of **1a-K**. Clear dipolar interactions are observed between the *ortho*-aromatic proton of the tolyl group at 7.4 ppm with the two aromatic protons of the tosyl group at 7.2 and 7.7 ppm for **1a-K** at 0.5 and 10 mM, while none exists at 0.05 mM (Supplementary Fig. 45). Such intermolecular contacts are in agreement with the XRD structure proposed in Fig. 5. Finally, the self-diffusion coefficient of **1a-K** was determined at various concentrations (10, 5, 1, 0.5, 0.1 and 0.05 mM) in acetonitrile-d_3_ solutions at 295 K by the PGSE-NMR technique (Supplementary Fig. 46). Its value drops from the most diluted to the most concentrated solution, which suggests the formation of aggregates in compliance with the substantial increases of the estimated hydrodynamic radii and molecular weight (Supplementary Table 4).

The presence of the iodoalkyne **2a** does not alter the absorption properties of **1a-K** (Supplementary Fig. 10). Nevertheless, we observed by ^19^F NMR a significant shift of the peak associated to the fluorine atom of **2a** upon its titration by a related potassic amide **1g-K** at constant concentration of **2a** (Supplementary Fig. 11). This observation may suggest that **1g-K** and **2a** yield a complex engaged in a fast exchange with the free species. ^1^H and ^13^C NMR analyses corroborated this finding (see Supplementary Figs. 12 and 13).

**Fig. 4:**
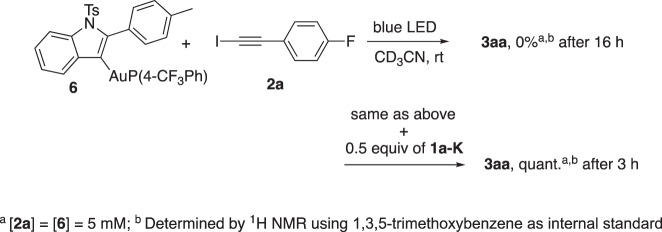
Impact of 1a-K on the direct irradiation of a mixture of 6 and 2a under blue LED irradiation. The presence of **1a-K** is necessary to obtain the indole cross-coupling product **3aa** from the preformed vinylgold intermediate **6**.

We obtained a satisfactory fit of the data by applying a 1:1 association model and retrieved *K*_a_^298K^ = 69 M^−1^ for the **1g-K**: **2a** complex association constant at 298 K (Supplementary Fig. 14). This value is consistent with the results of Goroff and Diederich^40^, who reported halogen bonding between amines and iodoalkynes of type **2**^41,42^. In contrast no such interaction was observed between **1a-K** and **2a-Br** and **2a-Cl** (Supplementary Figs. 15–24), which is consistent with the order of halogen bonding donor ability I > Br > Cl^42,43^. DFT calculations also supported the formation of an halogen bond between **1a-K** and **2a** and **2b** (Supplementary Figs. 38–40). By analogy with the phenol case^28^, we investigated the reaction of vinylgold **6** with iodoalkyne **2a** under blue LEDs irradiation. Thus, at 5 mM which is close to the maximum concentration of **6** in the preparative reactions (i.e. 3.3 mM of gold species, based on 0.3 mmol of **1a** and 5 mol% of **[AuCF**_**3**_**]** in 4.5 ml of MeCN, Supplementary Discussions II. 1. d.) almost no cross-coupling took place, since only traces of **3aa** were observed after 16 h of reaction (see Fig. 4 and Supplementary Figs. 25–27). This observation marked a sharp contrast with the one-pot reaction of entry 5 of Table 1 that smoothly delivers **3aa**. We surmised from these findings that a component formed in the reaction medium must promote the transformation. In view of the preceding series of experiments, we raised our suspicions on intermediate **1a-K**. Indeed, running the same NMR monitoring experiment in the presence of 0.5 equiv of **1a-K** vs. **1a** and **6** revealed a contrasting evolution of the reaction (Supplementary Figs. 28 and 29). As early as after 10 min, 17 % of **3aa** was formed, 45% of **3aa** after 0.5 h. After 3 h, the NMR yield reached 100% of **3aa**, based on the limiting one equivalent of **2a**. Two reactions can contribute to this yield. The first one would consist in the reaction of the vinylgold **6** photoexcited by **1a-K** with **2a** that also liberates (*p*-CF_3_Ph)_3_PAuI (see below). The latter can in turn catalyze the reaction of **1a-K** with **2a**, which also contributes to the formation of **3aa**. As an additional hint of a photosensitization pathway: direct irradiation of a mixture of **2a** and **6** in more concentrated conditions (20 mM) yields a rapid reaction leading to **3aa**, see Supplementary Figs. 25 and 30.

To gain further insight into the mechanism, we investigated the impact of **6** on the steady-state luminescence of the **1a-K** aggregate excited at 380 nm. We observed a marked luminescence drop when the concentration of **6** was increased (Supplementary Fig. 31). Stern–Volmer analysis yielded a slope equal to 9.4 × 10^3^ mol^−1^ l at 298 K (Supplementary Fig. 32). To properly interpret this value, we showed that the lifetime of 1a-K* was below 20 ns (Supplementary Fig. 33), which suggests that the excited aggregated 1a-K* is a singlet state. Hence, by assuming the quenching to be dynamic^28^, one would extract *k*_Q_^298K^ = 4.7.10^11^ l mol^−1^ s^−1^ as the lower limit of the rate constant of 1a-K* fluorescence quenching^44^. However, this value is significantly higher than the second-order rate constant for a reaction under control of the solvent diffusion evaluated to *k*_MeCN_^298K^ = 1.8 10^10^ l mol^−1^ s^−1^ (Supplementary Discussions II. 1. e.)^45,46^. To further eliminate the possibility of a dynamic quenching, we showed that a drop of the temperature increased the quenching efficiency (Supplementary Fig. 34): the slope of I/I_0_([**6**]) continuously increases from 1.8 × 10^3^ l mol^−1^ at 25 °C to 6.3 × 10^4^ l mol^−1^ at 0 °C. This could not be explained by a quenching under control of diffusion since the solvent viscosity increases when the temperature drops. Therefore, we concluded the quenching to be static and associated to the formation of a non-emissive complex between the **1a-K** aggregate and **6**. The slope of the Stern–Volmer analysis was interpreted as the thermodynamic association constant at 25 °C^44^ and its temperature dependence was analyzed with the Van’t-Hoff equation to retrieve Δ_r_H^°^ = −106.2 kJ mol^−1^ and Δ_r_S^°^ = −299.44 J mol^−1^ K^−1^ for the enthalpy and entropy of the **1a-K**:**6** complex formation respectively (Supplementary Figs. 35 and 36). Interestingly, the luminescence quenching of a **1a-K:2a** mixture by **6** even showed a higher slope and allowed to determine a fourfold higher association constant of **1a-K**:**2a** with **6** (Supplementary Fig. 37), which is consistent with a cooperative effect of halogen bonding between the amide and the iodoalkynes, this effect being absent in the case of a bromo- or a chloroalkyne.

**Fig. 5:**
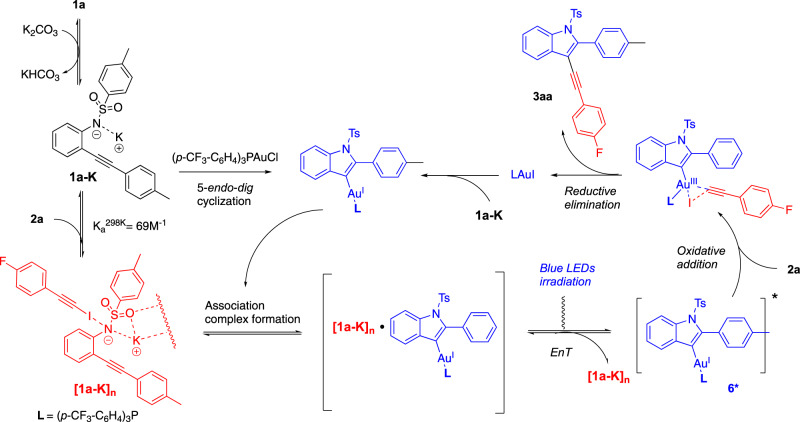
Synoptic mechanism. The blue light-excited **1a-K** aggregates in contact with the vinylgold **6** promote direct Dexter energy transfer. After intersystem conversion, triplet state 6* undergoes oxidative addition to iodoalkynes **2**. Reductive elimination liberates indoles **3**.

This would transpose in the reaction medium in such a way that the blue light-excited **1a-K** aggregates in contact with the vinylgold **6** would promote direct Dexter energy transfer. Further intersystem conversion would yield the triplet state 6*, which undergoes oxidative addition to iodoalkynes **2** (see Fig. 5 and Supplementary Fig. 41). After reductive elimination, the liberated LAuI could promote another catalytic cycle. In addition, DFT calculations supported the whole catalytic cycle and notably the photosensitized oxidative addition through bending of the iodoalkyne partner (Supplementary Fig. 41).

This cross-coupling process enters the examples of photochemical transformations under visible-light for which no exogenous photocatalyst is required to observe a photochemical activation. Several synthetic processes have been shown to follow such pathways and a large part of them have involved photoactive reactants or products^47^, electron donor-acceptor complexes^48–50^ or the in situ generation of visible-light absorbing intermediates^51^. Herein, the aggregation of one reactant is the trigger for reactivity. This type of activation remains poorly described and the most closely related example is the aggregation-induced photoreaction of 1,2-diisocyanoarenes^52^.

### Scope of the reaction

**Fig. 6:**
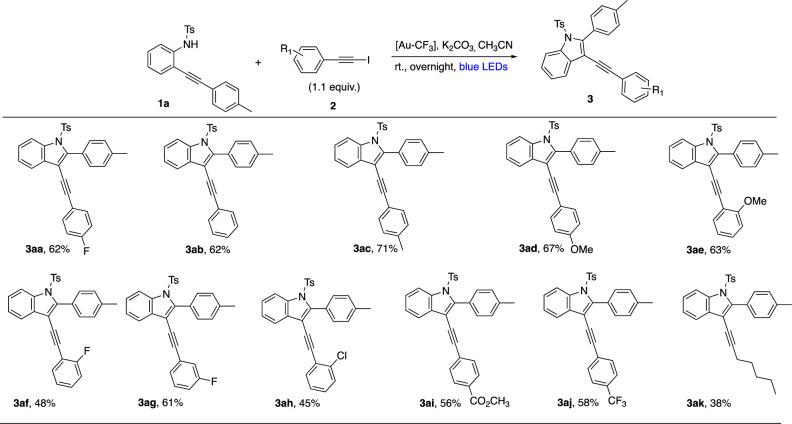
Scope of alkynes. The reaction works well with arylalkyne substrates **2** bearing a donor group or an electron-withdrawing group on the aromatic ring. In contrast, an alkylalkyne gave a more modest yield.

Having determined the activation process that governs these transformations, we studied the scope of the reaction. We first varied the alkynyl iodide partners **2** (Fig. 6). The reaction proved to be workable on a series of arylalkyne substrates bearing a donor group on the aromatic ring (67% of **3ad** and 63% of **3ae**, both of them bearing a methoxy group) or with an electron-withdrawing group (56% of **3ai** with an ester group, 58% of **3aj** with a CF_3_ group). In contrast, an alkyl chain on the alkyne gave a more modest yield (**3ak**, 38%).

**Fig. 7:**
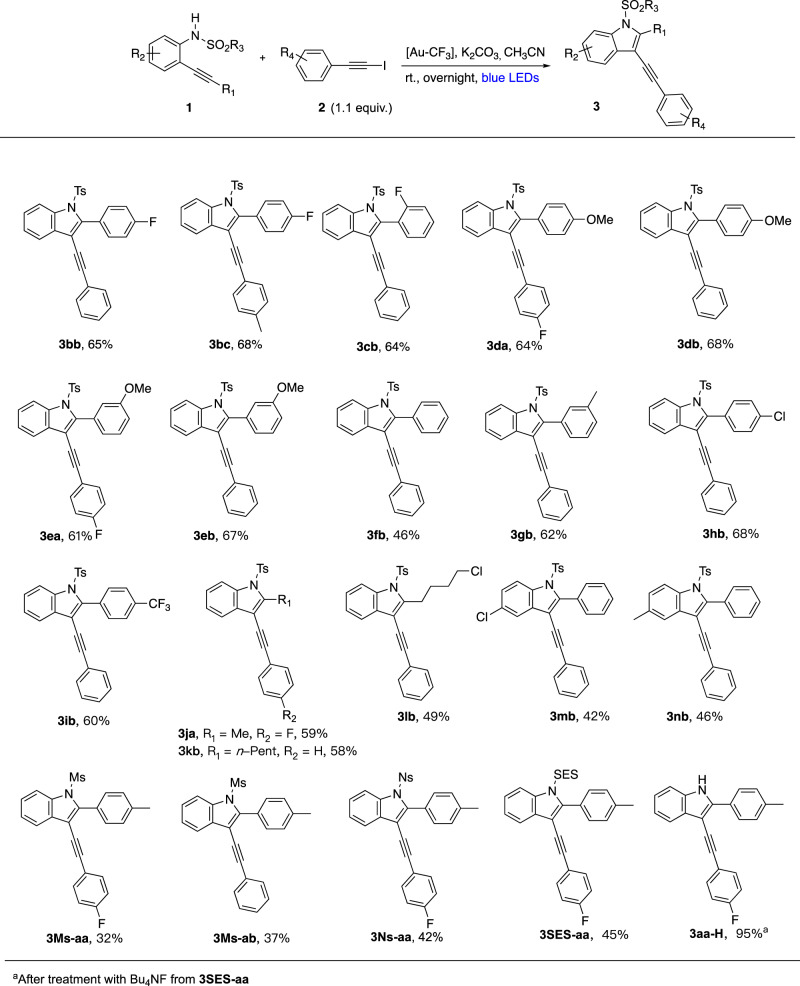
Scope of anilines. The reaction is compatible with a large scope of alkyne R_1_ substituents (aryl or alkyl). A methyl group on the alkyne is even possible (**3ja**). Variation of the sulfonyl group is possible. Easily removed nosyl (Ns) and 2-(trimethylsilyl)ethanesulfonyl (SES) groups are compatible with the reaction.

The aniline platform **1** could also be modulated (Fig. 7). Variation of the alkynyl substituent R_1_ was first examined. When dealing with an aromatic alkyne, no much yield variation was observed since most yields of products **3** were above 60% even with more contrasting electronic demand (64% of **da** and 68% of **db** with a 4-methoxy group vs. 60% of **3ib** with a 4-CF_3_ group). Of note, the presence of a 4-methoxy group on the aromatic ring did not significantly result in protodeauration on the contrary to our previous arylative cyclization of *O*-alkynyl phenols^28^. While for R_1_ = H no conversion was observed, donor alkyl groups on the alkyne proved also competent providing 2-alkyl indoles **3ja** (R_1_ = Me, R_2_ = F), **3** **kb** (R_1_ = *n*-Pent, R_2_ = H) and **3** **lb** (R_1_ = 4-Cl-*n*-Bu) in respectively 59%, 58% and 49% yields. Introduction of a chlorine or a methyl group in 4-position of the aniline precursor gave average yields (<50%) of products **3mb** and **3nb**. We also wished to survey the importance of a sulfonyl group attached on the nitrogen atom of the aniline precursor by varying the R_3_ group. In general more modest yields of indole products were obtained with mesylate precursors that gave the corresponding products **3Ms-aa** in 32% yield or **3Ms-ab** in 37% yield. Although the *N*-detosylation of indoles is known^53^, even more easily disposed sulfonamide groups were tested and 42% of nosyl-indole **3Ns-aa** and 45% of 2-(trimethylsilyl)ethanesulfonyl (SES) indole **3SES-aa** were obtained. The latter could be easily converted into the free NH indole **3aa-H** by treatment with Bu_4_NF as shown in Fig. 7.

**Fig. 8:**
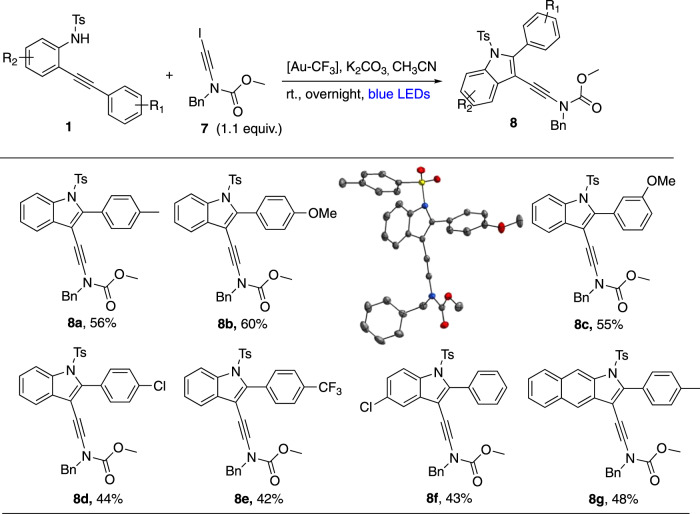
2-Iodo-ynamides as electrophiles. The extension of scope of alkynes **2** to the barely used 2-iodo-ynamide **7** partner proves to be possible. The corresponding products **8** offer post-functionalization opportunities.

In order to extend the synthetic scope of this process and also to provide substrates for valuable post-functionalizations, we focused our attention on 2-iodo-ynamide **7** as an electrophilic partner (Fig. 8). Interestingly, this substrate has been only described once by Danheiser who used it as a [2 + 2] cycloaddition partner^54^. From a broader perspective, very few examples of metal-catalyzed cross-coupling of such halo-ynamides have been published, all of them from bromo- or chloro-ynamides with copper^55–57^ but none with gold. Gratifyingly, though 2-iodo-ynamide **7** reacted a little bit less efficiently than iodoalkynes **2** with anilines **1**, a series of functionalized scaffolds **8** (for instance **8b**^33^) was obtained. Of note, a naphthyl aniline precursor could also be engaged to give adduct **8g** in 48%.

**Fig. 9:**
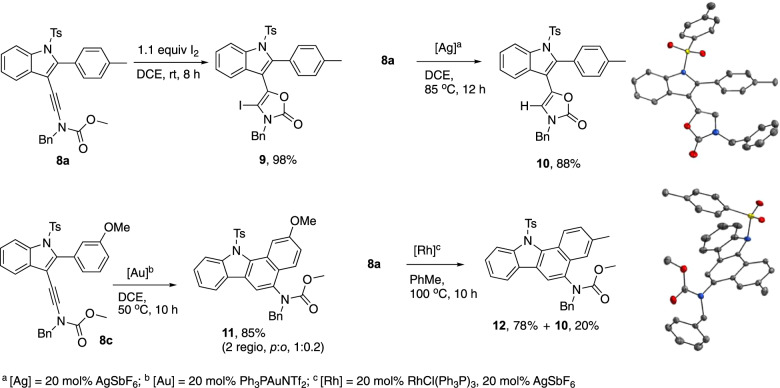
Post-functionalizations. Ynamide-indoles **8** lend themselves to various transformation providing valuable products such as iodo-oxazolone **9** and benzo[a]carbazoles **11** and **12**.

Taking advantage of the electron-richness of the ynamide moiety, we devised several transformations on polyfunctionalized substrates **8** (Fig. 9). While iodine smoothly promoted the formation of iodo-oxazolone **9**, a so far unknown type of derivatives, the corresponding Ag(I)-catalyzed cyclization in (wet) DCE provided the corresponding oxazolone **10** via protodesilveration^58,59^. Engaging the pendant aromatic ring in hydroarylation reaction was possible whether by gold(I) catalysis from **8c** or by cationic rhodium catalysis^60^ from the less activated substrate **8a** to provide the corresponding carbazoles **10** and **12**^61^. Benzo[a]carbazoles derivatives are important structural units found in many natural products and biologically active molecules^62,63^ as well as organic materials^64^.

In conclusion, this study uncovers an access to 2,3-disubstituted indoles from *o*-alkynyl aniline derivatives via a gold-catalyzed sequence under visible-light irradiation. Subsequent to the formation of a vinylgold(I) intermediate, a photosensitized oxidative addition at gold(I) of an alkynyl iodide yields to C*sp*^2^-C*sp* cross-coupling reactions. Of note, no exogenous photocatalyst is required in these processes. On the contrary, it was found that one of the in situ formed reactant, the potassium sulfonyl amide generates emissive aggregates in the reaction medium. Variation of the temperature in the fluorescence quenching studies established a static quenching of these aggregates by the vinylgold(I) intermediate to promote its excited state. After ISC to the triplet state of the latter, reaction with an electrophile via oxidative addition and reductive elimination forges the key C-C bond. Of note, 2-iodo-ynamides could be used as electrophiles in this cross-coupling reaction. The resulting *N*-alkynyl indoles lend themselves to various post-functionalizations providing valuable scaffolds including benzo[a]carbazoles. The present reactant-induced photoactivation of an organogold intermediate confirms the versatility of the photosensitized oxidation process and it opens rich perspectives in the field of cross-coupling reactions for the assembly of complex frameworks.

## Methods

### General procedure for the preparation of indoles **3**

The gold(I) complex (*p*-CF_3_Ph)_3_PAuCl (5 mol%), K_2_CO_3_ (2.5 equiv.), the appropriate iodoalkynes **2** (0.33 mmol, 1.1 equiv.) and *o*-alkynyl anilines derivatives **1** (0.3 mmol, 1.0 equiv.) were introduced in a Schlenk tube equipped with a magnetic stirring bar in which MeCN (4.5 ml) was added. The mixture was degassed using three freeze pump-thaw cycles and purged with argon, then irradiated with blue LEDs light for 15 h (unless mentioned). The stirring speed was equal to or more than 1200 rpm. The reaction was quenched with EtOAc (5 ml) and a 2 M HCl solution (6 ml) and the solution was extracted by EtOAc (3 × 5 ml). The combined organic layer was dried over MgSO_4_, filtered and concentrated under reduced pressure to give the crude product. The residue was purified by flash chromatography on silica gel to afford the desired product **3**.

## Supplementary information

Supplementary_Information

Description of Additional Supplementary Files

Supplementary Data 1

## Data Availability

Crystallographic data for the structures reported in this article have been deposited at the Cambridge Crystallographic Data Center under deposition numbers 2150836 (**1a**), 2090317 (**3aa**), 2090321 (**6**), 2090323 (**1a-K**), 2090316 (**8b-Triclinic**), 2090315 (**8b-Monoclinic**), 2090322 (**10**), 2090314 (**12**). Copies of the data can be obtained free of charge via https://www.ccdc.cam.ac.uk/structures/. All other data supporting the findings of this study are available within the article and the Supplementary Information, or from the corresponding authors upon request.
