# Supplementary material for: Reactant-induced photoactivation of in situ generated organogold intermediates leading to alkynylated indoles via Csp2-Csp cross-coupling
Source: Nat Commun. 2022 Apr 28;13:2295. doi: 10.1038/s41467-022-29982-2 (PMC9051093; doi:10.1038/s41467-022-29982-2)
Supplement: Supplementary file 1 — Supplementary_Information [file 41467_2022_29982_MOESM1_ESM.pdf]

Supplementary Information

Reactant-Induced Photoactivation of In Situ Generated Organogold Intermediates Leading to Alkynylated Indoles *via* C*sp*^{2}-C*sp* Cross-Coupling

Fen Zhao,[a]‡ Mehdi Abdellaoui,[a]‡ Wided Hagui,[a] Maria Ballarin Marion,[a] Jérôme Berthet,[b] Vincent Corcé,[a] Stéphanie Delbaere,[b] Héloïse Dossmann,[a] Agathe Espagne,[c] Jérémy Forté,[a] Ludovic Jullien,[c] Thomas Le Saux,[c] Virginie Mouriès-Mansuy,[a]* Cyril Ollivier,[a]* Louis Fensterbank[a]*

[a] Sorbonne Université, CNRS, Institut Parisien de Chimie Moléculaire, IPCM, 75005 Paris, France
[b] Univ Lille, INSERM, CHU Lille, UMR-S 1172, Lille Neuroscience and Cognition Research Center, 59000, Lille, France
[c] PASTEUR, Département de chimie, École normale supérieure, PSL University, Sorbonne Université, CNRS, 24, rue Lhomond, 75005 Paris, France

‡ These authors contributed equally to the work

Table of Contents

I. Supplementary Notes 3
II. Supplementary Discussions 4
1. Mechanistic investigations 4
a. Stoichiometry, counterion and solvent 4
b. Absorption and emission studies 5
c. NMR titration and bind-fitting experiments 11
d. Stoichiometric experiments with vinylgold(I) 6 26
e. Quenching experiments 33
f. Modelling part 38
g. NMR experiments on 1a-K 45
2. Experimental details and analytical data 50
a. Synthesis of starting materials 1 50
b. Synthesis of iodoalkynes derivatives 2 and 7 58
c. Synthesis of methyl benzyl(iodoethynyl)carbamate 7[39] 61
d. Synthesis of vinylgold(I) complex 6 62
e. Synthesis of indoles 3 and 8 63
f. Post-functionalization 83
g. Side products 86
3. NMR Spectra 87
4. X-Ray crystal structure determinations 169
III. Supplementary References 177

I. Supplementary Notes

All reactions involving air sensitive reagents or intermediates were carried out in pre-heated glassware under an argon atmosphere using standard Schlenk techniques.

All solvents and chemicals were used as received from suppliers (*Alfa Aesar, Sigma Aldrich, TCI*). Acetonitrile and dichloroethane were purified by distillation over calcium hydride under dry Argon atmosphere. Photocatalyst $Ir[dF(CF_3)ppy]_2(dtbbpy)PF_6$ was prepared according to the procedure of Yoon[1] and Weaver[2] respectively. The gold complex is commercially available and is used as received.

Chromatographic purifications of products were accomplished using force-flow chromatography (FC) on Macherey-Nagel SI 60 Å (40 –63 µm) silica gel according to the method of Still. Thin layer chromatography (TLC) was performed on Merck 60 F254 silica gel plates. Filtrations through Celite® were performed using Hyflo Super Cel from VWR. ^{1}H NMR spectra were recorded on a Brucker 400 AVANCE or 300 AVANCE (400 and 300 MHz respectively) and are calibrated with residual $CDCl_3$ protons signals at δ7.26 ppm and δ1.93 ppm for CD_3CN. ^{13}C NMR spectra were recorded on a Brucker 400 AVANCE or 300 AVANCE (100 and 75 MHz respectively) and are calibrated with $CDCl_3$ signal at δ77.16 ppm, ^{19}F spectra were recorded at 376.5 or 282.4 MHz and were calibrated with fluorobenzene in CD_3CN as internal standard at δ-115.0 ppm. Data are reported as follows: chemical shift (δ ppm), multiplicity (s = singlet, d = doublet, t = triplet, q = quartet, qt = quintuplet, m = multiplet, bs = broad signal), coupling constant (Hz) and integration.

High-resolution mass spectra were recorded on a micrOTOF (Bruker Daltonics) or a LTQ-XL/Orbitrap hybrid instrument (Thermo Fisher) using electrospray (ESI) or Atmospheric Pressure Chemical Ionization (APCI).

II. Supplementary Discussions

1. Mechanistic investigations

a. Stoichiometry, counterion and solvent

Procedure for the optimization of the stoichiometry

To a Schlenk flame-dried reactor, the alkyne **2a** (81 mg, 0.33 mmol, 1.1 equiv.), the aniline **1a** (108 mg, 0.3 mmol, 1 equiv.), the gold catalyst (11 mg, 0.015 mmol, 5 mol %), the base K_2CO_3 (103 mg, 0.75 mmol, 2.5 equiv.) and the additive were added. The reaction flask is placed under vacuum and filled with argon. The acetonitrile was degassed prior adding to the reaction mixture. The Schlenk tube was irradiated under blue light and stirred for one night. the volume of MeCN was fixed at 4.5 mL.

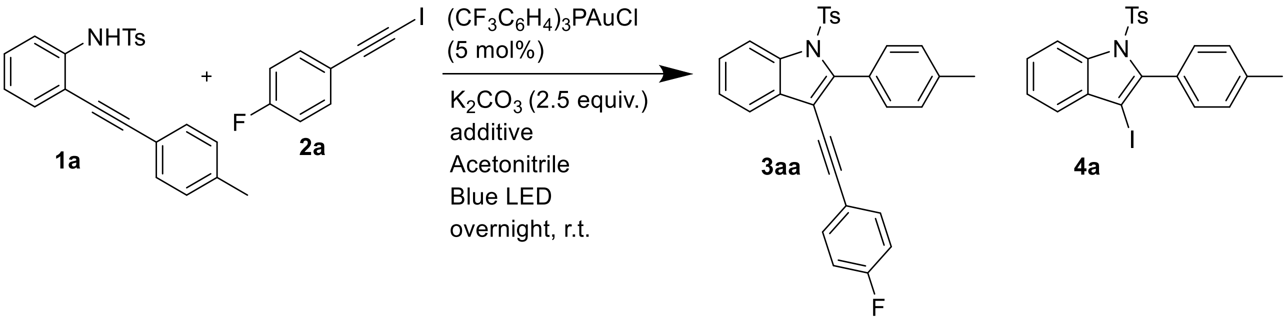

Procedure for the counterion study for the optimization

The aniline **1a** (36 mg, 0.10 mmol), the alkyne iodine **2a** (25 mg, 0.11 mmol, 1.1 equiv.) and the gold catalyst (2.8 mg, 0.005 mmol, 5 mol %) were introduced in a flame-dried Schlenk tube under argon. The mixed solids and oils were placed under vacuum for 10 min and filled with argon prior to add the degassed acetonitrile (1.2 mL). The reaction mixture was stirred under blue light irradiation at room temperature for 24 h.

Supplementary Table 1 Solvent screening for the optimization

Entry	**1a**:**2a** Ratio	Base	Solvent	**3aa**, Yield (%)	**4a**, Yield (%)
**1**	1:1.1	K_2CO_3	Toluene	47	16
**2**	1:1.1	K_2CO_3	DCM	16	16
**3**	1:1.1	K_2CO_3	THF	11	17
**4**	1:1.1	K_2CO_3	MeCN	62	10

1a (0.1 mmol), **2a** (0.11 mmol), [Au-CF_3] (5 mol%), K_2CO_3 (2.5 equiv), degassed solvent, Blue LEDs, stirring for overnight, Yield determined by ^1H NMR by using 1,3,5-trimethoxybenzene as an internal standard.

b. Absorption and emission studies

The absorption spectra of various species involved in the reaction were recorded using acetonitrile as solvent.

Optical set-up: Absorption spectra were recorded on a Cary 50 (Varian) spectrophotometer equipped with a Peltier apparatus (298 K). Fluorescence spectra were recorded on a FP-6200 spectrofluorimeter (Jasco) equipped with a Peltier apparatus (298K).

Absorption spectra of various species

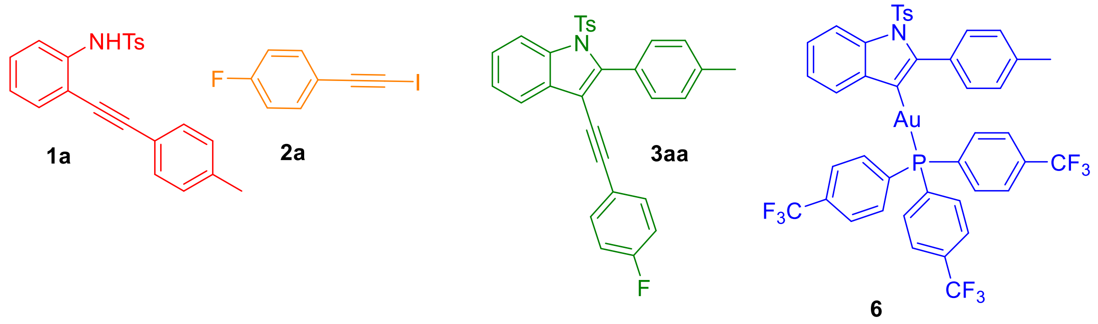

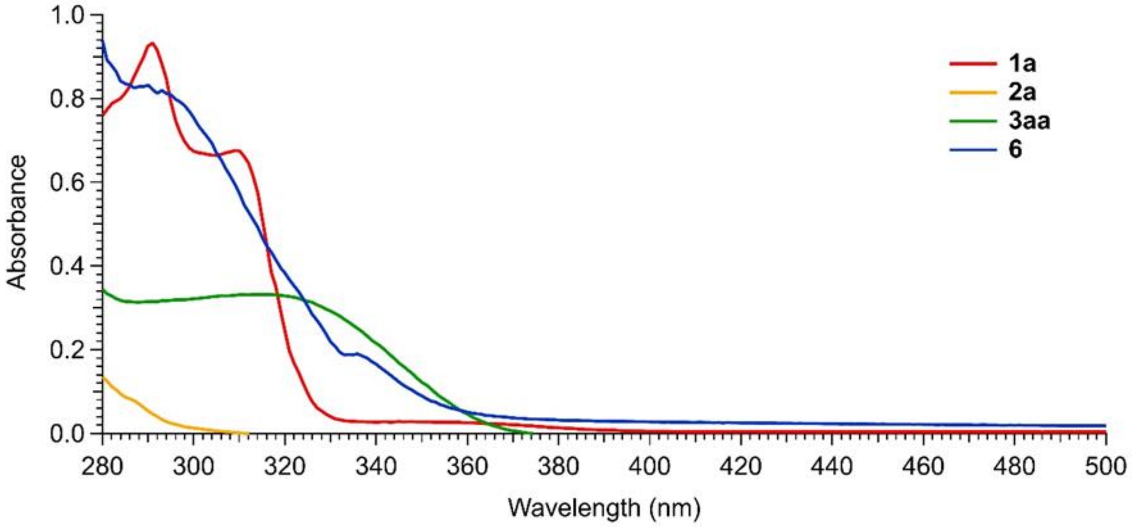

Supplementary Fig. 1 Absorption spectra of **1a** (red), **2a** (orange), **3aa** (green) and **6** (blue) in Ar-saturated acetonitrile for 50 µM solutions.

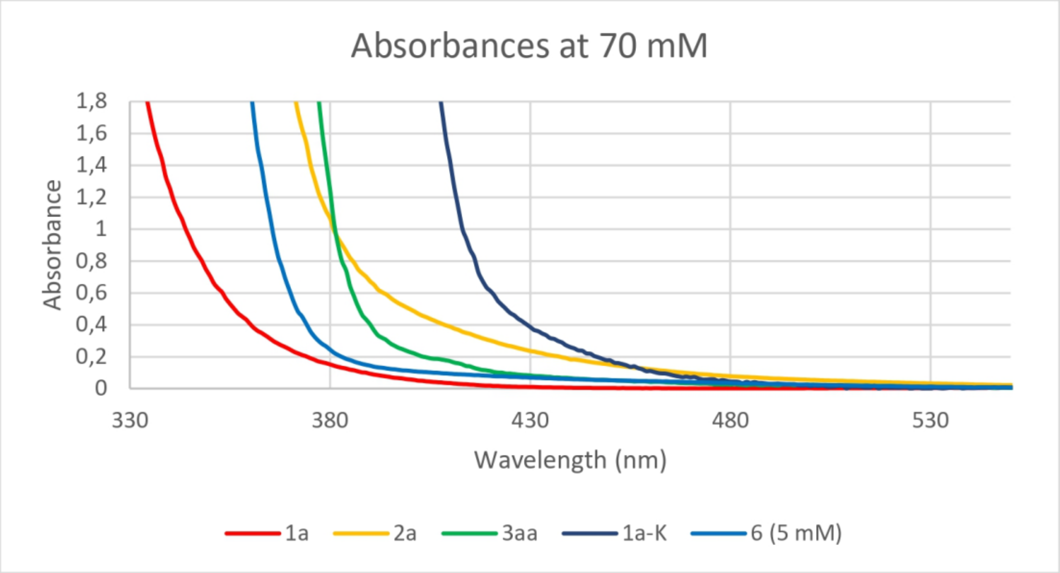

Supplementary Fig. 2 Absorption spectra of **1a** (red), **2a** (orange), **3aa** (green) and **6** (blue) in Ar-saturated acetonitrile for 70 mM for **1a**, **1a-K**, **2a** and **3aa** and 5 mM for **6**.

At high concentration, **1a**, **3**aa and **1a-K** are poorly soluble in the solvent and so a manual correction was necessary to render the baseline values to zero and only show their absorption properties in the reaction condition. However and despite these issues, absorption spectra have been recorded at 70 mM for **1a**, **1a-K**, **2a** and **3aa** and 5 mM for **6** and revealed a significant absorption at 450 nm of **1a-K** and **2a**. The other compounds seem unlikely to absorb the light of the LED.

The two vinylgold(I) intermediates **6** and **6-*O*** exhibit similar absorption properties. Of note, **6** presents a wide emission band centered on 410 nm in contrast to **6-*O***.[3]

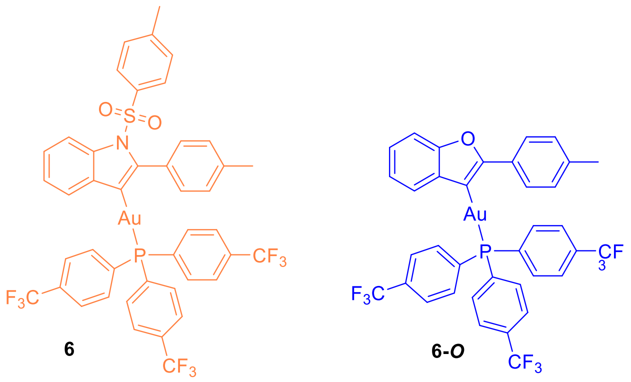

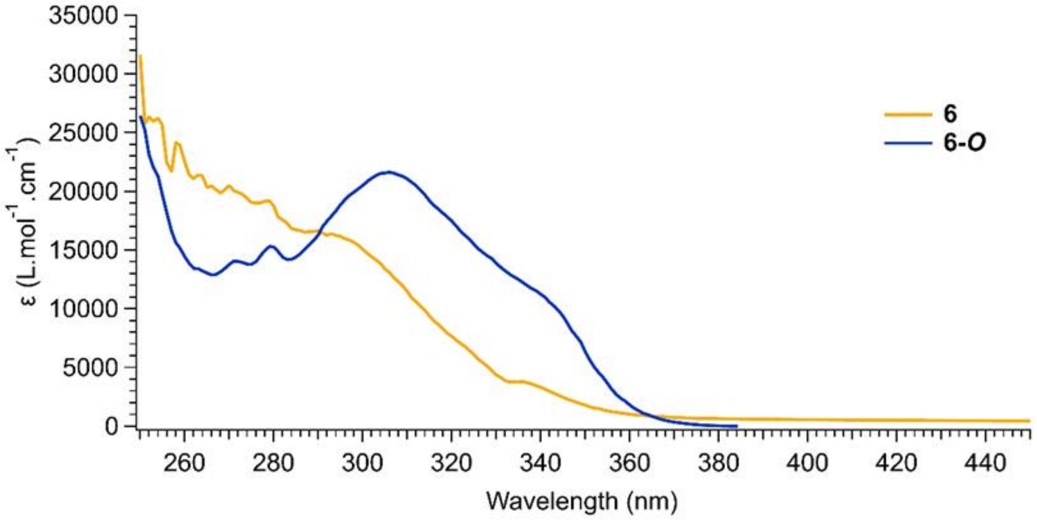

Supplementary Fig. 3 Absorption spectra of the vinylgold(I) complexes involved in the formation of the indole and the benzofuran in Ar-saturated acetonitrile. **6** in orange and **6-*O*** in blue.

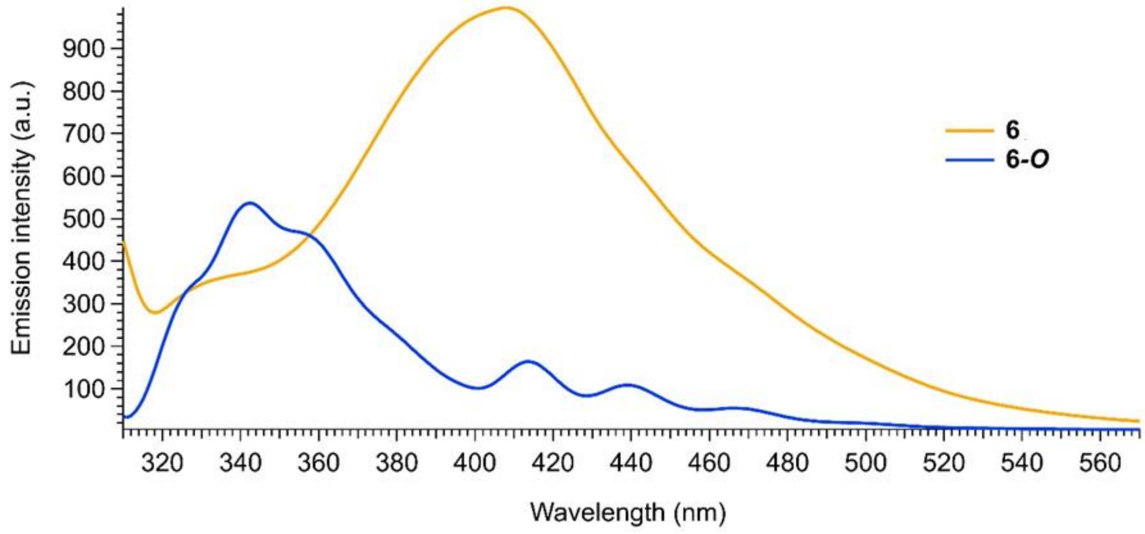

Supplementary Fig. 4 Emission spectra of **6** (orange) and **6-*O*** (blue) in Ar-saturated acetonitrile for 50 µM solutions at 300 nm.

Preparation of **1a-K** from the corresponding aniline (**1a**)

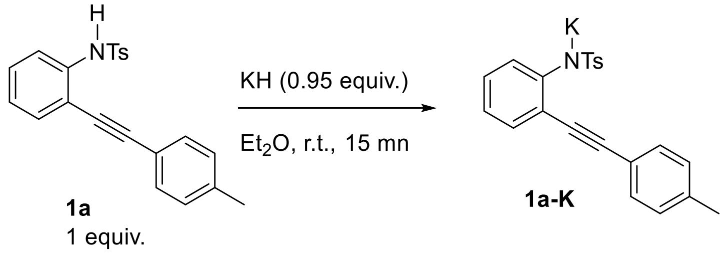

Potassium hydride in paraffin (40 % wt) (0.95 equiv., 0.14 mmol, 14.2 mg) was introduced in a flame-dried flask purged by three argon-vacuum cycles after coming back to room temperature. The hydride was washed three times with distilled petroleum ether to remove the grease and dried under vacuum for half an hour. **1a** (1 equiv., 0.14 mmol, 50 mg) in solution in distilled Et_2O (around 5 mL in saturated with argon) was added onto the hydride and stirred at room temperature for 10-30 min. The solvent was removed under vacuum and 14 mL of degassed acetonitrile (dried over molecular sieve) was added to provide the 10 mM stock solution $\mathbf{S_0}$. All the solutions used for the following experiments were prepared from dilution of $\mathbf{S_0}$.

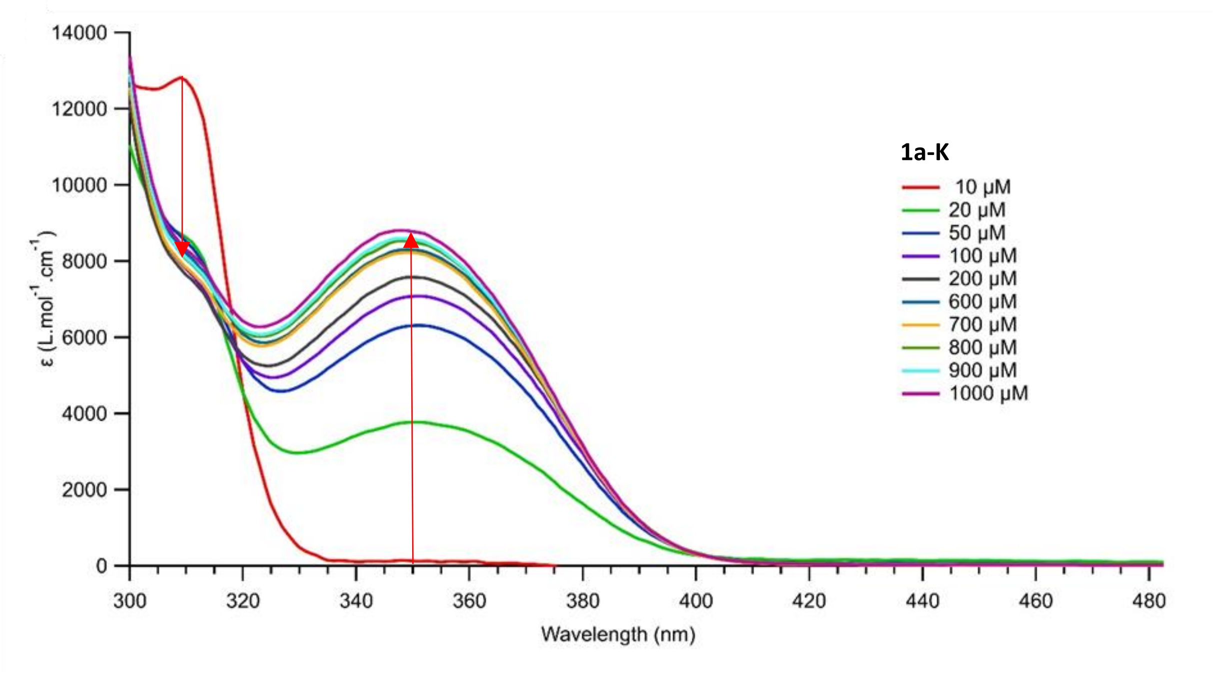

Supplementary Fig. 5 Absorption spectra for different concentrations in **1a-K** in acetonitrile.

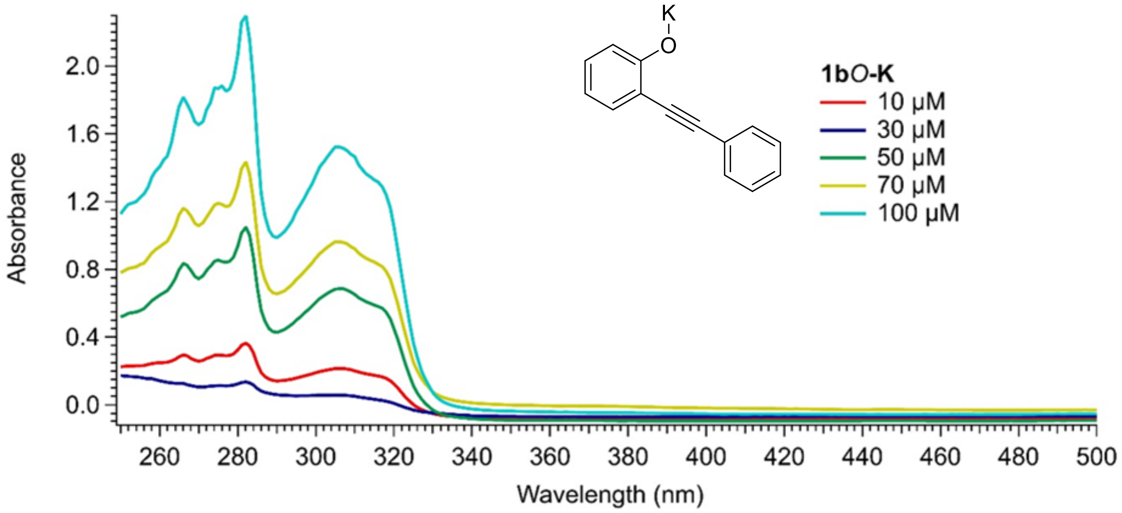

Supplementary Fig. 6 Absorption spectra for different concentrations in **1b*O*-K**.

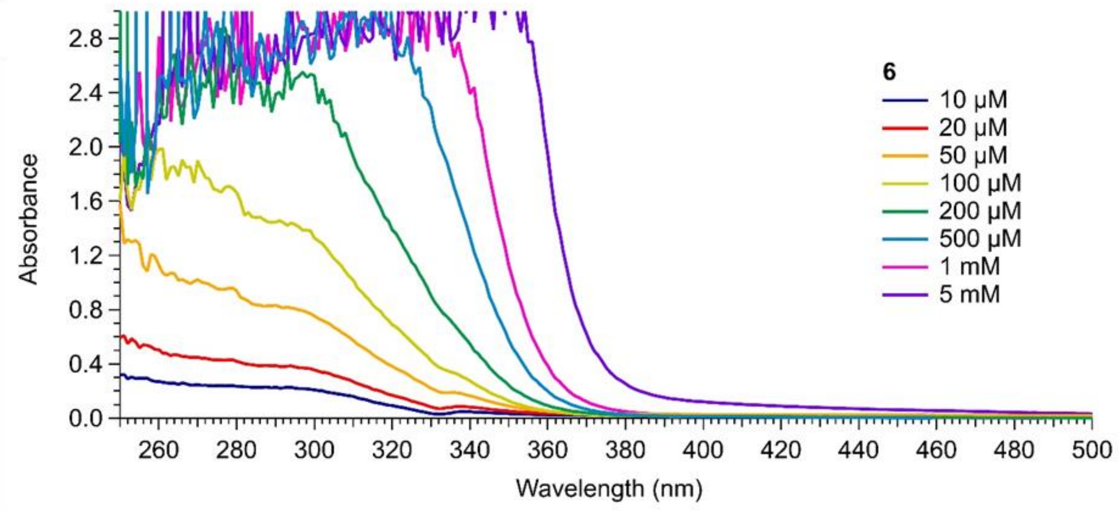

Supplementary Fig. 7 Absorption spectra of various solutions of **6** at different concentrations.

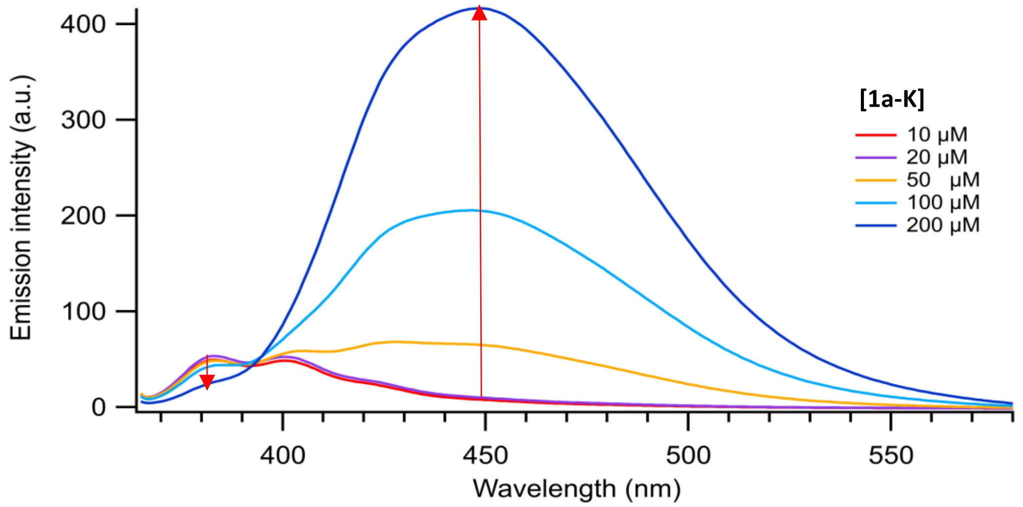

Supplementary Fig. 8 Emission spectra for different concentrations in **1a-K** in acetonitrile under light excitation at 350 nm.

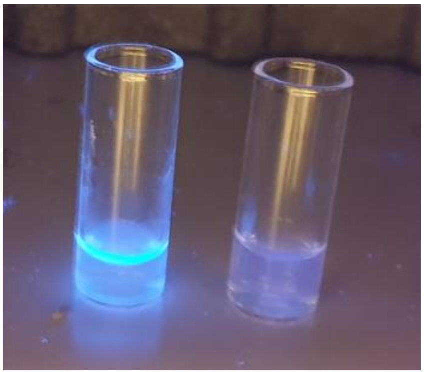

Supplementary Fig. 9 On the left, an aliquot of $\mathbf{S_0}$ and on the right, a solution of **1a** in acetonitrile excited at 365 nm.

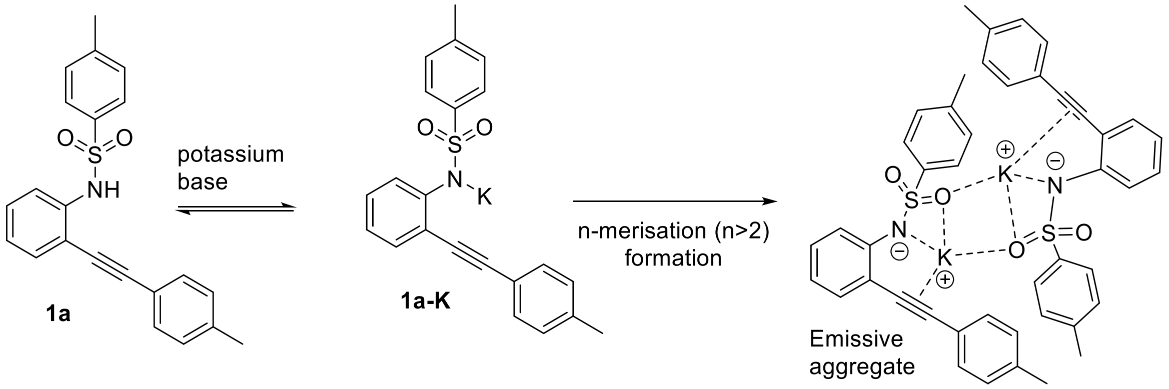

The analyses of Figure S4a and S5 suggest the formation of an emissive aggregate of **1a-K**, which is likely to absorb the light of the LED. Interestingly, no such emissive aggregate is formed in the case of the phenate **1b*O*-K**.

Influence of the iodoalkyne **2a** on the absorption of the aggregate of **1a-K**:

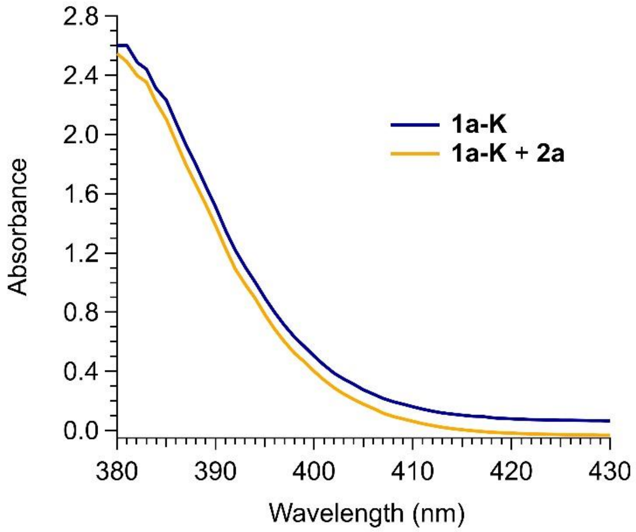

Supplementary Fig. 10 Absorbance of **1a-K** in the presence (blue) or not (orange) of one equivalent of **2a** at 1 mM in acetonitrile at 298 K.

c. NMR titration and bind-fitting experiments

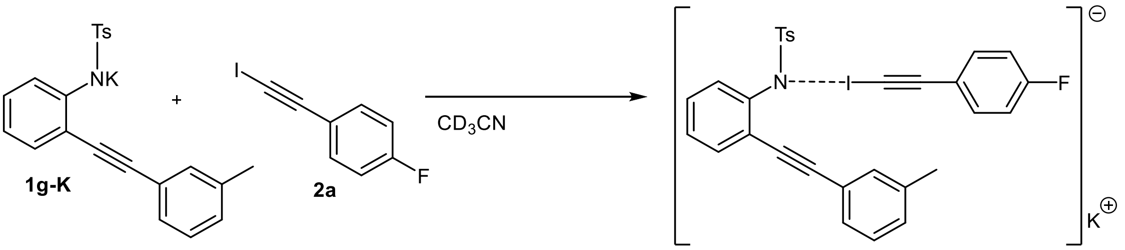

Procedures

Two solutions were prepared to carry on the NMR titration:

- Solution A (0.2 M in **2a**): The iodoalkyne (0.75 g, 3 mmol) was dissolved in CD_3CN (6 mL) with 10 mol % of fluoro-benzene as internal standard under argon.

-Solution B (**2a** (0.2 M) + **1g-K** (0.9M)): The aniline (0.64 g, 1.78 mmol) was deprotonated by potassium hydride (0.95 equiv.) in Et_2O (4 mL) at room temperature under argon and stirring. After 5 min, the solvent was removed and the solid was then dissolved in 2 mL of solution A.

To plot the following curves, different ratios of solutions A and B were mixed in an NMR tube. The overall volume was fixed at 550 µL and the concentration in halogen bond donor at 0.2 M.

^{1}H and ^{19}F NMR shifts due to the halogen bond

[1g-K]/[2b] =

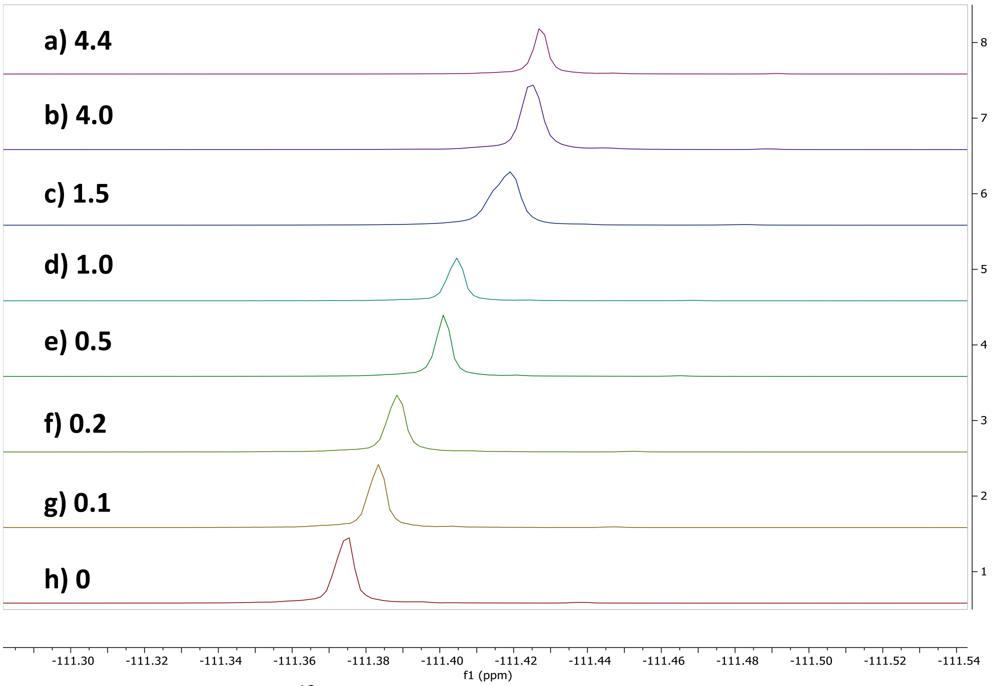

Supplementary Fig. 11 ^{19}F NMR spectra of mixture of solutions A and B with a [**1g-K**]/[**2b**] ratio varying from **a)** 4,4 down to **h)** 0.

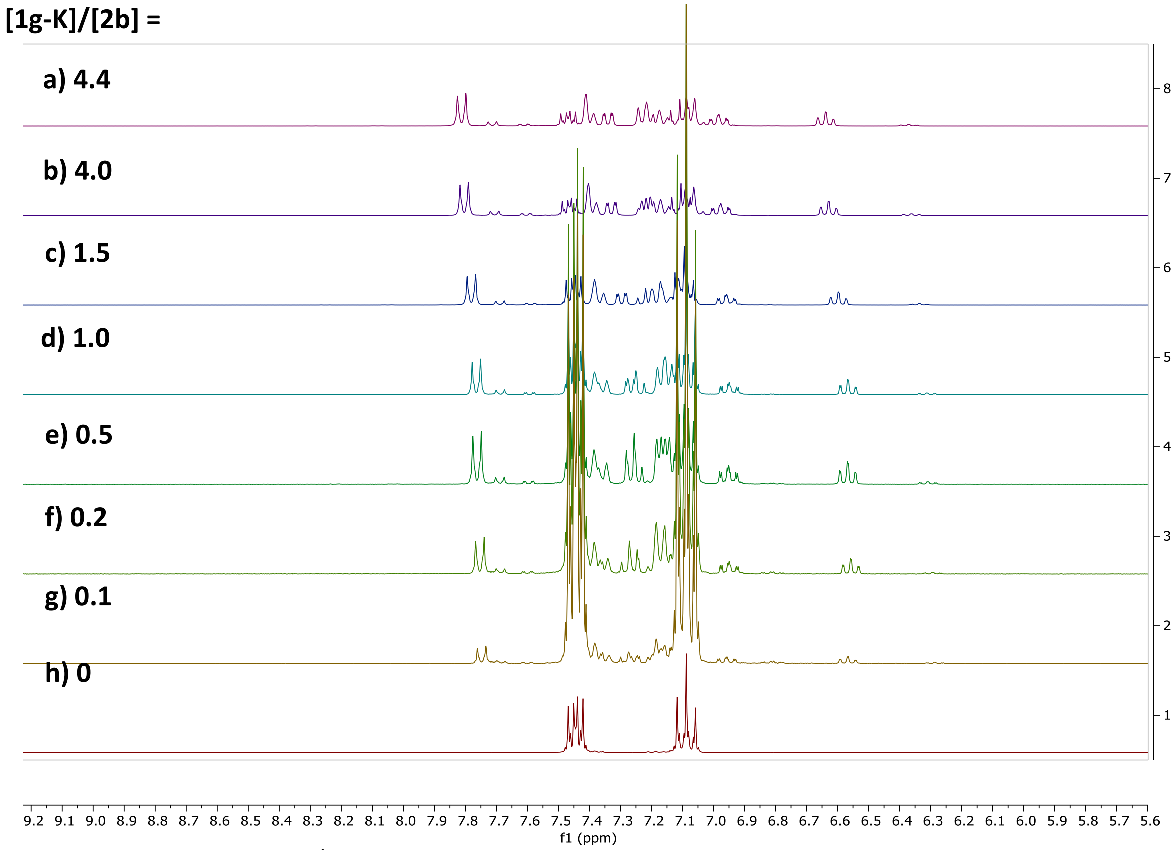

Supplementary Fig. 12 ^{1}H NMR spectra of mixture of solutions A and B with a [**1g-K**]/[**2b**] ratio varying from **a)** 4.4 down to **h)** 0.

We observed a significant shift for three signals linked to the sulfonamide moiety (the doublet at 7.7 ppm and the two triplets at 6.6 and 7.0 ppm), which is consistent with a halogen bond between the iodo-alkyne and the anion.

^{13}C NMR monitoring of the C_{sp}-I bond:

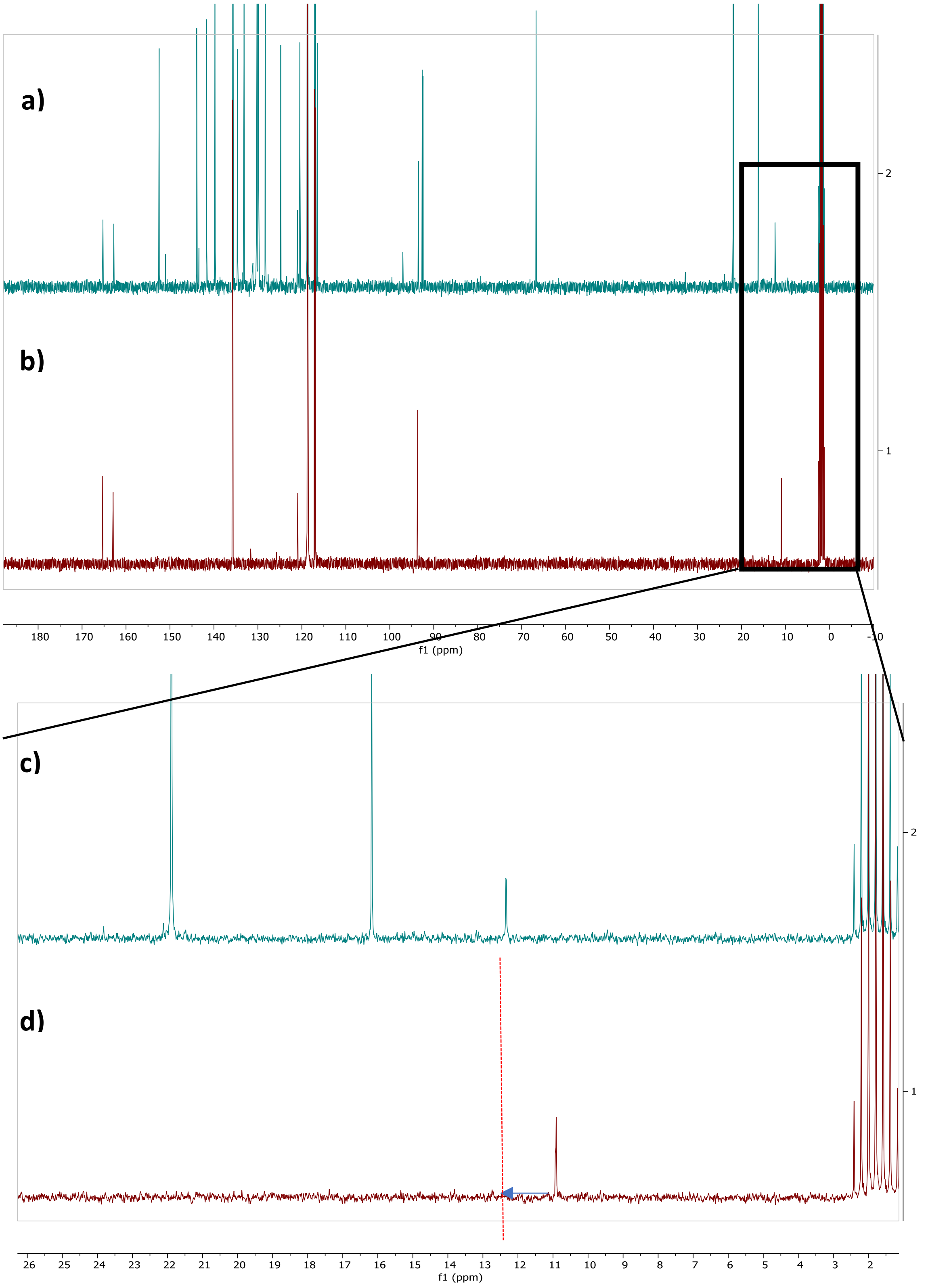

Supplementary Fig. 13 ^{13}C-NMR spectrum in CD_3CN of **a)** and **c)** the mixing of solutions A and B with a ratio [**1g-K**]/[**2b**] of 4,4 (-10 to 190 ppm shift range in **a** and 1 to 26.5 ppm shift range in **c**, respectively) and **b)** and **d)** the free alkyne and a (**1g-K + 2b**) solution.

The shift of the C_{sp}-I carbon of **2a** was displaced by 1.5 ppm in the presence of **1g-K** (**Supplementary Fig. 13 c) and d)**) is consistent with the formation of a non-covalent bond between the alkyne iodine and the anion.

^{19}F NMR titration of the non-covalent complex :

The previous chemical shift provided us the following data:

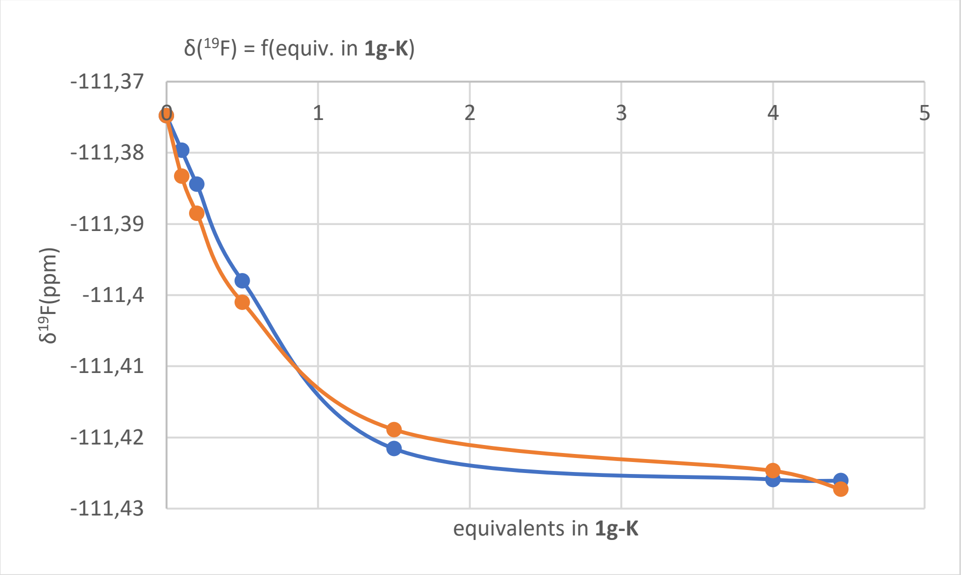

Supplementary Fig. 14 NMR titration at 298K of the halogen bond between **1g-K** and **2a**, in blue the theoretical curve[4-6] for a 1:1 association complex with an association constant of 69 M^{-1} in orange.

Theory-experiment gap analysis
Root Mean Squared Error: 0.2%
We used the free website designed by Stoddart[6] to fit the curve ($\delta^{19}F$ = f([**1g-K**]/[**2a**]) with a binding isotherm to obtain the association constant K_a^{298K} = 69 M^{-1}.

Influence of the halide on the non-covalent interaction with **1a-K** :

Case of **2a-Br** with **1a-K**

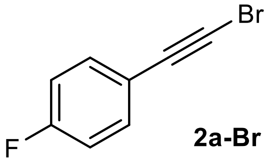

2a-Br

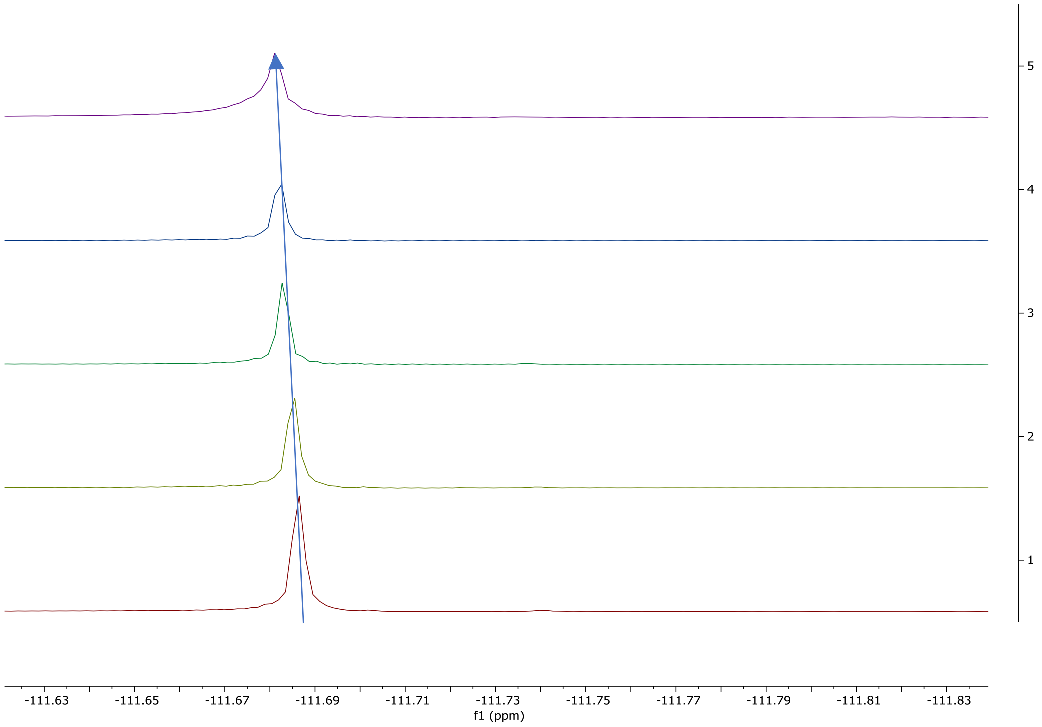

Supplementary Fig. 15 From line 1 to 5, ^{19}F NMR of a mixture of **2a-Br** with 0; 0,33; 0.67; 1 and 3 equivalents of **1a-K** in solution with **2a-Br** for an overall concentration [**2a-Br**]+[**1a-K**] = 0.2M

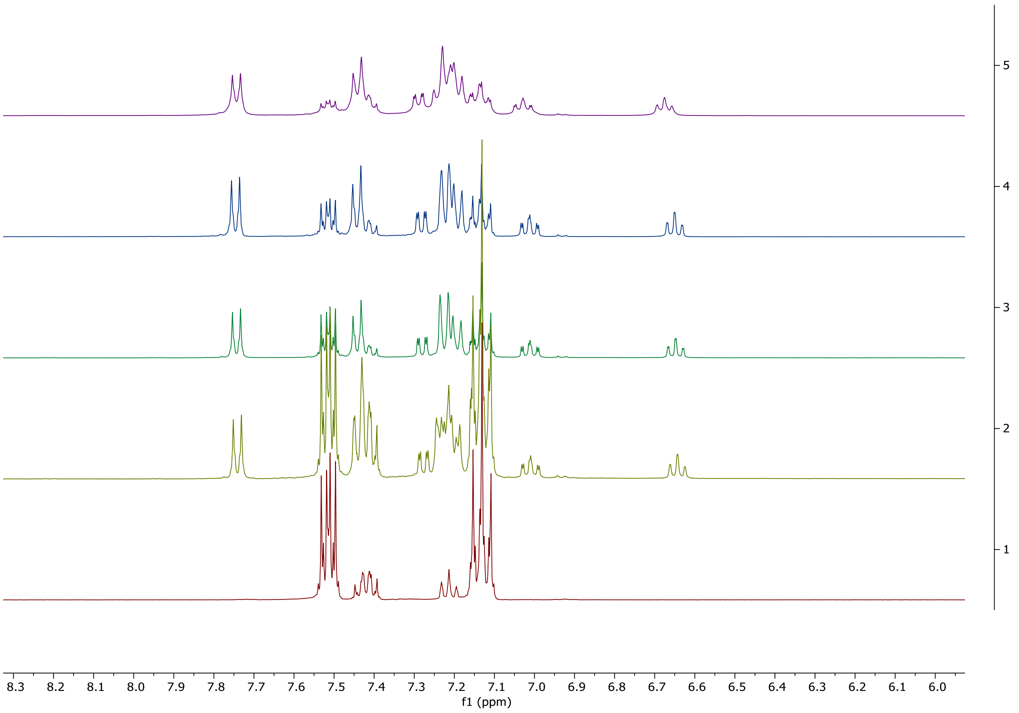

Supplementary Fig. 16 From line 1 to 5, ^{1}H NMR of a mixture of **2a-Br** with 0; 0,33; 0.67; 1 and 3 equivalents of **1a-K** in solution with **2a-Br** for an overall concentration [**2a-Br**]+[**1a-K**] = 0.2M

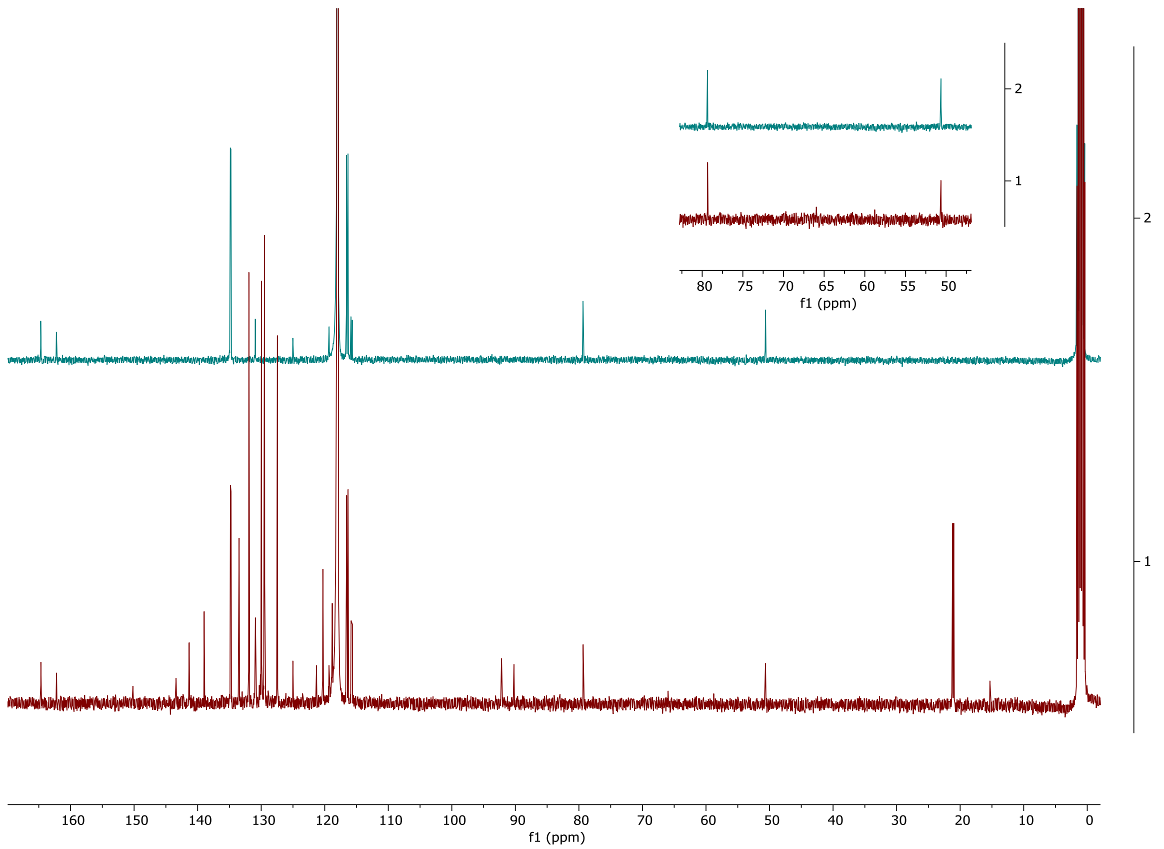

Supplementary Fig. 17 ^{13}C NMR of **2a-Br** alone (line 2) and a stoechiometric mixture of **2a-Br** with **1a-K** for an overall concentration [**2a-Br**]+[**1a-K**] = 0.2M

Case of **2a-Cl** with **1a-K**

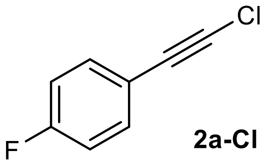

2a-Cl

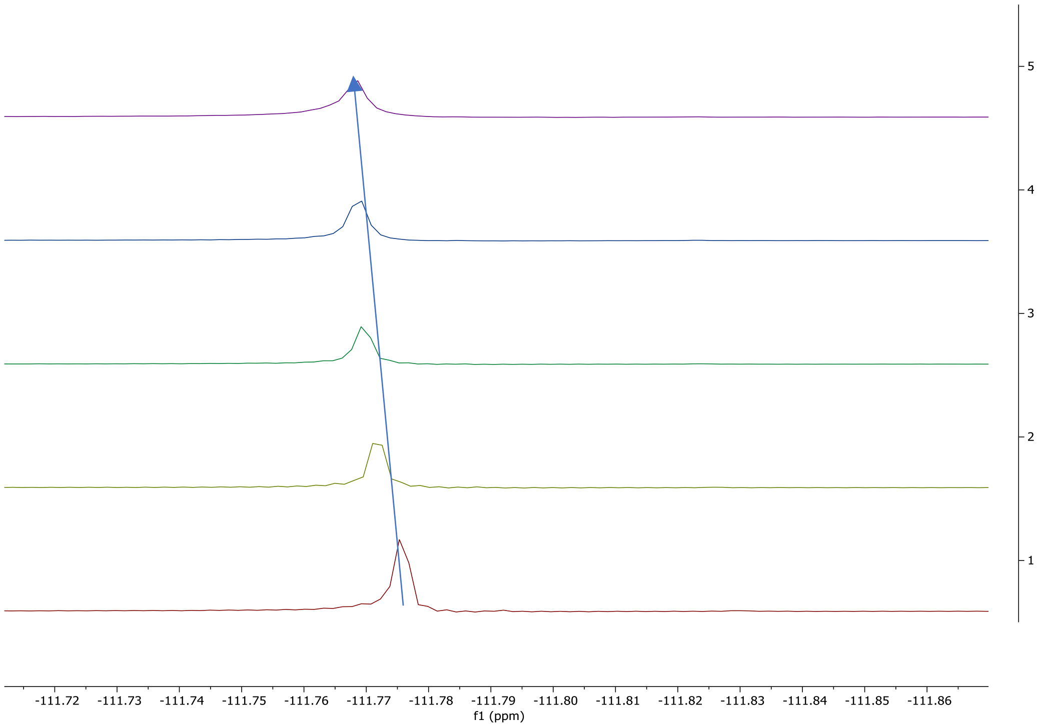

Supplementary Fig. 18 From line 1 to 5, ^{19}F NMR 0; 0,33; 0.67; 1 and 3 equivalents of **1a-K** in solution with **2a-Cl** for an overall concentration [**2a-Cl**]+[**1a-K**] = 0.1M

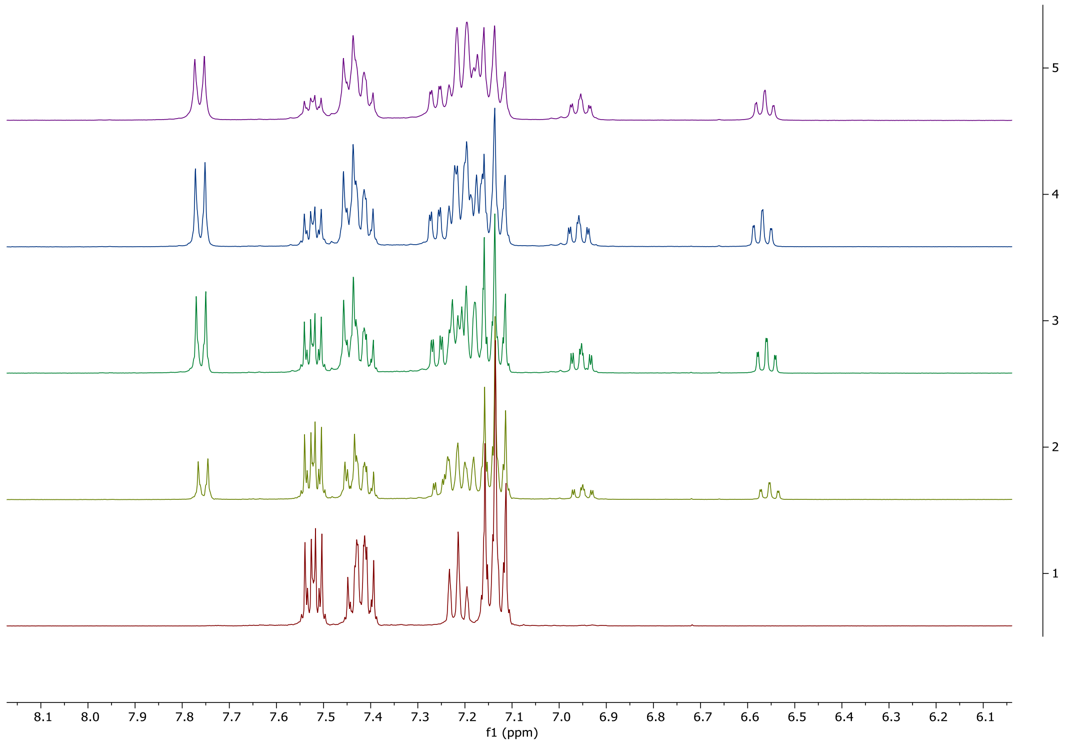

Supplementary Fig. 19 From line 1 to 5, ^{1}H NMR of a mixture of **2a-Cl** with 0; 0,33; 0.67; 1 and 3 equivalents of **1a-K** in solution with **2a-Cl** for an overall concentration [**2a-Cl**]+[**1a-K**] = 0.1M

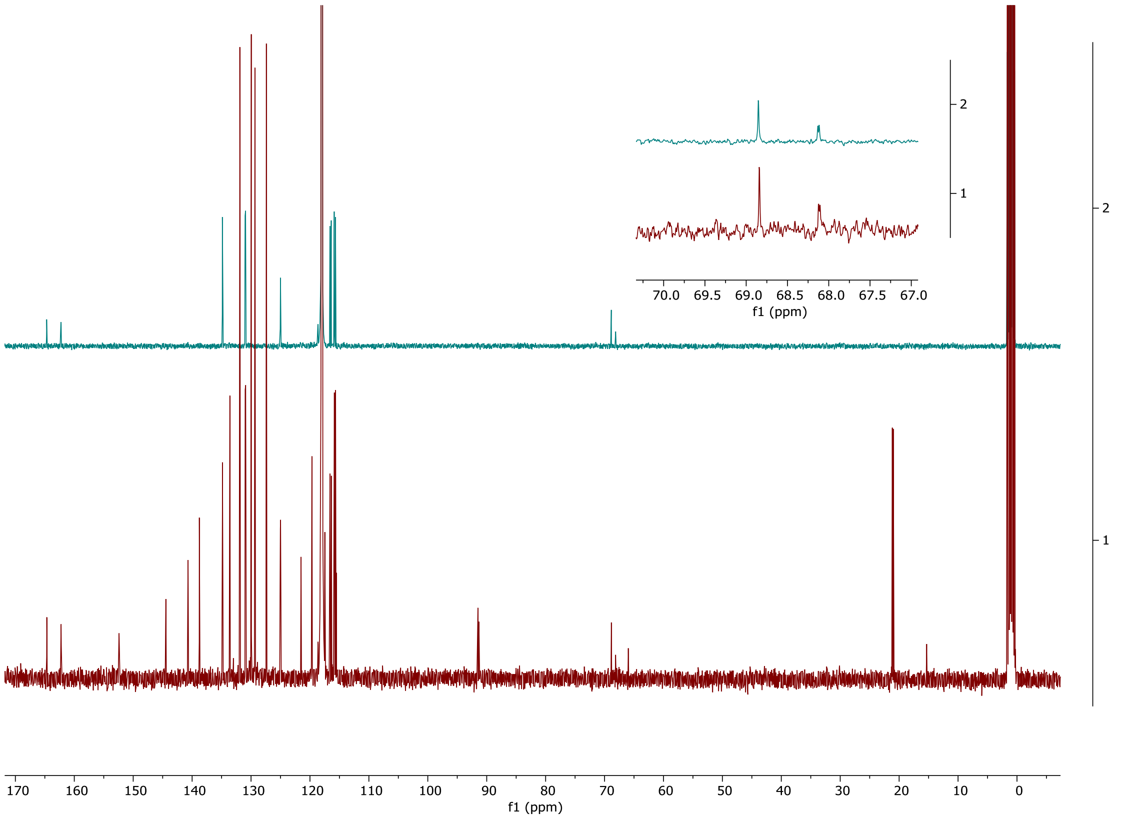

Supplementary Fig. 20 ^{13}C NMR of **2a-Cl** alone (line 2) and a stoechiometric mixture of **2a-Cl** with **1a-K** for an overall concentration [**2a-Cl**]+[**1a-K**] = 0.1M

<u>Case of **2a** with **1a-K**</u>

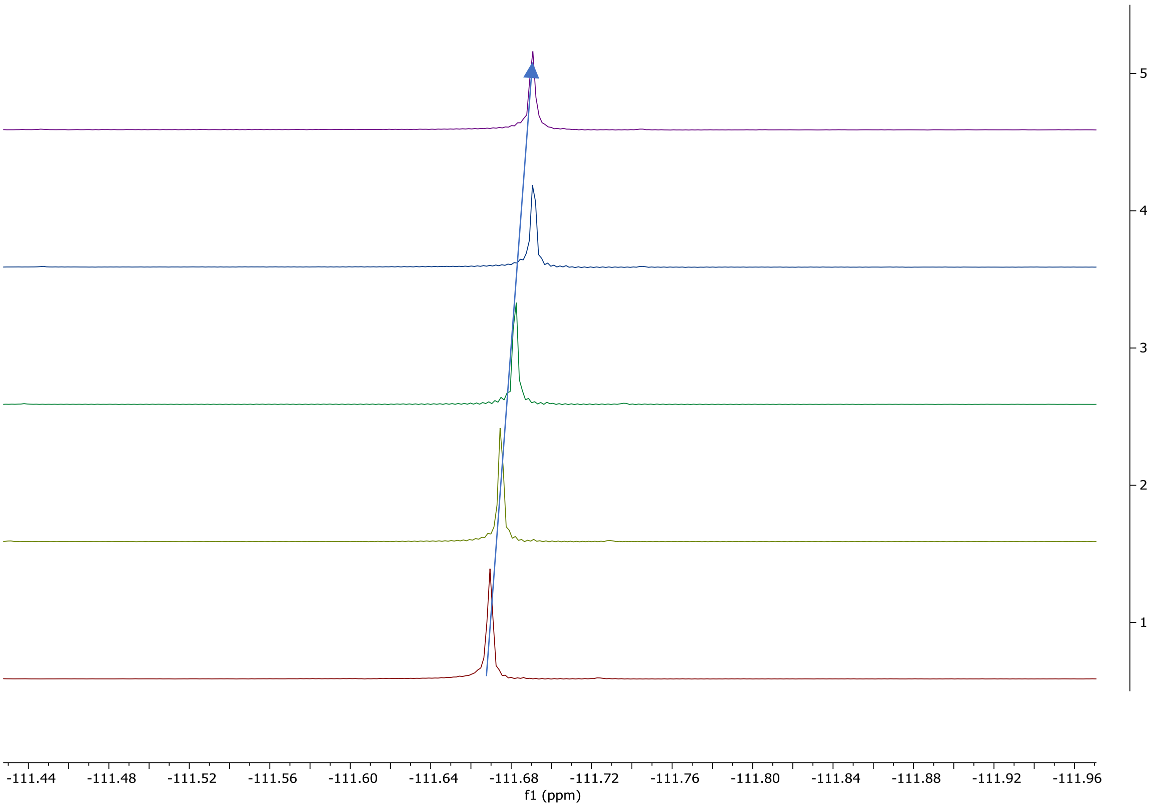

Supplementary Fig. 21 From line 1 to 5, ^{19}F NMR of a mixture of **2a** with 0; 0,25; 0.43; 1 and 1.5 equivalents of **1a-K** in solution with **2a** for an overall concentration [**2a**]+[**1a-K**] = 0.1M

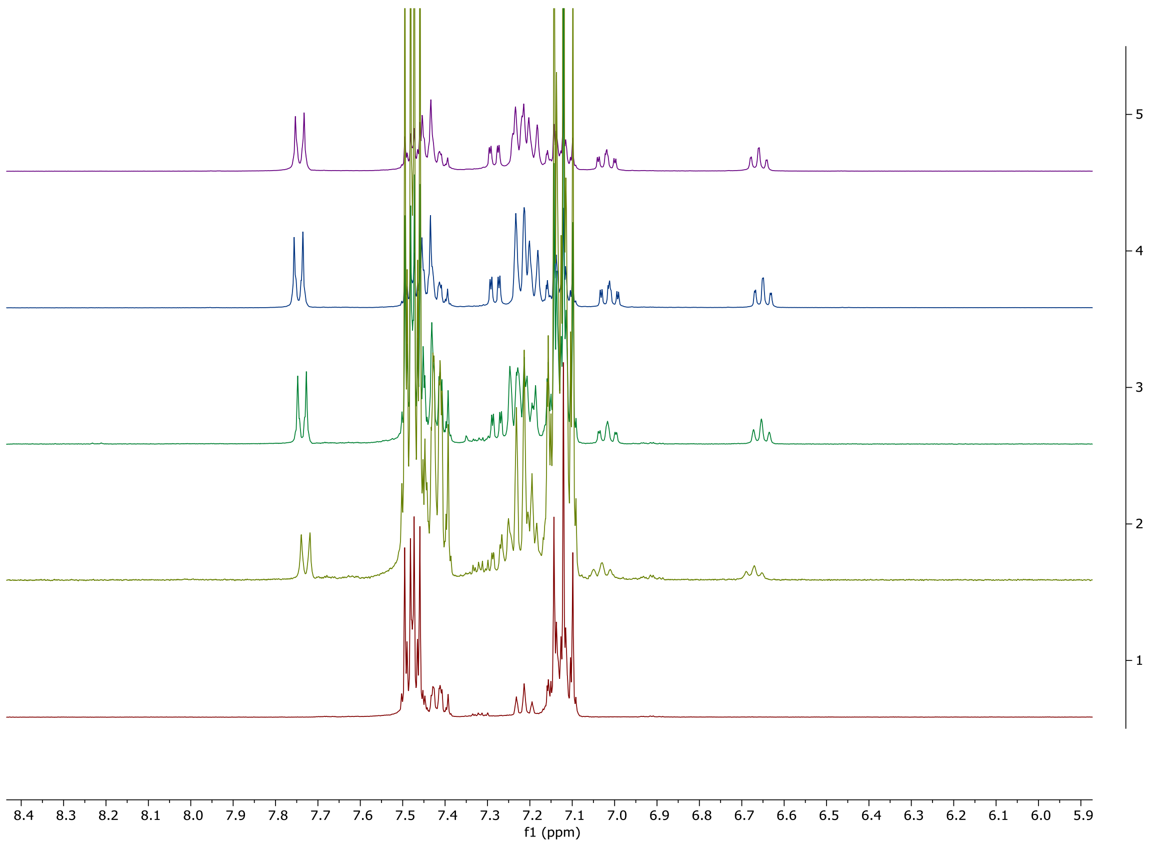

Supplementary Fig. 22 From line 1 to 5, ^{1}H NMR of a mixture of **2a** with 0; 0,25; 0.43; 1 and 1.5 equivalents of **1a-K** in solution with **2a** for an overall concentration [**2a**]+[**1a-K**] = 0.1M

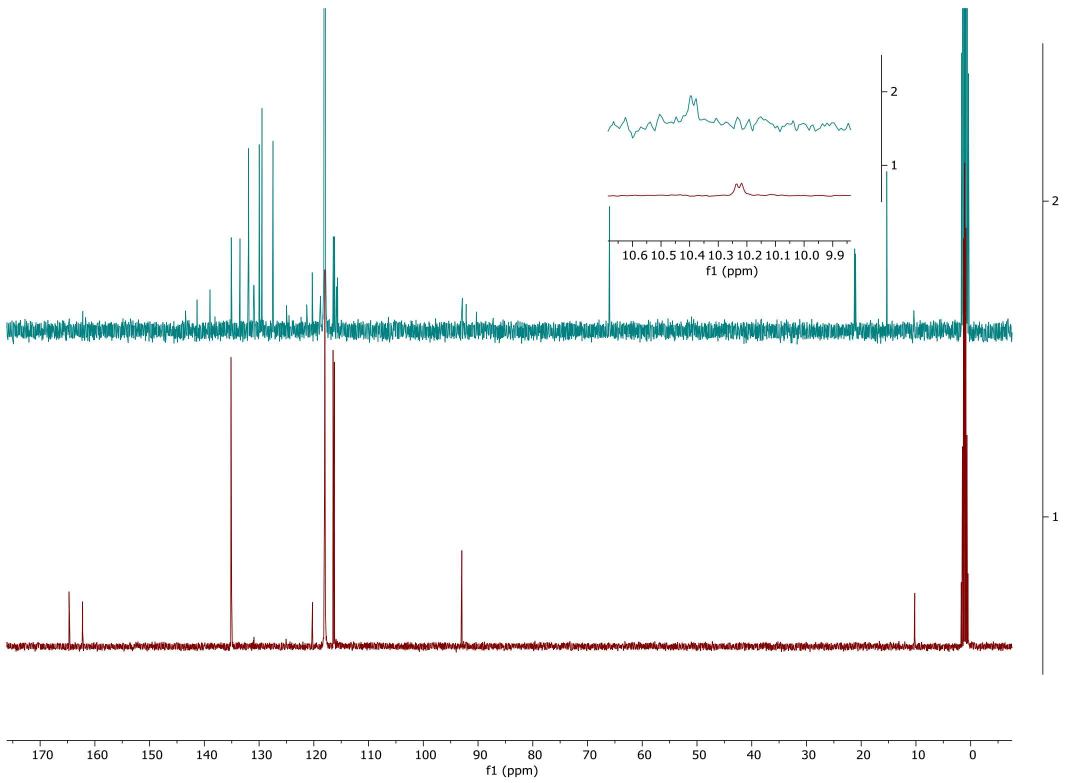

Supplementary Fig. 23 ^{13}C NMR of **2a-Cl** alone (line 2) and a stoechiometric mixture of **2a-Cl** with **1a-K** for an overall concentration [**2a-Cl**]+[**1a-K**] = 0.1M

Influence of the nature of the halogen on the non-covalent interaction with **1a-K**

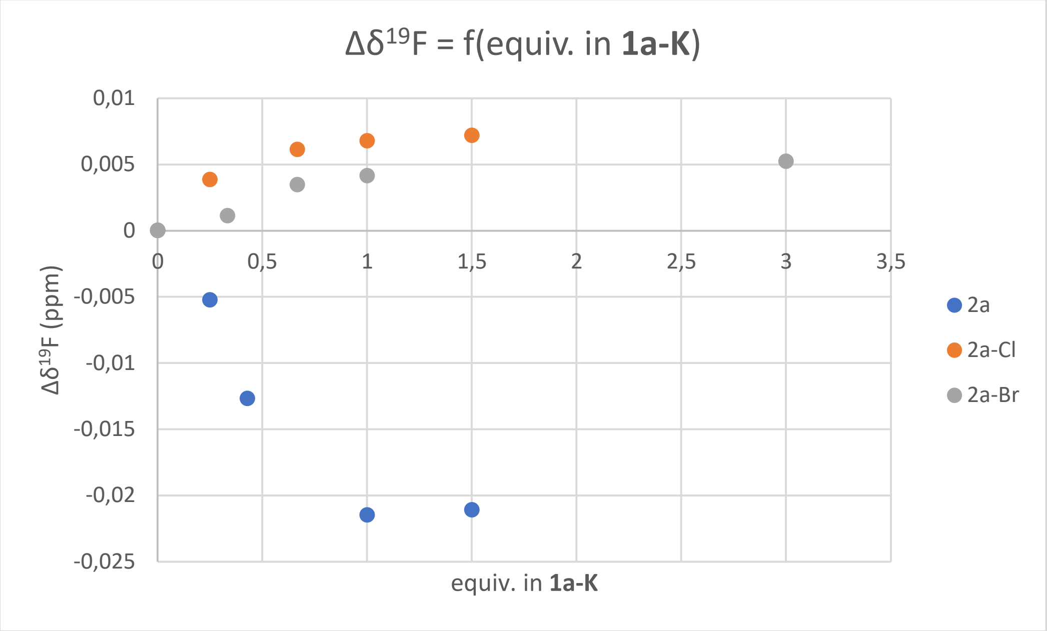

Supplementary Fig. 24 ^{19}F NMR titration plots for the halogen bonds between **1a-K** with **2a**, **2a-Cl** and **2a-Br**

Alkynes **2a-Br** and **2a-Cl** interact with the amide salt however, contrary to **2a**, the ^{19}F resonance is deshielded by this interaction and no significant shift is observed in ^{13}C NMR for the C_{sp}-X, which suggests a different type of interaction than for the iodinated alkyne. A plausible explanation is a side π-interaction between **1a-K** and the alkynyl halide (through its aromatic ring or its triple bond). The carbon halogen bond would be less activated/weakened than for **2a**, which would explain their difference of reactivity.

d. Stoichiometric experiments with vinylgold(I) 6

Procedures:

Preparations of the stock solutions:

- Solution A: **2a** (25 mg, 0.1 mmol) was dissolved in 1 mL of CD_3CN to provide a solution at 100 mM. The solution A was obtained by diluting the previous solution 10 times (100 µL with 900 µL of CD_3CN). $[\mathbf{2a}]_A$= 10 mM

Preparation of the reaction mixture:

In a flame-dried NMR tube capped with an NMR septum and purged by three argon-vacuum cycles, 3.6 mg of **6** (0.004 mmol) , trimethoxybenzene (9 mg, 0.053 mmol) and fluorobenzene (2 µL, 0.021 mmol) were dissolved in 350 µL of CD_3CN. 350 µL of the solution A were added so that: $[\mathbf{6}]_{t=0} = [\mathbf{2a}]_{t=0}$ = 5 mM. Protect the tube from the light. The reaction is monitored by ^1H and ^{19}F NMR at 0 min, 10 mn, 20 min, 30 min, 1.5h, 3h and 16h of irradiation at the blue LED.

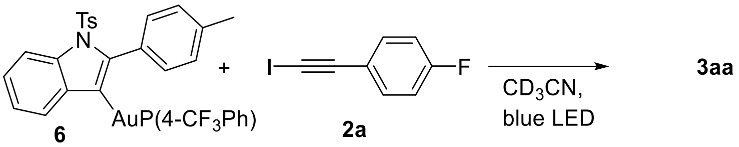

NMR yields of **3aa**[a]

c \ t	10 min	20 min	0.5 h	1 h	1.5 h	3 h
5 mM[b]	0	–	0	–	–	0[d]
5 mM[b,c]	17%	37%	45%	63%	77%	**quant.**
20 mM	50%	66%	76%	–	**92%**	–

[a]Determined by ^1H NMR using 1,3,5-trimethoxybenzene as internal standard ; [b] Concentration of **2a**: 5 mM;
[c] In the presence of 0.5 equiv of **1a-K**; [d] 0% after 16h

Supplementary Fig. 25 Reaction of **6** with **2a** under blue LED irradiation in different conditions.

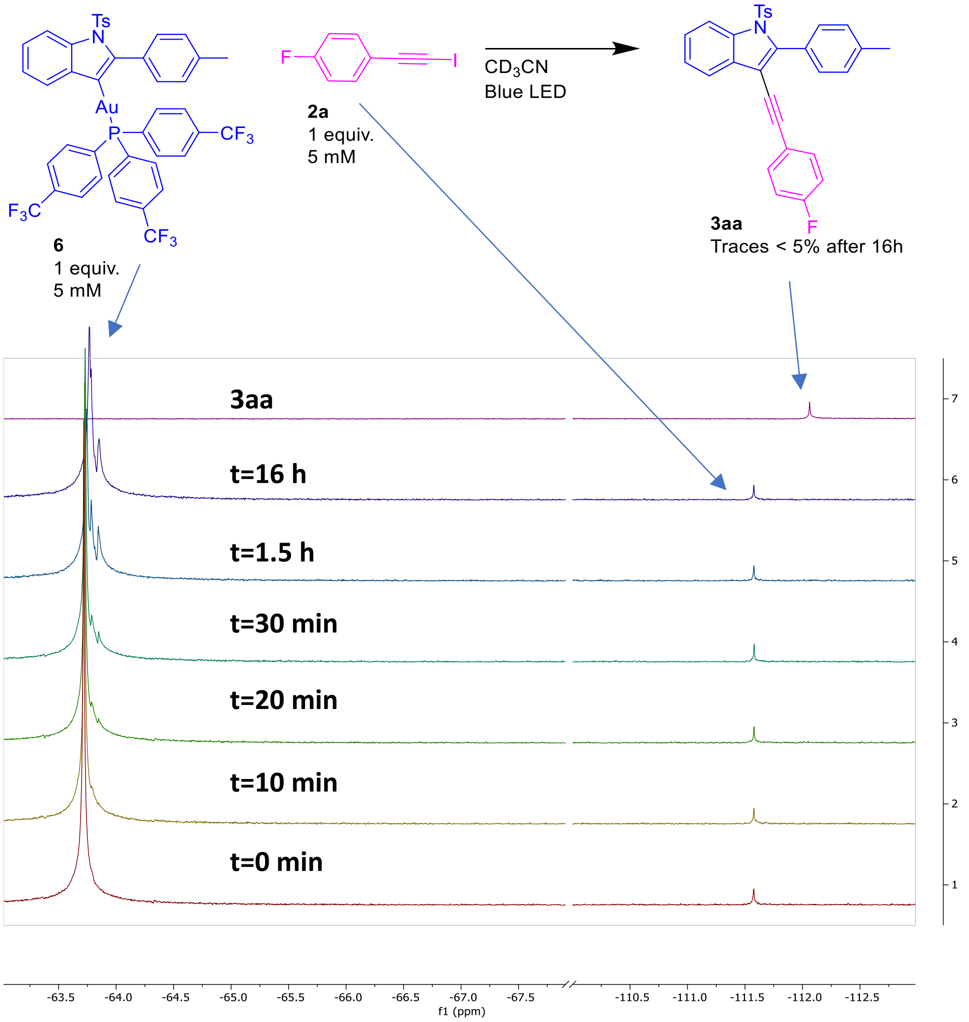

Supplementary Fig. 26 ^{19}F NMR of the mixture after 0 min (line 1), 10 min (line 2), 20 min (line 3), 30 min (line 4), 1.5 h (line 5), and 16 h (line 6) of irradiation, the spectrum of **3aa** (line 7) as a reference.

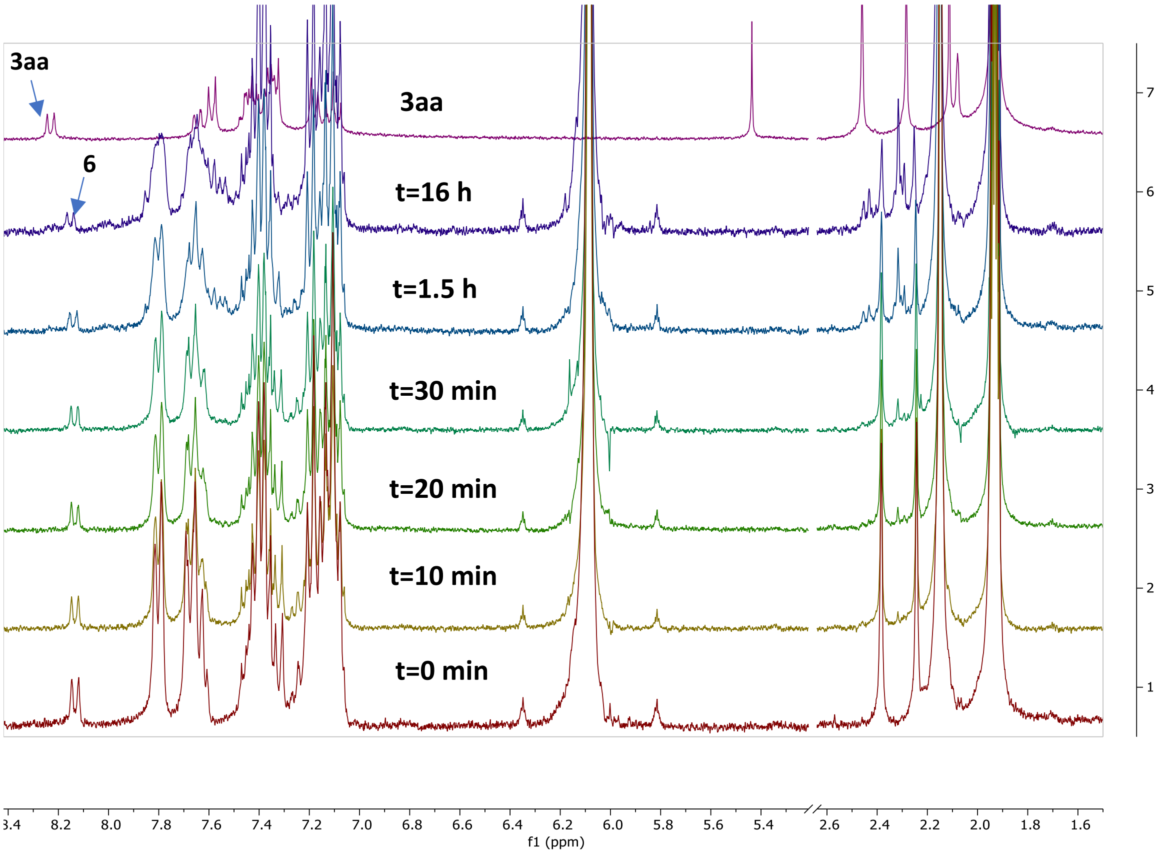

Supplementary Fig. 27 ^{1}H NMR of the mixture after 0 min (line 1), 10 min (line 2), 20 min (line 3), 30 min (line 4), 1.5 h (line 5), and 16 h (line 6) of irradiation, the spectrum of **3aa** (line 7) as a reference.

Influence of **1a-K**

Procedures:

Preparations of the stock solutions:

- Solution A: **2a** (25 mg, 0.1 mmol) was dissolved in 1 mL of CD_3CN to provide a solution at 100 mM. $[\mathbf{2a}]_A$= 100 mM

-Solution B: **1a** (18 mg, 0.05 mmol) was deprotonated in the presence of (washed) potassium hydrid (39% wt in parrafin, 1 equiv., 5 mg, 0.05 mmol) in diethyl ether under argon. After appearance of a white precipitate, the solvent was removed under vacuum and 1 mL of CD_3CN was added. For the resulting mixture, $[\mathbf{1a\text{-}K}]_B$ = 50 mM.

-Solution C: 100 µL of the solution A, 100 µL of the solution B were diluted with 800 µL of CD_3CN under argon. $[\mathbf{1a\text{-}K}]_C$ = 5 mM and $[\mathbf{2a}]_C$ = 10 mM

Preparation of the reaction mixture:

In a flame-dried NMR tube capped with an NMR septum and purged by three argon-vacuum cycles, 3.6 mg of **6** (0.004 mmol), trimethoxybenzene (8.5 mg, 0.05 mmol) and fluorobenzene (2 µL, 0.021 mmol) were dissolved in 350 µL of CD_3CN. 350 µL of the solution C were added. Protect the tube from the light. The reaction is monitored by ^1H and ^{19}F NMR at 0 min, 10 min, 20 min, 30 min, 1h, 1.5h min, 3h and 16h of irradiation at the blue LED. $[\mathbf{1a\text{-}K}]_{t=0}$ = 2.5 mM and $[\mathbf{6}]_{t=0}$ = $[\mathbf{2a}]_{t=0}$ = 5 mM.

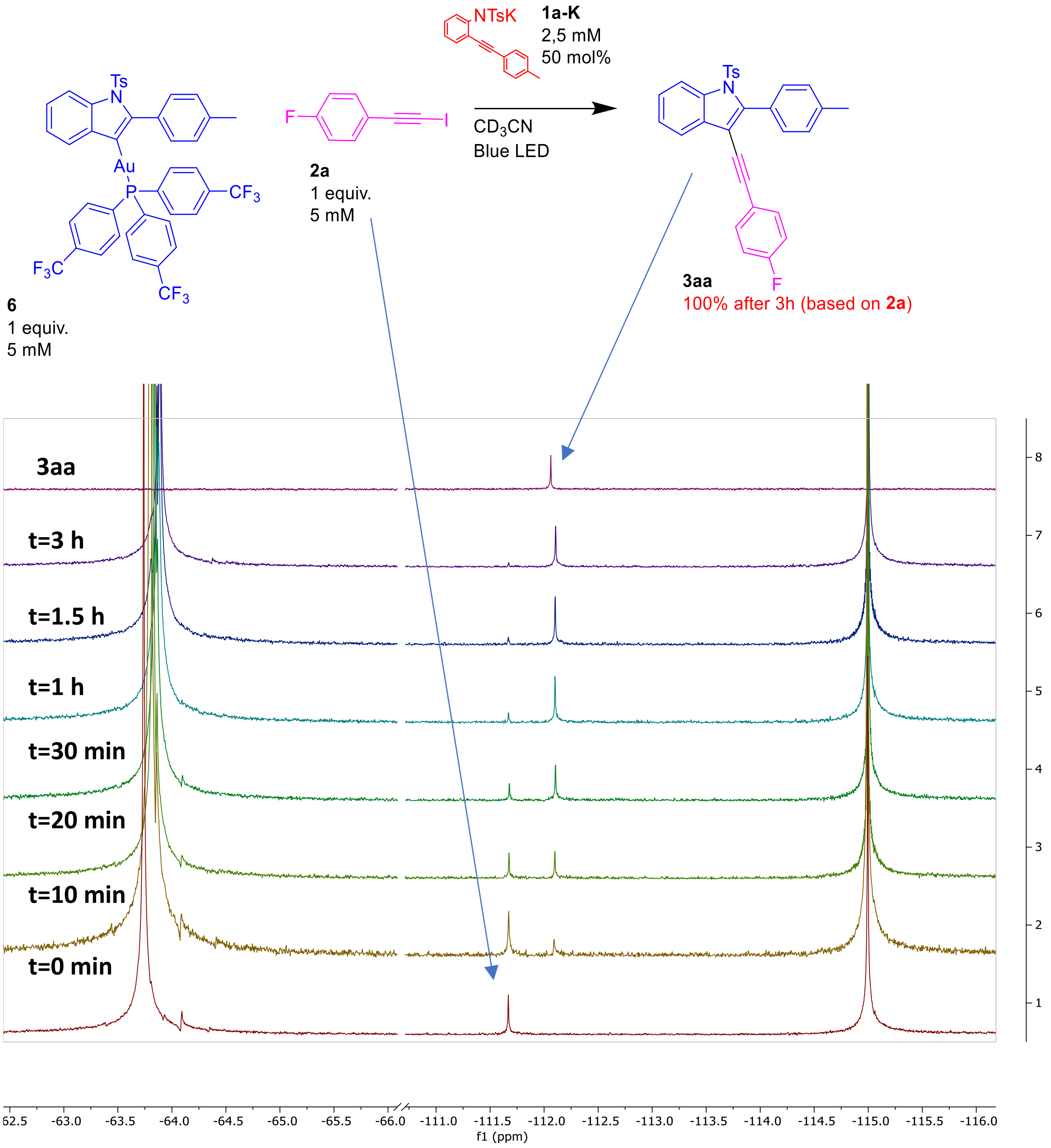

Supplementary Fig. 28 ^{19}F NMR of the mixture after 0 min (line 1), 10 min (line 2), 20 min (line 3), 30 min (line 4), 1 h (line 5), 1.5 h (line 6), and 3 h (line 7) of irradiation, the spectrum of **3aa** (line 8) as a reference.

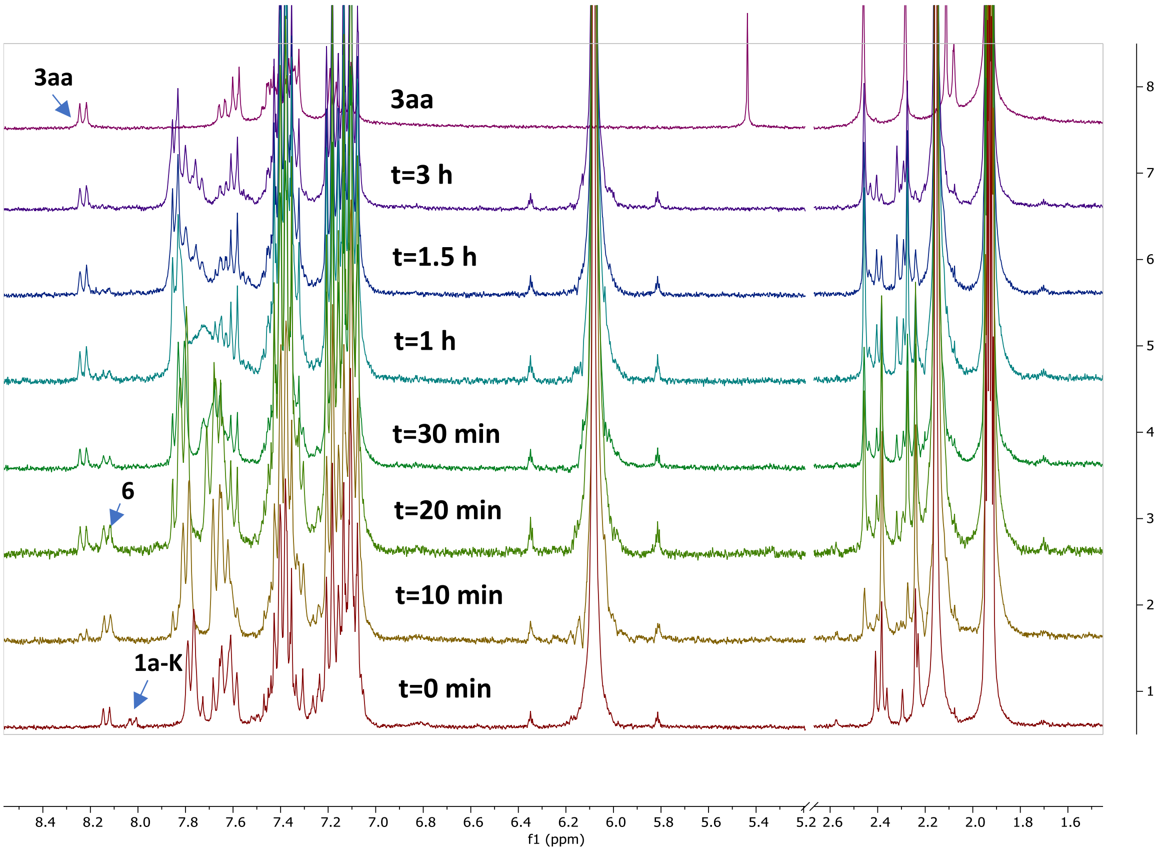

Supplementary Fig. 29 ^{1}H NMR of the mixture after 0 min (line 1), 10 min (line 2), 20 min (line 3), 30 min (line 4),1 h (line 5), 1.5 h (line 6), and 3 h (line 7) of irradiation, the spectrum of **3aa** (line 8) as a reference.

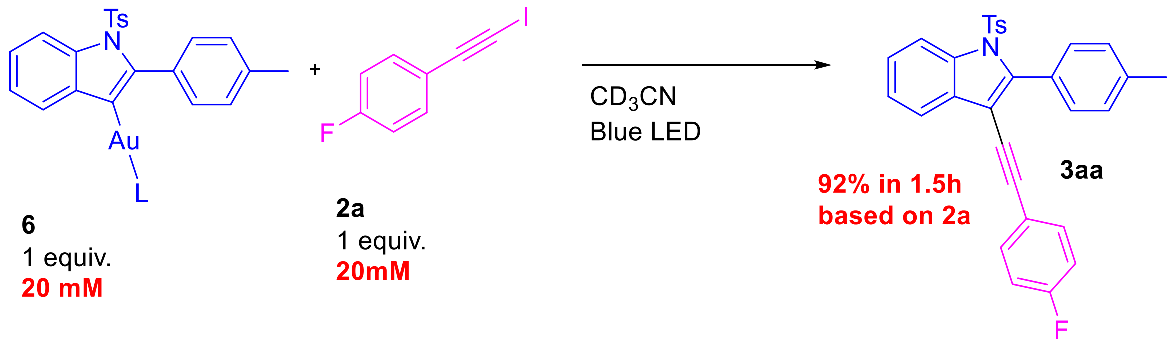

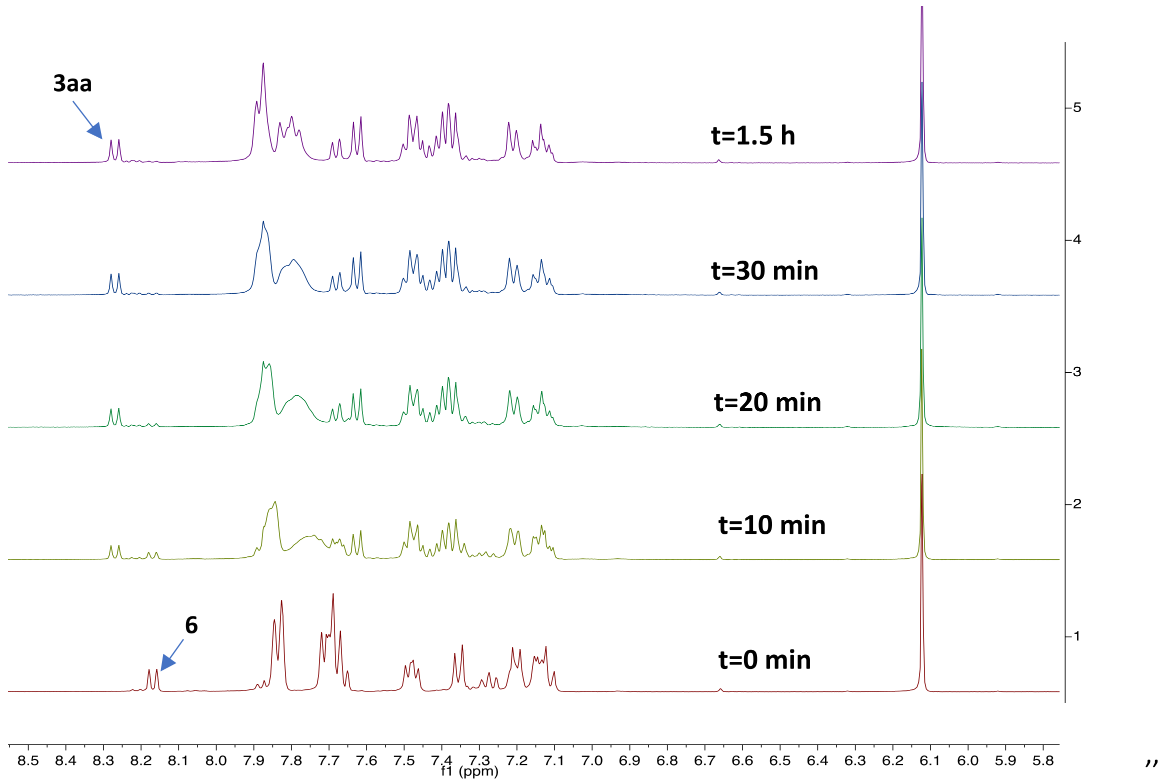

Supplementary Fig. 30 ^{1}H NMR of the mixture after 0 min (line 1), 10 min (line 2), 20 min (line 3), 30 min (line 4), 1.5 h (line 5) of irradiation.

e. Quenching experiments

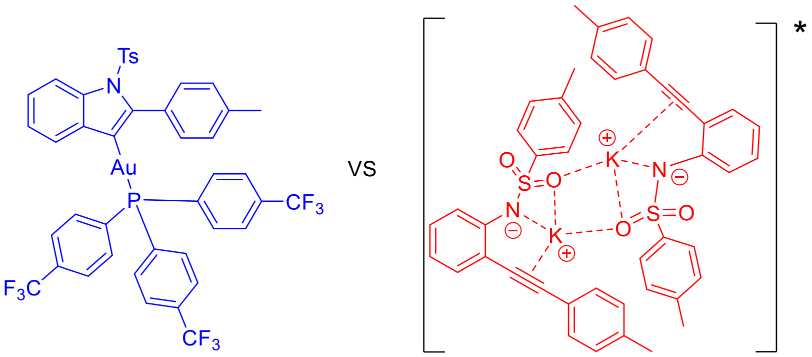

6 **1a-K**

The quenching of the luminescence of **1a-K** in the presence of **6** has been probed in acetonitrile. Different solutions of **1a-K** (50 µM at which aggregate **1a-K** is formed) with an increasing amount in **6** were prepared and their emission spectra were recorded. 380 nm was chosen as excitation wavelength to selectively excite the aggregate **1a-K** and not **6**.

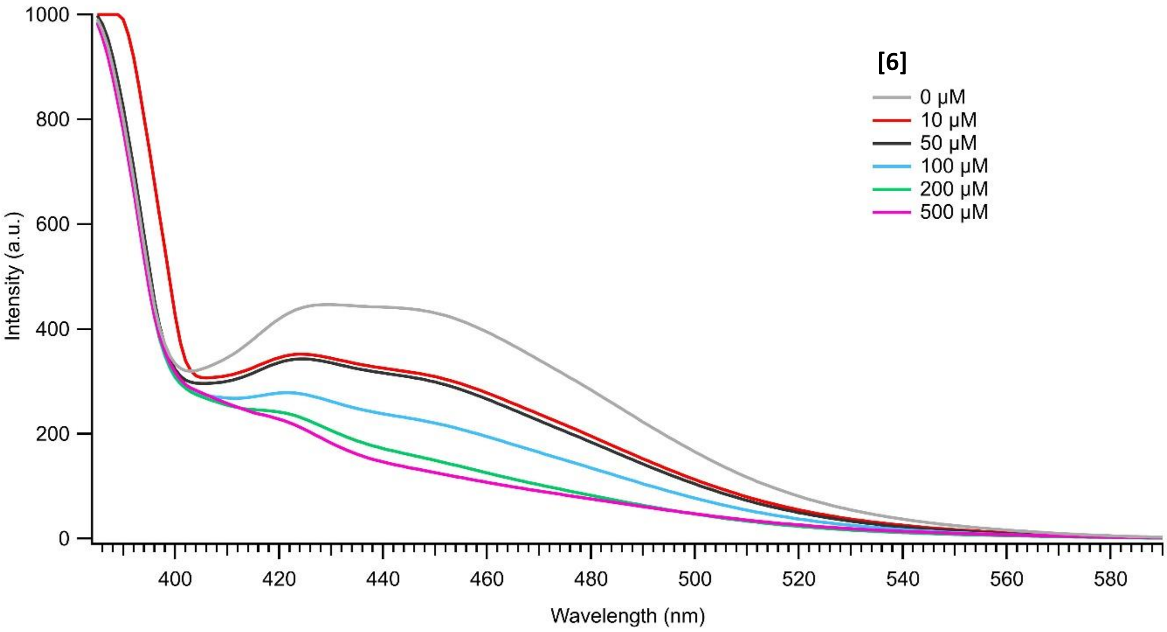

Supplementary Fig. 31 Emission spectra of **1a-K** (50 µM) in acetonitrile in the presence of an increasing amount in **6**, from 0 up to 500 µM upon light excitation at 380 nm at 298K.

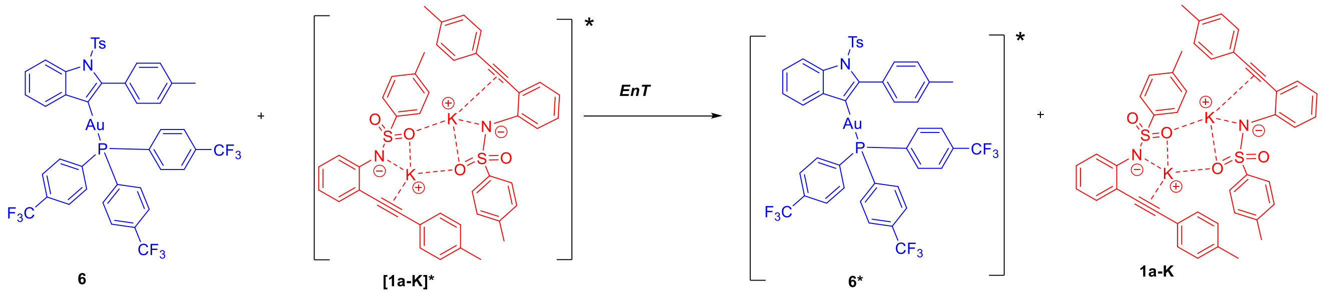

Stern-Volmer treatment

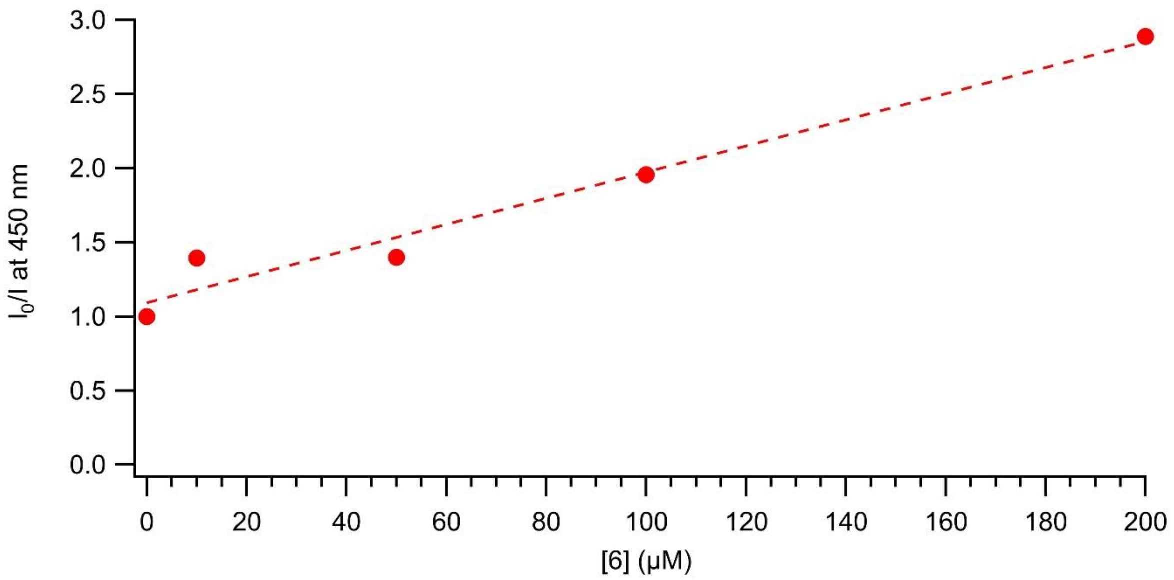

Supplementary Fig. 32 Stern-Volmer plot from the data **Supplementary Fig. 31** at the maximum of emission of **1a-K**.

I_0 designates the luminescence intensity of the solution at 50 µM in **1a-K** in the absence of **6** and I (450 nm) is the one of the solution in the presence of the quencher. The dependence of I/I_0 on **[6]** is linearly fitted with a good correlation constant (96.3%) and yields 9.4 10^3 $L.mol^{-1}$ for the slope. Using the following equation related to the bimolecular quenching of an excited moiety (1), we can extract the value for the product $k_Q\tau$ = 9.4 10^3 $L.mol^{-1}$:

$$\mathbf{6} + [\mathbf{1a-K}]^* \rightarrow [\mathbf{6}]^* + \mathbf{1a-K} \qquad k_Q$$

$$\frac{I(450\ nm)}{I_0} = 1 + k_Q\tau[\mathbf{6}] \qquad (1)$$

Luminescence decay of **1a-K**

Optical set-up: A homebuilt setup was used for performing lifetime measurements. It includes a low current LED as modulable light source at 405 nm. The luminescence lifetime measured at 25°C in the frequency domain by collecting the fluorescence signal filtered at 480 ± 30 nm (Chroma Technology, Bellows Falls, VT) with a f = 50 mm lens onto a multipixel photon counter (C13366-1350GA, Hamamatsu Photonics K. K., Hamamatsu, Japan) and analyzing the amplitude of the fluorescence modulation as a function of the angular frequency of light modulation as indicated in **Supplementary Fig. 33**[9].

The luminescence decay experiments have been performed on a **1a-K** solution of salt (10 mM) in acetonitrile. Under sinusoidal forcing of radial frequency ω, the normalized amplitude A of the fluorescence emission can be expressed as (2) where τ designates the luminescence lifetime[10]:

$$A = \sqrt{\frac{1}{1+\omega^2\tau^2}} \qquad (2)$$

Since we observed above 95% of modulation amplitude at 2 MHz modulation frequency, the luminescence lifetime of **1a-K** is less than 26 ns. Direct curve fitting of **Supplementary Fig. 33c** with Eq. 2 gives 20 ns as the upper limit of τ.

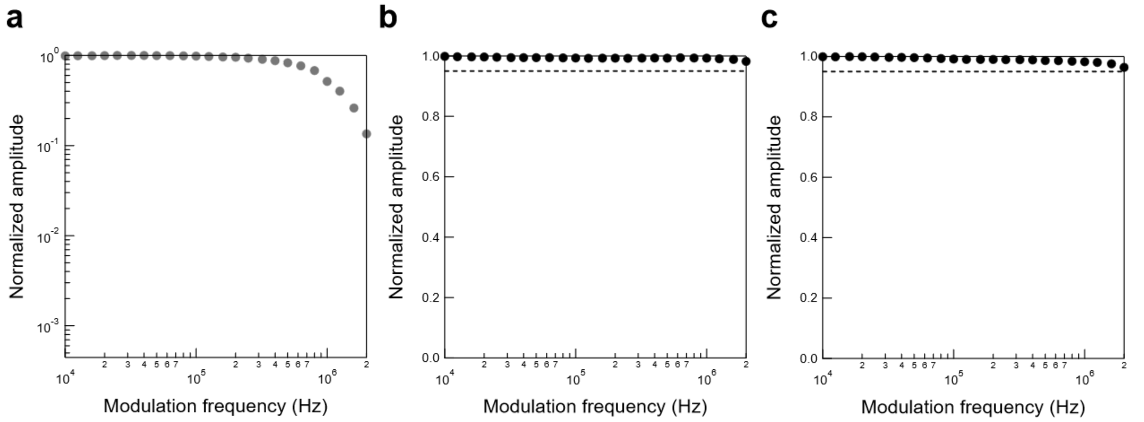

Supplementary Fig. 33 Estimation of the lifetime of **1a-K**. **a**: Instrument response in the 10kHz – 2MHz range of modulation frequency. **b**: Control experiment with fluorescein in PBS buffer. **c**: Normalized amplitude of the luminescence response of **1a-K**. Dashed line is 0.95 level.

Upon assuming the quenching to be dynamic, we can estimate a lower limit of the quenching constant from the estimated lifetime of the excited state of the salt (presumably a singlet state according to the value of τ) and the slope of the Stern-Volmer plot (**Supplementary Fig. 32**):

$$k_Q\tau \ = 9.4.10^3\ \mathrm{L.mol^{-1}}\ and\ \tau\ \leq\ 20\ \mathrm{ns} \qquad (4)$$

$$\rightarrow\ k_Q \geq 4.7.10^{11}\ \mathrm{L.mol^{-1}.s^{-1}}$$

This quenching constant is much higher than the estimate of the second order diffusion rate in the acetonitrile[11,12]:

$$k_{diff} = \frac{8RT}{3\eta_{acetonitrile}} = 1.8.10^{10}\text{L.mol}^{-1}.\text{s}^{-1} \quad (5)$$

$$\rightarrow\ k_Q \geq 26\ k_{diff}$$

Influence of the temperature

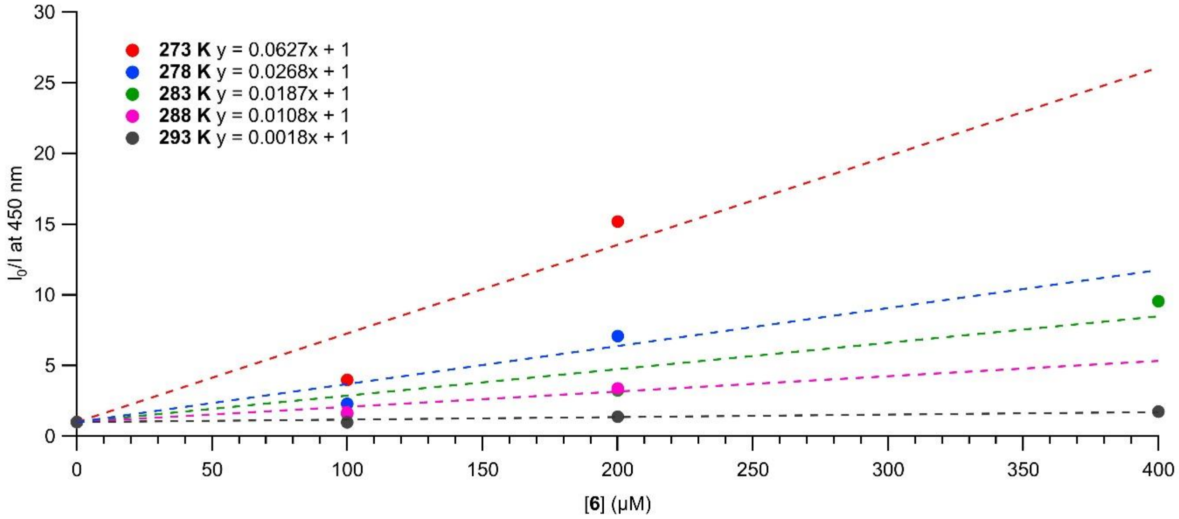

Supplementary Fig. 34 Stern-Volmer plots at different temperatures.

In this case, one is left to conclude that quenching is not dynamic but static. Then, the Stern-Volmer plots are still linear and their slopes are related to the thermodynamic association constant (**K**):

$$[\mathbf{1a} - \boldsymbol{K}] + \mathbf{6} \rightarrow [\mathbf{1a} - \boldsymbol{K} - \mathbf{6}] \qquad \boldsymbol{K}°(\boldsymbol{T}) \quad (1)$$

$$\frac{\boldsymbol{I_0}}{\boldsymbol{I}} = \mathbf{1} + \boldsymbol{K}°(\boldsymbol{T})[\mathbf{6}] \qquad (2)$$

$$\boldsymbol{\Delta_r G}^{\circ} = -RTln\left(\boldsymbol{K}^{\circ}(\boldsymbol{T})\right) = \boldsymbol{\Delta_r H}^{\circ} - T\boldsymbol{\Delta_r S}^{\circ} \qquad (3)$$

Below are the Van't Hoff plots extracted from the Stern-Volmer experiments:

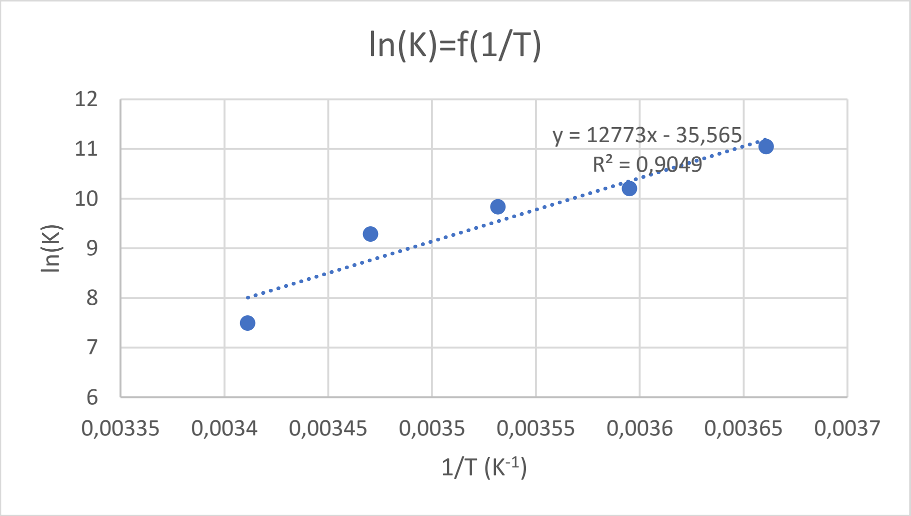

Supplementary Fig. 35 Van't-Hoff analysis (1).

The related enthalpy for the formation of [**1a-K-6**] $\Delta_r H^{\circ}$=-106.2 kJ.mol^{-1}.

To determine the reaction entropy, we used the following equation:

$$\Delta_r G^{\circ} = -RTln(K^{\circ}) = \Delta_r H^{\circ} - T\Delta_r S^{\circ}$$

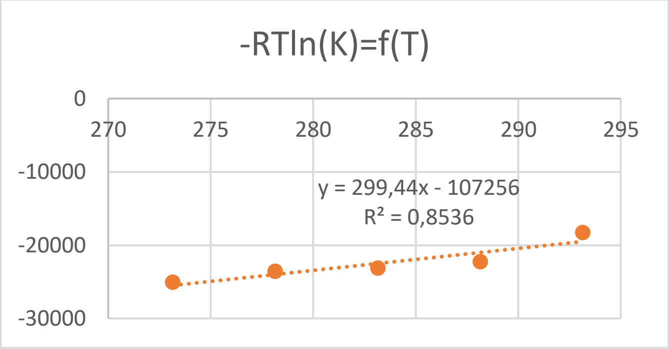

Supplementary Fig. 36 Van't-Hoff analysis (2).

The reaction entropy $\Delta_r S^{\circ}$= -299.44 J.mol^{-1}.K^{-1} is negative.

Influence of **2a** on the luminescence quenching

The quenching of the luminescence of a stoechiometric mixture of **1a-K** and **2a** in the presence of **6** has been probed in acetonitrile. Different solutions of **1a-K:2a** (50 µM for both of them) with an increasing amount in **6** were prepared and their emission spectra were recorded. 380 nm was chosen as excitation wavelength to selectively excite the aggregate **1a-K** and not **6**.

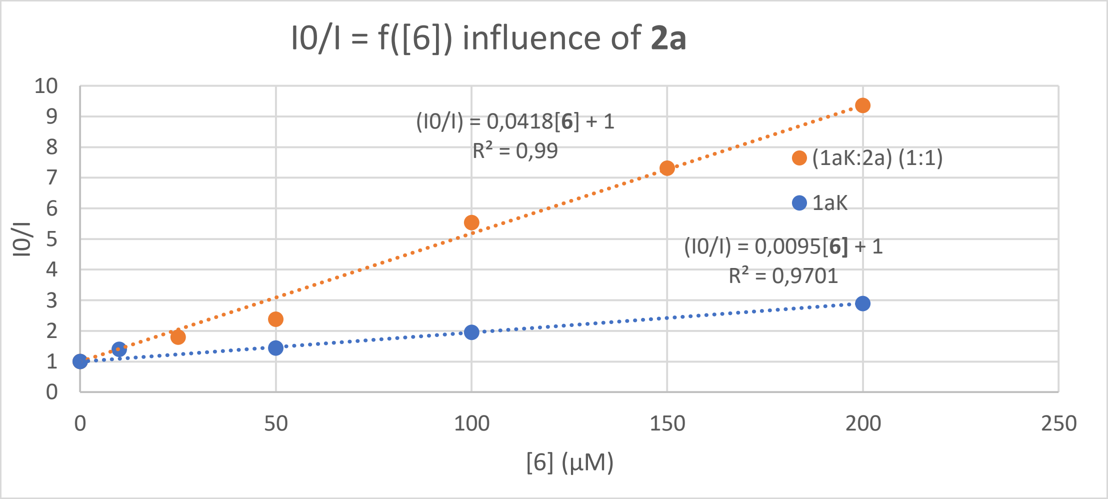

Supplementary Fig. 37 Stern Volmer plots for a stoechiometric mixture 1a-K:2a and 1a-K quenched by 6.

Assuming that the nature of the quenching does not change in the presence of **2a**, the Stern-Volmer plot here above points out a significant increase in the association thermodynamic constant of **1a-K** with **6**, ($K_{\mathbf{1a\text{-}K:2a}}$/$K_{1a\text{-}K}$ =4.4 at 298K). This last result suggests a synergetic effect in the mechanism. The halogen bond between **1a-K** and **2a** would foster the association and the energy transfer between the amide salt and **6** and gather the excited metallic center to the iodoalkyne to perform the oxidative addition.

f. Modelling part

Computational details

All Density Functional Theory (DFT) calculations were performed using the Gaussian 09 software package.[13] Geometries optimizations and frequency calculations were done with the PBE0 functional and the split valence Ahlrichs-type basis set def2-SVP[14] for all atoms. Vibrational analysis served to locate minima (no imaginary frequency) or transition structures (one imaginary frequency). Single point energy calculations were obtained using the PBE0 functional and the triple-ζ Ahlrichs-type basis set def2-TZVP[14] with the Polarizable Continuum Model (PCM) model to mimic the ACN solvent[15] Definitions of the basis sets were obtained from the *Basis Set Exchange* library[16]. The X-bonding interaction between the iodo-alkyne and the sulfonamide anion was considered using an energy decomposition analysis (EDA) following a Morokuma-like energy partition of the bond[17], using the Amsterdam Density Functional (ADF)[18] program at the BP86-D3BJ/TZ2P(ZORA) level of theory[19]. Combining the EDA approach with the natural orbitals for chemical valence (NOCV) theory[20] enables to further decompose qualitatively and quantitatively the orbital term into the different deformation density contributions of the bonding. NOCVs with the same absolute energy eigenvalues can be grouped together to describe charge-transfer channels between the molecular fragments. The visualization of these pairwise NOCVs allows their assignment as donating and backdonating

processes. Computed structures shown in this work have been depicted using the Chemcraft software[21].

Association complex between an iodoalkyne and a sulfonamide

The presence of an iodine atom and a negatively charged nitrogen atom on the complex led us to consider the formation a non-covalent halogen bonding (XB)[22] entity between the iodoalkyne and the sulfonamide anion. This hypothesis was confirmed by the computed molecular electrostatic potential (MEP)[23] of the iodo-alkyne which is displayed in **Fig. 38**. A clear positive region is observed on the outermost region of the halogen atom indicating there the presence of a σ-hole. Such feature is known to be responsible for the non-covalent interaction between a halogen atom and a Lewis base, forming a X-bond.

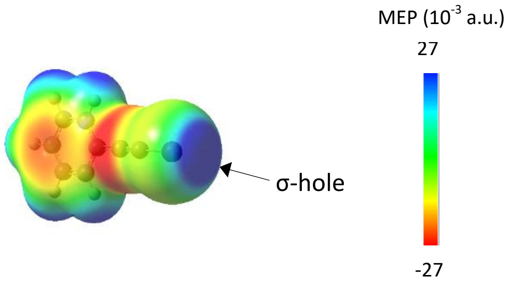

Supplementary Fig. 38 Molecular electrostatic potential on the 0.001 a.u. electron density isosurface of iodo-alkyne.

Looking more closely to the interaction between the alkyne iodine and the amide anion shows that a X-bond is indeed expected between the iodide atom and the negatively charged nitrogen, with the characteristic geometric parameters defined for such non-covalent bond (**Supplementary Fig. 39**) : a I-N distance of 2.78 Å, smaller than the sum of the two van der Waals radii of I and N, combined with an interaction angle close to 180°.[22] The association between the two compounds is determined to be exothermic by 5.0 kcal/mol, in agreement with the usual exothermicities observed[22].

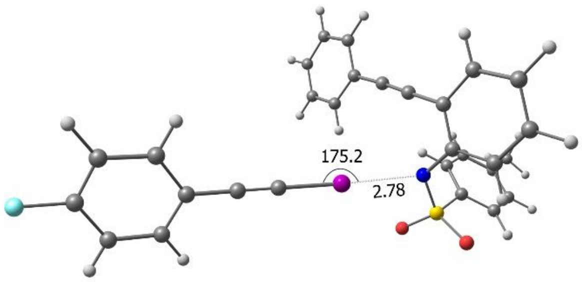

Supplementary Fig. 39 Optimized geometry of the **2b**-**1a** non-covalent complex (bond length in Å, angle in degree).

The electronic depiction of such X-bond was investigated using the approach combined with a ETS-NOCV scheme. In **Fig. 40**, the contour plots of the major NOCV contribution is presented for the alkyne iodide and the alkyne iodide-NTos complex. On the alkyne iodide, the σ-hole is visible on the outermost surface of the iodine atom. By adding the NTos moiety, the EDA results show that the main attractive contribution to the I-N bond is electrostatic. In **Fig. 40**, the main deformation density (more than 94 % of the total orbital interaction) and the only one located in the halogen area is depicted. A positive electron density is observed between the I and N atoms (pink area), indicating a chemical bond.

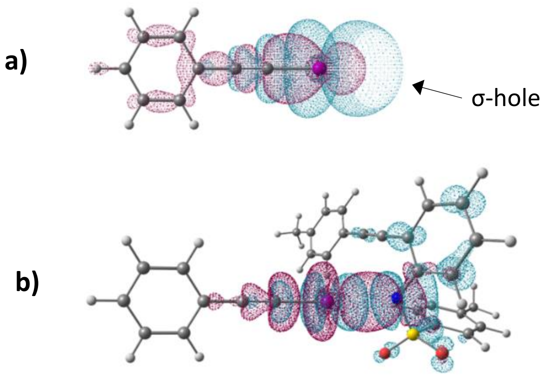

Supplementary Fig. 40 Contours of deformation density Δρ connected with the pairs of interacting orbitals in the a) C_6H_5CC-I and b) alkyne iodide-NTos complex. Blue surfaces represent a loss of electron density and pink surfaces, a gain of electron density. Isosurface value: 0.0025 a.u.

These theoretical investigations on the halogen bond between the alkyne iodide and the NTos were also performed on the alkyne iodide interacting with the o-alkynyl-phenate. In this case, no X-bonding could be established which thus shows a fundamental difference between the two systems. The stabilizing bond created between the alkyne iodide and NTos in **1a-K** enables to keep together all the partners of the oxidative addiction reaction and may act as a reactivity enhancer.

<u>Mechanism of the oxidative addition</u>

The previous results led us to consider the formation of an excited (and presumably singlet) electronic state of **6**. By analogy with our former study, an inter-system crossing of the excited intermediate may be assumed to generate a triplet complex (^{3}**6**) able to carry out the oxidative addition of the alkyne iodine. Following this guiding principle, a theoretical study of the interaction of ^{3}**6** facing to the iodo-alkyne was undertaken in order to determine the reaction pathway leading to the **3ab** product.

In the same vein than for ^{3}**6-*O***, we could establish that the approach of the alkyne iodine (in its ground state) to ^{3}**6** leads to a C_{sp}-I bending which strongly reminds the C_{sp}-I bending observed on triplet ^{3}**2b** (**Supplementary Fig. 41**). This feature suggests an energy transfer of ^{3}**6** to **2b** which was previously shown to occur at a relatively long distance (3.6 Å) for the ^{3}**6-*O*** analogue (but not detailed here). This bending gives rise to the stabilized ^{3}**II** complex (10.4 kcal.mol^{-1} below ^{3}**6** + **2b**) which is then able to lead to the C_{sp}^{alkyne}-C_{sp}^{indole} bond formation (intermediate ^{3}**III**) *via* the key transition structure **TS1** located 11.2 kcal.mol^{-1} above ^{3}**II**. The ^{3}**III** complex is then subjected to a stabilizing rearrangement in which the Au atom moves away from the C_{sp}^{indole} (d(Au-C_{sp}^{indol}) increases from 2.22 up to 3.00 Å) gets closer and bonds to the C_{sp}^{alkyne} (Complex ^{3}**IV**, -50.6 kcal.mol^{-1}, d(Au-C_{sp}^{alkyne}) = 2.08 Å vs 2.89 Å in ^{3}**III**). Finally, the system evolves into the ^{3}**V** complex by increasing the distance between

Au and C_{sp}^{alkyne} (up to 5.21 Å). From ^{3}**V**, one can assume either a deexcitation step leading to the **3ab** product through the corresponding singlet complex of ^{3}**V** which appears to be dissociative or the dissociation of the ^{3}**V** intermediate giving rise to the **3ab** product and ^{3}(p-$CF_3Ph)_3$PAuI which then deexcites to form the products located -104.9 kcal.mol^{-1} below the ^{3}**6** + **2b** reactants.

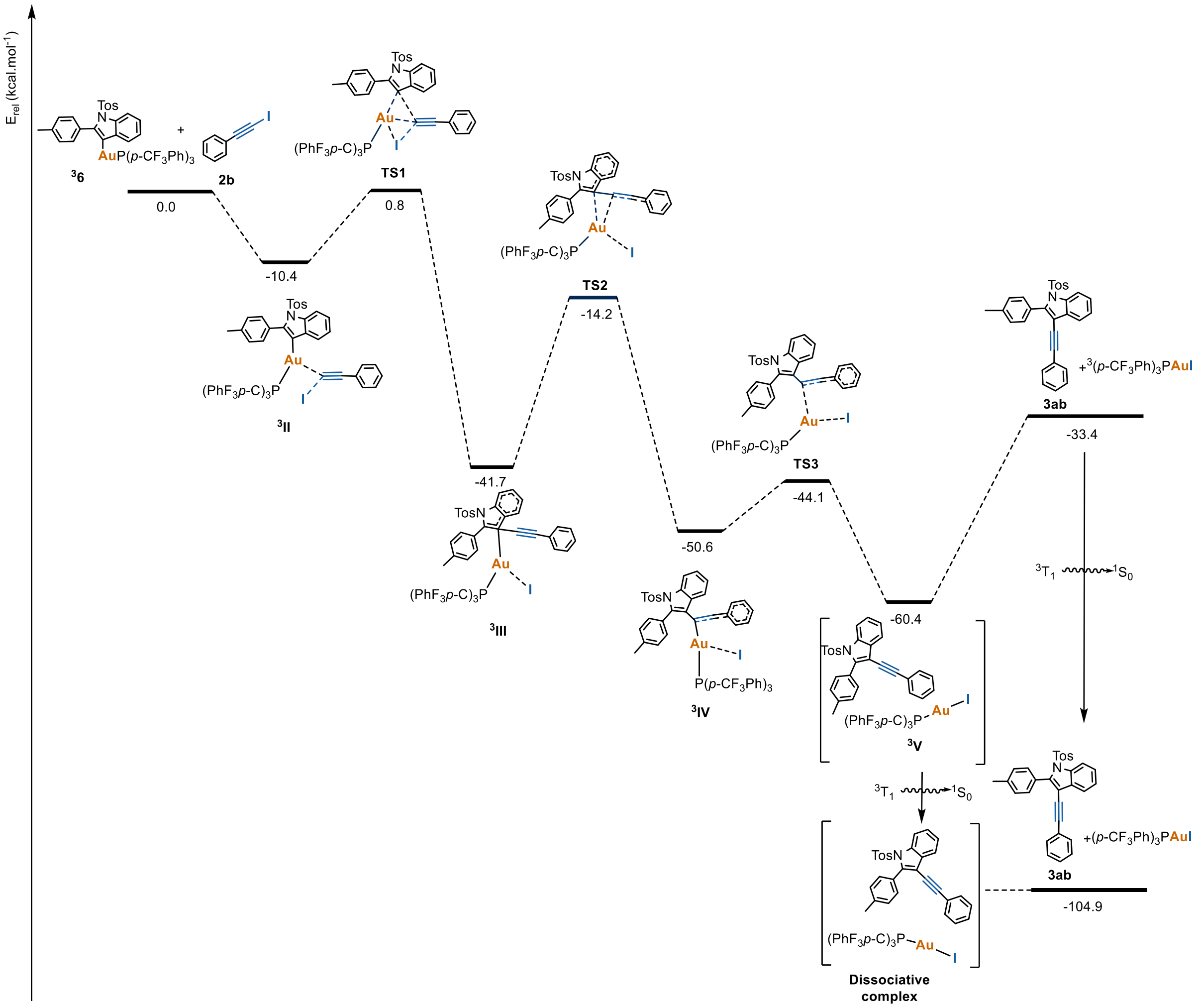

Supplementary Fig. 41 Potential surface energy of the reaction of ^{3}**6** with **2b** leading to the **3ab** product. Energies are given relative to the starting products and are in kcal.mol^{-1}.

Structural and energetics data

Supplementary Table 2 Calculated electronic (Eel) and zero-point (ZPE) energies (in hartrees, H) of optimized structures of the compounds studied in this work

	**Eel**	**ZPE**
**^{3}6**	-3633.551643	0.6354944
**2a**	-605.2497529	0.1011879
**^{3}II**	-4238.821739	0.7404879
**TS1**	-4238.804732	0.7386291
**^{3}III**	-4238.869605	0.7384761
**TS2**	-4238.869219	0.7379814
**^{3}IV**	-4238.886497	0.7412485
**TS3**	-4238.876926	0.739545
**^{3}V**	-4238.902175	0.7412485
**3ab**	-1759.16167	0.4499927
**^{3}(*p*-CF_3Ph)$_3$PAuI**	-2479.812596	0.2924413
**(*p*-CF_3Ph)$_3$PAuI**	-2479.69807	0.2917695

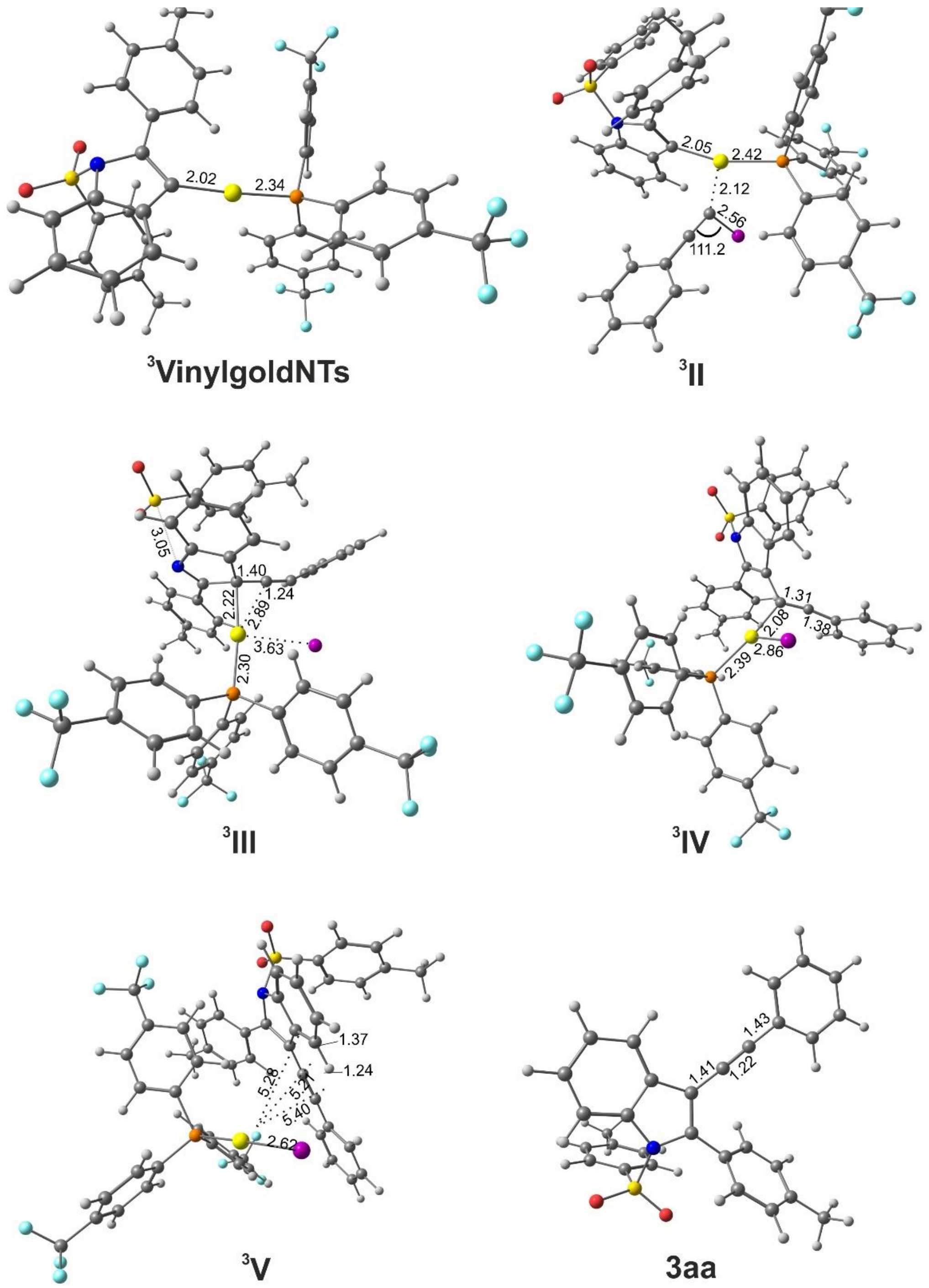

Supplementary Fig. 42 Structures of intermediates implied in this work. Selected bond lengths are in Å, angles in degrees.

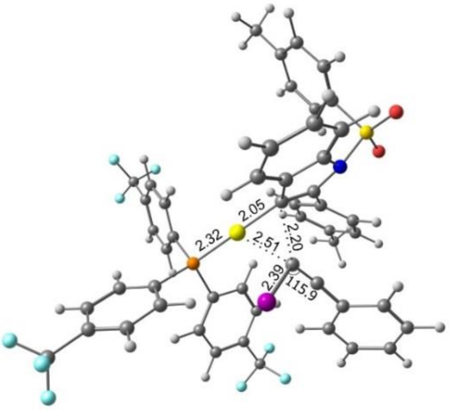

TS1

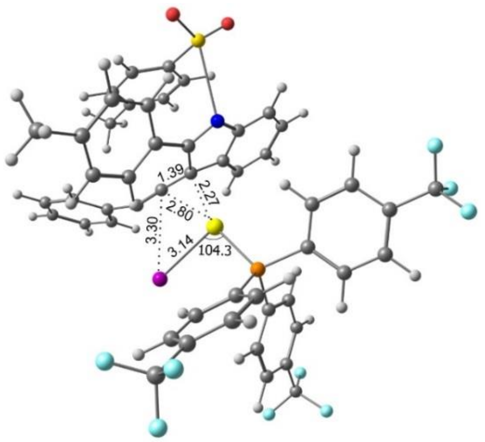

TS2

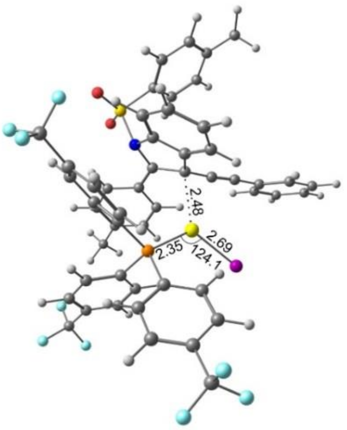

TS3

Supplementary Fig. 43 Structures of transition structures implied in this work. Selected bond lengths are in Å, angles in degrees.

Cartesian coordinates

Supplementary Table 3 Cartesian coordinates of the optimized structures of the compounds involved in this work

g. NMR experiments on 1a-K

NMR spectra were recorded on Bruker NEO ^{1}H 500 MHz equipped with a TXI probe using standard pulse sequences. Samples in acetonitrile-d_3 were prepared in 5 mm NMR glass tubes. Spectra were processed with Bruker Topspin 4.0.2.

<u>^{1}H NMR spectra of **1a-K** and **1a**</u>

^{1}H NMR spectra of eight samples of **1a-K** were recorded from 10 (**Supplementary Fig. 44**a) to 0.05 (**Supplementary Fig. 44**h) mM.

Upon decreasing the concentration, the chemical shifts of the two tolyl groups (H2, H3, H8, H9) do not vary significantly. In contrast, the aromatic proton signals of the di-*ortho* substituted aromatic ring (H4, H5, H6, H7) broaden and shift. At 0.1 mM (**Supplementary Fig. 44**g), the broadening is so high than they are no longer observed. Finally, they are re-observed at 0.05 mM (**Supplementary Fig. 44**h). Comparison of this last spectrum (**Supplementary Fig. 44**h) with that of pure **1a** (**Supplementary Fig. 44**i) underlines that the chemical shifts in **1a-K** at 0.05 mM are almost the same than those in pure **1a**.

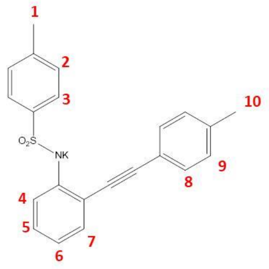

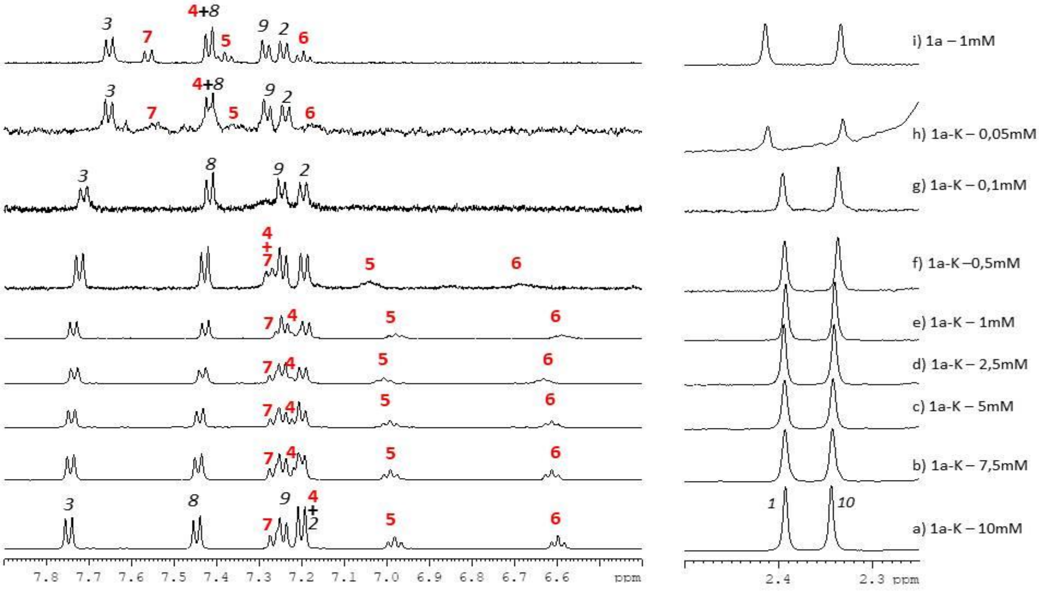

Supplementary Fig. 44 ^{1}H NMR spectra of **1a-K** in ACN-d_3 at various concentrations (from a) to h) and ^{1}H NMR spectrum of **1a** at 1 mM (i).

^{1}H NMR NOE-1D spectra of **1a-K**

1D NOESY using selective refocussing with a shaped pulse (selnogpzs.2 (avance-version (17/01/23)) was performed on **1a-K** at 0.05, 0.5 and 10 mM by saturating H8. Dipolar effects were observed only at 0.5 and 10 mM with protons H2 and H3 (see red arrows)

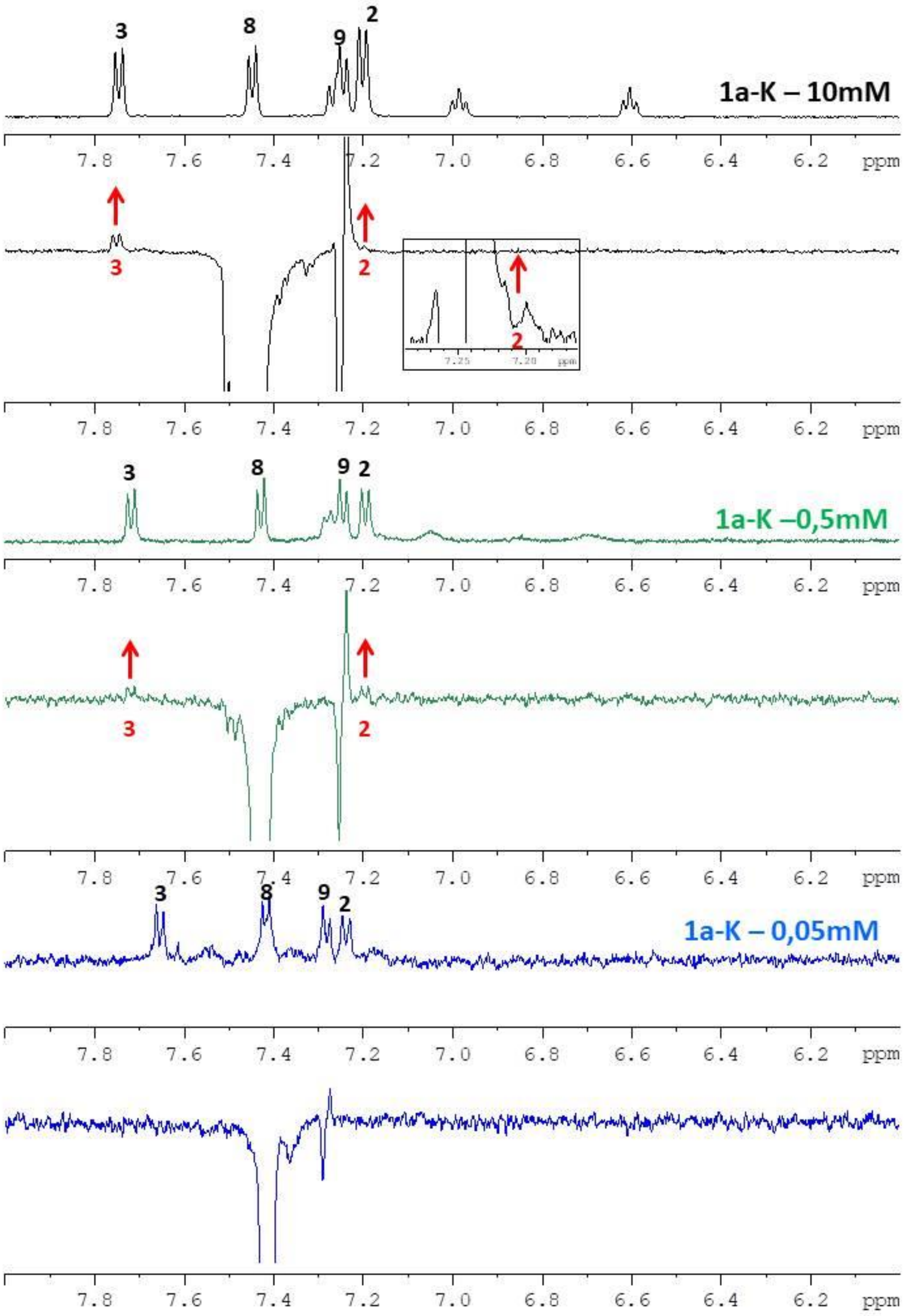

Supplementary Fig. 45 ^{1}H and Noe-1D spectra of **1a-K** in ACN-d_3 at 10, 0.5, and 0.05 mM concentrations

Diffusion Studies

The self-diffusion coefficient of the **1a-K** (10, 5, 1, 0.5, 0.1 and 0.05 mM) solutions in ACN-d_3 at 295 K were determined by the PGSE-NMR technique, by monitoring the ^{1}H signal on a Bruker Avance 500 spectrometer equipped with a field gradient probe unit. This method was first introduced by Stejskal and Tanner.[24] Here, the ledbpgp2s 2D sequence combining constant time, bipolar pulse, stimulated echo, and the longitudinal eddy current delay method, was used.[25] All the NMR signals give rise to monoexponential decays following $A = A_0 \exp[-\gamma_H^2\ \delta\ ^2G^2(\ \Delta - \delta\ /3)\ D]$ (Eq. 1) where A is the echo amplitude in the presence of the gradient pulse, A_0 is the echo amplitude in the absence of the gradient pulse, γH is the proton gyromagnetic ratio, δ is the gradient pulse length, G is the strength of the applied field gradient, Δ is the interval between two field gradient pulses, and D is the diffusion coefficient. By varying the field gradient amplitude G, a series of experiments was collected, and the diffusion coefficient was extracted using a simple fit to eq 1 for the aromatic peaks and the aliphatic methyls. Fitted curves are reported in **Supplementary Fig. 46**.

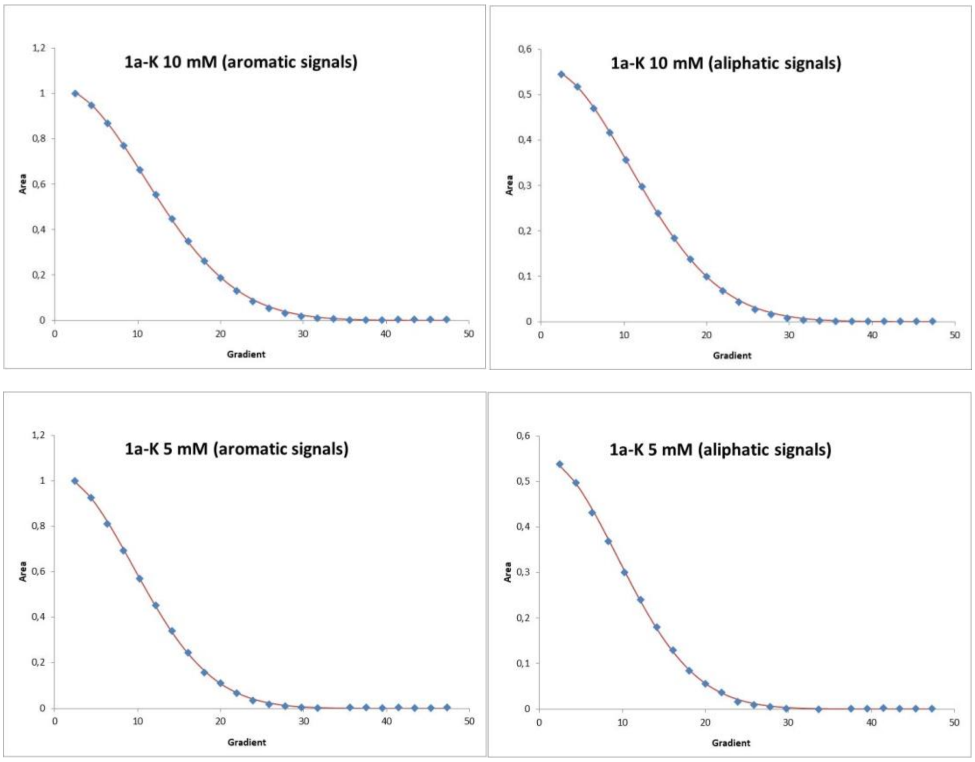

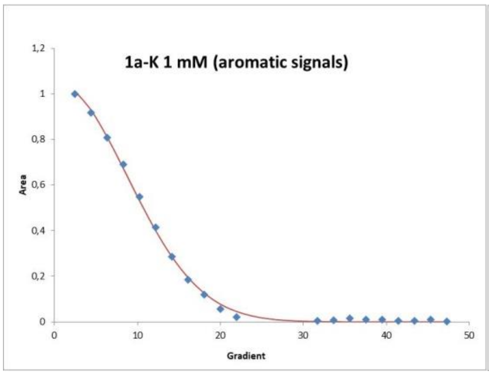
1a-K 1 mM (aromatic signals)
Area
Gradient

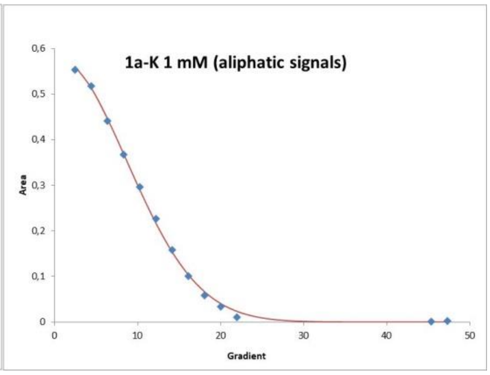
1a-K 1 mM (aliphatic signals)
Area
Gradient

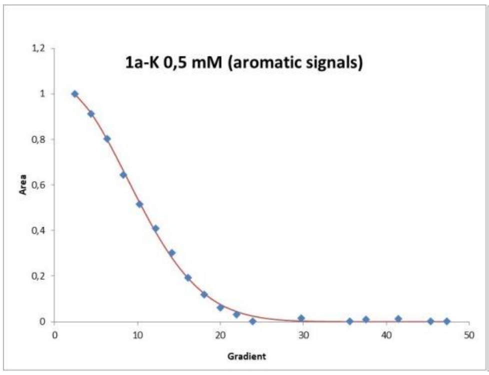
1a-K 0,5 mM (aromatic signals)
Area
Gradient

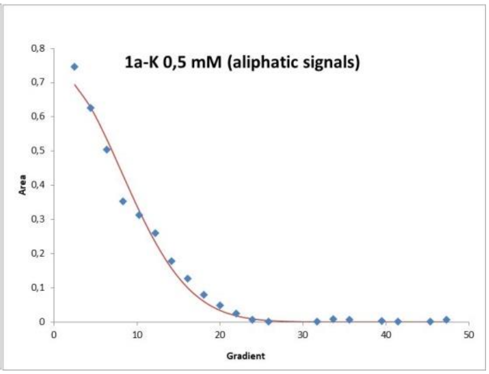
1a-K 0,5 mM (aliphatic signals)
Area
Gradient

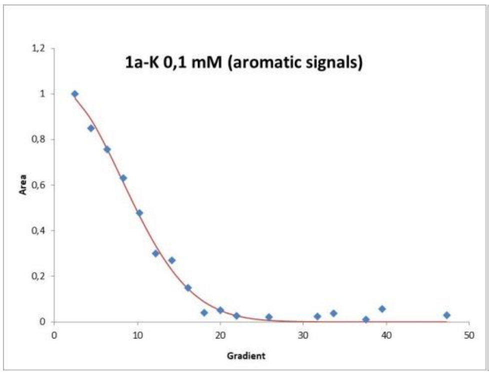
1a-K 0,1 mM (aromatic signals)
Area
Gradient

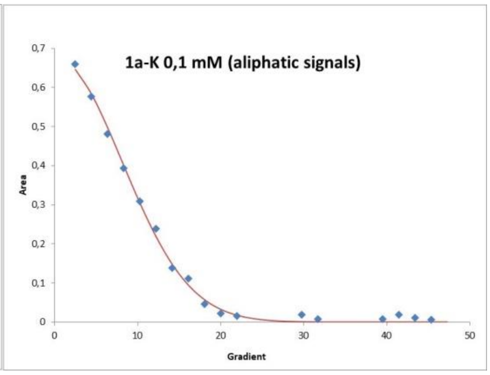
1a-K 0,1 mM (aliphatic signals)
Area
Gradient

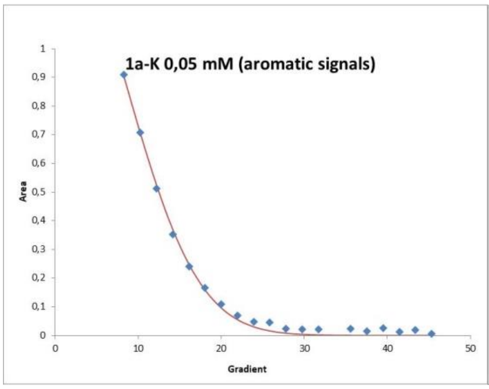
1a-K 0,05 mM (aromatic signals)
Area
Gradient

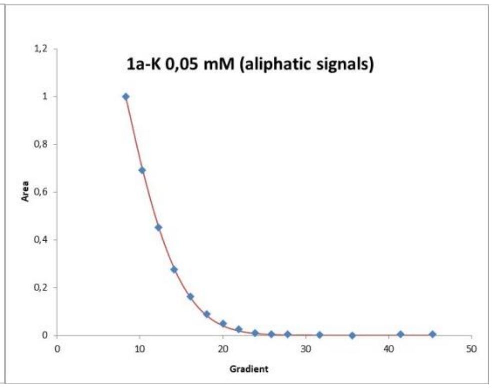
1a-K 0,05 mM (aliphatic signals)
Area
Gradient

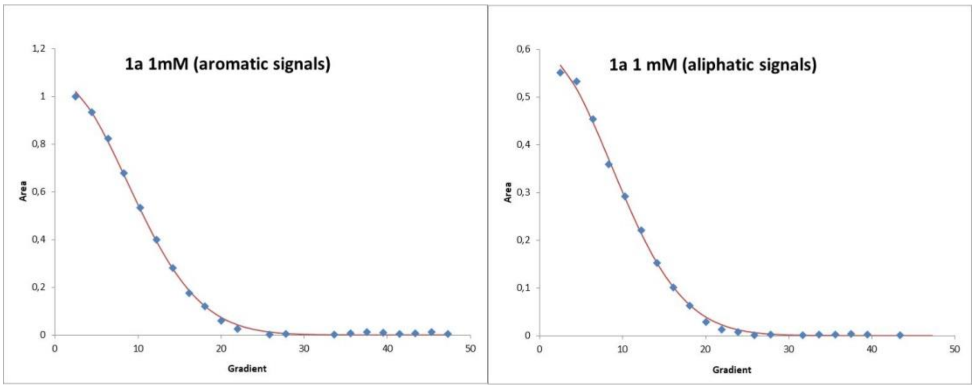

Supplementary Fig. 46 Fitted curves of **1a-K** in ACN-d_3 at 295 K at various concentrations and **1a** at 1 mM.

The set of average values are gathered in **Supplementary Table 4**. Assuming the molecular assemblies to be spherical, their hydrodynamic radii r_H can be retrieved from the translational diffusion coefficients with the Stokes – Einstein law [26] : D = $\frac{kT}{6\pi\eta R_H}$, where k_B is the Boltzmann's constant, T is the absolute temperature, η is the solvent viscosity, and D is the translational diffusion coefficient. Moreover, in the spherical approximation, the ratio of the translational diffusion coefficients for two different molecular species (Di/Dj) is inversely proportional to the cubic-root of the ratio of their molecular weight (MW).[27] Hence the evolution of the molecular weight of the molecular assembly on the concentration could be estimated taking the most diluted sample (0.05 mM) as a reference for the values of D and MW.

Concentration/mM	D / nm².s^{-1}	R_H / Å	MW g/mol
**1a-K (10)**	2.52 ± 0.03	2.30 ± 0.03	2840 (calc)
**1a-K (5)**	3.34 ± 0.03	1.74 ± 0.02	1220 (calc)
**1a-K (1**	3.89 ± 0.05	1.49 ± 0.02	780 (calc)
**1a-K 0,5**	4.18 ± 0.33	1.39 ± 0.11	620 (calc)
**1a-K 0,1**	4.47 ± 0.01	1.30 ± 0.01	510 (calc)
**1a-K 0,05**	4.85 ± 0.87	1.19 ± 0.21	399,5
**1a (1mM)**	3.97 ± 0.04	1.46 ± 0.01	361,5

Supplementary Table 4. Variation of the translational diffusion coefficient of **1a-K** as a function of its concentration and of **1a** at 1 mM in ACN-d_3 at 295 K and estimated hydrodynamic radius and molecular weight.

2. Experimental details and analytical data

a. Synthesis of starting materials 1

General Procedure 1 **(GP1):** Synthesis of **1a** – **1m** and **1Ms-a**.

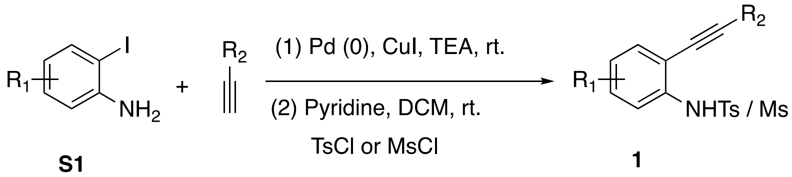

S1 **1**

To a solution of the corresponding 2-iodo aniline **S1** (4.0 mmol, 1.0 equiv.) in dry Et_3N (5 mL), the corresponding terminal alkyne (6.0 mmol, 1.5 equiv.) and $PdCl_2(PPh_3)_2$ (0.04 mmol, 10 mol%) were added. After 5 min stirring at room temperature, CuI (0.08 mmol, 20 mol%) was added and the reaction mixture stirred at same temperature for completion (4 to 12 h). After completion of the, the reaction mixture was poured into ice-cold water and extracted into EtOAc (3 x 10 mL). The combined organic layers were washed with brine solution, dried and evaporated under reduced pressure. The crude residue was purified by flash chromatography on silica gel to give the products in good to excellent yield.

Then the obtained compound (1 mmol) was dissolved in dichloromethane (20 mL) .Pyridine (3 equiv.) followed by tosyl chloride or methanesulfonyl chloride (1.3 equiv.) were added at 0 ^{o}C over a period of 15 min. After the addition was complete, reaction was stirred at RT for 12 hours. After completion of the reaction (monitored by TLC), reaction mass quenched with ice-cold water and extracted into dichloromethane. The combined organic layer was washed with 2N HCl and brine solution and dried and evaporated to give a crude residue. The obtained crude material was passed through flash column chromatography to give pure product in good yield (**1**).

4-Methyl-*N*-(2-(*p*-tolylethynyl)phenyl)benzenesulfonamide (**1a**)

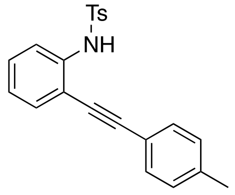

Prepared according to the general procedure **GP1** on a 1.0 mmol scale as a yellow solid (347 mg, 96 % yield). The spectroscopic data is in agreement with that previously reported.[28] ^{1}H NMR (400 MHz, $CDCl_3$) δ 7.65 (d, *J* = 8.3 Hz, 2H), 7.62 (s, 1H), 7.37 - 7.32 (m, 3H), 7.28 - 7.24 (m, 1H), 7.22 - 7.19 (m, 1H), 7.16 (dd, *J* = 12.3, 7.9 Hz, 4H), 7.03 (td, *J* = 7.6, 1.2 Hz, 1H), 2.38 (s, 3H), 2.31 (s, 3H).

N-(2-((4-Fluorophenyl)ethynyl)phenyl)-4-methylbenzenesulfonamide (**1b**)

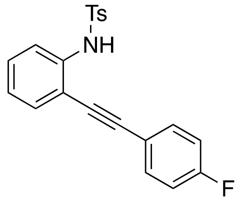

Prepared according to the general procedure **GP1** on a 1.0 mmol scale as a yellow solid (328 mg, 90 % yield). The spectroscopic data is in agreement with that previously reported.[29] ^{1}H NMR (300 MHz, $CDCl_3$) δ 7.67 (d, *J* = 8.3 Hz, 2H), 7.61 (d, *J* = 8.2 Hz, 1H), 7.50 - 7.41 (m, 2H), 7.40 - 7.27 (m, 2H), 7.18 (d, *J* = 8.0 Hz, 3H), 7.13 - 7.03 (m, 3H), 2.35 (s, 3H). ^{19}F NMR (376 MHz, $CDCl_3$) δ –109.38.

N-(2-((2-Fluorophenyl)ethynyl)phenyl)-4-methylbenzenesulfonamide (**1c**)

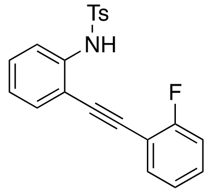

Prepared according to the general procedure **GP1** on a 1.0 mmol scale as a yellow solid (328 mg, 90 % yield). The spectroscopic data is in agreement with that previously reported.[30] ^{1}H NMR (400 MHz, $CDCl_3$) δ 7.71 (t, *J* = 9.0 Hz, 3H), 7.50 - 7.41 (m, 2H), 7.40 - 7.34 (m, 2H), 7.34 - 7.27 (m, 1H), 7.16 (dd, *J* = 11.6, 7.8 Hz, 4H), 7.05 (t, *J* = 7.6 Hz, 1H), 2.29 (s, 3H).

N-(2-((4-Methoxyphenyl)ethynyl)phenyl)-4-methylbenzenesulfonamide (**1d**)

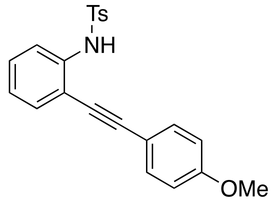

Prepared according to the general procedure **GP1** on a 1.0 mmol scale as a grey solid (358 mg, 95 % yield). The spectroscopic data match those previously reported in the literature.[30] ^{1}H NMR (400 MHz, $CDCl_3$) δ 7.67 (d, *J* = 8.3 Hz, 2H), 7.61 (d, *J* = 8.1 Hz, 1H), 7.41 (d, *J* = 8.8 Hz, 2H), 7.35 (d, *J* = 9.0 Hz, 1H), 7.28 (d, *J* = 8.9 Hz, 1H), 7.23 - 7.15 (m, 3H), 7.05 (t, *J* = 7.6 Hz, 1H), 6.91 (d, *J* = 8.8 Hz, 2H), 3.86 (s, 3H), 2.34 (s, 3H).

N-(2-((3-Methoxyphenyl)ethynyl)phenyl)-4-methylbenzenesulfonamide (**1e**)

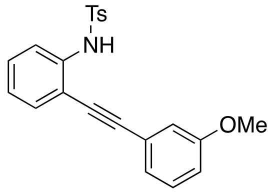

Prepared according to the general procedure **GP1** on a 1.0 mmol scale as a grey solid (359 mg, 96 % yield). The spectroscopic data match those previously reported in the literature.[31] ^{1}H NMR (400 MHz, $CDCl_3$) δ 7.68 (d, *J* = 8.2 Hz, 2H), 7.63 (d, *J* = 8.2 Hz, 1H), 7.37 (d, *J* = 7.1 Hz, 1H), 7.28 (t, *J* = 7.9 Hz, 3H), 7.14 (s, 2H), 7.06 (dd, *J* = 10.1, 7.7 Hz, 2H), 7.00 (s, 1H), 6.94 (d, *J* = 8.3 Hz, 1H), 3.82 (s, 3H), 2.31 (s, 3H).

4-Methyl-*N*-(2-(phenylethynyl)phenyl)benzenesulfonamide (**1f**)

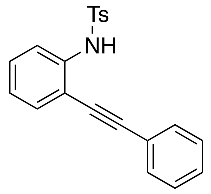

Prepared according to the general procedure **GP1** on a 1.0 mmol scale as a yellow solid (312 mg, 90 % yield). The spectroscopic data match those previously reported in the literature.[29] ^{1}H NMR (300 MHz, $CDCl_3$) δ 7.70 (d, *J* = 8.2 Hz, 2H), 7.65 (d, *J* = 8.2 Hz, 1H), 7.50 (dd, *J* = 6.7, 3.0 Hz, 2H), 7.45 - 7.37 (m, 4H), 7.32 (s, 1H), 7.26 - 7.15 (m, 3H), 7.09 (t, *J* = 7.6 Hz, 1H), 2.37 (s, 3H).

4-Methyl-*N*-(2-(*m*-tolylethynyl)phenyl)benzenesulfonamide (**1g**)

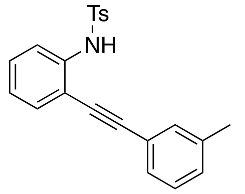

Prepared according to the general procedure **GP1** on a 1.0 mmol scale as a yellow solid (343 mg, 95 % yield). The spectroscopic data match those previously reported in the literature. [29] ^{1}H NMR (300 MHz, $CDCl_3$) δ 7.72 (d, *J* = 8.3 Hz, 2H), 7.66 (d, *J* = 8.0 Hz, 1H), 7.39 (d, *J* = 7.7 Hz, 1H), 7.29 (d, *J* = 3.3 Hz, 5H), 7.21 (dd, *J* = 13.7, 5.9 Hz, 3H), 7.12 - 7.04 (m, 1H), 2.41 (s, 3H), 2.35 (s, 3H).

N-(2-((4-Chlorophenyl)ethynyl)phenyl)-4-methylbenzenesulfonamide (**1h**)

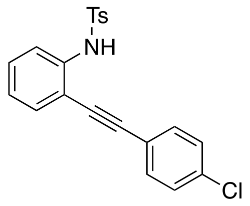

Prepared according to the general procedure **GP1** on a 1.0 mmol scale as a yellow solid (335 mg, 88 % yield). The spectroscopic data match those previously reported in the literature.[29] ^{1}H NMR (300 MHz, $CDCl_3$) δ 7.66 (d, *J* = 8.2 Hz, 2H), 7.61 (d, *J* = 8.3 Hz, 1H), 7.37 (d, *J* = 7.6 Hz, 5H), 7.29 (d, *J* = 8.3 Hz, 1H), 7.21 - 7.13 (m, 3H), 7.07 (t, *J* = 7.6 Hz, 1H), 2.35 (s, 3H).

4-Methyl-*N*-(2-((4-(trifluoromethyl)phenyl)ethynyl)phenyl)benzenesulfonamide (**1i**)

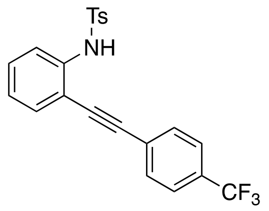

Prepared according to the general procedure **GP1** on a 1.0 mmol scale as a gray solid (369 mg, 89 % yield). The spectroscopic data match those previously reported in the literature.[29] ^{1}H NMR (300 MHz, $CDCl_3$) δ 7.71 (d, *J* = 8.3 Hz, 2H), 7.65 (d, *J* = 7.5 Hz, 3H), 7.59 (d, *J* = 8.4 Hz, 2H), 7.45 - 7.39 (m, 1H), 7.39 - 7.31 (m, 1H), 7.29 (s, 1H), 7.19 (d, *J* = 8.2 Hz, 2H), 7.11 (t, *J* = 7.6 Hz, 1H), 2.36 (s, 3H). ^{19}F NMR (376 MHz, $CDCl_3$) δ -62.89.

Synthesis of 1j

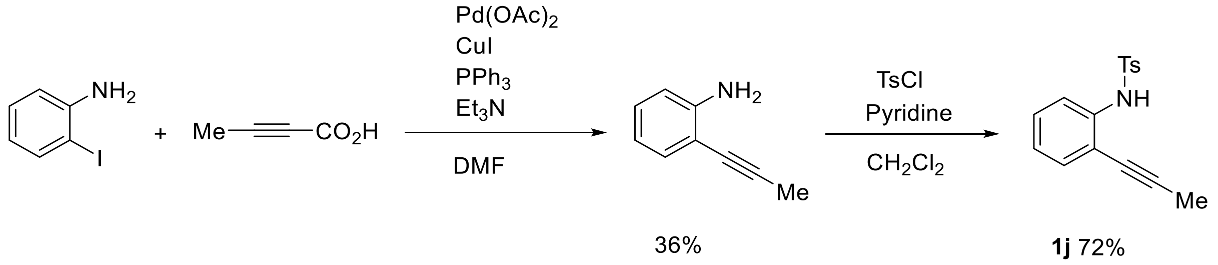

4-Methyl-N-(2-(prop-1-yn-1-yl)phenyl)benzenesulfonamide (**1j**)

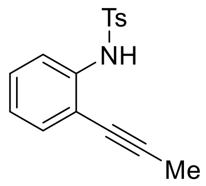

Prepared according to the procedure described in literature for the first step[45] and following general procedure 1 (**GP1**) for the second step to afford **1j** as a white solid (338 mg, 72%). The spectroscopic data is in agreement with that previously reported.[46] ^{1}H NMR (400 MHz, $CDCl_3$) δ 7.67 (d, *J* = 8.36 Hz, 2H), 7.53 (d, *J* = 7.90 Hz, 1H), 7.24 – 7.17 (m, 5H), 6.97 (t, *J* = 7.90 Hz, 1H), 2.37 (s, 3H), 2.06 (s, 3H).

N-(2-(Hept-1-yn-1-yl)phenyl)-4-methylbenzenesulfonamide (**1k**)

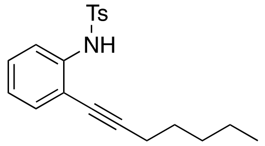

Prepared according to the general procedure **GP1** on a 1.0 mmol scale as a yellow solid (279 mg, 82 % yield). The spectroscopic data match those previously reported in the literature.[32] ^{1}H NMR (400 MHz, $CDCl_3$) δ 7.66 (d, *J* = 8.3 Hz, 2H), 7.56 (d, *J* = 8.2 Hz, 1H), 7.26 - 7.17 (m, 5H), 6.97 (t, *J* = 7.6 Hz, 1H), 2.41 (t, *J* = 7.1 Hz, 2H), 2.36 (s, 3H), 1.60 (p, *J* = 7.1 Hz, 2H), 1.46 - 1.35 (m, 4H), 0.95 (t, *J* = 7.0 Hz, 3H).

N-(2-(6-Chlorohex-1-yn-1-yl)phenyl)-4-methylbenzenesulfonamide (**1l**)

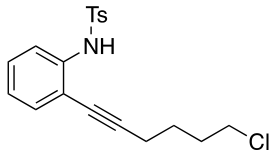

Prepared according to the general procedure **GP1** on a 1.0 mmol scale as a yellow solid (282 mg, 78 % yield). The spectroscopic data match those previously reported in the literature.[32] ^{1}H NMR (400 MHz, $CDCl_3$) δ 7.70 - 7.64 (m, 2H), 7.55 (dd, *J* = 8.3, 1.1 Hz, 1H), 7.26 - 7.16 (m, 5H), 6.97 (td, *J* = 7.6, 1.2 Hz, 1H), 3.59 (t, *J* = 6.4 Hz, 2H), 2.46 (t, *J* = 7.0 Hz, 2H), 2.35 (s, 3H), 1.96 - 1.85 (m, 2H), 1.74 (p, *J* = 7.1 Hz, 2H).

N-(4-Chloro-2-(phenylethynyl)phenyl)-4-methylbenzenesulfonamide (**1m**)

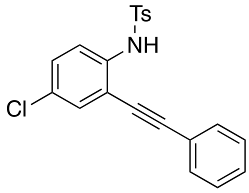

Prepared according to the general procedure **GP1** on a 1.0 mmol scale as a gray solid (324 mg, 85 % yield). The spectroscopic data match those previously reported in the literature.[29] ^{1}H NMR (400 MHz, $CDCl_3$) δ 7.66 (s, 2H), 7.58 (s, 2H), 7.40 (t, *J* = 20.9 Hz, 6H), 7.17 (s, 3H), 2.34 (s, 3H).

4-Methyl-*N*-(4-methyl-2-(phenylethynyl)phenyl)benzenesulfonamide (**1n**)

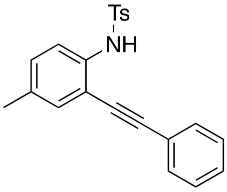

Prepared according to the general procedure **GP1** on a 1.0 mmol scale as a gray solid (322 mg, 89 % yield). The spectroscopic data match those previously reported in the literature.[29] ^{1}H NMR (400 MHz, $CDCl_3$) δ 7.66 (d, *J* = 8.2 Hz, 2H), 7.53 (d, *J* = 8.3 Hz, 1H), 7.45 (dd, *J* = 6.6, 3.0 Hz, 2H), 7.41 - 7.34 (m, 3H), 7.20 - 7.08 (m, 5H), 2.32 (s, 3H), 2.26 (s, 3H).

N-(2-(*p*-Tolylethynyl)phenyl)methanesulfonamide (**1Ms-a**)

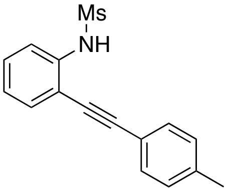

Prepared according to the general procedure **GP1** on a 1.0 mmol scale as a yellow solid (257 mg, 90 % yield). The spectroscopic data match those previously reported in the literature.[31] ^{1}H NMR (400 MHz, $CDCl_3$) δ 7.62 (d, *J* = 8.2 Hz, 1H), 7.54 (d, *J* = 7.3 Hz, 1H), 7.43 (d, *J* = 7.9 Hz, 2H), 7.37 (t, *J* = 7.5 Hz, 1H), 7.18 (dd, *J* = 15.0, 7.7 Hz, 3H), 7.04 (s, 1H), 3.04 (s, 3H), 2.39 (s, 3H).

General Procedure 2 **(GP2):** Synthesis of **1Ns-a** and **1SES-a**.

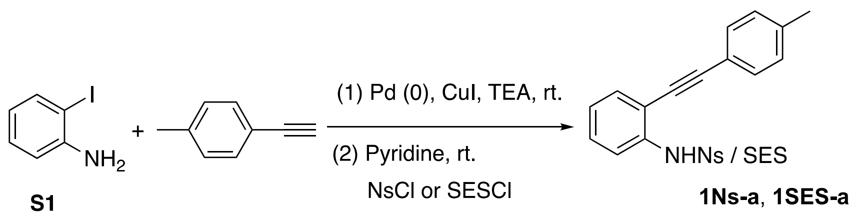

To a solution of 2-iodoaniline **S1** (0.876 g, 4.0 mmol, 1.0 equiv.) in dry Et_3N (5 mL), 1-ethynyl-4-methylbenzene (0.696 g, 6.0 mmol, 1.5 equiv.) and $PdCl_2(PPh_3)_2$ (28 mg, 0.04 mmol, 10 mol%) were added. After 5 min stirring at room temperature, CuI (15 mg, 0.08 mmol, 20 mol%) was added and the reaction mixture stirred at same temperature for completion (4 to 12 h). After the reaction was completed, the reaction mixture was poured into ice-cold water and extracted with EtOAc. The combined organic layers were washed with a brine solution, dried and evaporated under reduced pressure. The crude residue was purified by flash chromatography (Pent/Et_2O: 9/1) to give the product in 92% yield as a yellow solid.
Then the obtained compound (1 mmol) was dissolved in pyridine (10 mL) and 4-nitrobenzenesulfonyl chloride (NsCl, 0.288 g, 1.3 equiv.) or 2-trimethylsilylethylsulfonyl chloride (SES-Cl, 0.261g, 1.3 mmol, 1.3 equiv.) was added at 0 ^{o}C over a period of 15 min. After the addition was complete, the middle reaction was stirred at room temperature for 12 hours. After completion (monitored by TLC), the reaction was quenched with ice-cold water (10 mL) and extracted with dichloromethane (3 x 15 mL). The combined organic layers were washed with a 2N HCl solution and a brine solution, and dried and evaporated to give crude residue. The obtained crude material was purified by flash column chromatography to give pure product (**1Ns-a, 1SES-a**) in good yield.

4-Nitro-*N*-(2-(*p*-tolylethynyl)phenyl)benzenesulfonamide (**1Ns-a**)

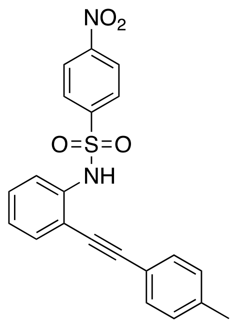

Prepared according to the general procedure **GP2** on a 1.0 mmol scale as a yellow solid (267 mg, 68 % yield). ^{1}H NMR (400 MHz, $CDCl_3$) δ 8.16 (d, *J* = 8.9 Hz, 2H), 7.89 (d, *J* = 8.8 Hz, 2H), 7.66 (dd, *J* = 8.2, 1.1 Hz, 1H), 7.41 - 7.34 (m, 2H), 7.29 (d, *J* = 8.2 Hz, 2H), 7.23 - 7.13 (m, 4H), 2.41 (s, 3H). ^{13}C NMR (101 MHz, $CDCl_3$) δ 150.26, 144.58, 139.81, 136.08, 132.16, 131.34, 129.66, 129.47, 128.49, 125.98, 124.08, 122.14, 118.44, 116.24, 96.69, 82.78, 21.59. HRMS (APCI) calc. for $C_{21}H_{17}N_2O_4S^+$ $[M+H]^+$: 372.1448, found 372.1449.

N-(2-(*p*-Tolylethynyl)phenyl)-2-(trimethylsilyl)ethane-1-sulfonamide (**1Ns-a**)

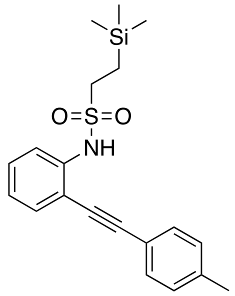

Prepared according to the general procedure **GP2** on a 1.0 mmol scale as a yellow solid (156 mg, 42 % yield).^{1}H NMR (400 MHz, $CDCl_3$) δ 7.66 (d, *J* = 8.2 Hz, 1H), 7.52 (dd, *J* = 7.8, 1.5 Hz, 1H), 7.42 (d, *J* = 7.9 Hz, 2H), 7.34 (ddd, *J* = 8.6, 7.6, 1.5 Hz, 1H), 7.19 (d, *J* = 7.8 Hz, 2H), 7.13 (dd, *J* = 8.2, 7.1 Hz, 1H), 7.05 (s, 1H), 3.06 - 2.97 (m, 2H), 2.39 (s, 3H), 1.14 - 1.07 (m, 2H), -0.08 (s, 9H). ^{13}C NMR (101 MHz, $CDCl_3$) δ 139.50, 137.87, 132.20, 131.44, 129.74, 129.33, 124.42, 119.38, 118.73, 114.14, 97.13, 83.25, 47.86, 21.53, 10.52, -2.24. HRMS (APCI) calc. for $C_{20}H_{26}NO_2SSi^+$ $[M+H]^+$: 372.1448, found 372.1449.

Synthesis of starting material **1o**

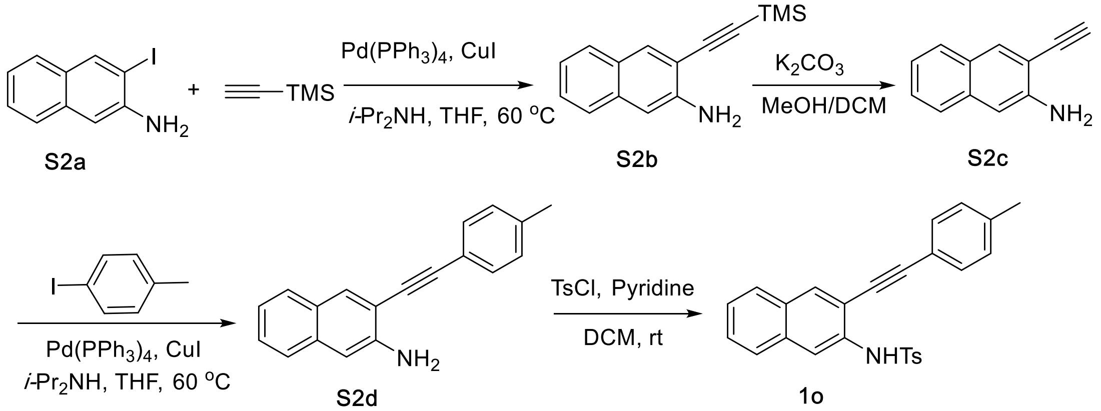

In a sealed tub, a solution of 3-iodo-2-naphthalenamine **S2a** (1.05 g, 3.93 mmol) in a 1:1 mixture of THF/*i*-Pr_2NH (21 mL) was stirred for 12 min under N_2. Then, ethynyltrimethylsilane (2.25 mL, 15.69 mmol) was added via syringe. After an additional 5 min stirring, CuI (27 mg, 0.15 mmol) and $Pd(PPh_3)_4$ (49.5 mg, 0.03 mmol) were added to the reaction mixture. The reaction medium was then stirred for an additional 2 min. Then, the tube was sealed and heated at 60 °C for 12 h. After cooling, the mixture was concentrated and the residue was purified by column chromatography on silica gel (5:1, hexanes/EtOAc) to give **S2b** (779 mg, 83 % yield) as a light orange-colored solid.

To a solution of alkyne **S2b** (1.0 equiv.) in MeOH/$CHCl_3$ (1:1, 0.01 M) was added K_2CO_3 (3 equiv.). The suspension was stirred at room temperature for 3 h, and then filtered through a pad of celite. After evaporation of the solvent, the crude material was used directly in the next step. To a solution of 1-iodo-4-methylbenzene and terminal acetylene **S2c** (1.0 equiv.) in 1:1 THF/*i*-Pr_2NH, were added 1 mol% of $Pd(PPh_3)_4$ and 2 mol% of CuI. The solution was heated to 60 °C for 12 h under an N_2

atmosphere. The reaction mixture was cooled, concentrated *in vacuo* and purified by flash chromatography to give the desired product **S2d** as a yellow-orange solid in 85 %.[33]

The obtained compound **S2d** (1 mmol) was dissolved in dichloromethane (20 ml) and pyridine (3 equiv.) followed by addition of tosyl chloride or methanesulfonyl chloride (1.3 equiv.) were added at 0 °C over a period of 15 min. After the addition was complete, the reaction was stirred at room temperature for 12 hours. After completion (monitored by TLC), the reaction was quenched with ice-cold water (10 mL) and extracted with dichloromethane (3 x 15 mL). The combined organic layers were washed with a 2N HCl solution, a brine solution, dried over $MgSO_4$ and evaporated under reduced pressure to give a crude residue. The obtained crude material was purified by flash column chromatography to give pure product **1o** in 92 % yield as a yellow solid (378 mg). ^1H NMR (400 MHz, $CDCl_3$) δ 8.03 (s, 1H), 7.90 (s, 1H), 7.79 (d, *J* = 8.2 Hz, 1H), 7.70 (dd, *J* = 7.8, 5.7 Hz, 3H), 7.51 - 7.45 (m, 1H), 7.41 (t, *J* = 7.8 Hz, 3H), 7.35 (s, 1H), 7.22 (d, *J* = 7.9 Hz, 2H), 7.13 (d, *J* = 8.1 Hz, 2H), 2.42 (s, 3H), 2.30 (s, 3H). ^{13}C NMR (101 MHz, $CDCl_3$) δ 143.92, 139.48, 136.04, 133.42, 133.33, 132.21, 131.51, 130.21, 129.56, 129.35, 127.71, 127.45, 127.36, 127.28, 125.91, 118.84, 117.48, 114.53, 96.36, 83.42, 21.58, 21.46. HRMS (APCI) calc. for $C_{26}H_{22}NO_2S^+$ $[M+H]^+$: 412.1293, found 412.1289.

b. Synthesis of iodoalkynes derivatives 2 and 7

General Procedure **3 (GP3):** Synthesis of **(Iodoethynyl)benzene** Derivatives **2a**-**2k**[34]

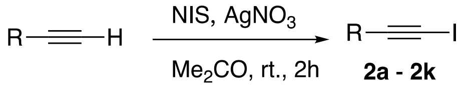

To a stirred solution of the terminal alkyne (2 mmol, 1equiv.) in acetone (5 mL) was added NIS (2.2 mmol, 1.1 equiv.) and $AgNO_3$ (0.2 mmol, 10 mol%). The reaction mixture was stirred at room temperature for 3 h. After completion, the solvent was removed under reduced pressure and the residue was filtered through a pad of celite with petroleum ether. Removal of the solvent under reduced pressure followed by purification by flash column chromatography on silica gel (petroleum ether as eluent) afforded compounds **2a-2k** in 78 – 98 % yield.

1-Fluoro-4-(iodoethynyl)benzene (**2a**)

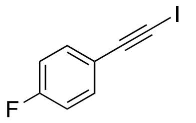

Following general procedure **GP3** with 1-ethynyl-4-fluorobenzene (480 mg, 4 mmol) to afford **2a** (0.88 g, 3.6 mmol, 90 %) as colorless oil. The spectroscopic data match those previously reported in the literature.[3] ^1H NMR (400 MHz, $CDCl_3$) δ 7.45 - 7.39 (m, 1H), 7.04 - 6.98 (m, 1H). ^{19}F NMR (376 MHz, $CDCl_3$) δ -109.72.

(Iodoethynyl)benzene (**2b**)

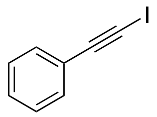

Following general procedure **GP3** with phenylacetylene (408 mg, 4 mmol) to afford **2b** (0.866 g, 3.8 mmol, 95 %) as colorless oil. The spectroscopic data match those previously reported in the literature.[3] ^{1}H NMR (400 MHz, $CDCl_3$) δ 7.44 (m, *J* = 7.4, 2.3 Hz, 2H), 7.32 (m, *J* = 5.2, 2.0 Hz, 3H).

1-(Iodoethynyl)-4-methylbenzene (**2c**)

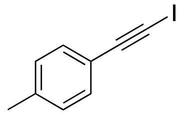

Following general procedure **GP3** 1-ethynyl-4-methylbenzene (464 mg, 4 mmol) to afford **2c** (0.94 g, 3.9 mmol, 98 %) as colorless oil. The spectroscopic data match those previously reported in the literature.[3] ^{1}H NMR (400 MHz, $CDCl_3$) δ 7.33 (m, *J* = 7.9 Hz, 2H), 7.11 (m, *J* = 7.8 Hz, 2H), 2.35 (s, 3H).

1-(Iodoethynyl)-4-methoxybenzene (**2d**)

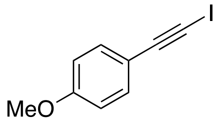

Following general procedure **GP3** with 1-ethynyl-4-methoxybenzene (528 mg, 4 mmol) to afford **2d** (0.94 g, 3.8 mmol, 98 %) as white solid. The spectroscopic data match those previously reported in the literature.[3] ^{1}H NMR (400 MHz, $CDCl_3$) δ 7.47 - 7.34 (m, 2H), 6.82 - 6.85(m, *J* = 8.4 Hz, 2H), 3.81 (s, 3H).

1-(Iodoethynyl)-2-methoxybenzene (**2e**)

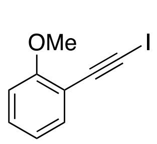

Following general procedure **GP3** with 1-ethynyl-2-methoxybenzene (528, 4 mmol) to afford **2e** (0.95 g, 3.7 mmol, 93 %) as white solid. The spectroscopic data match those previously reported in the literature.[35] ^{1}H NMR (400 MHz, $CDCl_3$) δ 7.40 (d, J = 7.6 Hz, 1H), 7.30 (d, *J* = 7.8 Hz, 1H), 6.92 - 6.85 (m, 2H), 3.88 (s, 3H).

1-Fluoro-2-(iodoethynyl)benzene (**2f**)

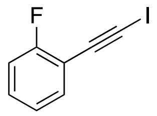

Following general procedure **GP3** with 1-ethynyl-2-fluorobenzene (480 mg, 4 mmol) to afford **2f** (0.86 g, 3.5 mmol, 88 %) as colorless oil. The spectroscopic data match those previously reported in the literature.[36] ^{1}H NMR (400 MHz, $CDCl_3$) δ 7.47 - 7.40 (m, 1H), 7.34 - 7.27 (m, 1H), 7.08 (q, *J* = 9.5, 8.7 Hz, 2H). ^{19}F NMR (376 MHz, $CDCl_3$) δ -110.07.

1-Fluoro-3-(iodoethynyl)benzene (**2g**)

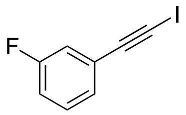

Following general procedure **GP3** with 1-ethynyl-2-fluorobenzene (480 mg, 4 mmol) to afford **2g** (0.85 g, 3.48 mmol, 87 %) as colorless oil. The spectroscopic data match those previously reported in the literature.[3] ^{1}H NMR (400 MHz, $CDCl_3$) δ 7.28 (d, *J* = 8.5 Hz, 1H), 7.23 (d, *J* = 7.6 Hz, 1H), 7.14 (d, *J* = 9.2 Hz, 1H), 7.08 - 7.01 (m, 1H). ^{19}F NMR (376 MHz, $CDCl_3$) δ -112.5.

1-Chloro-2-(iodoethynyl)benzene (**2h**)

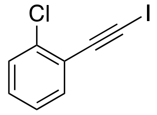

Following general procedure **GP3** with 1-chloro-2-ethynylbenzene (544 mg, 4 mmol) to afford **2h** (0.84 g, 3.2 mmol, 80 %) as brown oil. The spectroscopic data match those previously reported in the literature.[3] ^{1}H NMR (400 MHz, $CDCl_3$) δ 7.50 - 7.44 (m, 1H), 7.42 - 7.36 (m, 1H), 7.27 - 7.18 (m, 2H).

Methyl 4-(iodoethynyl)benzoate (**2i**)

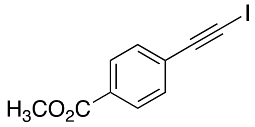

Following general procedure **GP3** with 1-ethynyl-4-(trifluoromethyl)benzene (640 mg, 4 mmol) to afford **2i** (0.89 g, 3.1 mmol, 78 %) as yellow solid. The spectroscopic data match those previously reported in the literature.[37] ^{1}H NMR (400 MHz, $CDCl_3$) δ 7.98 (d, *J* = 8.0 Hz, 2H), 7.49 (d, *J* = 8.0 Hz, 2H), 3.91 (s, 3H).

1-(Iodoethynyl)-4-(trifluoromethyl)benzene (**2j**)

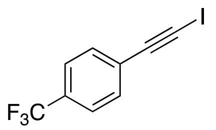

Following general procedure **GP3** with 1-ethynyl-4-(trifluoromethyl)benzene (680 mg, 4 mmol) to afford **2j** (1.0 g, 3.4 mmol, 85 %) as yellow oil. The spectroscopic data match those previously reported in the literature.[3] ^{1}H NMR (300 MHz, $CDCl_3$) δ 7.64–7.52 (m, 4H). ^{19}F NMR (376 MHz, $CDCl_3$) δ -62.95.

1-Iodohept-1-yne (**2k**)

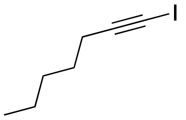

Following general procedure **GP3** with hept-1-yne (384 mg, 4 mmol) to afford **2k** (0.78 g, 3.5 mmol, 88 %) as yellow oil. The spectroscopic data match those previously reported in the literature.[3] ^{1}H NMR (400 MHz, $CDCl_3$) δ 2.35 (t, *J* = 6.8 Hz, 2H), 1.55-1.48 (m, 2H), 1.40-1.27 (m, 4H), 0.90 (t, *J* = 7.2 Hz, 3H).

c. Synthesis of methyl benzyl(iodoethynyl)carbamate 7[39]

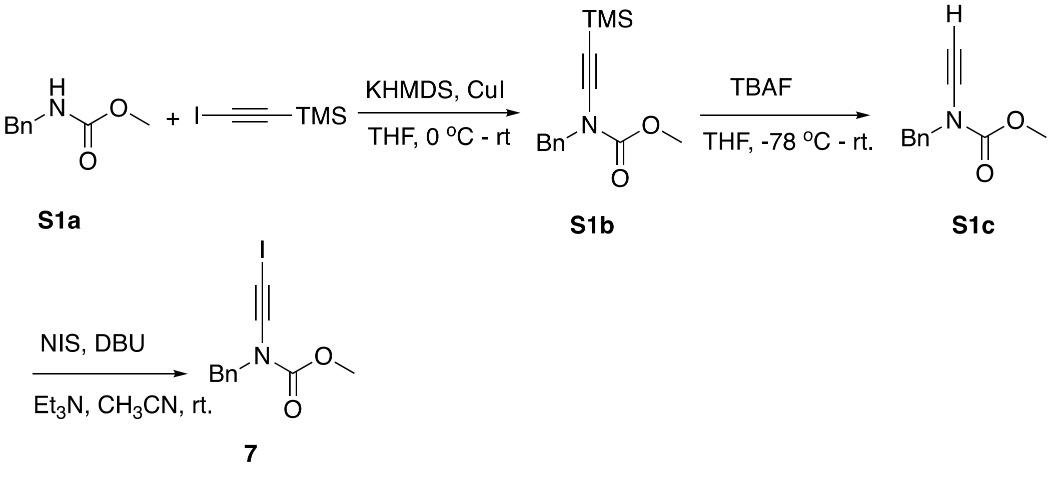

In a solution of **S1a** (1.0 equiv.) in THF (10 mL) was added, in one portion, CuI (1.73 g, 9.10 mmol, 1.0 equiv.) at 0°C, then the mixture was stirred 15 minutes at the same temperature. The resulting grass-green suspension was allowed to warm to rt and stirred for a total of 3 h. A solution of

iodo(trimethylsilyl)acetylene[39] (2.24 g, 10 mmol, 1.1 equiv.) in 8 mL of THF was added to the dark green reaction mixture dropwise over 1 h via syringe, and the reaction mixture was stirred at rt for 16 h. The resulting brown solution was diluted with 100 mL of Et_2O and washed with three 75 mL portions of a 2:1 mixture of brine and concentrated NH_4OH solution. The combined aqueous phases were extracted with two 80 mL portions of Et_2O, and the combined organic phases were washed with two 80 mL portions of 3 M aqueous HCl solution and 80 mL of brine, dried over $MgSO_4$, filtered, and concentrated to afford 4.23 g of dark brown oil. Column chromatography on silica gel (gradient elution with 0 to 5% EtOAc - petroleum ether) afforded 1.31 g (58 %) of ynamide **S1b** as a yellow oil. A 100-mL, round-bottomed flask equipped with a rubber septum and argon inlet needle was charged with ynamide **S1b** (0.500 g, 1.91 mmol, 1.0 equiv.) and 25 mL of THF. The yellow solution was cooled at -78 °C and TBAF (2.1 mL, 1.0 M in THF, 2.1 mmol, 1.1 equiv.) was added dropwise via syringe over 5 min. The resulting brown suspension was stirred for 10 min and then diluted with 25 mL of Et_2O, 25 mL of water, and 5 mL of brine. The aqueous phase was extracted with two 20 mL portions of Et_2O, and the combined organic phases were washed with 30 mL of brine, dried over $MgSO_4$, filtered, and concentrated under reduce pression to afford 0.441 g of orange oil. Column chromatography on 12 g of acetone-deactivated silica gel (elution with 4% EtOAc and 1% Et_3N in petroleum ether) afforded 0.294 g (81 %) of ynamide **S1c** as a viscous pale-yellow oil.

To a stirred solution of ynamide **S1c** (290 mg, 1.5 mmol) in MeCN (5.0 mL) was added NIS (383 mg, 1.7 mmol) and DBU (258 mg, 1.7 mmol). The mixture was stirred at room temperature for 5 min. The reaction mixture was poured into water and then extracted with EtOAc (3 × 20 mL). The combined organic phase was washed with saturated brine (10 mL), dried over $MgSO_4$ and filtered. The solvent was removed under reduced pressure and the crude product was purified by flash chromatography (silica gel, petroleum ether or n-pentane as eluent) to give **7** (425 mg, 90 %) as a yellow solid. The spectroscopic data match those previously reported in the literature.[38] ^1H NMR (300 MHz, $CDCl_3$) δ 7.41 – 7.29 (m, 5H), 4.63 (s, 2H), 3.81 (s, 3H).

d. Synthesis of vinylgold(I) complex 6

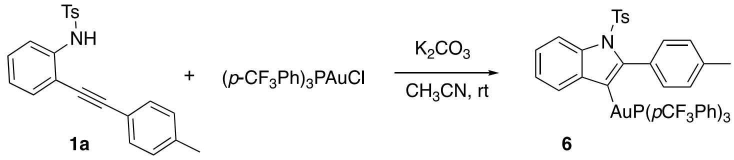

In a 25-mL Schlenk tube, 4-methyl-N-(2-(p-tolylethynyl)phenyl)benzenesulfonamide **1a** (0.1 mmol, 36 mg, 1.0 equiv.), (p-$CF_3Ph)_3$PAuCl (0.1 mmol, 69.5 mg, 1.0 equiv.), and K_2CO_3 (0.25 mmol, 34 mg, 2.5 equiv.) was dissolved in 10 mL of dry CH_3CN. The reaction mixture was stirred for 16 h at room temperature, filtered over a small column of basic aluminum oxide and wash with 20 mL of dry CH_3CN. The solvent was removed under reduced pressure without heating. Vinylgold(I) complex **6** was obtained as a light-yellow solid, 94 mg, 92 %. M.P. = 270 °C. ^1H NMR (400 MHz, $CDCl_3$) δ 8.27 (d, J = 8.2 Hz, 1H), 7.71 (dd, J = 8.3, 1.9 Hz, 6H), 7.66 - 7.60 (m, 3H), 7.56 (dd, J = 11.8, 8.0 Hz, 6H), 7.35 (d, J = 8.1 Hz, 2H), 7.24 (d, J = 1.2 Hz, 1H), 7.14 (d, J = 7.7 Hz, 3H), 7.02 (d, J = 8.1 Hz, 2H), 2.44 (s, 3H), 2.26 (s, 3H). ^{13}C NMR (101 MHz, $CDCl_3$) δ 155.54, 154.36, 147.35, 143.73, 139.67, 138.71, 136.84, 135.27, 134.75, 134.61, 134.02, 133.86, 133.69, 133.37, 131.14, 129.02, 127.65, 126.78, 126.26, 126.11, 124.61, 123.72, 123.32, 121.89, 116.16, 21.43, 21.34. ^{19}F NMR (376 MHz, $CDCl_3$) δ -63.28. ^{31}P NMR (162 MHz, $CDCl_3$) δ 44.96. HRMS (ESI) calc. for $C_{43}H_{30}AuF_9NaO_2P^+$ $[M+Na]^+$: 1046.1257, found 1046.1259.

e. Synthesis of indoles 3 and 8

General Procedure **4 (GP4):** *Ethynylative Cyclization of o-alkynyl anilines derivatives with Iodoalkynes*

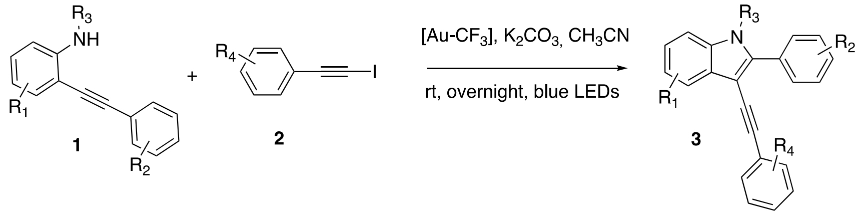

The gold(I) complex (p-$CF_3Ph)_3PAuCl$ (5 mol %), K_2CO_3 (2.5 equiv.), the appropriate iodoalkynes **2** (0.33 mmol,1.1 equiv.) and *o*-alkynyl anilines derivatives **1** (0.3 mmol, 1.0 equiv.) were introduced in a Schlenk tube equipped with a magnetic stirring bar in which MeCN (4.5 mL) was added. The mixture was degassed using three freeze pump-thaw cycles and purged with argon, then irradiated with blue LEDs light for 15 h (unless mentioned). The stirring speed was equal to or more than 1200 rpm. The reaction was quenched with EtOAc (5 mL) and a 2 M HCl solution (6 mL) and the solution was extracted by EtOAc (3 × 5 mL). The combined organic layer was dried over $MgSO_4$, filtered and concentrated under reduced pressure to give the crude product. The residue was purified by flash chromatography on silica gel to afford the desired product **3** or the yield was determined by ^{1}H NMR using 1,3,5-trimethoxybenzene as internal standard.

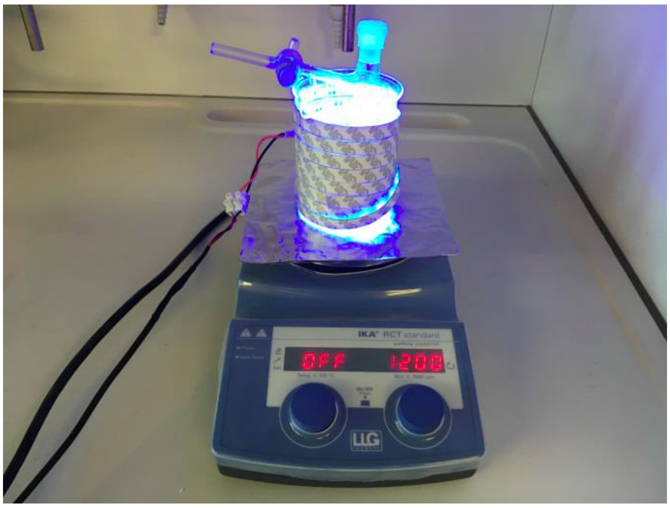

Photoreactor equipped with LED stripes (λmax = 450 nm) on a beaker (the diameter is 7.5 cm, the height is 9.5 cm). An aluminum foil was placed between the stirring plate and the beaker in order to reflect the light.

3-((4-Fluorophenyl)ethynyl)-2-(*p*-tolyl)-1-tosyl-1*H*-indole (**3aa**)

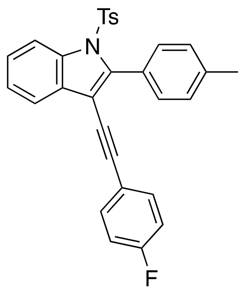

Following general procedure **GP4** with 4-methyl-*N*-(2-(*p*-tolylethynyl)phenyl)benzenesulfonamide **1a** (108 mg, 0.3 mmol) and 1-fluoro-4-(iodoethynyl)benzene **2a** (81 mg, 0.33 mmol). The crude product was purified by flash column chromatography (Petroleum ether : EtOAc = 20 : 1) to afford **3aa** as a yellow solid (89 mg, 62 %). Mp 108 ^{o}C. ^{1}H NMR (400 MHz, $CDCl_3$) δ 8.32 (d, *J* = 8.3 Hz, 1H), 7.63 (d, *J* = 6.4 Hz, 1H), 7.54 (d, *J* = 8.1 Hz, 2H), 7.44 - 7.39 (m, 1H), 7.38 - 7.36 (m, 1H), 7.36 - 7.32 (m, 2H), 7.32 - 7.29 (m, 2H), 7.28 (d, *J* = 3.1 Hz, 2H), 7.05 (d, *J* = 7.7 Hz, 2H), 6.99 (t, *J* = 8.7 Hz, 2H), 2.48 (s, 3H), 2.29 (s, 3H). ^{13}C NMR (101 MHz, $CDCl_3$) δ 163.68, 161.19, 144.81, 143.80, 139.16, 137.12, 134.51, 133.34, 133.26, 131.07, 130.81, 129.30, 128.07, 127.77, 126.86, 125.64, 124.69, 119.94, 119.32, 119.28, 116.72, 115.71, 115.49, 107.68, 93.50, 81.36, 21.60, 21.54. ^{19}F NMR (376 MHz, $CDCl_3$) δ -110.77. HRMS (APCI) calc. for $C_{30}H_{23}FNO_2S^+$ $[M+H]^+$: 480.1428, found 480.1426.

3-(Phenylethynyl)-2-(*p*-tolyl)-1-tosyl-1*H*-indole (**3ab**)

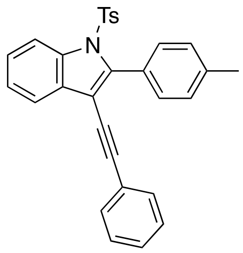

Following general procedure **GP4** with 4-methyl-*N*-(2-(*p*-tolylethynyl)phenyl)benzenesulfonamide **1a** (108 mg, 0.3 mmol) and (iodoethynyl)benzene **2b** (75 mg, 0.33 mmol). The crude product was purified by flash column chromatography (Petroleum ether : EtOAc = 15 : 1) to afford **3ab** as a white solid (86 mg, 62 %). Mp 118 ^{o}C. ^{1}H NMR (400 MHz, $CDCl_3$) δ 8.32 (d, *J* = 8.2 Hz, 1H), 7.65 (d, *J* = 7.4 Hz, 1H), 7.56 (d, *J* = 8.1 Hz, 2H), 7.44 – 7.35 (m, 4H), 7.34 – 7.27 (m, 7H), 7.05 (d, *J* = 8.2 Hz, 2H), 2.48 (s, 3H), 2.29 (s, 3H) . ^{13}C NMR (101 MHz, $CDCl_3$) δ 144.79, 143.77, 139.11, 137.15, 134.48, 131.44, 131.09, 130.94, 129.29, 128.28, 128.22, 128.16, 128.07, 127.81, 126.86, 125.61, 124.69, 123.21, 120.01, 116.73, 94.66, 81.64, 21.62, 21.55. HRMS (APCI) calc. for $C_{30}H_{24}NO_2S^+$ $[M+H]^+$: 462.1522, found 462.1521.

2-(*p*-Tolyl)-3-(*p*-tolylethynyl)-1-tosyl-1*H*-indole (**3ac**)

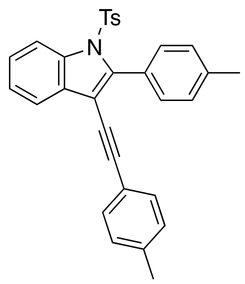

Following general procedure **GP4** with 4-methyl-*N*-(2-(*p*-tolylethynyl)phenyl)benzenesulfonamide **1a** (108 mg, 0.3 mmol) and 1-(iodoethynyl)-4-methylbenzene **2c** (80 mg, 0.33 mmol). The crude product was purified by flash column chromatography (Petroleum ether : EtOAc = 15 : 1) to afford **3ac** as a white solid (101 mg, 71 %). Mp 118 °C. ^{1}H NMR (400 MHz, $CDCl_3$) δ 8.32 (d, *J* = 8.3 Hz, 1H), 7.65 (d, *J* = 7.6 Hz, 1H), 7.57 (d, *J* = 7.7 Hz, 2H), 7.44 – 7.33 (m, 2H), 7.29 (d, *J* = 8.1 Hz, 6H), 7.11 (d, *J* = 7.8 Hz, 2H), 7.05 (d, *J* = 8.0 Hz, 2H), 2.47 (s, 3H), 2.34 (s, 3H), 2.29 (s, 3H). ^{13}C NMR (101 MHz, $CDCl_3$) δ 144.74, 143.49, 139.01, 138.40, 137.17, 134.45, 131.34, 131.07, 131.02, 129.27, 129.04, 128.04, 127.87, 126.84, 125.56, 124.66, 120.11, 120.03, 116.73, 108.15, 94.87, 80.91, 21.59, 21.52, 21.49. HRMS (APCI) calc. for $C_{31}H_{26}NO_2S^+$ $[M+H]^+$: 476.1679, found 476.1671.

3-((4-Methoxyphenyl)ethynyl)-2-(*p*-tolyl)-1-tosyl-1*H*-indole (**3ad**)

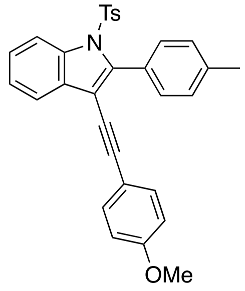

Following general procedure **GP4** with 4-methyl-*N*-(2-(*p*-tolylethynyl)phenyl)benzenesulfonamide **1a** (108 mg, 0.3 mmol) and 1-(iodoethynyl)-4-methoxybenzene **2d** (85 mg, 0.33 mmol). The crude product was purified by flash column chromatography (Petroleum ether : EtOAc = 20 : 1) to afford **3ad** as a yellow solid (99 mg, 67 %). Mp 110 °C; ^{1}H NMR (400 MHz, $CDCl_3$) δ 8.32 (d, *J* = 8.2 Hz, 1H), 7.64 (d, *J* = 8.4 Hz, 1H), 7.57 (d, *J* = 8.1 Hz, 2H), 7.43 - 7.38 (m, 1H), 7.33 (d, *J* = 8.8 Hz, 3H), 7.28 (s, 4H), 3.80 (s, 3H), 2.47 (s, 3H), 2.29 (s, 3H); ^{13}C NMR (101 MHz, $CDCl_3$) δ 159.61, 144.72, 143.22, 138.95, 137.18, 134.42, 132.90, 131.05, 129.25, 128.02, 127.94, 126.84, 125.54, 124.65, 120.03, 116.74, 115.30, 113.95, 108.29, 94.68, 80.21, 55.28, 21.59, 21.51. HRMS (APCI) calc. for $C_{31}H_{26}NO_3S^+$ $[M+H]^+$: 492.1550, found 492.1545.

3-((2-Methoxyphenyl)ethynyl)-2-(*p*-tolyl)-1-tosyl-1*H*-indole (**3ae**)

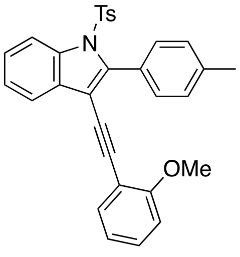

Following general procedure **GP4** with 4-methyl-*N*-(2-(*p*-tolylethynyl)phenyl)benzenesulfonamide **1a** (108 mg, 0.3 mmol) and 1-(iodoethynyl)-2-methoxybenzene **2e** (85 mg, 0.33 mmol). The crude product was purified by flash column chromatography (Petroleum ether : EtOAc = 20 : 1) to afford **3ae** as a Yellow solid (93 mg, 63 %). Mp 107 °C. ^{1}H NMR (400 MHz, $CDCl_3$) δ 8.33 (d, *J* = 8.2 Hz, 1H), 7.71 (d, *J* = 7.6 Hz, 1H), 7.64 (d, *J* = 7.8 Hz, 2H), 7.46 - 7.34 (m, 3H), 7.30 (dq, *J* = 10.1, 4.1 Hz, 5H), 7.05 (d, *J* = 8.0 Hz, 2H), 6.93 - 6.84 (m, 2H), 3.88 (s, 3H), 2.49 (s, 3H), 2.30 (s, 3H). ^{13}C NMR (101 MHz, $CDCl_3$) δ 159.97, 144.71, 143.31, 138.85, 137.23, 134.38, 133.15, 131.22, 131.15, 129.68, 129.24, 127.97, 127.89, 126.84, 125.52, 124.69, 120.40, 120.19, 116.74, 112.52, 110.67, 108.52, 91.27, 85.64, 55.76, 21.60, 21.50. HRMS (APCI) calc. for $C_{31}H_{26}NO_3S^+$ $[M+H]^+$: 492.1628, found 492.1625.

3-((2-Fluorophenyl)ethynyl)-2-(*p*-tolyl)-1-tosyl-1*H*-indole (**3af**)

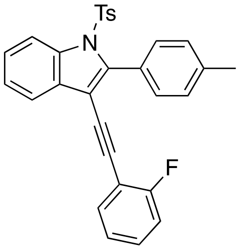

Following general procedure **GP4** with 4-methyl-*N*-(2-(*p*-tolylethynyl)phenyl)benzenesulfonamide **1a** (108 mg, 0.3 mmol) and 1-fluoro-2-(iodoethynyl)benzene **2f** (81 mg, 0.33 mmol). The crude product was purified by flash column chromatography (Petroleum ether : EtOAc = 20 : 1) to afford **3af** as a Yellow solid (69 mg, 48 %). Mp 102 °C. ^{1}H NMR (400 MHz, $CDCl_3$) δ 8.33 (d, *J* = 8.2 Hz, 1H), 7.68 (d, *J* = 7.6 Hz, 1H), 7.59 (d, *J* = 7.7 Hz, 2H), 7.42 (t, *J* = 7.7 Hz, 1H), 7.39 – 7.34 (m, 2H), 7.30 (dd, *J* = 8.1, 4.0 Hz, 5H), 7.06 (t, *J* = 8.0 Hz, 4H), 2.48 (s, 3H), 2.29 (s, 3H). ^{13}C NMR (101 MHz, $CDCl_3$) δ 163.83, 161.32, 144.84, 144.08, 139.17, 137.12, 134.43, 133.14, 131.07, 130.84, 129.92, 129.85, 129.30, 128.09, 127.60, 126.84, 125.66, 124.79, 123.88, 123.84, 120.05, 116.70, 115.53, 115.32, 111.95, 111.79, 107.64, 88.05, 86.81, 86.78, 21.59, 21.52. ^{19}F NMR (376 MHz, $CDCl_3$) δ -109.56. HRMS (APCI) calc. for $C_{30}H_{23}FNO_2S^+$ $[M+H]^+$: 480.1355, found 480.1356.

3-((3-Fluorophenyl)ethynyl)-2-(*p*-tolyl)-1-tosyl-1*H*-indole (**3ag**)

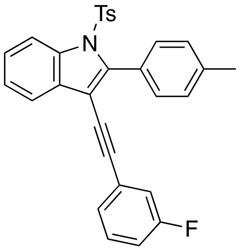

Following general procedure **GP4** with 4-methyl-*N*-(2-(*p*-tolylethynyl)phenyl)benzenesulfonamide **1a** (108 mg, 0.3 mmol) and 1-fluoro-3-(iodoethynyl)benzene **2g** (81 mg, 0.33 mmol). The crude product was purified by flash column chromatography (Petroleum ether : EtOAc = 20 : 1) to afford **3ag** as a Yellow solid (87 mg, 61 %). Mp 104 °C. ^{1}H NMR (400 MHz, $CDCl_3$) δ 8.33 (d, *J* = 8.2 Hz, 1H), 7.64 (d, *J* = 7.6 Hz, 1H), 7.54 (d, *J* = 7.8 Hz, 2H), 7.41 (d, *J* = 7.6 Hz, 1H), 7.37 (d, *J* = 7.5 Hz, 1H), 7.30 (dd, *J* = 8.1, 4.6 Hz, 4H), 7.25 (d, *J* = 5.4 Hz, 1H), 7.15 (d, *J* = 7.7 Hz, 1H), 7.06 (d, *J* = 8.3 Hz, 3H), 7.00 (d, *J* = 2.5 Hz, 1H), 2.48 (s, 3H), 2.30 (s, 3H). ^{13}C NMR (101 MHz, $CDCl_3$) δ 163.53, 161.08, 144.87, 144.26, 139.29, 137.08, 134.53, 131.07, 130.67, 129.89, 129.81, 129.33, 128.10, 127.65, 127.30, 127.27, 126.85, 125.69, 125.08, 124.99, 124.73, 119.93, 118.24, 118.01, 116.69, 115.62, 115.41, 107.36, 93.33, 93.30, 82.71, 21.60, 21.54. ^{19}F NMR (376 MHz, $CDCl_3$) δ -112.96. HRMS (APCI) calc. for $C_{30}H_{23}FNO_2S^+$ $[M+H]^+$: 480.1428, found 480.1427.

.

3-((2-Chlorophenyl)ethynyl)-2-(*p*-tolyl)-1-tosyl-1*H*-indole (**3ah**)

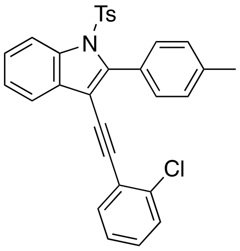

Following general procedure **GP4** with 4-methyl-*N*-(2-(*p*-tolylethynyl)phenyl)benzenesulfonamide **1a** (108 mg, 0.3 mmol) and 1-chloro-2-(iodoethynyl)benzene **2h** (86 mg, 0.33 mmol). The crude product was purified by flash column chromatography (Petroleum ether : EtOAc = 15 : 1) to afford **3ah** as a yellow solid (67 mg, 45 %). Mp 109 °C. ^{1}H NMR (400 MHz, $CDCl_3$) δ 8.33 (d, *J* = 8.2 Hz, 1H), 7.72 (d, *J* = 7.6 Hz, 1H), 7.59 (s, 2H), 7.45 - 7.35 (m, 4H), 7.30 (dd, *J* = 8.3, 2.1 Hz, 4H), 7.20 (ddd, *J* = 9.3, 6.8, 1.9 Hz, 2H), 7.06 (d, *J* = 8.0 Hz, 2H), 2.48 (s, 3H), 2.30 (s, 3H). ^{13}C NMR (101 MHz, $CDCl_3$) δ 144.86, 144.17, 139.19, 137.08, 135.59, 134.51, 133.10, 131.16, 130.87, 129.33, 129.21, 129.13, 128.10, 127.62, 126.86, 126.37, 125.67, 124.79, 123.18, 120.14, 116.63, 107.60, 91.41, 86.93, 77.32, 77.00, 76.68, 21.60, 21.53. HRMS (APCI) calc. for $C_{30}H_{23}ClNO_2S^+$ $[M+H]^+$: $[M+H]^+$: 496.1133, found 496.1136.

Methyl 4-((2-(*p*-tolyl)-1-tosyl-1*H*-indol-3-yl)ethynyl)benzoate (**3ai**)

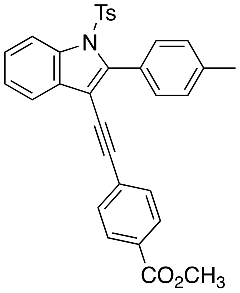

Following general procedure **GP4** with 4-methyl-*N*-(2-(*p*-tolylethynyl)phenyl)benzenesulfonamide **1a** (108 mg, 0.3 mmol) and methyl 4-(iodoethynyl)benzoate **2i** (94 mg, 0.33 mmol). The crude product was purified by flash column chromatography (Petroleum ether : EtOAc = 15 : 1) to afford **3ai** as a yellow solid (87 mg, 56 %). Mp 125 °C. ^{1}H NMR (400 MHz, $CDCl_3$) δ 8.33 (d, *J* = 8.2 Hz, 1H), 7.96 (d, *J* = 8.4 Hz, 2H), 7.68 - 7.63 (m, 1H), 7.58 - 7.52 (m, 2H), 7.45 - 7.40 (m, 3H), 7.37 (dd, *J* = 7.5, 1.1 Hz, 1H), 7.33 - 7.27 (m, 4H), 7.08 - 7.01 (m, 2H), 3.91 (s, 3H), 2.49 (s, 3H), 2.29 (s, 3H). ^{13}C NMR (101 MHz, $CDCl_3$) δ 166.46, 144.90, 144.50, 139.35, 137.08, 134.51, 131.23, 131.10, 130.58, 129.44, 129.33, 128.10, 127.88, 127.59, 126.84, 125.73, 124.75, 119.93, 116.69, 107.28, 93.92, 84.92, 52.19, 21.61, 21.53. HRMS (APCI) calc. for $C_{32}H_{26}NO_4S^+$ [M+H]$^+$: 520.1577, found]$^+$: 520.1577.

2-(*p*-Tolyl)-1-tosyl-3-((4-(trifluoromethyl)phenyl)ethynyl)-1*H*-indole (**3aj**)

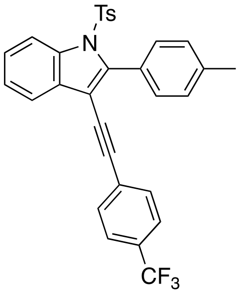

Following general procedure **GP4** with 4-methyl-*N*-(2-(*p*-tolylethynyl)phenyl)benzenesulfonamide **1a** (108 mg, 0.3 mmol) and 1-(iodoethynyl)-4-(trifluoromethyl)benzene **2j** (98 mg, 0.33 mmol). The crude product was purified by flash column chromatography (Petroleum ether : EtOAc = 15 : 1) to afford **3aj** as a yellow solid (92 mg, 58 %). Mp 119 °C. ^{1}H NMR (400 MHz, $CDCl_3$) δ 8.34 (d, *J* = 8.3 Hz, 1H), 7.65 (d, *J* = 7.6 Hz, 1H), 7.55 (dd, *J* = 8.3, 2.5 Hz, 4H), 7.44 (dd, *J* = 14.3, 7.6 Hz, 3H), 7.41 - 7.26 (m, 6H), 7.07 (d, *J* = 8.2 Hz, 2H), 2.49 (s, 3H), 2.30 (s, 3H). ^{13}C NMR (101 MHz, $CDCl_3$) δ 144.95, 144.61, 139.40, 137.07, 134.57, 131.57, 131.12, 130.53, 129.37, 128.12, 127.59, 126.88, 125.77, 125.27, 125.24, 125.20, 125.16, 124.77, 119.91, 116.69, 107.11, 93.17, 84.35, 21.62, 21.55. ^{19}F NMR (376 MHz, $CDCl_3$) δ -62.82. HRMS (APCI) calc. for $C_{31}H_{23}F_3NO_2S^+$ [M+H]$^+$: 530.1396, found 530.1399.

3-(Hept-1-yn-1-yl)-2-(*p*-tolyl)-1-tosyl-1*H*-indole (**3ak**)

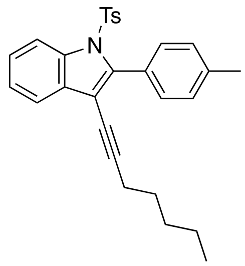

Following general procedure **GP4** with 4-methyl-*N*-(2-(*p*-tolylethynyl)phenyl)benzenesulfonamide **1a** (108 mg, 0.3 mmol) and 1-iodohept-1-yne **2k** (73 mg, 0.33 mmol). The crude product was purified by flash column chromatography (Petroleum ether : EtOAc = 20 : 1) to afford **3ak** as a yellow solid (52 mg, 38 %). Mp 125 °C. ^{1}H NMR (400 MHz, $CDCl_3$) δ 8.33 (d, *J* = 8.2 Hz, 1H), 7.96 (d, *J* = 8.4 Hz, 2H), 7.68 - 7.63 (m, 1H), 7.58 - 7.52 (m, 2H), 7.45 - 7.40 (m, 3H), 7.37 (dd, *J* = 7.5, 1.1 Hz, 1H), 7.33 - 7.27 (m, 4H), 7.08 - 7.01 (m, 2H), 3.91 (s, 3H), 2.49 (s, 3H), 2.29 (s, 3H). ^{13}C NMR (101 MHz, $CDCl_3$) δ 166.46, 144.90, 144.50, 139.35, 137.08, 134.51, 131.23, 131.10, 130.58, 129.44, 129.33, 128.10, 127.88, 127.59, 126.84, 125.73, 124.75, 119.93, 116.69, 107.28, 93.92, 84.92, 52.19, 21.61, 21.53. HRMS (APCI) calc. for $C29H30NO_2S^+$ $[M+H]^+$: 456.1992, found 456.1992.

2-(4-Fluorophenyl)-3-(phenylethynyl)-1-tosyl-1*H*-indole (**3bb**)

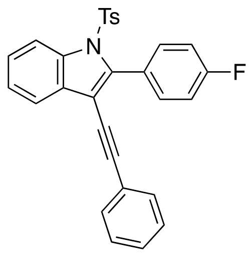

Following general procedure **GP4** with *N*-(2-((4-fluorophenyl)ethynyl)phenyl)-4-methylbenzenesulfonamide **1b** (109 mg, 0.3 mmol) and 1-iodohept-1-yne **2b** (75 mg, 0.33 mmol). The crude product was purified by flash column chromatography (Petroleum ether : EtOAc = 20 : 1) to afford **3bb** as a yellow solid (90 mg, 65 %). Mp 104 °C. ^{1}H NMR (400 MHz, $CDCl_3$) δ 8.35 (d, J = 8.3 Hz, 1H), 7.65 (ddd, J = 14.1, 8.4, 6.1 Hz, 3H), 7.47 - 7.41 (m, 1H), 7.40 - 7.35 (m, 3H), 7.30 (dd, J = 6.6, 3.2 Hz, 4H), 7.27 (s, 1H), 7.19 (t, J = 8.6 Hz, 2H), 7.07 (d, J = 8.1 Hz, 2H), 2.30 (s, 3H). ^{13}C NMR (101 MHz, $CDCl_3$) δ 164.48, 162.00, 145.02, 142.26, 137.11, 134.52, 133.19, 133.10, 131.40, 130.58, 129.39, 128.38, 128.34, 126.76, 126.72, 126.69, 125.89, 124.79, 122.92, 120.13, 116.62, 114.60, 114.38, 108.31, 94.89, 81.18, 21.53. ^{19}F NMR (376 MHz, $CDCl_3$) δ -111.53. HRMS (APCI) calc. for $C_{29}H_{21}FNO_2S^+$ $[M+H]^+$: 466.1272, found 466.1265.

2-(4-Fluorophenyl)-3-(*p*-tolylethynyl)-1-tosyl-1*H*-indole (**3bc**)

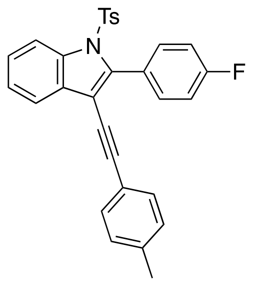

Following general procedure **GP4** with *N*-(2-((4-fluorophenyl)ethynyl)phenyl)-4-methylbenzenesulfonamide **1b** (109 mg, 0.3 mmol) and 1-(iodoethynyl)-4-methylbenzene **2c** (80 mg, 0.33 mmol). The crude product was purified by flash column chromatography (Petroleum ether : EtOAc = 20 : 1) to afford **3bc** as a yellow solid (98 mg, 68 %). Mp 110 °C. ^{1}H NMR (400 MHz, $CDCl_3$) δ 8.36 (d, *J* = 8.2 Hz, 1H), 7.71 - 7.62 (m, 3H), 7.48 - 7.42 (m, 1H), 7.39 (d, *J* = 7.5 Hz, 1H), 7.29 (d, *J* = 8.0 Hz, 4H), 7.20 (t, *J* = 8.7 Hz, 2H), 7.12 (d, *J* = 7.8 Hz, 2H), 7.07 (d, *J* = 8.0 Hz, 2H), 2.35 (s, 3H), 2.30 (s, 3H). ^{13}C NMR (101 MHz, $CDCl_3$) δ 164.41, 161.93, 144.96, 141.97, 138.59, 137.11, 134.44, 133.15, 133.06, 131.28, 130.66, 129.35, 129.08, 126.78, 126.75, 126.72, 125.83, 124.76, 120.14, 119.80, 116.61, 114.55, 114.33, 108.54, 95.12, 80.46, 21.49, 21.46. ^{19}F NMR (376 MHz, $CDCl_3$) δ -111.58. HRMS (APCI) calc. for $C_{30}H_{23}FNO_2S^+$ $[M+H]^+$: 480.1428, found 480.1422.

2-(2-Fluorophenyl)-3-(phenylethynyl)-1-tosyl-1*H*-indole (**3cb**)

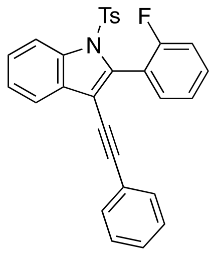

Following general procedure **GP4** with *N*-(2-((2-fluorophenyl)ethynyl)phenyl)-4-methylbenzenesulfonamide **1c** (109 mg, 0.3 mmol) and (iodoethynyl)benzene **2b** (75 mg, 0.33 mmol). The crude product was purified by flash column chromatography (Petroleum ether : EtOAc = 20 : 1) to afford **3cb** as a yellow solid (94 mg, 64 %). Mp 115 °C. ^{1}H NMR (400 MHz, $CDCl_3$) δ 8.29 (d, *J* = 8.3 Hz, 1H), 7.70 (d, *J* = 7.7 Hz, 1H), 7.57 - 7.49 (m, 2H), 7.41 (t, *J* = 8.3 Hz, 3H), 7.38 - 7.33 (m, 3H), 7.31 - 7.26 (m, 4H), 7.23 (t, J = 9.1 Hz, 1H), 7.10 (d, J = 8.1 Hz, 2H), 2.31 (s, 3H). ^{13}C NMR (101 MHz, $CDCl_3$) 162.01, 159.52, 144.99, 136.89, 136.76, 134.79, 132.91, 132.89, 131.46, 130.25, 129.51, 128.27, 126.82, 125.91, 124.45, 123.17, 123.00, 120.28, 115.93, 115.60, 115.39, 109.13, 95.43, 80.68, 21.55. ^{19}F NMR (376 MHz, $CDCl_3$) δ -109.70. HRMS (APCI) calc. for $C_{29}H_{21}FNO_2S^+$ $[M+H]^+$: 466.1272, found 466.1271.

3-((4-Fluorophenyl)ethynyl)-2-(4-methoxyphenyl)-1-tosyl-1*H*-indole (**3da**)

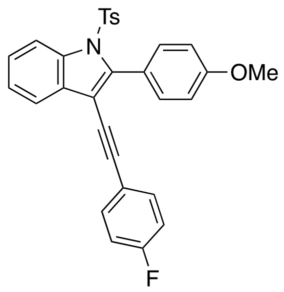

Following general procedure **GP4** with *N*-(2-((4-methoxyphenyl)ethynyl)phenyl)-4-methylbenzenesulfonamide **1d** (113 mg, 0.3 mmol) and 1-fluoro-4-(iodoethynyl)benzene **2a** (81 mg, 0.33 mmol). The crude product was purified by flash column chromatography (Petroleum ether : EtOAc = 15 : 1) to afford **3da** as a yellow solid (95 mg, 64 %). Mp 113 °C; ^{1}H NMR (400 MHz, $CDCl_3$) δ 8.37 (d, *J* = 8.3 Hz, 1H), 7.68 (d, *J* = 7.6 Hz, 1H), 7.45 (d, *J* = 8.3 Hz, 1H), 7.43 - 7.35 (m, 5H), 7.34 (s, 1H), 7.27 (s, 1H), 7.22 (d, *J* = 2.1 Hz, 1H), 7.09 (d, *J* = 8.1 Hz, 3H), 7.03 (d, *J* = 9.1 Hz, 2H), 3.90 (s, 3H), 2.32 (s, 3H). ^{13}C NMR (101 MHz, $CDCl_3$) δ 163.69, 161.20, 158.56, 144.88, 143.29, 137.16, 134.51, 133.34, 133.26, 131.80, 130.58, 129.31, 128.25, 126.88, 125.82, 124.70, 123.75, 120.05, 116.66, 116.53, 115.72, 115.50, 115.24, 108.00, 93.81, 81.16, 55.35, 21.52. ^{19}F NMR (376 MHz, $CDCl_3$) δ -110.59. HRMS (APCI) calc. for $C_{30}H_{23}FNO_3S^+$ $[M+H]^+$: 496.1377, found 496.1375.

2-(4-Methoxyphenyl)-3-(phenylethynyl)-1-tosyl-1*H*-indole (**3db**)

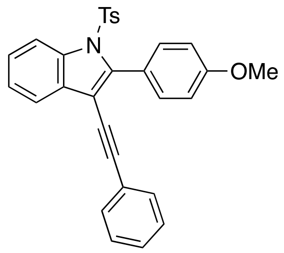

Following general procedure **GP4** with *N*-(2-((4-methoxyphenyl)ethynyl)phenyl)-4-methylbenzenesulfonamide **1d** (113 mg, 0.3 mmol) and (iodoethynyl)benzene **2b** (75 mg, 0.33 mmol). The crude product was purified by flash column chromatography (Petroleum ether : EtOAc = 20 : 1) to afford **3db** as a yellow solid (97 mg, 68 %). Mp 108 °C. ^{1}H NMR (300 MHz, $CDCl_3$) δ 8.33 (d, *J* = 7.6 Hz, 1H), 7.65 (d, *J* = 7.2 Hz, 1H), 7.60 (d, *J* = 8.8 Hz, 2H), 7.44 - 7.35 (m, 4H), 7.32 - 7.27 (m, 4H), 7.25 (s, 1H), 7.03 (t, *J* = 8.8 Hz, 4H), 3.92 (s, 3H), 2.29 (s, 3H). ^{13}C NMR (75 MHz, $CDCl_3$) δ 160.29, 144.79, 143.58, 137.10, 134.54, 132.68, 131.41, 130.89, 129.29, 128.28, 128.19, 126.84, 125.52, 124.69, 123.20, 122.96, 119.92, 116.75, 112.79, 107.53, 94.57, 81.71, 55.31, 21.54. HRMS (APCI) calc. for $C_{30}H_{24}NO_3S^+$ $[M+H]^+$: 478.1399, found 478.1397.

3-((4-Fluorophenyl)ethynyl)-2-(3-methoxyphenyl)-1-tosyl-1*H*-indole (**3ea**)

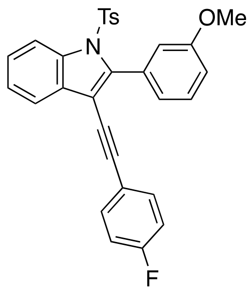

Following general procedure GP5 with N-(2-((3-methoxyphenyl)ethynyl)phenyl)-4-methylbenzenesulfonamide **1e** (113 mg, 0.3 mmol) and 1-fluoro-4-(iodoethynyl)benzene **2a** (81 mg, 0.33 mmol). The crude product was purified by flash column chromatography (Petroleum ether : EtOAc = 16 : 1) to afford **3ea** as a yellow solid (90 mg, 61 %). Mp 115 °C ^{1}H NMR (400 MHz, $CDCl_3$) δ 8.36 (d, *J* = 8.3 Hz, 1H), 7.67 - 7.64 (m, 1H), 7.63 - 7.57 (m, 2H), 7.46 - 7.41 (m, 1H), 7.41 - 7.36 (m, 3H), 7.31 - 7.28 (m, 2H), 7.11 - 6.97 (m, 6H), 3.94 (s, 3H), 2.32 (s, 3H). ^{13}C NMR (101 MHz, $CDCl_3$) δ 163.66, 161.17, 160.31, 144.82, 143.61, 137.07, 134.56, 133.31, 133.23, 132.66, 130.77, 129.30, 126.83, 125.56, 124.69, 122.92, 119.85, 119.31, 119.28, 116.73, 115.71, 115.49, 112.79, 107.31, 93.42, 81.41, 55.31, 21.53. ^{19}F NMR (376 MHz, $CDCl_3$) δ -110.78. HRMS (APCI) calc. for $C_{30}H_{23}FNO_3S^+$ $[M+H]^+$: 496.1377, found 496.1375.

2-(3-Methoxyphenyl)-3-(phenylethynyl)-1-tosyl-1*H*-indole (**3eb**)

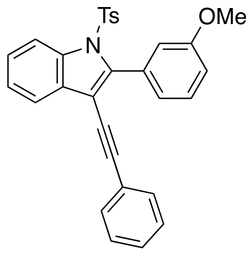

Following general procedure **GP4** with *N*-(2-((3-methoxyphenyl)ethynyl)phenyl)-4-methylbenzenesulfonamide **1e** (113 mg, 0.3 mmol) and (iodoethynyl)benzene **2b** (75 mg, 0.33 mmol). The crude product was purified by flash column chromatography (Petroleum ether : EtOAc = 15 : 1) to afford **3eb** as a yellow solid (105 mg, 67 %). Mp 112 °C. ^{1}H NMR (400 MHz, $CDCl_3$) δ 8.35 (d, *J* = 8.3 Hz, 1H), 7.68 (d, *J* = 7.4 Hz, 1H), 7.45 (d, *J* = 7.4 Hz, 1H), 7.43 – 7.34 (m, 5H), 7.33 (s, 1H), 7.30 (dd, *J* = 6.4, 3.1 Hz, 4H), 7.22 – 7.19 (m, 1H), 7.05 (t, *J* = 7.2 Hz, 3H), 3.87 (s, 3H), 2.30 (s, 3H). ^{13}C NMR (101 MHz, $CDCl_3$) δ 158.55, 144.86, 143.26, 137.20, 134.50, 131.82, 131.42, 130.70, 129.30, 128.28, 128.27, 128.24, 126.89, 125.78, 124.70, 123.80, 123.08, 120.12, 116.66, 116.45, 115.32, 108.23, 94.97, 81.44, 55.35, 21.52. HRMS (APCI) calc. for $C_{30}H_{24}NO_3S^+$ $[M+H]^+$: 478.1471, found 478.1472.

2-Phenyl-3-(phenylethynyl)-1-tosyl-1*H*-indole (**3fb**)

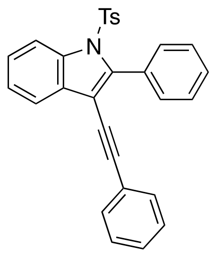

Following general procedure **GP4** with *N*-(2-((3-methoxyphenyl)ethynyl)phenyl)-4-methylbenzenesulfonamide **1f** (113 mg, 0.3 mmol) and (iodoethynyl)benzene **2b** (75 mg, 0.33 mmol). The crude product was purified by flash column chromatography (Petroleum ether : EtOAc = 15 : 1) to afford **3fb** as a yellow solid (62 mg, 46 %). Mp 105 °C. ^{1}H NMR (400 MHz, $CDCl_3$) δ 8.36 (d, *J* = 8.2 Hz, 1H), 7.72 - 7.65 (m, 3H), 7.53 - 7.48 (m, 3H), 7.44 (t, *J* = 7.2 Hz, 1H), 7.40 - 7.35 (m, 3H), 7.30 (d, *J* = 6.6 Hz, 5H), 7.06 (d, *J* = 8.2 Hz, 2H), 2.30 (s, 3H). ^{13}C NMR (101 MHz, $CDCl_3$) δ 144.85, 143.49, 137.14, 134.50, 131.39, 131.23, 130.72, 130.69, 129.31, 129.07, 128.26, 128.24, 127.26, 126.82, 125.74, 124.69, 123.07, 120.10, 116.63, 108.20, 94.70, 81.46, 21.52. HRMS (APCI) calc. for $C_{29}H_{22}NO_2S^+$ $[M+H]^+$: 448.1366, found 448.1365.

3-(Phenylethynyl)-2-(*m*-tolyl)-1-tosyl-1*H*-indole (**3gb**)

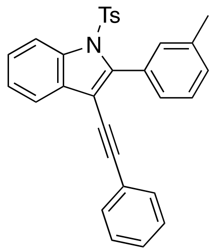

Following general procedure **GP4** with 4-methyl-N-(2-(m-tolylethynyl)phenyl)benzenesulfonamide **1g** (108 mg, 0.3 mmol) and (iodoethynyl)benzene **2b** (75 mg, 0.33 mmol). The crude product was purified by flash column chromatography (Petroleum ether : EtOAc = 15 : 1) to afford **3gb** as a yellow solid (86 mg, 62 %). Mp 110 °C. ^{1}H NMR (400 MHz, $CDCl_3$) δ 8.34 (d, *J* = 8.2 Hz, 1H), 7.67 (d, *J* = 7.3 Hz, 1H), 7.50 - 7.41 (m, 3H), 7.40 - 7.34 (m, 4H), 7.30 (ddd, *J* = 6.5, 4.0, 2.3 Hz, 6H), 7.06 (d, *J* = 8.2 Hz, 2H), 2.46 (s, 3H), 2.30 (s, 3H). ^{13}C NMR (101 MHz, $CDCl_3$) δ 144.80, 143.75, 137.15, 136.72, 134.58, 131.86, 131.41, 130.75, 130.58, 129.86, 129.28, 128.28, 128.21, 127.17, 126.89, 125.66, 124.65, 123.17, 120.07, 116.63, 108.01, 94.73, 81.61, 21.52, 21.47. HRMS (APCI) calc. for $C_{30}H_{24}NO_2S^+$ $[M+H]^+$: 462.1522, found 462.1520.

2-(4-Chlorophenyl)-3-(phenylethynyl)-1-tosyl-1*H*-indole (**3hb**)

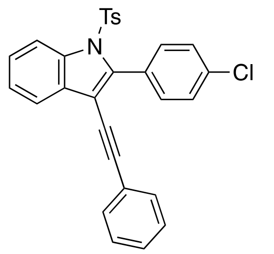

Following general procedure **GP4** with *N*-(2-((4-chlorophenyl)ethynyl)phenyl)-4-methylbenzenesulfonamide **1h** (114 mg, 0.3 mmol) and (iodoethynyl)benzene **2b** (75 mg, 0.33 mmol). The crude product was purified by flash column chromatography (Petroleum ether : EtOAc = 15 : 1) to afford **3hb** as a yellow solid (98 mg, 68 %). Mp 115 °C. ^{1}H NMR (400 MHz, $CDCl_3$) δ 8.35 (d, *J* = 8.3 Hz, 1H), 7.68 (d, *J* = 7.4 Hz, 1H), 7.63 (s, 2H), 7.49 (d, *J* = 8.5 Hz, 2H), 7.45 (d, *J* = 8.5 Hz, 1H), 7.43 - 7.37 (m, 3H), 7.32 (s, 3H), 7.29 (d, *J* = 8.4 Hz, 2H), 2.32 (s, 3H). ^{13}C NMR (101 MHz, $CDCl_3$) δ 145.06, 142.02, 137.25, 135.19, 134.36, 132.46, 131.45, 130.69, 129.42, 129.20, 128.46, 128.37, 127.65, 126.78, 126.03, 124.87, 122.85, 120.21, 116.71, 108.67, 95.18, 81.06, 21.55. HRMS (APCI) calc. for $C_{29}H_{21}ClNO_2S^+$ [M+H]$^+$: 482.0976, found 482.0979.

3-(Phenylethynyl)-1-tosyl-2-(4-(trifluoromethyl)phenyl)-1*H*-indole (**3ib**)

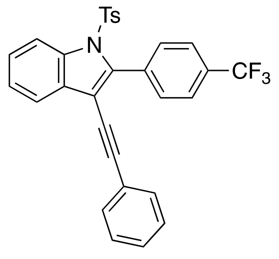

Following general procedure **GP4** with 4-methyl-*N*-(2-((4-(trifluoromethyl)phenyl)ethynyl)phenyl)benzenesulfonamide **1i** (124 mg, 0.3 mmol) and (iodoethynyl)benzene **2b** (75 mg, 0.33 mmol). The crude product was purified by flash column chromatography (Petroleum ether : EtOAc = 12 : 1) to afford **3ia** as a yellow solid (92 mg, 60 %). Mp 120 °C. ^{1}H NMR (400 MHz, $CDCl_3$) δ 8.33 (d, *J* = 8.3 Hz, 1H), 7.80 (d, *J* = 8.2 Hz, 2H), 7.75 (d, *J* = 8.3 Hz, 2H), 7.68 (d, *J* = 7.3 Hz, 1H), 7.46 (t, *J* = 8.5 Hz, 1H), 7.41 - 7.35 (m, 3H), 7.34 - 7.30 (m, 3H), 7.29 (s, 1H), 7.26 (s, 1H), 7.07 (d, *J* = 8.1 Hz, 2H), 2.30 (s, 3H). ^{13}C NMR (101 MHz, $CDCl_3$) δ 145.19, 141.42, 137.37, 134.41, 134.20, 131.46, 131.42, 130.65, 129.47, 128.60, 128.40, 126.76, 126.33, 124.98, 124.30, 124.26, 122.68, 120.39, 116.71, 109.51, 95.50, 80.77, 21.55. ^{19}F NMR (376 MHz, $CDCl_3$) δ -62.82. HRMS (APCI) calc. for $C_{30}H_{21}FlNO_2S^+$ [M+H]$^+$: 516.1240, found 516.1239.

3-((4-Fluorophenyl)ethynyl)-2-methyl-1-tosyl-1*H*-indole (**3ja**)

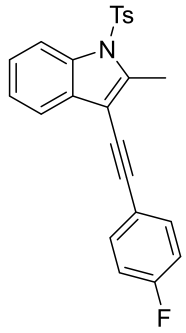

Following general procedure **GP4** with 4-methyl-N-(2-(prop-1-yn-1-yl)phenyl)benzenesulfonamide **(1j)** (86 mg, 0.3 mmol) and 1-fluoro-4-(iodoethynyl)benzene **2a** (81 mg, 0.33 mmol). The crude product was purified by flash column chromatography (Petroleum ether : EtOAc = 100 : 1) to afford 3**ja** as a white solid (71 mg, 59 %). Mp 105°C. ^{1}H NMR (400 MHz, $CDCl_3$) δ 8.19 (d, *J* = 7.4 Hz, 1H), 7.70 (d, *J* = 8.6 Hz, 2H), 7.61 (m, 1H), 7.53 - 7.50 (m, 2H), 7.35-7.28 (m, 2H), 7.22 (d, *J* = 8.2 Hz, 2H), 7.05 (m, 2H), 2.77 (s, 3H), 2.35 (s, 3H). ^{13}C NMR (101 MHz, $CDCl_3$) δ 163.89, 161.41, 145.31, 140.77, 136.11, 135.99, 133.52, 133.44, 130.16, 129.72, 126.63, 125.03, 124.06, 119.66, 119.57, 119.54, 115.96, 115.74, 114.66, 105.09, 94.63, 80.71, 21.73, 14.74. ^{19}F NMR (376 MHz, $CDCl_3$) δ -110.88. HRMS (ESI) calc. for $C_{24}H_{18}FNO_2S^+$ $[M+H]^+$: 404.1113, found 404.1115.

2-Pentyl-3-(phenylethynyl)-1-tosyl-1*H*-indole (**3kb**)

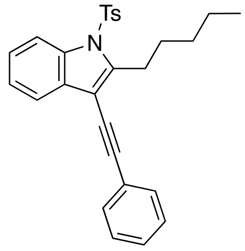

Following general procedure **GP4** with *N*-(2-(hept-1-yn-1-yl)phenyl)-4-methylbenzenesulfonamide **1k** (102 mg, 0.3 mmol) and (iodoethynyl)benzene **2b** (75 mg, 0.33 mmol). The crude product was purified by flash column chromatography (Petroleum ether : EtOAc = 18 : 1) to afford **3kb** as a yellow solid (77 mg, 58 %). Mp 128 ^{o}C. ^{1}H NMR (400 MHz, $CDCl_3$) δ 8.23 - 8.13 (m, 1H), 7.68 - 7.60 (m, 3H), 7.54 (dd, *J* = 7.2, 2.3 Hz, 2H), 7.39 - 7.29 (m, 5H), 7.19 (d, *J* = 8.0 Hz, 2H), 3.24 (t, *J* = 7.6 Hz, 2H), 2.33 (s, 3H), 1.85 (s, 2H), 1.43 (dt, *J* = 6.6, 3.6 Hz, 4H), 0.92 (t, *J* = 6.9 Hz, 3H). ^{13}C NMR (101 MHz, $CDCl_3$) δ 145.86, 144.92, 136.12, 135.88, 131.37, 129.89, 129.85, 128.38, 128.20, 126.32, 124.81, 123.99, 123.40, 119.56, 115.02, 105.57, 95.66, 81.04, 31.53, 30.13, 28.14, 22.35, 21.52, 14.01. HRMS (APCI) calc. for $C_{28}H_{28}NO_2S^+$ $[M+H]^+$: 442.1835, found 442.1834.

2-(4-Chlorobutyl)-3-(phenylethynyl)-1-tosyl-1*H*-indole (**3lb**)

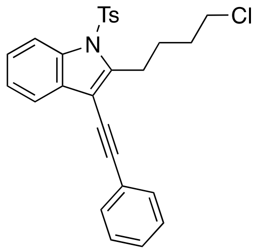

Following general procedure **GP4** with *N*-(2-(6-chlorohex-1-yn-1-yl)phenyl)-4-methylbenzenesulfonamide **1l** (138 mg, 0.3 mmol) and (iodoethynyl)benzene **2b** (75 mg, 0.33 mmol). The crude product was purified by flash column chromatography (Petroleum ether : EtOAc = 18 : 1) to afford **3lb** as a yellow solid (68 mg, 49 %). Mp 125 °C. ^{1}H NMR (400 MHz, $CDCl_3$) δ 8.18 (d, *J* = 7.2 Hz, 1H), 7.63 (d, *J* = 8.6 Hz, 3H), 7.55 (dd, *J* = 7.3, 2.4 Hz, 2H), 7.41 - 7.28 (m, 5H), 7.19 (d, *J* = 8.1 Hz, 2H), 3.61 (t, *J* = 6.6 Hz, 2H), 3.28 (t, *J* = 7.2 Hz, 2H), 2.34 (s, 3H), 2.02 (dt, *J* = 14.3, 6.4 Hz, 2H), 1.92 (dt, *J* = 13.5, 6.5 Hz, 2H). ^{13}C NMR (101 MHz, $CDCl_3$) δ 145.09, 144.50, 136.16, 135.70, 131.44, 129.77, 128.43, 128.37, 126.31, 126.20, 125.06, 124.11, 123.15, 119.69, 115.05, 106.15, 96.00, 80.72, 44.65, 32.02, 27.63, 27.33, 21.54. HRMS (APCI) calc. for $C_{27}H_{25}ClNO_2S^+$ $[M+H]^+$: 462.1289, found 462.1287.

5-Chloro-2-phenyl-3-(phenylethynyl)-1-tosyl-1*H*-indole (**3mb**)

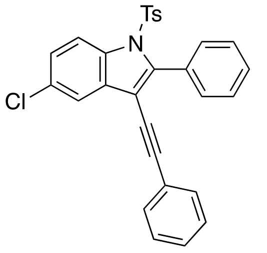

Following general procedure **GP4** with *N*-(4-chloro-2-(phenylethynyl)phenyl)-4-methylbenzenesulfonamide **1m** (114 mg, 0.3 mmol) and (iodoethynyl)benzene **2b** (75 mg, 0.33 mmol). The crude product was purified by flash column chromatography (Petroleum ether : EtOAc = 12 : 1) to afford **3mb** as a brown solid (60 mg, 42 %). Mp 102 °C. ^{1}H NMR (400 MHz, $CDCl_3$) δ 8.28 (d, *J* = 8.8 Hz, 1H), 7.65 (dd, *J* = 4.2, 1.8 Hz, 3H), 7.50 (dd, *J* = 5.1, 1.9 Hz, 3H), 7.41 - 7.35 (m, 3H), 7.32 - 7.25 (m, 5H), 7.08 (d, *J* = 8.1 Hz, 2H), 2.32 (s, 3H). ^{13}C NMR (101 MHz, $CDCl_3$) δ 145.18, 144.65, 135.46, 134.30, 131.98, 131.43, 131.25, 130.62, 130.21, 129.45, 129.38, 128.43, 128.30, 127.33, 126.83, 125.91, 122.79, 119.78, 117.74, 107.45, 95.05, 80.68, 21.55. HRMS (APCI) calc. for $C_{29}H_{21}ClNO_2S^+$ $[M+H]^+$: 482.0976, found 482.0955.

5-Methyl-2-phenyl-3-(phenylethynyl)-1-tosyl-1*H*-indole (**3nb**)

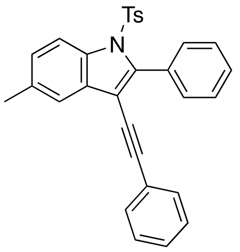

Following general procedure **GP4** with 4-methyl-*N*-(4-methyl-2-(phenylethynyl)phenyl)benzenesulfonamide **1n** (108 mg, 0.3 mmol) and (iodoethynyl)benzene **2b** (75 mg, 0.33 mmol). The crude product was purified by flash column chromatography (Petroleum ether : EtOAc = 12 : 1) to afford **3nb** as a brown solid (64 mg, 46 %). Mp 106 °C. ^{1}H NMR (400 MHz, $CDCl_3$) δ 8.21 (d, *J* = 8.5 Hz, 1H), 7.66 (dd, *J* = 6.6, 2.9 Hz, 2H), 7.49 (t, *J* = 2.7 Hz, 3H), 7.45 (s, 1H), 7.41 – 7.36 (m, 2H), 7.29 (dd, *J* = 7.5, 3.2 Hz, 5H), 7.26 – 7.22 (m, 1H), 7.05 (d, *J* = 8.2 Hz, 2H), 2.48 (s, 3H), 2.29 (s, 3H). ^{13}C NMR (101 MHz, $CDCl_3$) δ 144.73, 143.61, 135.36, 134.54, 134.49, 131.41, 131.21, 130.94, 130.80, 129.28, 129.01, 128.26, 128.21, 127.23, 127.13, 126.83, 123.14, 119.97, 116.36, 108.06, 94.59, 81.64, 21.52, 21.29. HRMS (APCI) calc. for $C_{30}H_{24}NO_2S^+$ $[M+H]^+$: 462.1449, found 462.1446.

3-((4-Luorophenyl)ethynyl)-1-(methylsulfonyl)-2-(*p*-tolyl)-1*H*-indole (**3Ms-aa**).

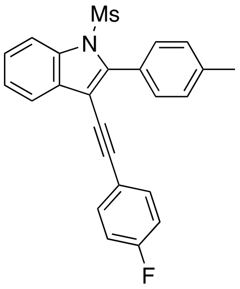

Following general procedure **GP4** with *N*-(2-(*p*-tolylethynyl)phenyl)methanesulfonamide **1Ms-a** (86 mg, 0.3 mmol) and 1-fluoro-4-(iodoethynyl)benzene **2a** (81 mg, 0.33 mmol). The crude product was purified by flash column chromatography (Petroleum ether : EtOAc = 18 : 1) to afford **3Ms-aa** as a yellow solid (39 mg, 32 %). Mp 128 °C. ^{1}H NMR (400 MHz, $CDCl_3$) δ 8.17 - 8.12 (m, 1H), 7.81 - 7.76 (m, 1H), 7.61 (d, *J* = 7.9 Hz, 2H), 7.48 - 7.38 (m, 4H), 7.30 (d, *J* = 7.8 Hz, 2H), 7.02 (t, *J* = 8.7 Hz, 2H), 2.77 (s, 3H), 2.45 (s, 3H). ^{13}C NMR (101 MHz, $CDCl_3$) δ 163.76, 161.28, 143.48, 139.43, 136.89, 133.45, 133.36, 130.81, 130.57, 128.35, 127.31, 126.00, 124.95, 120.32, 115.94, 115.76, 115.54, 107.46, 93.77, 80.98, 39.77, 21.57. ^{19}F NMR (376 MHz, $CDCl_3$)) δ -110.59. HRMS (APCI) calc. for $C_{24}H_{19}FNO_2S^+$ $[M+H]^+$: 404.1115, found 404.1116.

1-(methylsulfonyl)-3-(phenylethynyl)-2-(*p*-tolyl)-1*H*-indole (**3Ms-ab**)

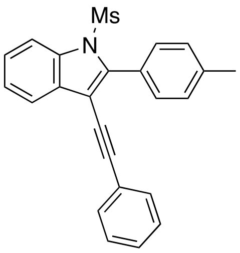

Following general procedure **GP4** with *N*-(2-(*p*-tolylethynyl)phenyl)methanesulfonamide **1Ms-a** (86 mg, 0.3 mmol) and (iodoethynyl)benzene **2b** (75 mg, 0.33 mmol). The crude product was purified by flash column chromatography (Petroleum ether : EtOAc = 18 : 1) to afford **3Ms-ab** as a yellow solid (43 mg, 37 %). Mp 123 °C. ^{1}H NMR (400 MHz, $CDCl_3$) δ 8.19 - 8.14 (m, 1H), 7.83 (dd, *J* = 6.3, 2.8 Hz, 1H), 7.65 (d, *J* = 8.0 Hz, 2H), 7.49 - 7.44 (m, 4H), 7.37 - 7.30 (m, 5H), 2.79 (s, 3H), 2.47 (s, 3H). ^{13}C NMR (101 MHz, $CDCl_3$) δ 143.45, 139.37, 136.92, 131.52, 130.83, 130.68, 128.68, 128.33, 128.32, 127.33, 125.96, 124.94, 123.13, 120.38, 115.94, 107.68, 94.92, 81.25, 39.69, 21.56. HRMS (APCI) calc. for $C_{24}H_{20}NO_2S^+$ [M+H]$^+$: 386.1209, found 386.1210.

3-((4-Fluorophenyl)ethynyl)-1-((4-nitrophenyl)sulfonyl)-2-(p-tolyl)-1H-indole (**3Ns-aa**)

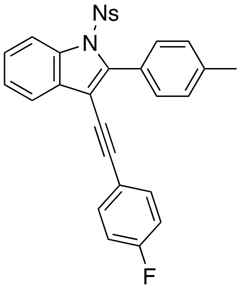

Following general procedure **GP4** with 4-nitro-*N*-(2-(*p*-tolylethynyl)phenyl)benzenesulfonamide **1Ns-a** (117 mg, 0.3 mmol) and 1-fluoro-4-(iodoethynyl)benzene **2a** (81 mg, 0.33 mmol). The crude product was purified by flash column chromatography (Petroleum ether : DCM = 1 : 1) to afford **3Ns-aa** as a yellow solid (64 mg, 42 %). Mp 145 °C. ^{1}H NMR (400 MHz, $CDCl_3$) δ 8.30 (d, *J* = 8.3 Hz, 1H), 8.10 (d, *J* = 8.7 Hz, 2H), 7.66 - 7.61 (m, 1H), 7.60 - 7.52 (m, 4H), 7.46 (t, *J* = 7.7 Hz, 1H), 7.42 - 7.30 (m, 5H), 7.00 (t, *J* = 8.6 Hz, 2H), 2.49 (s, 3H). ^{13}C NMR (101 MHz, $CDCl_3$) δ 163.85, 161.36, 150.56, 143.19, 142.11, 139.76, 136.91, 133.46, 133.38, 131.07, 130.96, 128.36, 128.15, 127.21, 126.27, 125.55, 123.88, 120.44, 118.94, 118.90, 116.77, 115.80, 115.58, 108.98, 94.39, 80.64, 21.62. ^{19}F NMR (376 MHz, $CDCl_3$) δ -110.20. HRMS (APCI) calc. for $C_{29}H_{20}FNO_4S^+$ [M+H]$^+$: 511.1122.

3-((4-Fluorophenyl)ethynyl)-2-(*p*-tolyl)-1-((2-(trimethylsilyl)ethyl)sulfonyl)-1*H*-indole (**3SES-aa**)

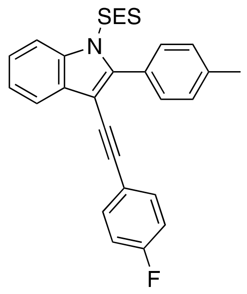

Following general procedure GP5 with *N*-(2-(*p*-tolylethynyl)phenyl)-2-(trimethylsilyl)ethane-1-sulfonamide**1SES-a** (111 mg, 0.3 mmol) and 1-fluoro-4-(iodoethynyl)benzene **2a** (81 mg, 0.33 mmol). The crude product was purified by flash column chromatography (Petroleum ether : DCM = 1 : 1) to afford **3SES-aa** as a yellow solid (66 mg, 45 %). Mp 155 °C. ^{1}H NMR (400 MHz, $CDCl_3$) δ 8.18 - 8.13 (m, 1H), 7.81 - 7.75 (m, 1H), 7.58 (d, *J* = 7.9 Hz, 2H), 7.45 - 7.38 (m, 4H), 7.29 (d, *J* = 7.9 Hz, 2H), 7.01 (t, *J* = 8.6 Hz, 2H), 2.96 - 2.88 (m, 2H), 2.45 (s, 3H), 0.69 - 0.61 (m, 2H), -0.12 (s, 9H). ^{13}C NMR (101 MHz, $CDCl_3$) δ 163.72, 161.23, 143.88, 139.35, 137.20, 133.38, 133.30, 131.07, 129.87, 128.27, 127.42, 125.71, 124.41, 120.18, 119.44, 119.40, 115.71, 115.58, 115.50, 106.27, 93.37, 81.26, 50.83, 21.54, 9.56, -2.24. ^{19}F NMR (376 MHz, $CDCl_3$) δ -110.85. HRMS (APCI) calc. for $C_{28}H_{29}FNO_2SSi^+$ $[M+H]^+$: 490.1667, found 490.1655.

3-((4-Fluorophenyl)ethynyl)-2-(p-tolyl)-1H-indole (**3aa-H**)

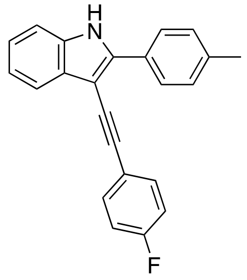

To a stirred solution of 3-((4-fluorophenyl)ethynyl)-2-(*p*-tolyl)-1-((2-(trimethylsilyl)ethyl)sulfonyl)-1*H*-indole **3SES-aa** (48.9 mg, 0.1 mmol, 1 equiv.) in THF (4.0 mL) was added TBAF (0.4 mmol, 4 equiv.). The mixture was stirred at room temperature for 3 h. The reaction mixture was poured into water and then extracted with EtOAc (3 × 10 mL). The combined organic phase was washed with saturated brine (10 mL), dried over $MgSO_4$ and filtered. The solvent was removed under reduced pressure and the crude product was purified by flash chromatography to give **3aa-H** (31 mg, 95 %) as a yellow solid. Mp 119 °C.^{1}H NMR (400 MHz, $CDCl_3$) δ 8.32 (s, 1H), 7.92 (d, *J* = 6.1 Hz, 2H), 7.82 (d, *J* = 6.6 Hz, 1H), 7.55 (s, 2H), 7.39 (d, *J* = 6.8 Hz, 1H), 7.32 (d, *J* = 6.6 Hz, 2H), 7.27 - 7.21 (m, 2H), 7.07 (t, *J* = 7.4 Hz, 2H), 2.43 (s, 3H). ^{13}C NMR (101 MHz, $CDCl_3$) δ 163.32, 160.85, 139.84, 138.55, 135.21, 133.02, 132.94, 130.28, 129.62, 128.72, 126.44, 123.36, 120.93, 120.53, 120.49, 119.95, 115.66, 115.44, 110.95, 95.29, 92.29, 83.87, 21.35. HRMS (APCI) calc. for $C_{23}H_{17}FN^+$ $[M+H]^+$: 326.1340, found 326.1340.

Methyl benzyl((2-(*p*-tolyl)-1-tosyl-1*H*-indol-3-yl)ethynyl)carbamate (**8a**)

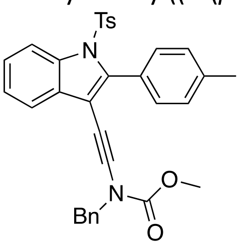

Following general procedure **GP4** with 4-methyl-*N*-(2-(*p*-tolylethynyl)phenyl)benzenesulfonamide **1a** (108 mg, 0.3 mmol) and methyl benzyl(iodoethynyl)carbamate **7** (104 mg, 0.33 mmol). The crude product was purified by flash column chromatography (Petroleum ether : EtOAc = 10 : 1) to afford **8a** as a yellow solid (92 mg, 56 %). Mp 129 °C. ^{1}H NMR (400 MHz, $CDCl_3$) δ 8.28 (d, *J* = 8.3 Hz, 1H), 7.45 (d, *J* = 7.8 Hz, 2H), 7.36 (t, *J* = 8.4 Hz, 1H), 7.32 - 7.22 (m, 11H), 7.03 (d, *J* = 8.2 Hz, 2H), 4.60 (s, 2H), 3.83 (s, 3H), 2.46 (s, 3H), 2.29 (s, 3H). ^{13}C NMR (101 MHz, $CDCl_3$) δ 155.50, 144.66, 142.15, 138.73, 137.02, 135.70, 134.44, 131.04, 130.96, 130.94, 129.24, 129.15, 128.51, 128.02, 127.98, 127.96, 126.82, 126.42, 125.44, 124.49, 119.87, 116.56, 107.72, 54.15, 53.88, 21.58, 21.50. HRMS (APCI) calc. for $C_{33}H_{29}N_2O_4S^+$ $[M+H]^+$: 549.1844, found 549.1843.

Methyl benzyl((2-(4-methoxyphenyl)-1-tosyl-1*H*-indol-3-yl)ethynyl)carbamate (**8b**)

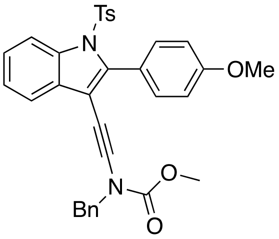

Following general procedure **GP4** with *N*-(2-((4-methoxyphenyl)ethynyl)phenyl)-4-methylbenzenesulfonamide **1d** (108 mg, 0.3 mmol) and methyl benzyl(iodoethynyl)carbamate **7** (104 mg, 0.33 mmol). The crude product was purified by flash column chromatography (Petroleum ether : EtOAc = 10 : 1) to afford **8b** as a yellow solid (101 mg, 60 %). Mp 135 °C. ^{1}H NMR (300 MHz, $CDCl_3$) δ 8.28 (d, *J* = 8.3 Hz, 1H), 7.47 (d, *J* = 8.7 Hz, 2H), 7.40 - 7.28 (m, 6H), 7.26 - 7.20 (m, 4H), 7.03 (d, *J* = 7.7 Hz, 2H), 6.94 (d, *J* = 8.8 Hz, 2H), 4.60 (s, 2H), 3.90 (s, 3H), 3.83 (s, 3H), 2.28 (s, 3H). ^{13}C NMR (75 MHz, $CDCl_3$) δ 160.06, 155.50, 144.66, 142.06, 136.97, 135.71, 134.52, 132.51, 129.38, 129.23, 128.67, 128.51, 128.15, 128.03, 127.92, 126.80, 125.34, 124.49, 123.09, 119.78, 116.58, 112.70, 107.30, 55.24, 54.16, 53.91, 21.50. HRMS (APCI) calc. for $C_{33}H_{29}N_2O_5S^+$ $[M+H]^+$: 565.1792, found 565.1791.

Methyl benzyl((2-(3-methoxyphenyl)-1-tosyl-1*H*-indol-3-yl)ethynyl)carbamate (**8c**)

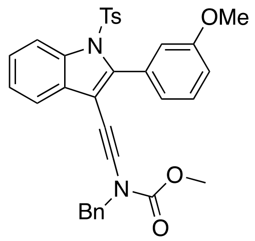

Following general procedure **GP4** with *N*-(2-((3-methoxyphenyl)ethynyl)phenyl)-4-methylbenzenesulfonamide **1e** (113 mg, 0.3 mmol) and methyl benzyl(iodoethynyl)carbamate **7** (104 mg, 0.33 mmol). The crude product was purified by flash column chromatography (Petroleum ether : EtOAc = 8 : 1) to afford **8c** as a yellow solid (93 mg, 55 %). Mp 126 °C. ^{1}H NMR (300 MHz, $CDCl_3$) δ 8.31 (d, *J* = 8.3 Hz, 1H), 7.44 - 7.35 (m, 2H), 7.35 - 7.27 (m, 9H), 7.19 - 7.10 (m, 2H), 7.10 - 7.00 (m, 3H), 4.62 (s, 2H), 3.86 (s, 3H), 3.85 (s, 3H), 2.31 (s, 3H). ^{13}C NMR (75 MHz, $CDCl_3$) δ 158.50, 155.46, 144.73, 141.47, 137.09, 135.65, 134.44, 132.05, 130.84, 129.43, 129.25, 128.53, 128.37, 128.15, 128.05, 127.94, 126.87, 125.62, 124.51, 123.67, 119.98, 116.59, 116.53, 114.74, 108.10, 55.23, 54.16, 53.91, 21.50. HRMS (APCI) calc. for $C_{33}H_{29}N_2O_5S^+$ [M+H]$^+$: 565.1792, found 565.1790.

Methyl benzyl((2-(4-chlorophenyl)-1-tosyl-1*H*-indol-3-yl)ethynyl)carbamate (**8d**)

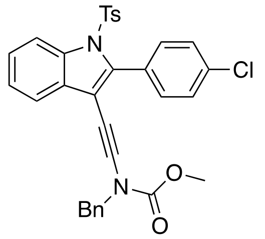

Following general procedure **GP4** with *N*-(2-((4-chlorophenyl)ethynyl)phenyl)-4-methylbenzenesulfonamide **1h** (114 mg, 0.3 mmol) and methyl benzyl(iodoethynyl)carbamate **7** (104 mg, 0.33 mmol). The crude product was purified by flash column chromatography (Petroleum ether : EtOAc = 8 : 1) to afford **8d** as a yellow solid (75 mg, 44 %). Mp 125 °C. ^{1}H NMR (400 MHz, $CDCl_3$) δ 8.27 (d, J = 8.4 Hz, 1H), 7.45 (d, J = 8.3 Hz, 2H), 7.41 - 7.35 (m, 3H), 7.34 - 7.27 (m, 5H), 7.25 - 7.18 (m, 4H), 7.03 (d, J = 8.1 Hz, 2H), 4.60 (s, 2H), 3.83 (s, 3H), 2.28 (s, 3H). ^{13}C NMR (101 MHz, $CDCl_3$) δ 155.43, 144.93, 140.50, 137.12, 135.54, 134.82, 134.30, 132.28, 130.83, 129.35, 129.29, 128.58, 128.41, 128.38, 128.27, 128.14, 127.55, 126.75, 125.85, 124.67, 120.06, 116.56, 108.50, 54.26, 53.84, 21.52. HRMS (APCI) calc. for $C_{32}H_{26}ClN_2O_4S^+$ [M+H]$^+$: 569.1296, found 569.1298.

Methyl benzyl((1-tosyl-2-(4-(trifluoromethyl)phenyl)-1*H*-indol-3-yl)ethynyl)carbamate (**8e**)

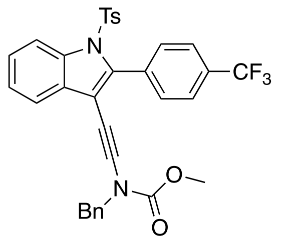

Following general procedure **GP4** with 4-methyl-*N*-(2-((4-(trifluoromethyl)phenyl)ethynyl)phenyl)benzenesulfonamide **1i** (114 mg, 0.3 mmol) and methyl benzyl(iodoethynyl)carbamate **7** (104 mg, 0.33 mmol). The crude product was purified by flash column chromatography (Petroleum ether : EtOAc = 18 : 1) to afford **8e** as a yellow solid (52 mg, 42 %). Mp 130 ^{o}C. ^{1}H NMR (400 MHz, $CDCl_3$) δ 8.28 (d, J = 8.4 Hz, 1H), 7.65 (s, 4H), 7.32 (dt, J = 5.5, 2.8 Hz, 4H), 7.30 – 7.21 (m, 6H), 7.05 (d, J = 8.1 Hz, 2H), 4.62 (s, 2H), 3.83 (s, 3H), 2.30 (s, 3H). ^{13}C NMR (75 MHz, $CDCl_3$) δ 155.38, 145.05, 137.31, 135.46, 134.50, 134.08, 131.21, 130.82, 130.56, 130.13, 129.52, 129.40, 128.59, 128.34, 128.18, 126.74, 126.17, 125.94, 124.81, 124.18, 122.33, 120.24, 116.63, 109.45, 54.26, 53.81, 21.53. ^{19}F NMR (376 MHz, $CDCl_3$) δ -62.47. HRMS (APCI) calc. for $C_{33}H_{26}F_3N_2O_4S^+$ $[M+H]^+$: 603.1560, found 603.1551.

Methyl benzyl((5-chloro-2-phenyl-1-tosyl-1*H*-indol-3-yl)ethynyl)carbamate (**8f**)

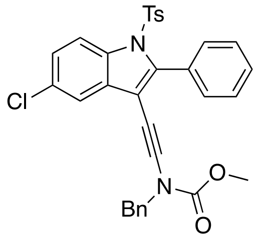

Following general procedure **GP4** with *N*-(4-chloro-2-(phenylethynyl)phenyl)-4-methylbenzenesulfonamide **1l** (114 mg, 0.3 mmol) and methyl benzyl(iodoethynyl)carbamate **7** (104 mg, 0.33 mmol). The crude product was purified by flash column chromatography (Petroleum ether : EtOAc = 18 : 1) to afford **8f** as a yellow solid (73 mg, 43 %). Mp 124 ^{o}C. ^{1}H NMR (400 MHz, $CDCl_3$) δ 8.20 (d, J = 8.9 Hz, 1H), 7.52 (d, J = 6.4 Hz, 2H), 7.45 (dd, J = 11.5, 7.1 Hz, 3H), 7.36 – 7.30 (m, 4H), 7.26 – 7.19 (m, 5H), 7.05 (d, J = 8.1 Hz, 2H), 4.59 (s, 2H), 3.83 (s, 3H), 2.31 (s, 3H). ^{13}C NMR (101 MHz, $CDCl_3$) δ 155.38, 145.04, 135.42, 135.37, 134.22, 132.21, 131.11, 130.44, 130.44, 130.36, 130.36, 129.39, 129.39, 129.09, 128.63, 128.59, 128.31, 127.25, 126.81, 125.75, 119.65, 117.63, 107.34, 54.22, 53.69, 21.54. HRMS (APCI) calc. for $C_{32}H_{26}ClN_2O_4S^+$ $[M+H]^+$: 569.1296, found 569.1300.

Methyl benzyl((2-(*p*-tolyl)-1-tosyl-1*H*-benzo[*f*]indol-3-yl)ethynyl)carbamate (**8g**)

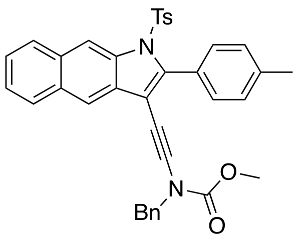

Following general procedure **GP4** with 4-Methyl-*N*-(3-(*p*-tolylethynyl)naphthalen-2-yl)benzenesulfonamide **1o** (123 mg, 0.3 mmol) and methyl benzyl(iodoethynyl)carbamate **7** (104 mg, 0.33 mmol). The crude product was purified by flash column chromatography (Petroleum ether : EtOAc = 18 : 1) to afford **8g** as a yellow solid (86 mg, 48 %). Mp 135 °C. ^{1}H NMR (400 MHz, $CDCl_3$) δ 8.72 (s, 1H), 8.07 - 8.02 (m, 1H), 7.88 -7.83 (m, 1H), 7.63 (s, 1H), 7.56 (d, *J* = 7.8 Hz, 2H), 7.52 - 7.46 (m, 2H), 7.39 - 7.30 (m, 5H), 7.29 - 7.22 (m, 5H), 6.97 (d, *J* = 8.1 Hz, 2H), 4.66 (s, 2H), 3.88 (s, 3H), 2.48 (s, 3H), 2.23 (s, 3H). ^{13}C NMR (101 MHz, $CDCl_3$) δ 155.53, 144.62, 139.08, 136.44, 135.66, 133.85, 131.94, 131.21, 131.18, 130.79, 129.24, 128.80, 128.71, 128.65, 128.60, 128.30, 128.18, 128.18, 128.08, 128.05, 127.76, 126.93, 125.22, 125.19, 117.80, 114.49, 108.01, 54.25, 53.82, 21.63, 21.47. HRMS (APCI) calc. for $C_{37}H_{31}N_2O_4S^+$ [M+H]$^+$: 599.1999, found 599.2008.

f. Post-functionalization

3-Benzyl-4-iodo-5-(2-(*p*-tolyl)-1-tosyl-1*H*-indol-3-yl)oxazol-2(3*H*)-one (**9**)

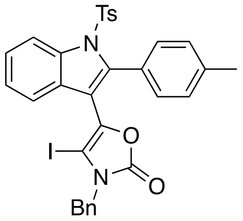

A mixture of **8a** (55 mg,0.1 mmol), I_2 (28 mg, 0.11 mmol) and DCE (2 mL) was stirred at room temperature for 8 h. The mixture was concentrated *in vacuo*, added to water, and extracted with EtOAc (3 × 10 mL). Organic layers were combined, dried over $MgSO_4$, filtered, concentrated, and purified by flash column chromatography to afford product **9** (65 mg, 98 %) as a yellow oil. ^{1}H NMR (400 MHz, $CDCl_3$) δ 8.39 (d, *J* = 8.4 Hz, 1H), 7.44 (dd, *J* = 8.3, 5.8 Hz, 3H), 7.38 - 7.30 (m, 5H), 7.28 (dd, *J* = 8.5, 4.8 Hz, 6H), 7.18 (d, *J* = 8.0 Hz, 4H), 7.09 (d, *J* = 7.9 Hz, 2H), 4.77 (s, 2H), 2.45 (s, 3H), 2.34 (s, 3H). ^{13}C NMR (101 MHz, $CDCl_3$) δ 154.63, 145.09, 142.59, 139.32, 136.88, 135.88, 135.35, 134.88, 131.44, 129.44, 129.20, 128.66, 128.26, 127.90, 127.36, 126.96, 126.81, 125.56, 124.57, 119.97, 116.35, 110.63, 77.32, 77.00, 76.68, 71.02, 48.16, 21.56. HRMS (APCI) calc. for $C_{32}H_{26}IN_2O_4S^+$ [M+H]$^+$: 661.0652, found 661.0653.

3-Benzyl-5-(2-(*p*-tolyl)-1-tosyl-1*H*-indol-3-yl)oxazol-2(3*H*)-one (**10**)

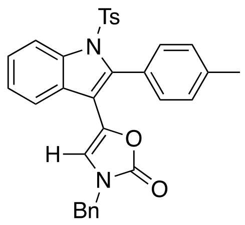

A mixture of **8a** (55 mg, 0.1 mmol), $AgSbF_6$ (6.9 mg, 0.02 mmol) and DCE (2 mL) was stirred at 85 °C for 12 h. The mixture was concentrated *in vacuo*, added to water, and extracted with EtOAc (3 × 10 mL). Organic layers were combined, dried over $MgSO_4$, filtered, concentrated, and purified by flash column chromatography to afford product **10** (47 mg, 88 %) as a yellow oil. ^{1}H NMR (400 MHz, $CDCl_3$) δ 8.36 (d, *J* = 8.4 Hz, 1H), 8.00 (d, *J* = 7.9 Hz, 1H), 7.39 (d, *J* = 8.4 Hz, 4H), 7.35 - 7.32 (m, 3H), 7.20 - 7.09 (m, 8H), 5.42 (s, 1H), 4.59 (s, 2H), 2.45 (s, 3H), 2.35 (s, 3H). ^{13}C NMR (75 MHz, $CDCl_3$) δ 154.57, 145.00, 139.43, 136.45, 136.21, 135.61, 135.04, 134.29, 130.84, 129.51, 128.83, 128.80, 128.19, 127.86, 127.62, 127.07, 126.89, 125.52, 124.35, 121.28, 115.38, 111.16, 111.01, 47.58, 21.57, 21.56. HRMS (APCI) calc. for $C_{32}H_{27}N_2O_4S^+$ [M+H]$^+$: 535.1613, found 535.1616.

Methyl benzyl(2-methoxy-11-tosyl-11*H*-benzo[*a*]carbazol-5-yl)carbamate (**11**)

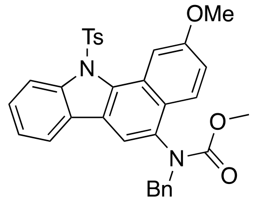

A mixture of **8c** (56 mg,0.1 mmol), $Ph_3PAuNTf_2$ (7.4 mg, 0.01 mmol) and DCE (2 mL) was stirred at 50 °C for 10 h. The mixture was concentrated in vacuo, added to water, and extracted with EtOAc (3 × 10 mL). Organic layers were combined, dried over $MgSO_4$, filtered, concentrated, and purified by flash column chromatography to afford product **11** (41 mg, 85 %) as a yellow solid. Mp 150 °C. ^{1}H NMR (400 MHz, $CDCl_3$) δ 8.56 (d, J = 8.7 Hz, 1H), 8.27 (d, J = 8.3 Hz, 1H), 7.59 (s, 1H), 7.44 - 7.37 (m, 3H), 7.24 (td, J = 9.3, 8.2, 4.6 Hz, 7H), 7.06 (s, 1H), 6.99 (d, J = 7.8 Hz, 1H), 6.75 (q, J = 8.3 Hz, 5H), 5.43 (d, J = 14.8 Hz, 1H), 4.14 (d, J = 14.8 Hz, 1H), 3.97 (s, 3H), 3.60 (s, 3H), 2.16 (s, 3H). ^{13}C NMR (101 MHz, $CDCl_3$) δ 157.18, 155.76, 144.18, 142.32, 137.98, 136.54, 136.15, 130.95, 129.76, 129.22, 129.03, 128.22, 128.12, 127.72, 127.24, 126.97, 126.08, 125.75, 121.55, 120.36, 120.09, 119.49, 119.33, 106.28, 56.03, 55.27, 52.75, 21.40. HRMS (APCI) calc. for $C_{33}H_{29}N_2O_5S^+$ [M+H]$^+$: 549.1792, found 549.1791.

Methyl benzyl(3-methyl-11-tosyl-11H-benzo[a]carbazol-5-yl)carbamate (**12**)

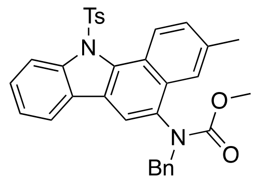

A mixture of **8a** (55 mg, 0.1 mmol), $RhCl(Ph_3P)_3$ (18.5 mg, 0.02 mmol), $AgSbF_6$ (6.9 mg, 0.02 mmol) and PhMe (2 mL) was stirred at 100 ^oC for 10 h. The mixture was concentrated *in vacuo*, added to water, and extracted with EtOAc (3 × 10 mL). Organic layers were combined, dried over $MgSO_4$, filtered, concentrated, and purified by flash column chromatography to afford product **12** (43 mg, 78 %) as a yellow solid. Mp 145 ^oC. ^1H NMR (400 MHz, $CDCl_3$) δ 8.91 (d, *J* = 8.7 Hz, 1H), 8.29 (d, *J* = 8.2 Hz, 1H), 7.57 (s, 1H), 7.52 (dd, *J* = 8.7, 1.7 Hz, 1H), 7.41 (t, *J* = 7.9 Hz, 2H), 7.29 (d, *J* = 7.6 Hz, 1H), 7.25 - 7.12 (m, 6H), 6.81 (d, *J* = 8.1 Hz, 2H), 6.74 (d, *J* = 8.1 Hz, 2H), 5.41 (d, *J* = 14.4 Hz, 1H), 4.38 (d, *J* = 14.4 Hz, 1H), 3.61 (s, 3H), 2.55 (s, 3H), 2.17 (s, 3H). ^{13}C NMR (101 MHz, $CDCl_3$) δ 157.08, 144.19, 141.79, 137.64, 136.78, 136.37, 135.96, 131.38, 130.60, 129.91, 129.19, 128.50, 128.27, 127.70, 127.50, 126.93, 126.63, 126.41, 125.63, 125.17, 121.89, 119.88, 119.20, 118.30, 54.44, 53.16, 21.97, 21.43. HRMS (APCI) calc. for $C_{33}H_{29}N_2O_4S^+$ $[M+H]^+$: 549.1792, found 549.1791.

g. Side products

3-Iodo-*N*-tosyl-2-*p*-tolylindole (**4a**)

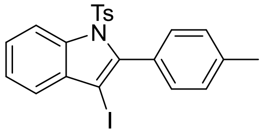

The spectroscopic data match those previously reported in the literature.[43]

^{1}H NMR (300 MHz, $CDCl_3$) δ 8.36 – 8.19 (m, 1H), 7.49 – 7.26 (m, 9H), 7.08 (d, *J* = 8.1 Hz, 2H), 2.46 (s, 3H), 2.31 (s, 3H).
^{13}C NMR (75 MHz, $CDCl_3$) δ 144.90, 141.27, 139.27, 136.97, 135.06, 132.31, 131.54 (2C), 129.40 (2C), 128.55, 128.26 (2C), 126.89 (2C), 125.91, 124.58, 122.08, 116.03, 75.66, 21.60, 21.56.

N-Tosyl-2-*p*-tolylindole (**5a**)

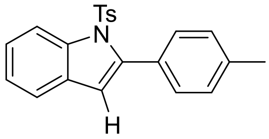

The spectroscopic data match those previously reported in the literature.[44]

^{1}H NMR (400 MHz, $CDCl_3$) δ 8.31 (dq, *J* = 8.4, 0.9 Hz, 1H), 7.50 – 7.33 (m, 4H), 7.31 – 7.17 (m, 5H), 7.09 – 6.96 (m, 2H), 6.52 (d, *J* = 0.8 Hz, 1H), 2.45 (s, 3H), 2.29 (s, 3H).
^{13}C NMR (101 MHz, $CDCl_3$) δ 144.42, 142.27, 138.57, 138.18, 134.62, 131.3448, 130.63, 130.16 (2C), 129.52, 129.13 (2C), 128.26 , 128.22 (2C) , 126.76 (2C), 124.57, 124.24, 120.54, 116.63, 113.25, 21.47, 21.41.

3. NMR Spectra

- Derivatives **1**

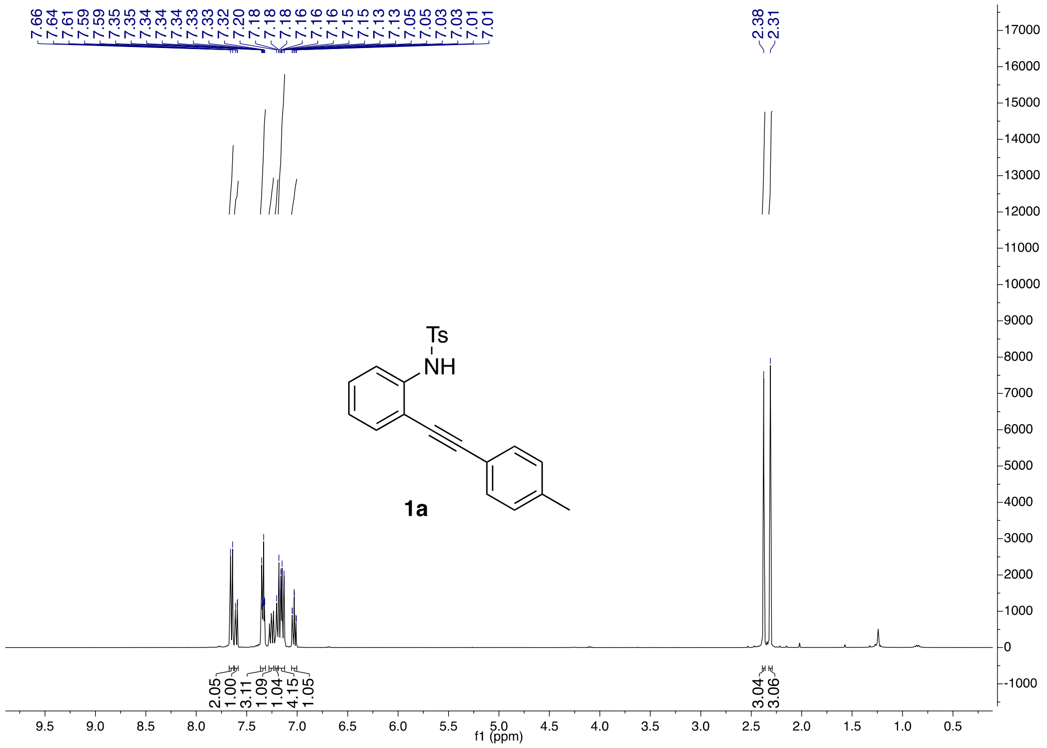

Supplementary Fig. 47 ^{1}H NMR spectrum of compound **1a**

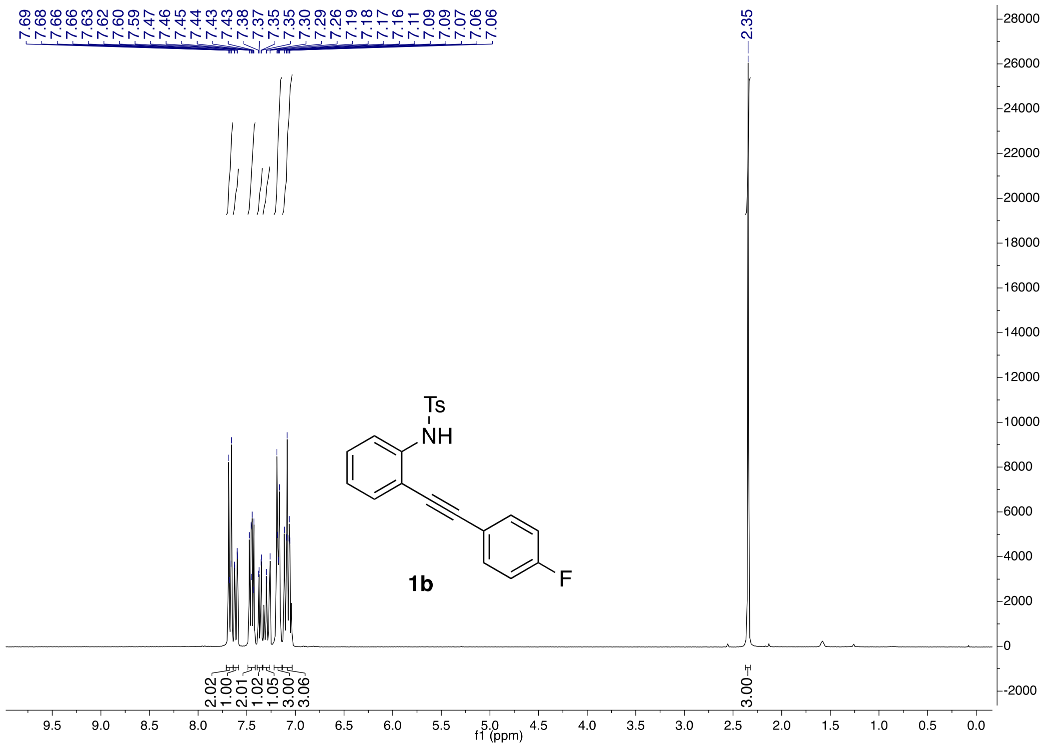

Supplementary Fig. 48 ^{1}H NMR spectrum of compound **1b**

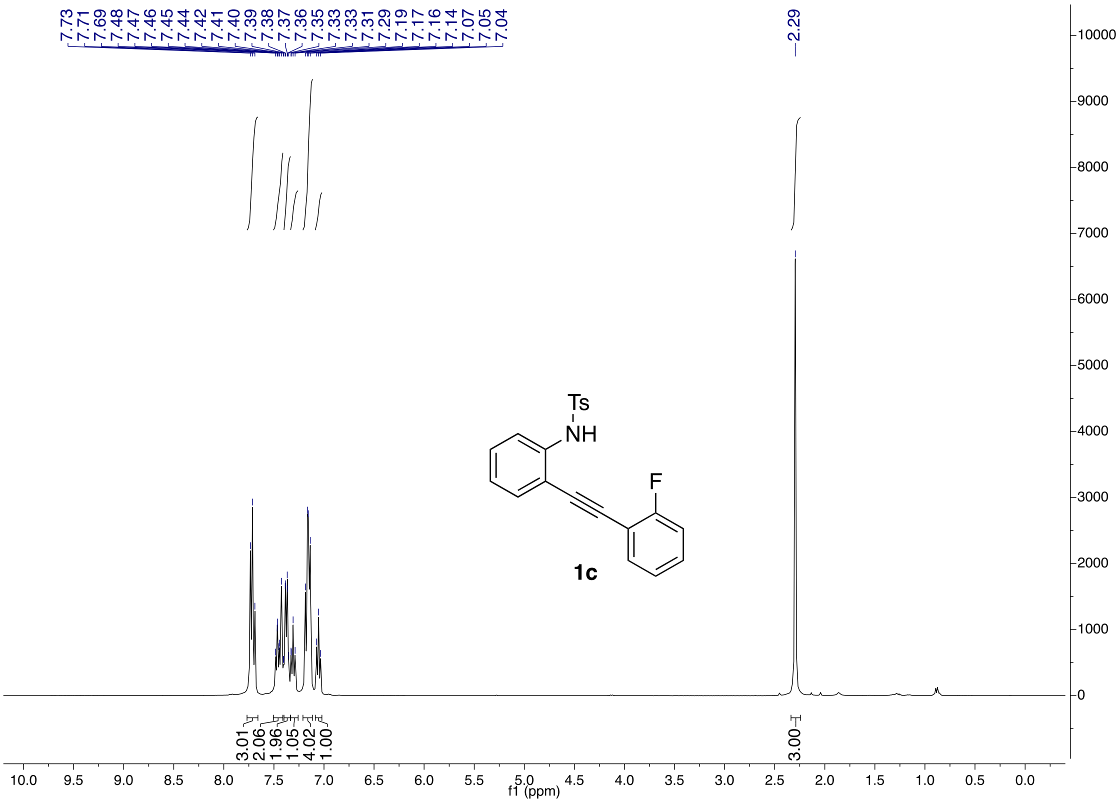

Supplementary Fig. 49 ^{1}H NMR spectrum of compound **1c**

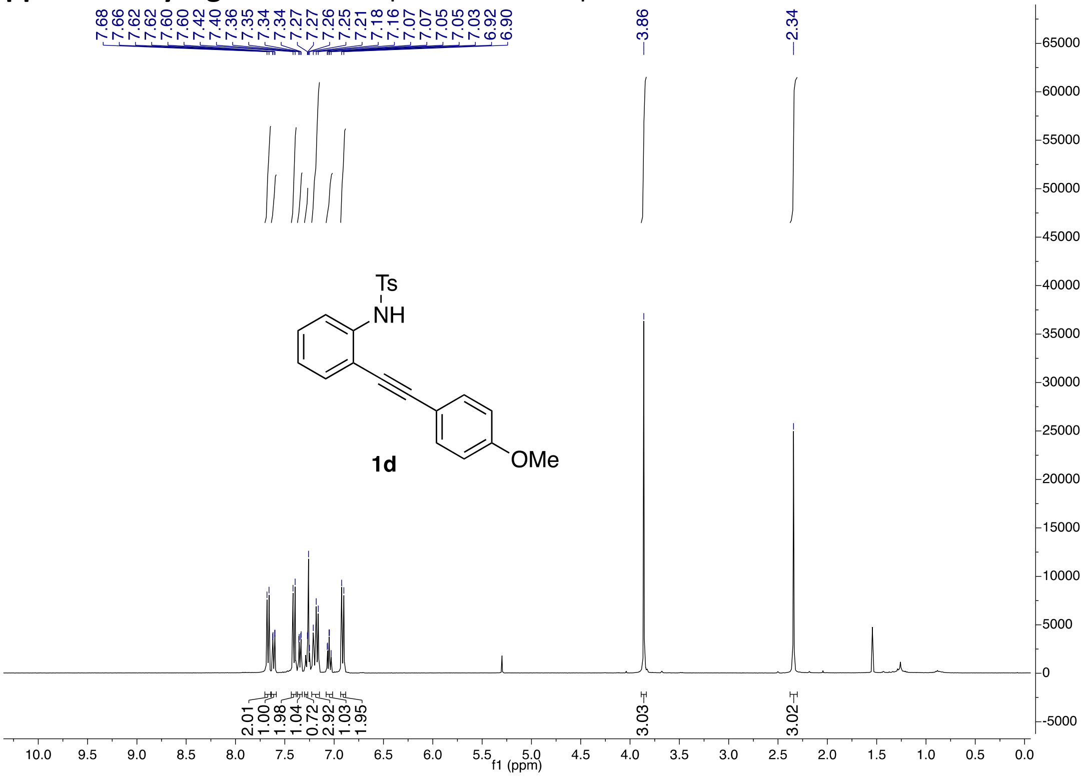

Supplementary Fig. 50 ^{1}H NMR spectrum of compound **1d**

Ts

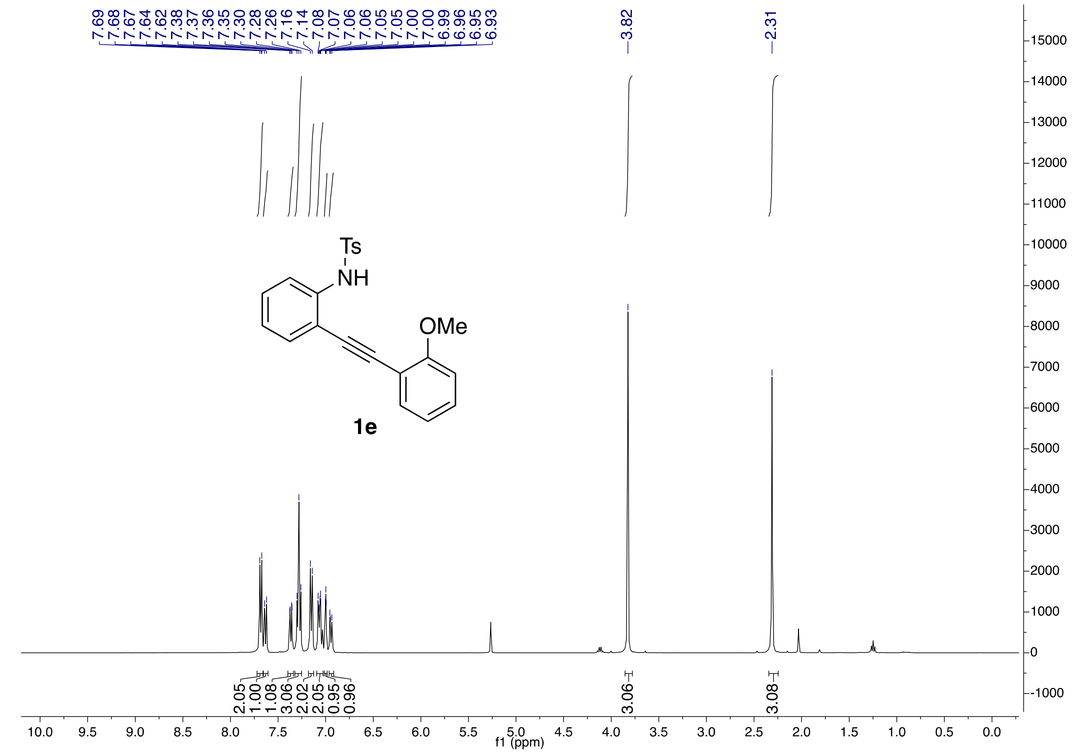

Supplementary Fig. 51 ^{1}H NMR spectrum of compound **1e**

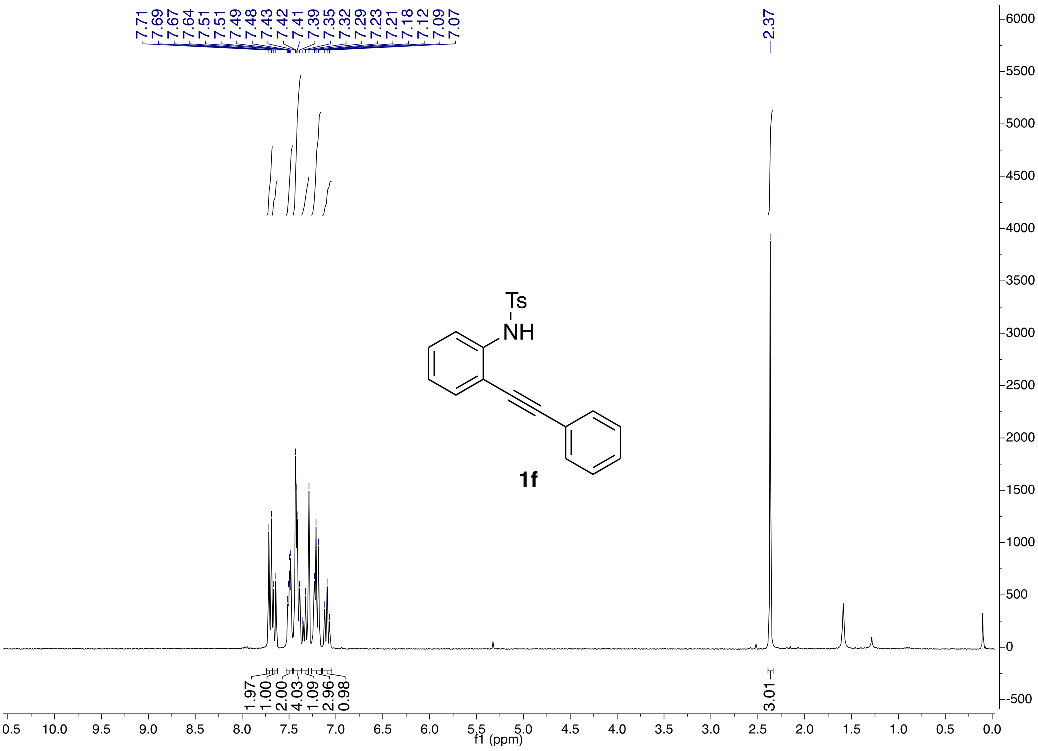

Supplementary Fig. 52 ^{1}H NMR spectrum of compound **1f**

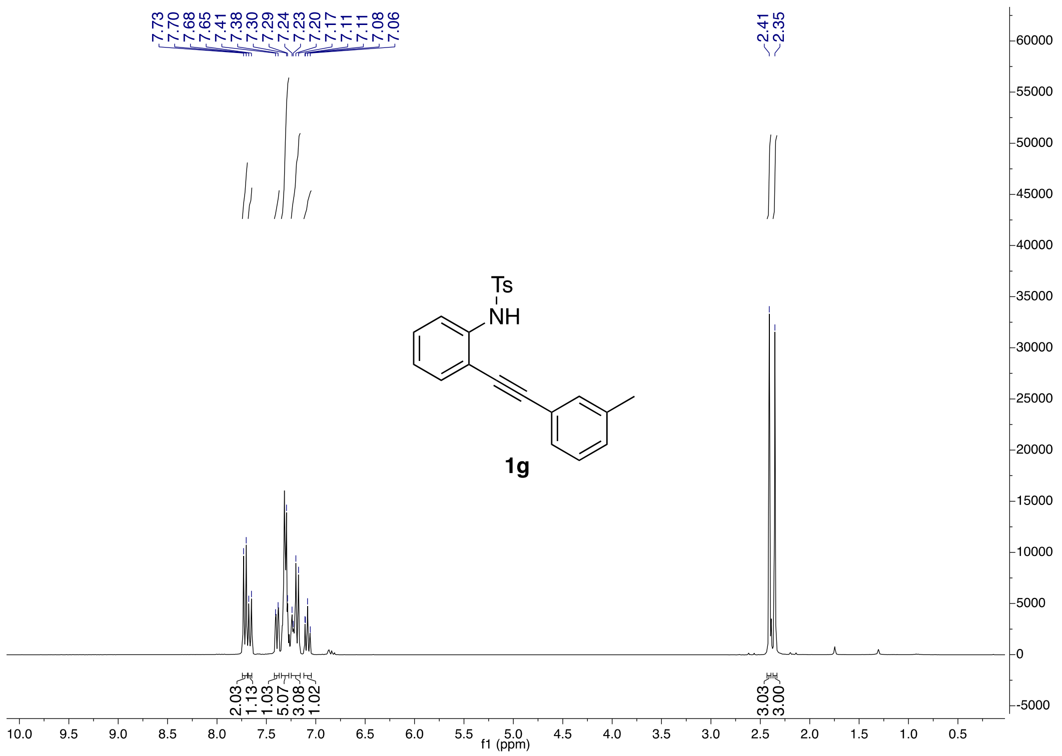

Supplementary Fig. 53 ^{1}H NMR spectrum of compound **1g**

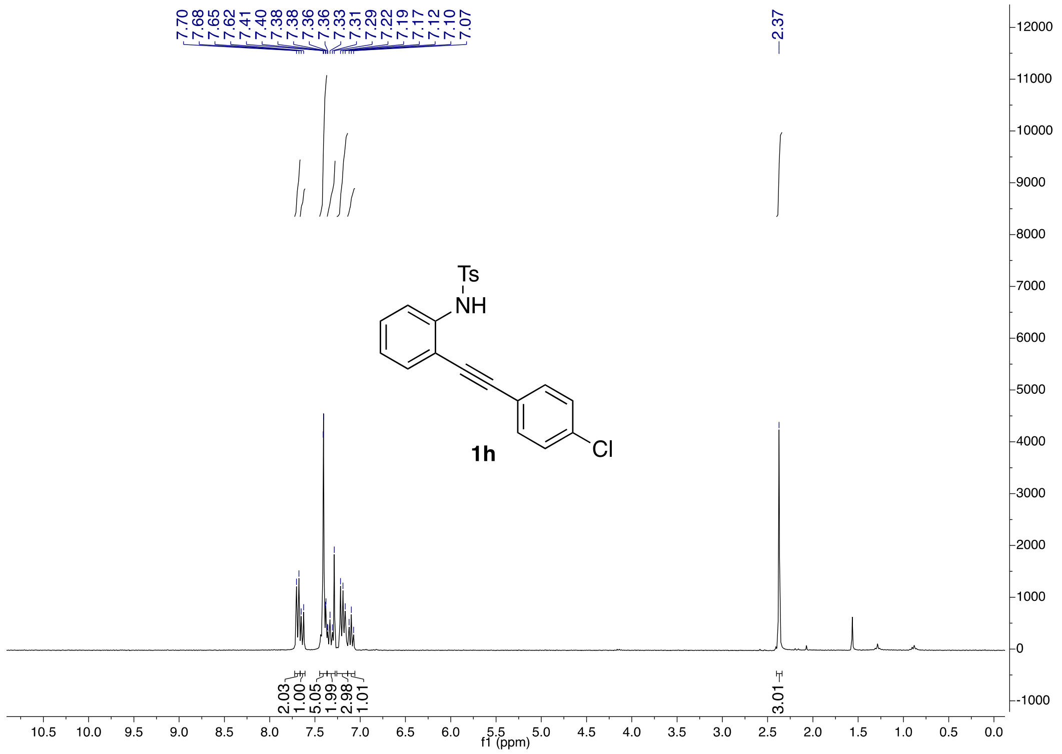

Supplementary Fig. 54 ^{1}H NMR spectrum of compound **1h**

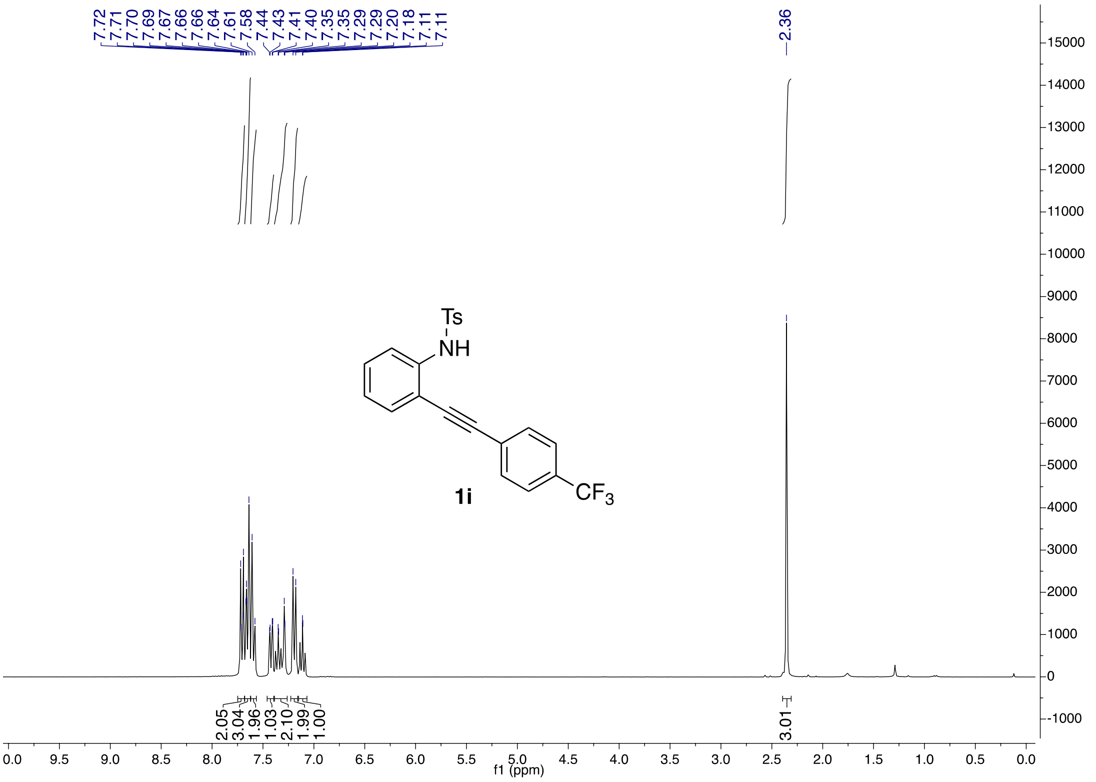

Supplementary Fig. 55 ^{1}H NMR spectrum of compound **1i**

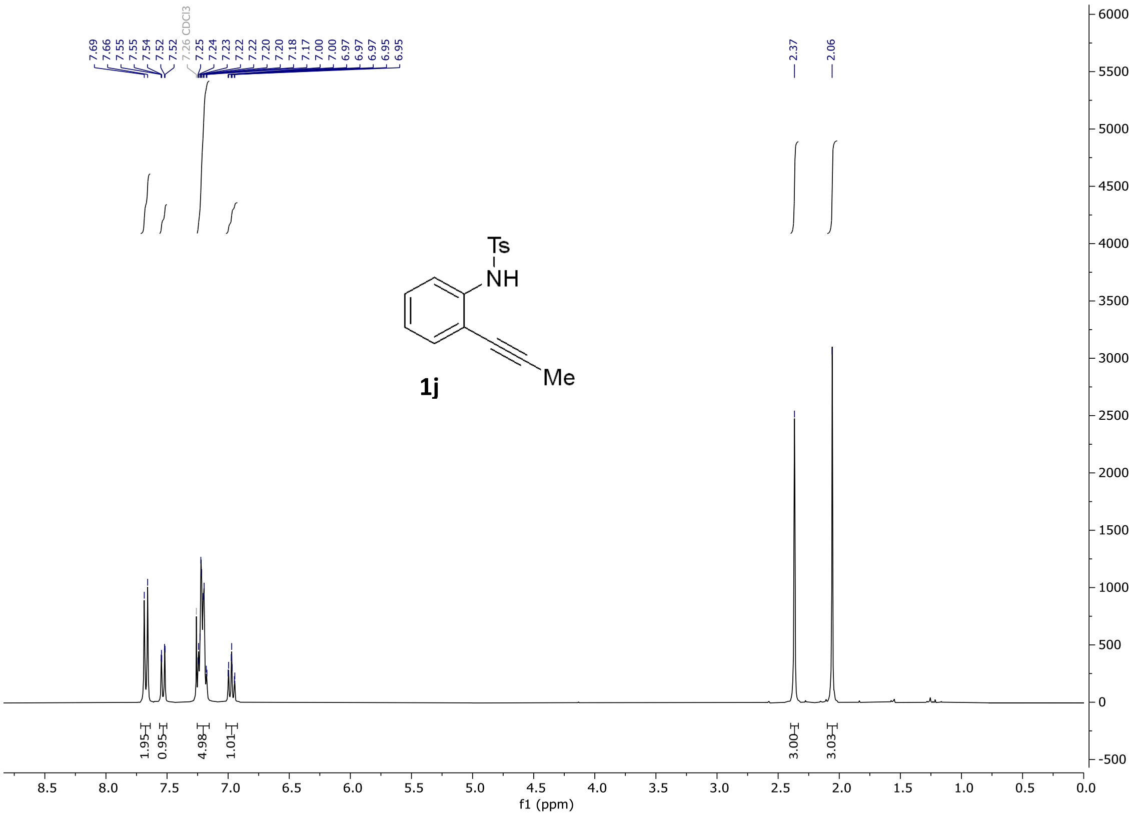

Supplementary Fig. 56 ^{1}H NMR spectrum of compound **1j**

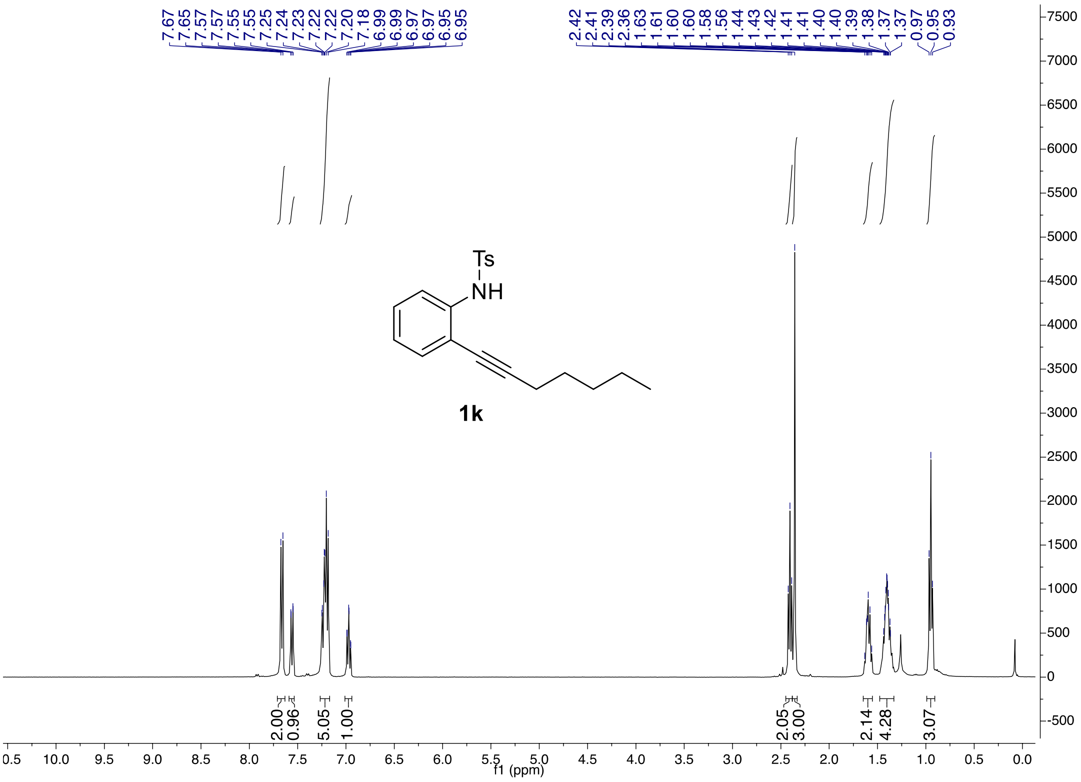

Supplementary Fig. 57 ^{1}H NMR spectrum of compound **1k**

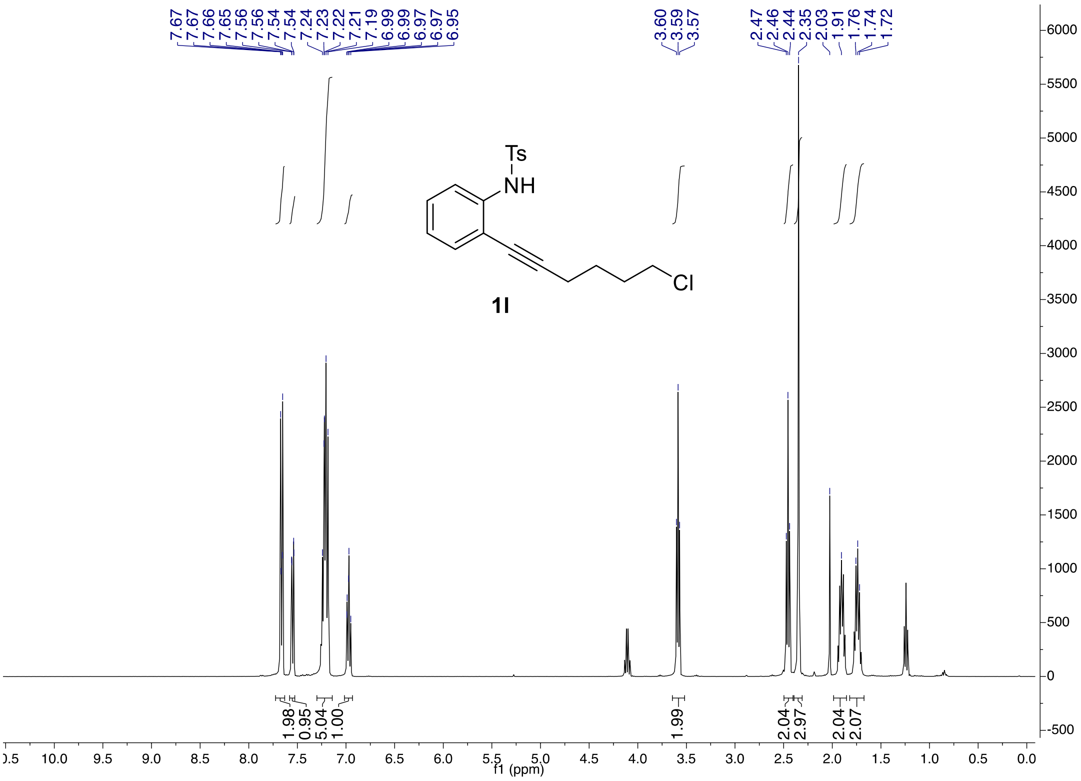

Supplementary Fig. 58 ^{1}H NMR spectrum of compound **1l**

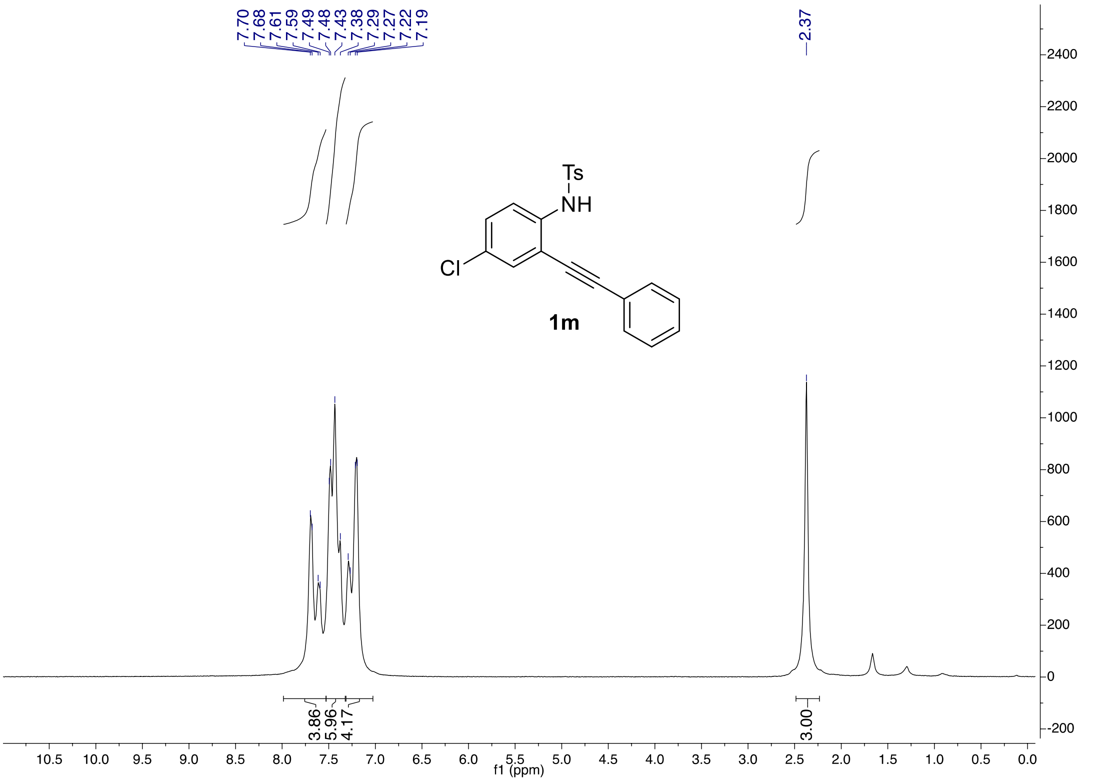

Supplementary Fig. 59 ^{1}H NMR spectrum of compound **1m**

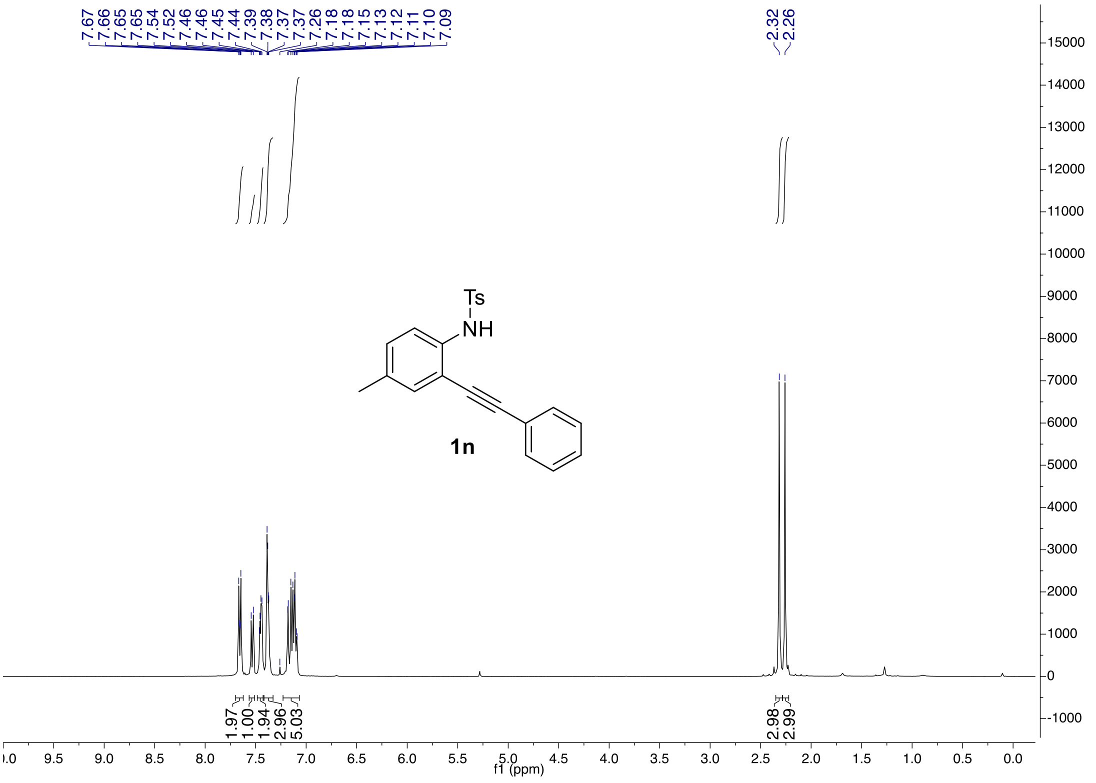

Supplementary Fig. 60 ^{1}H NMR spectrum of compound **1n**

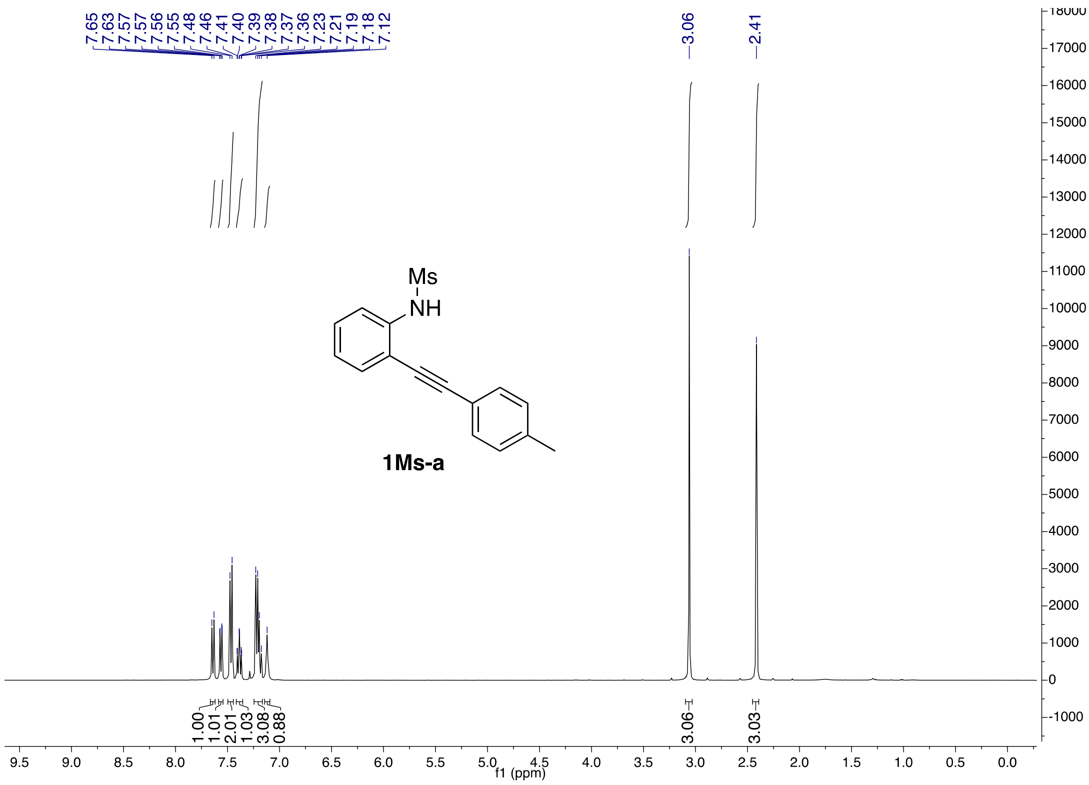

Supplementary Fig. 61 ^{1}H NMR spectrum of compound **1Ms-a**

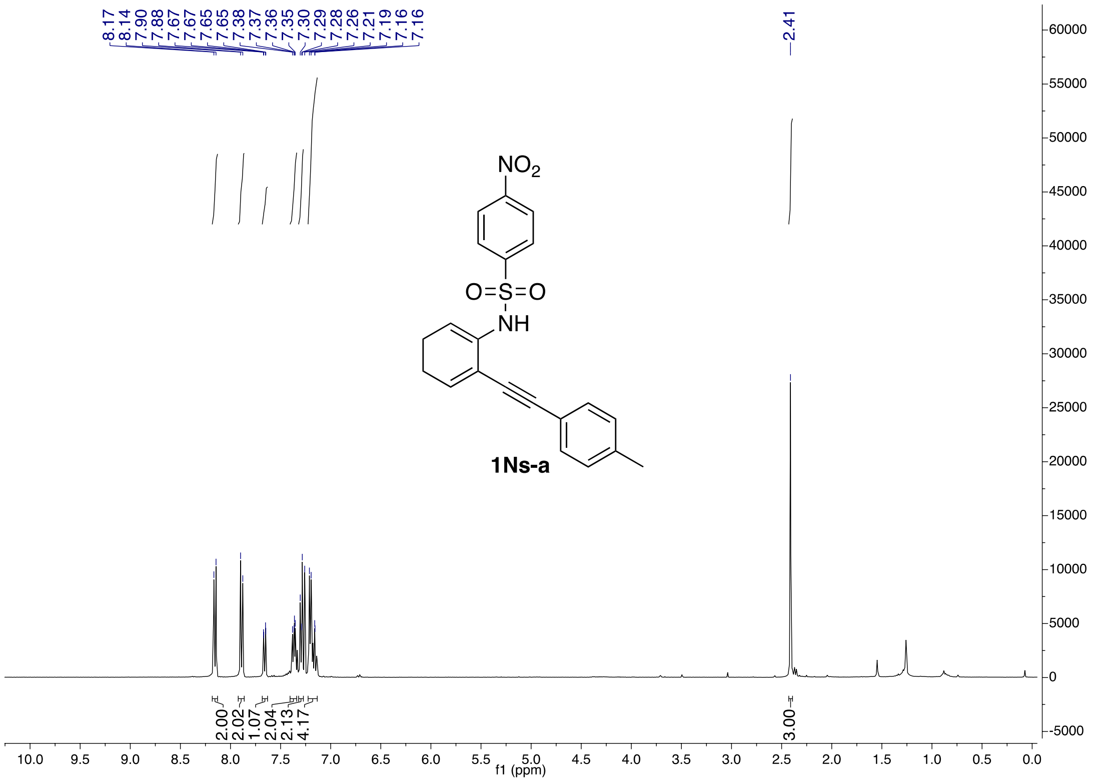

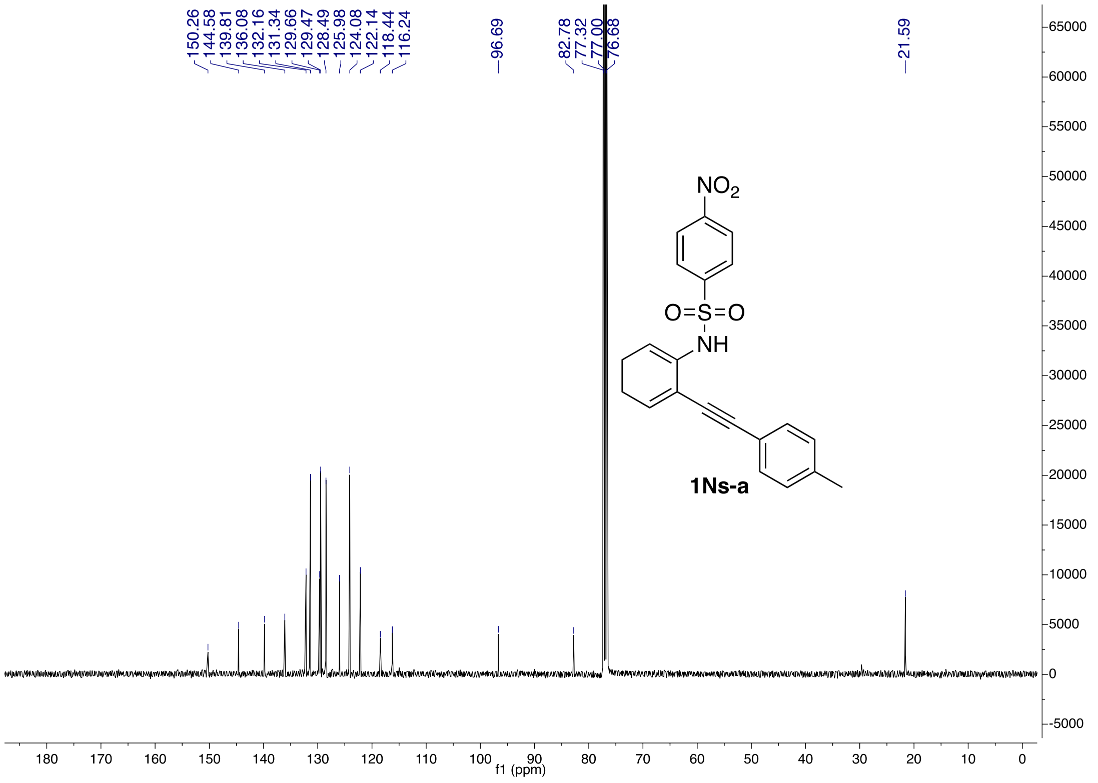

Supplementary Fig. 62 ^{1}H NMR and ^{13}C NMR spectra of compound **1Ns-a**

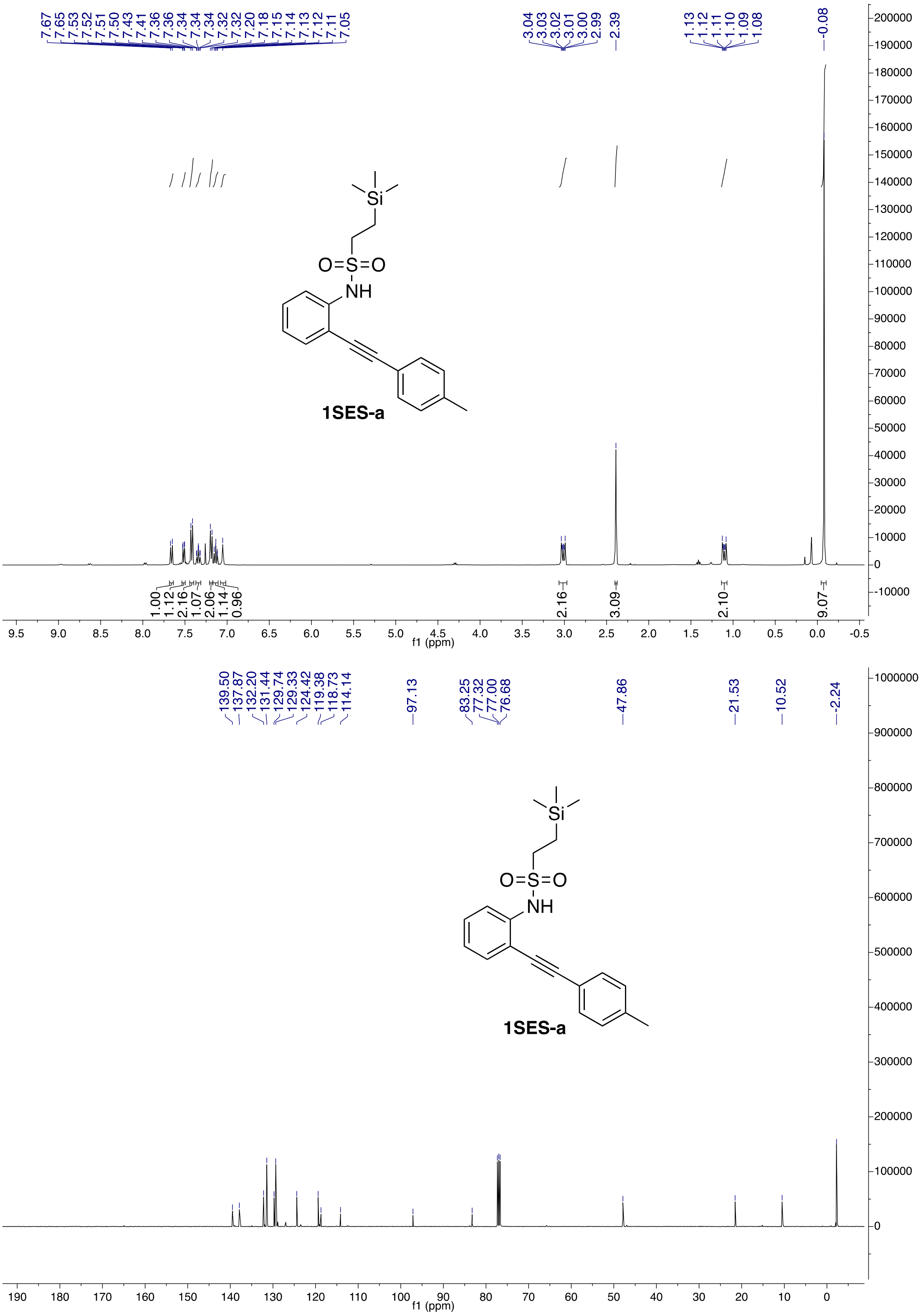

Supplementary Fig. 63 ^{1}H NMR and ^{13}C NMR spectra of compound **1SES-a**

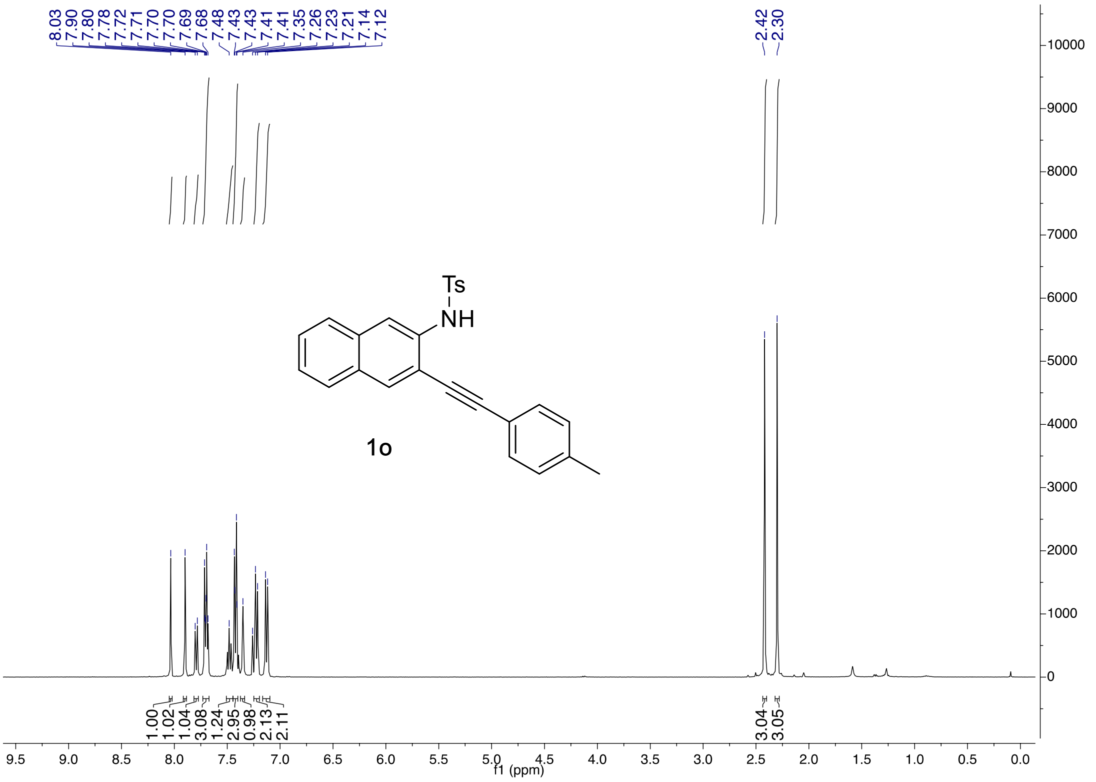

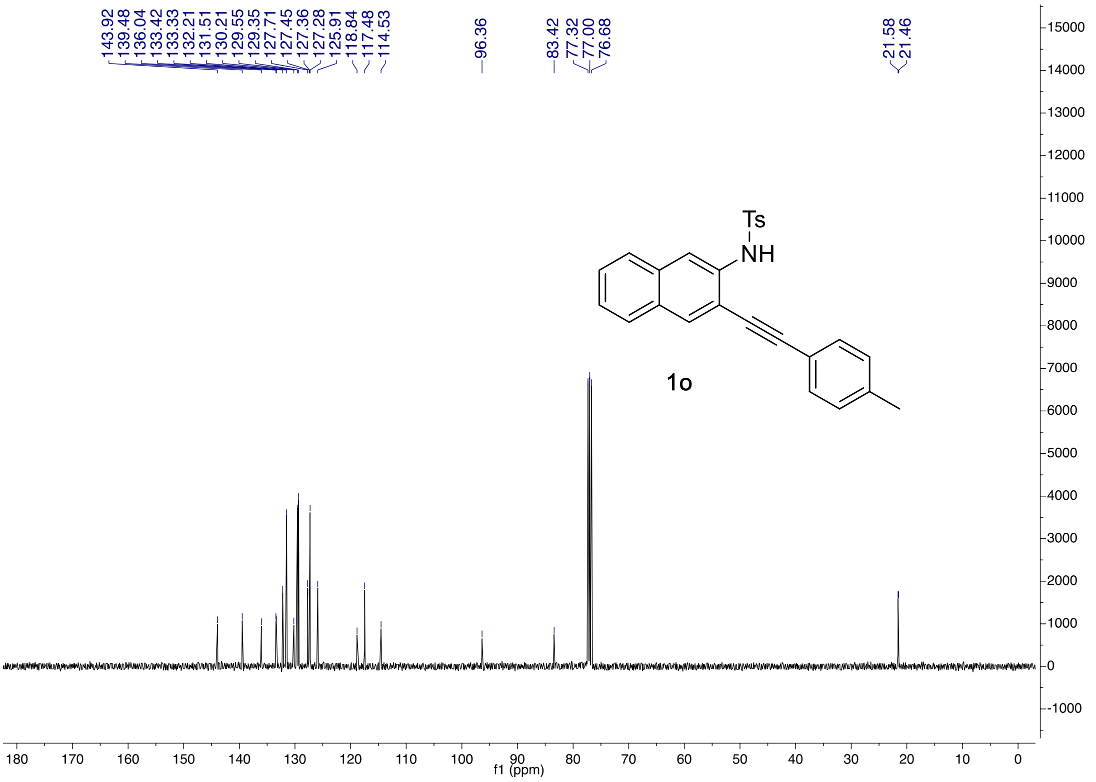

Supplementary Fig. 64 ^{1}H NMR and ^{13}C NMR spectra of compound **1o**

- Alkynyls iodide **2** and **7**

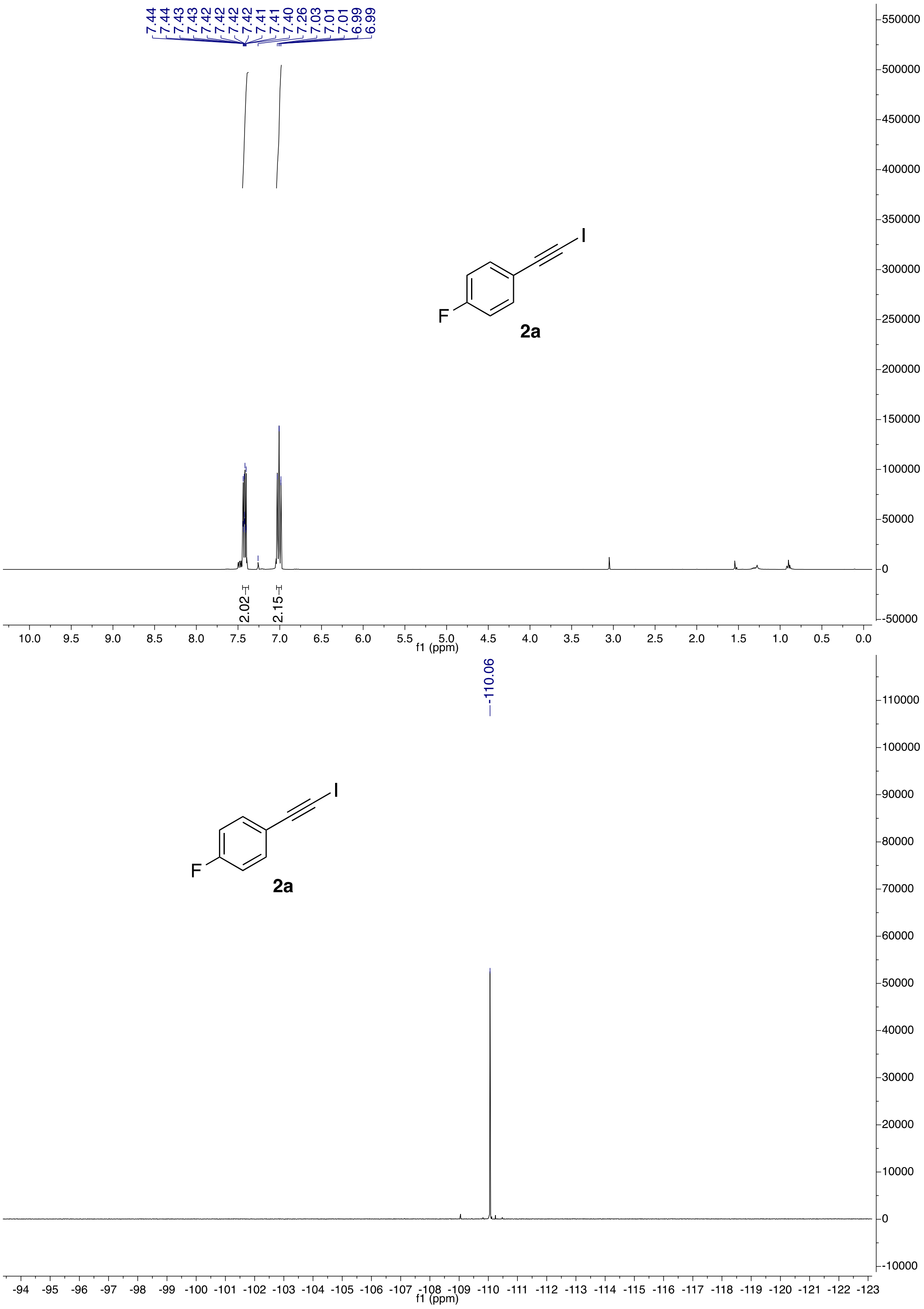

Supplementary Fig. 65 ^{1}H NMR and ^{19}F NMR spectra of compound **2a**

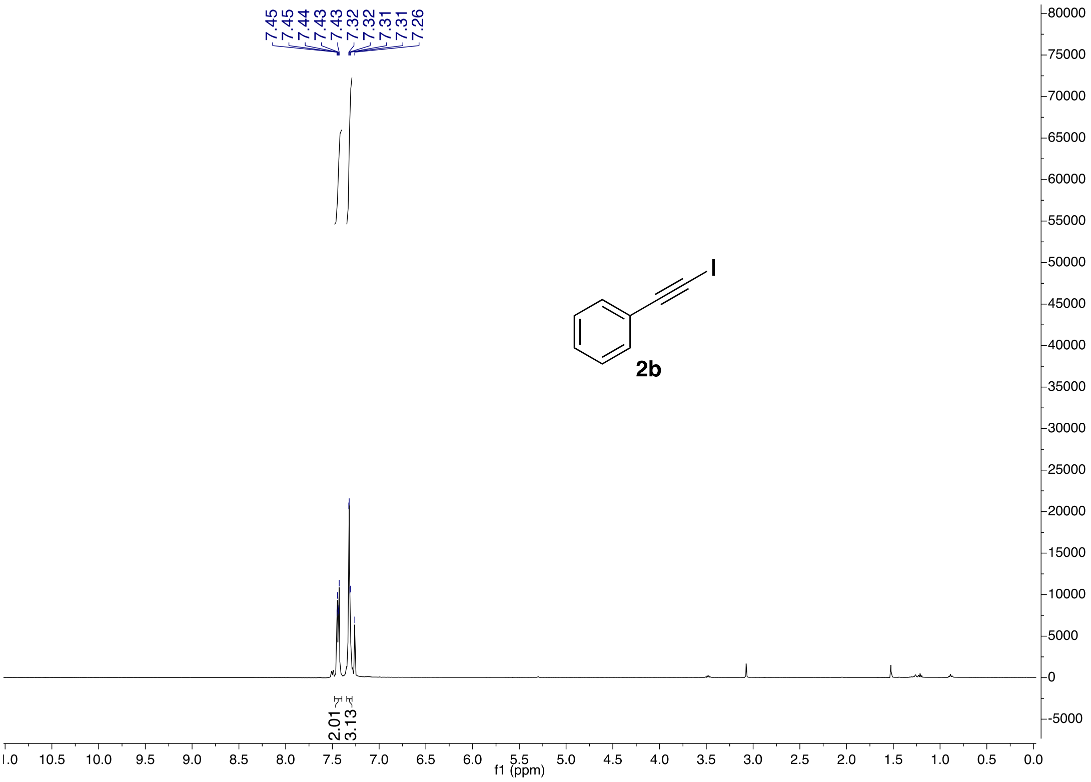

Supplementary Fig. 66 ^{1}H NMR spectrum of compound **2b**

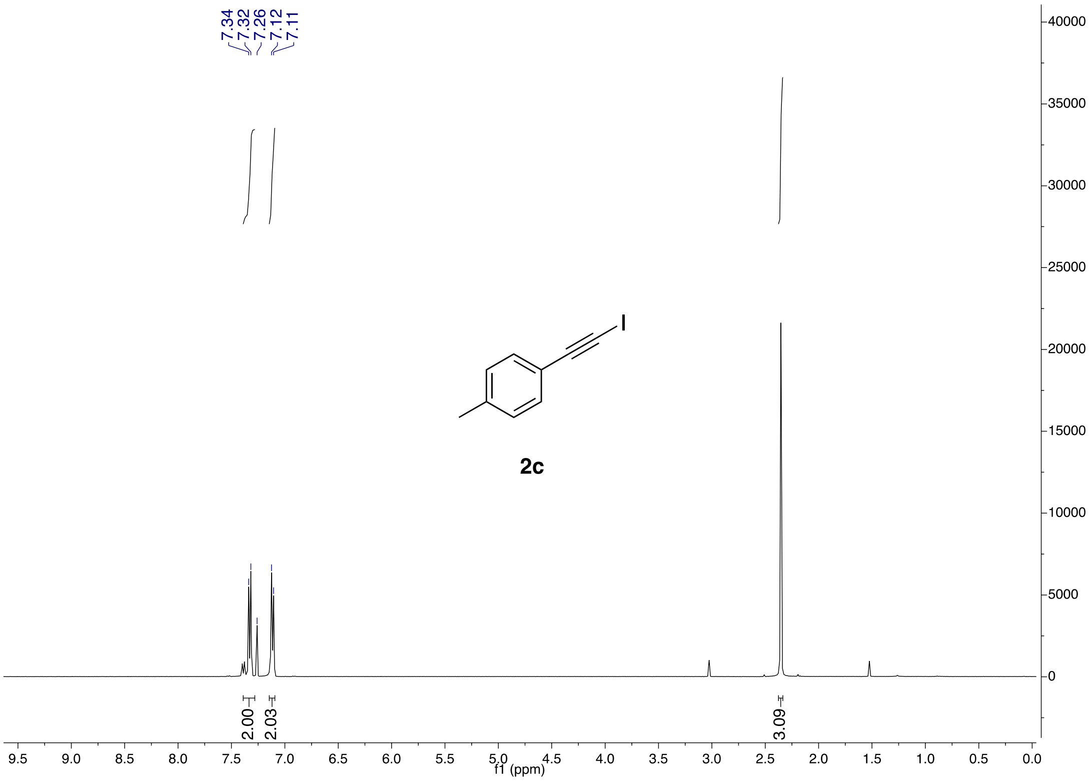

Supplementary Fig. 67 ^{1}H NMR spectrum of compound **2c**

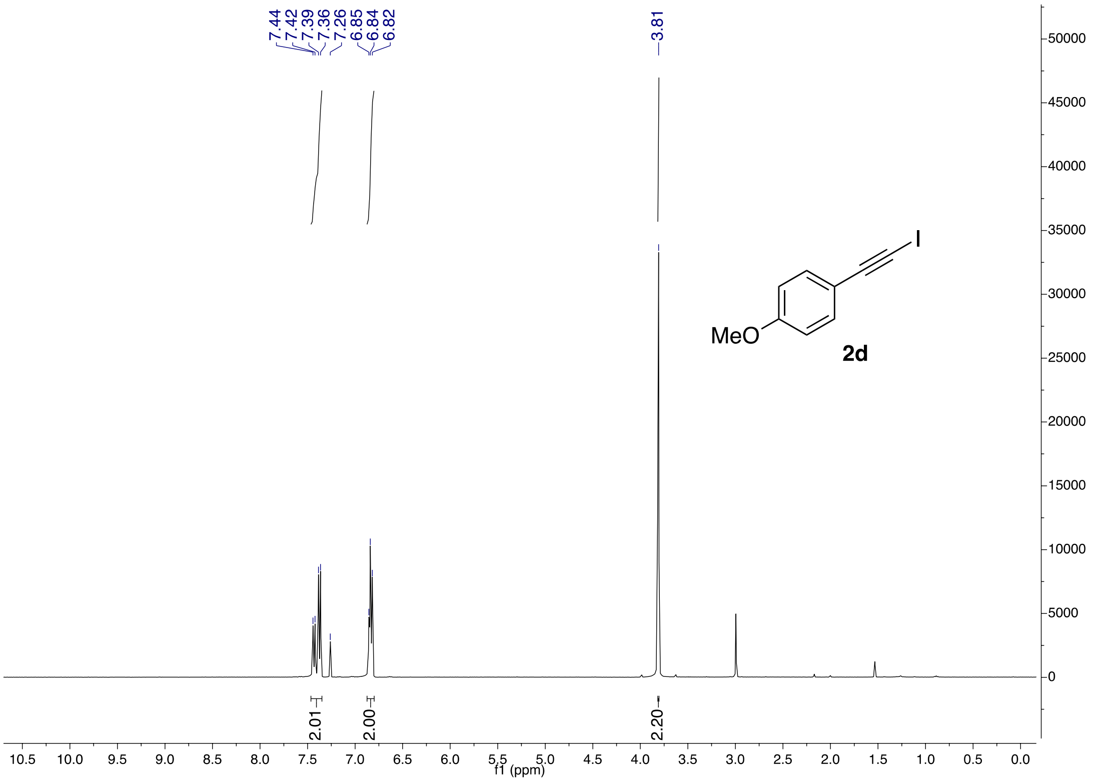

2d

Supplementary Fig. 68 ^{1}H NMR spectrum of compound **2d**

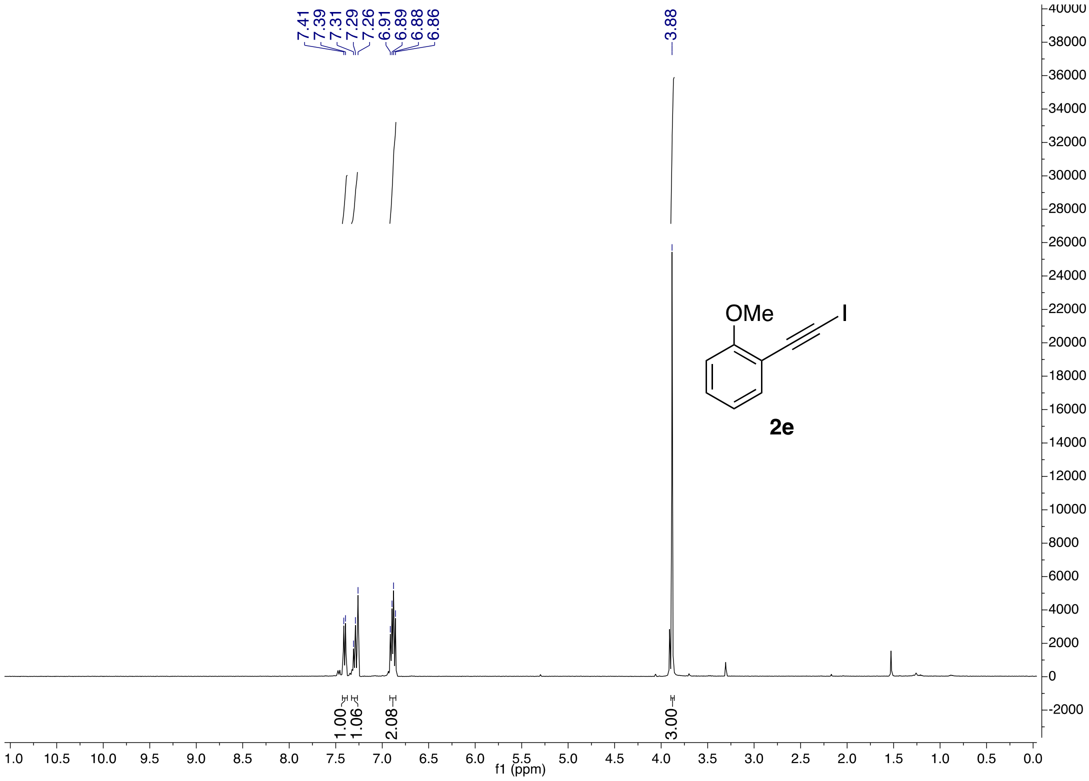

2e

Supplementary Fig. 69 ^{1}H NMR spectrum of compound **2e**

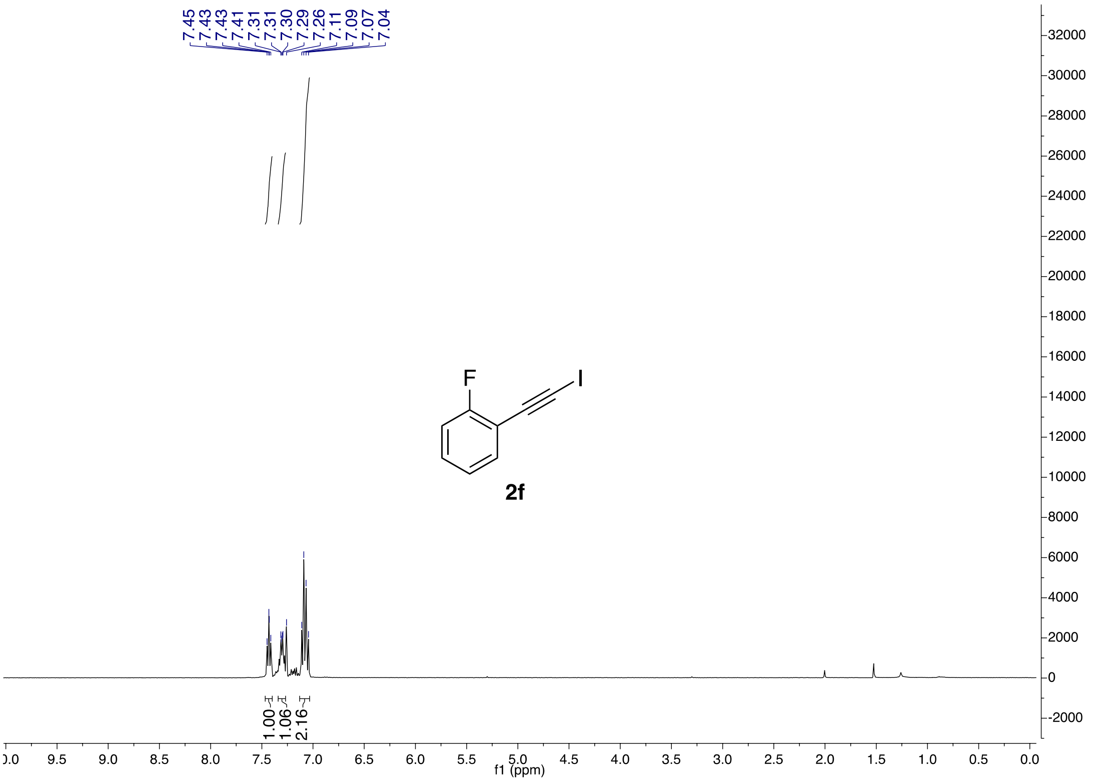

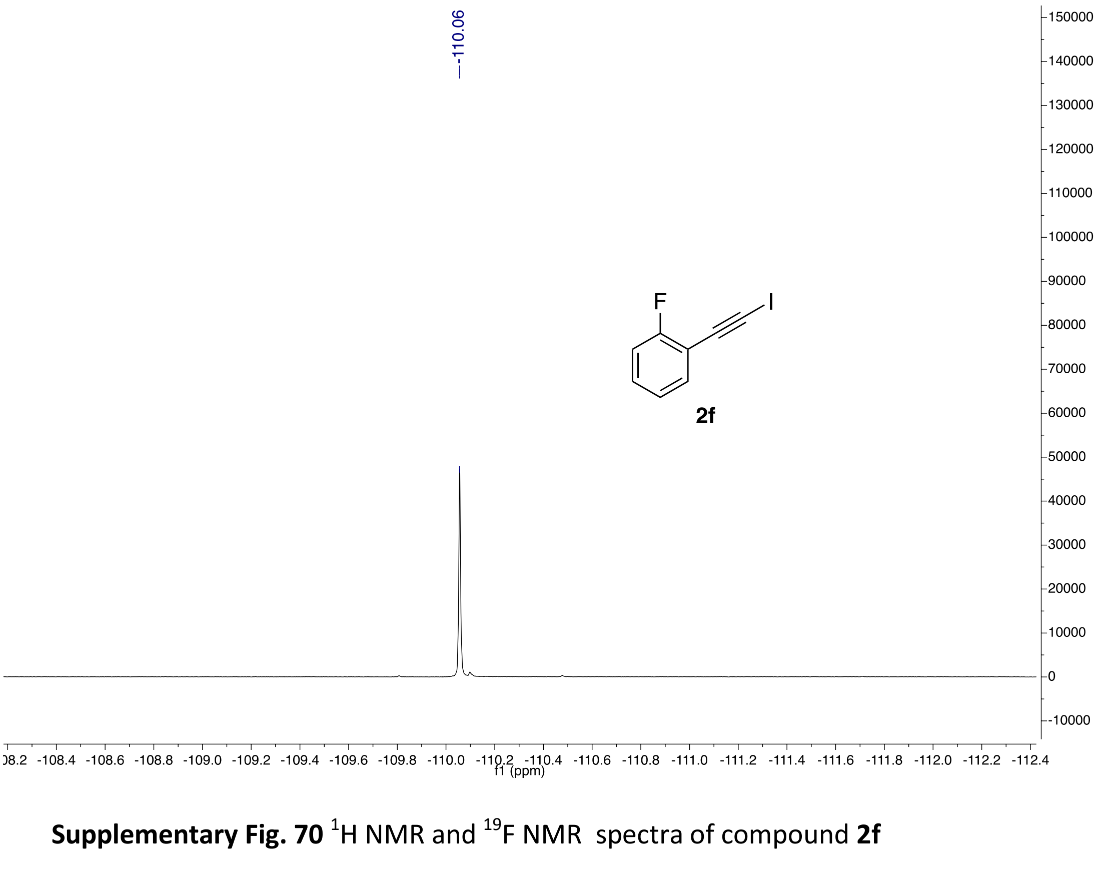

Supplementary Fig. 70 ^{1}H NMR and ^{19}F NMR spectra of compound **2f**

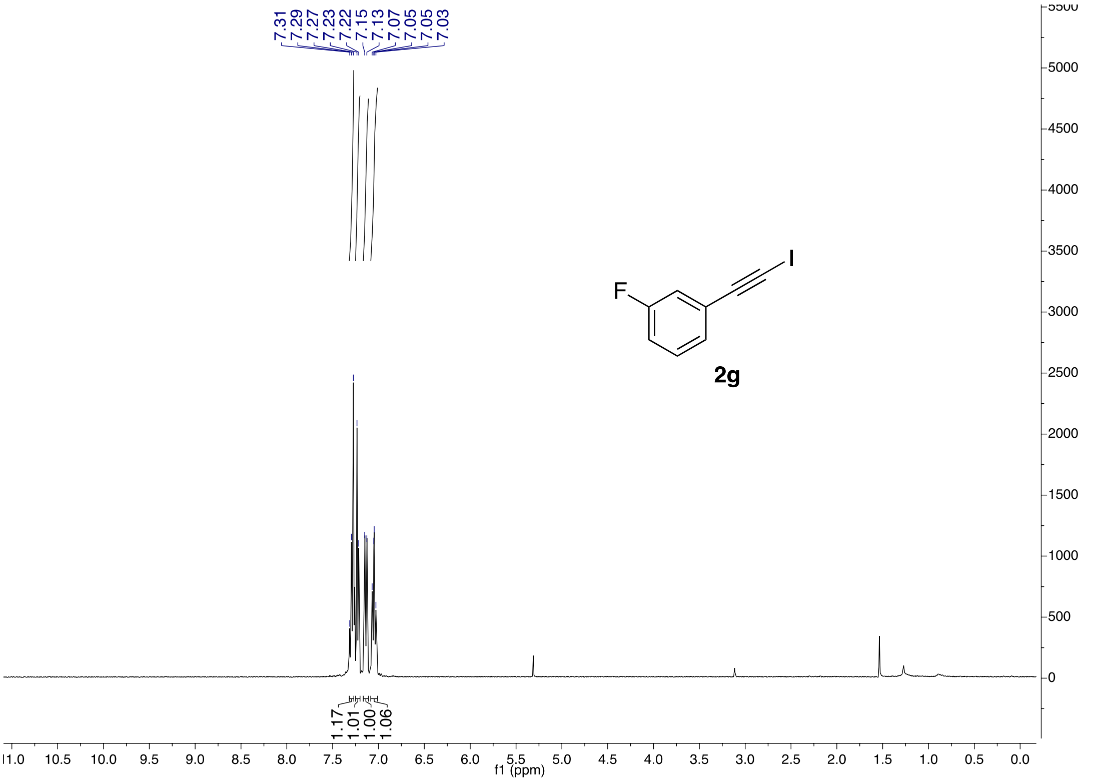

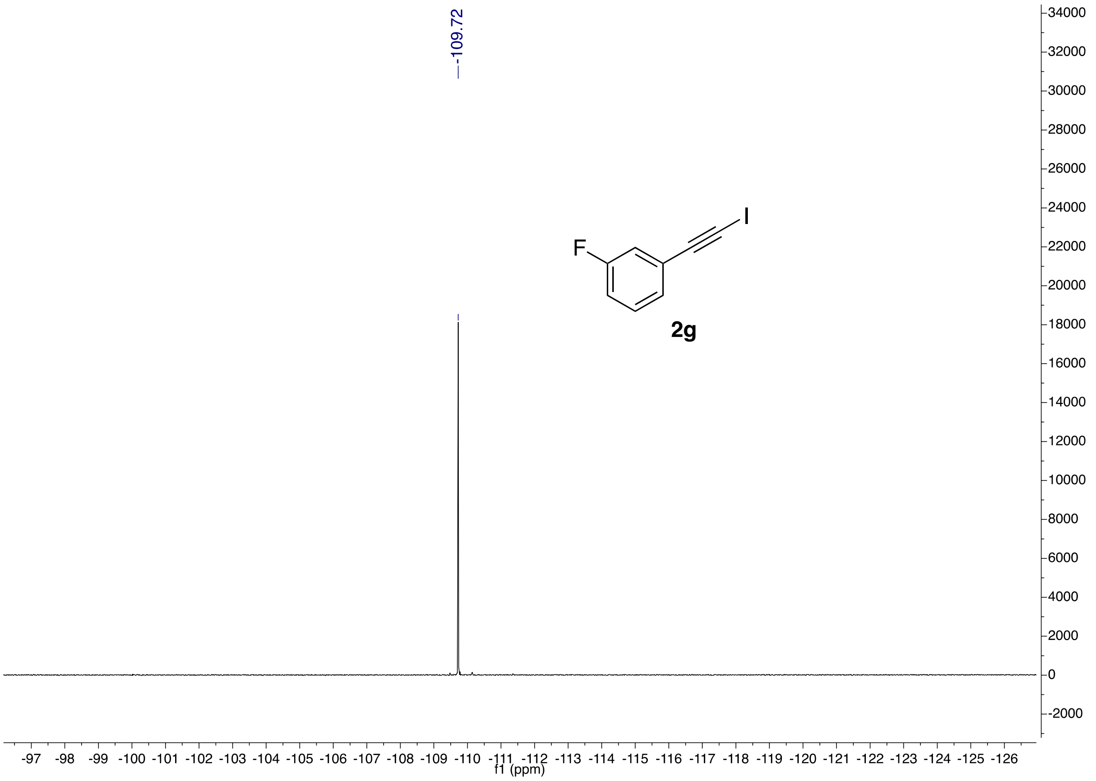

Supplementary Fig. 71 ^{1}H NMR and ^{19}F NMR spectra of compound **2g**

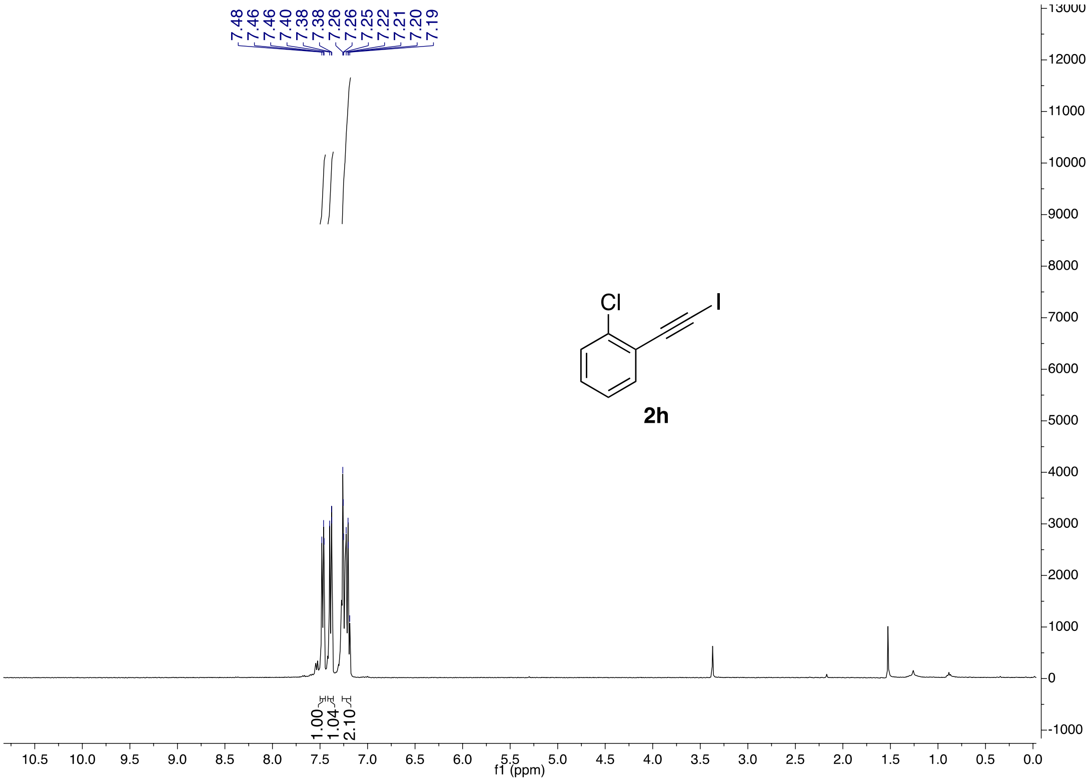

Supplementary Fig. 72 ^{1}H NMR spectrum of compound **2h**

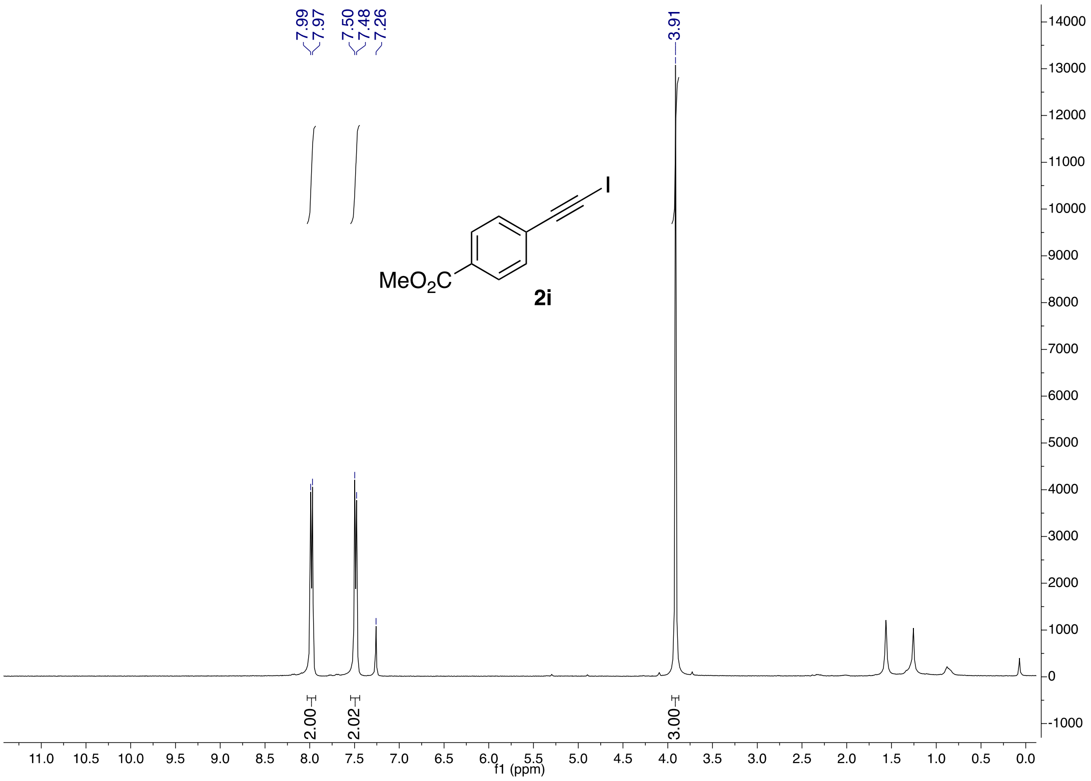

Supplementary Fig. 73 ^{1}H NMR spectrum of compound **2i**

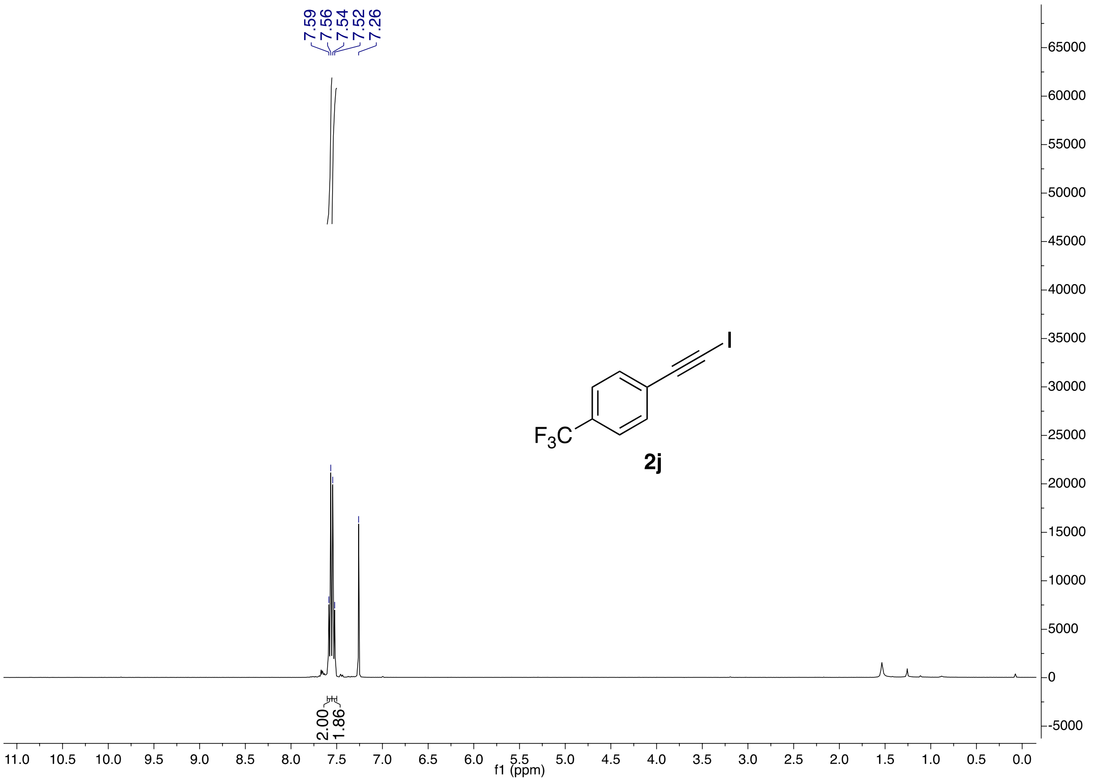

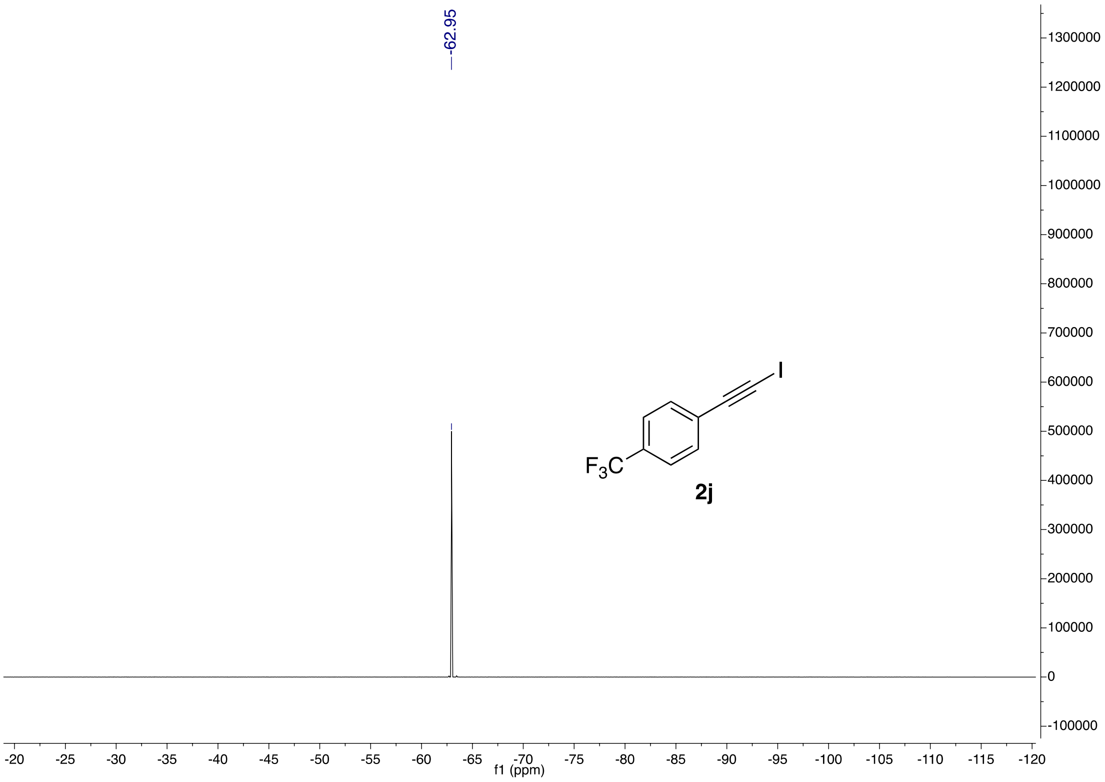

Supplementary Fig. 74 ^{1}H NMR and ^{19}F NMR spectra of compound **2j**

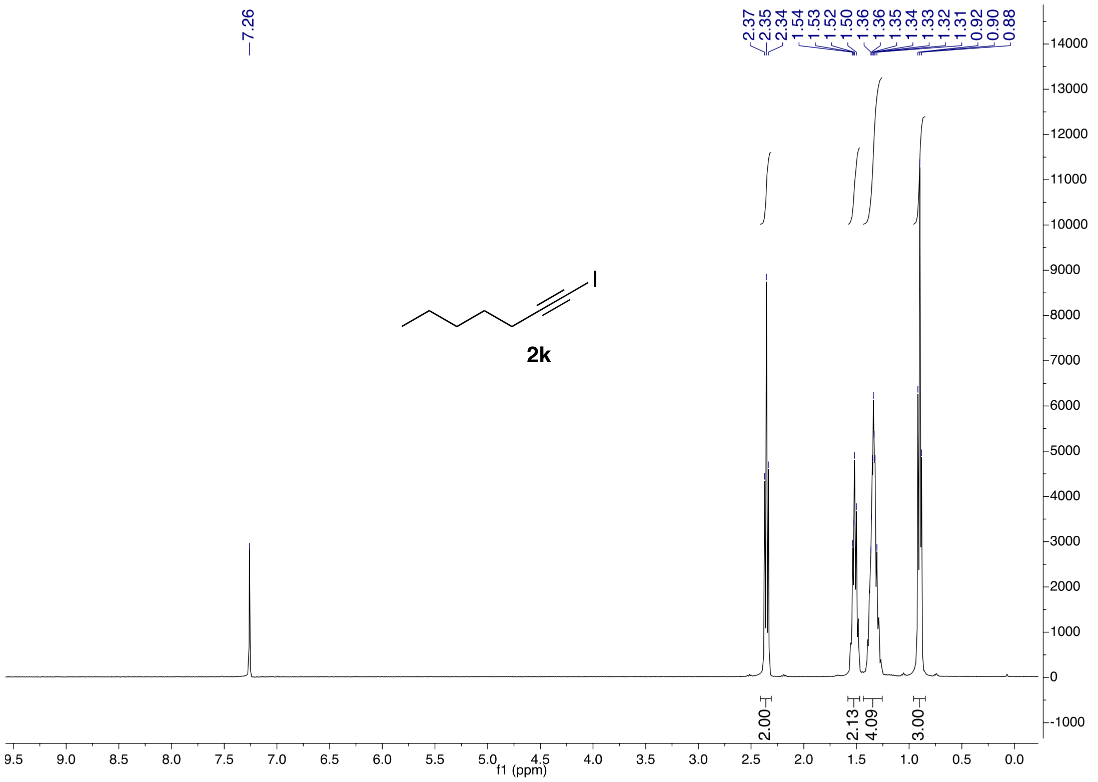

Supplementary Fig. 75 ^{1}H NMR spectrum of compound **2k**

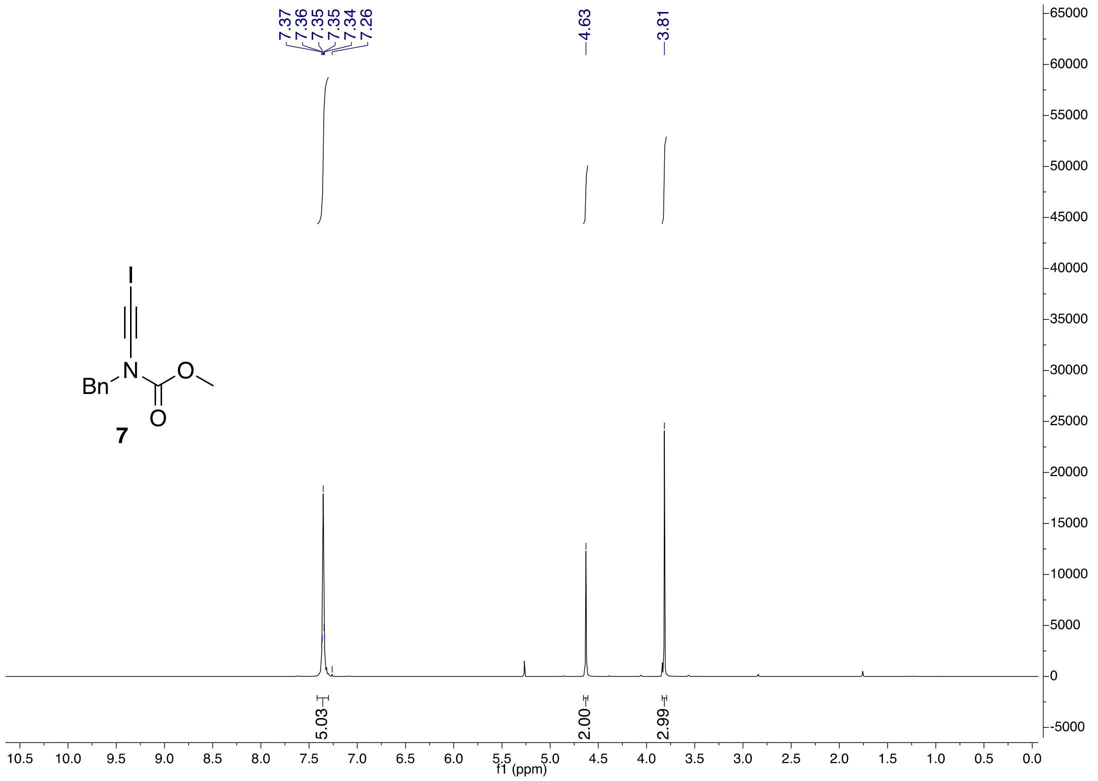

Supplementary Fig. 76 ^{1}H NMR spectrum of compound **7**

- <u>Vinylgold(I) complex **6**</u>

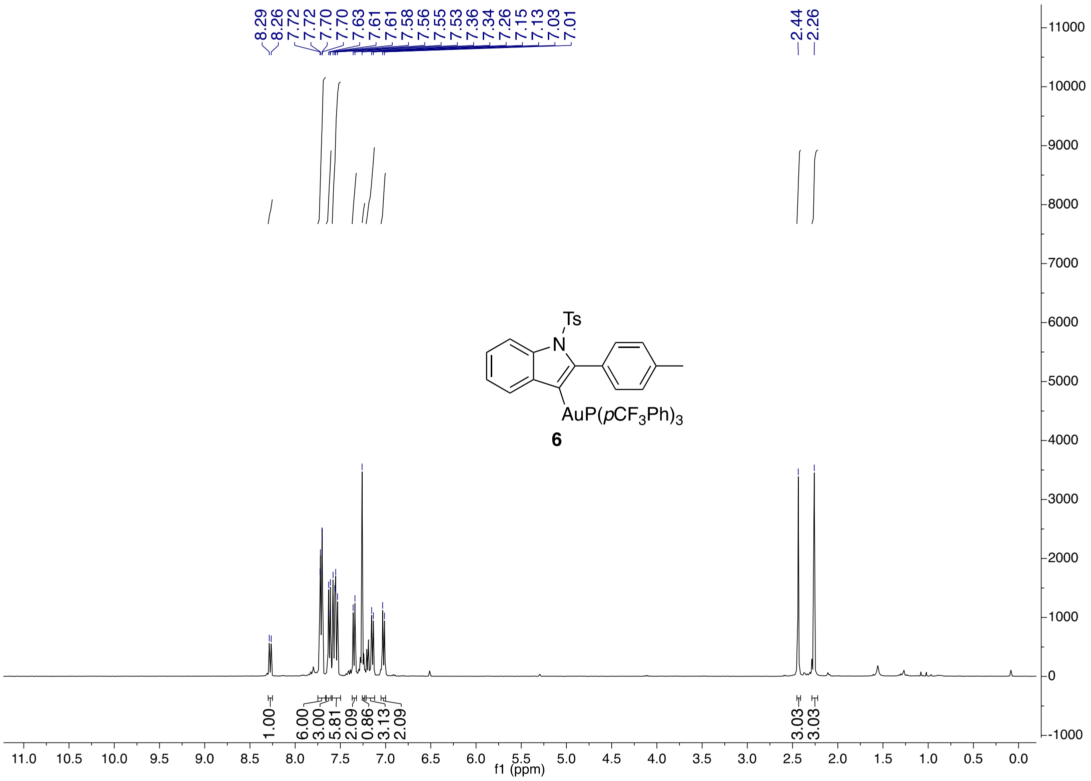

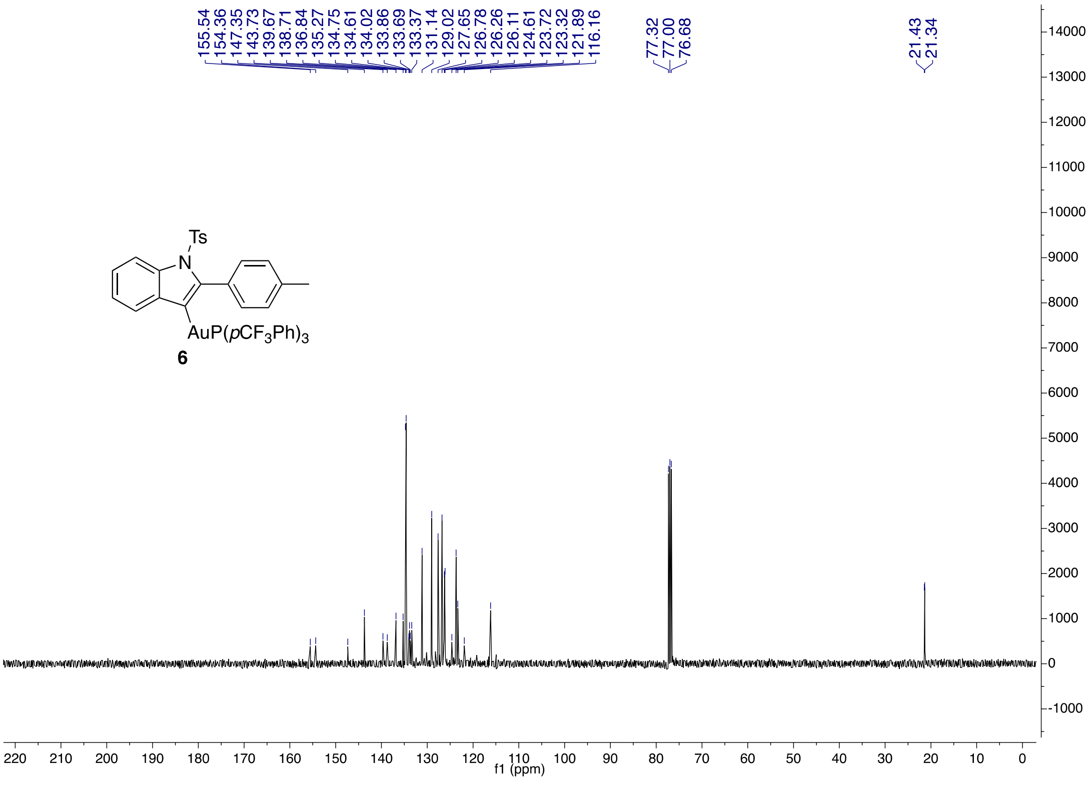

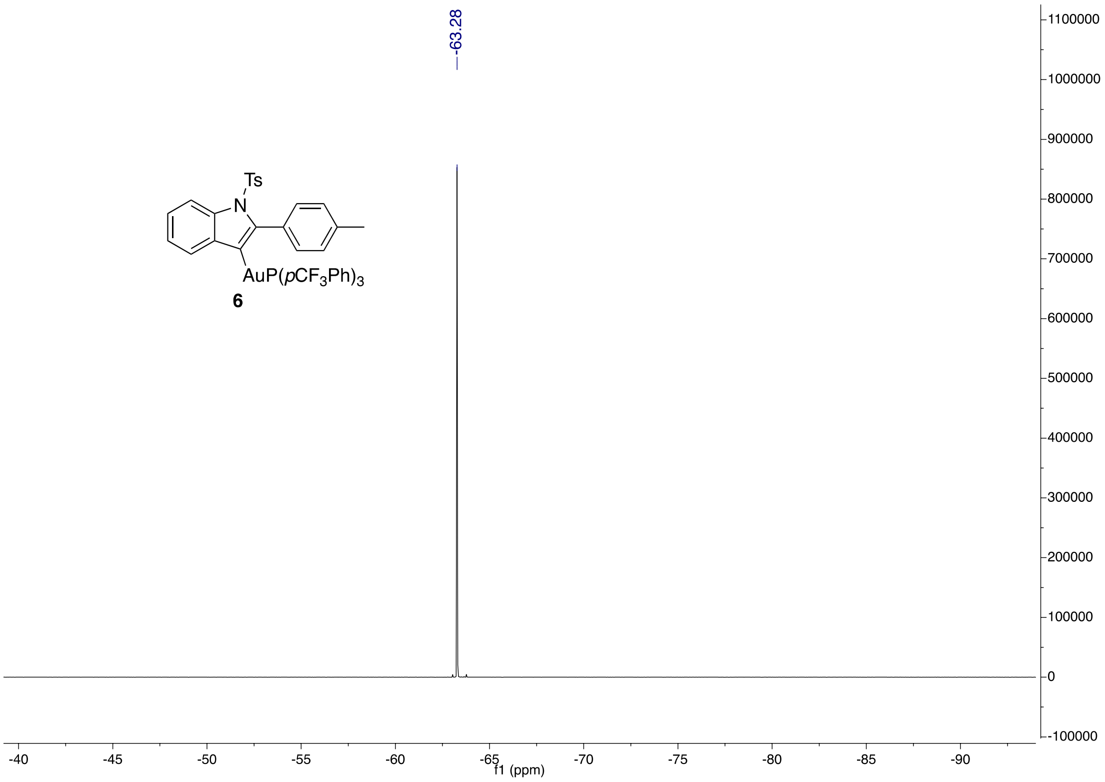

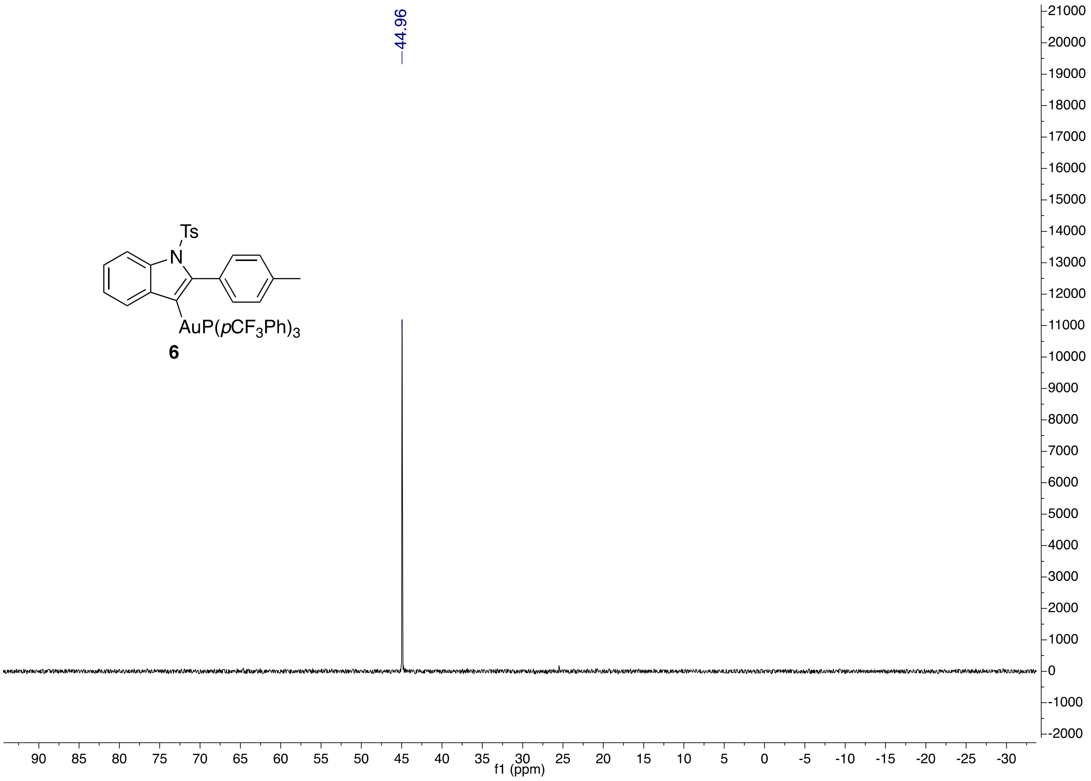

Supplementary Fig. 77 ^{1}H NMR, ^{13}C NMR, ^{19}F NMR and ^{31}P NMR spectra of compound **6**

- Indole derivatives

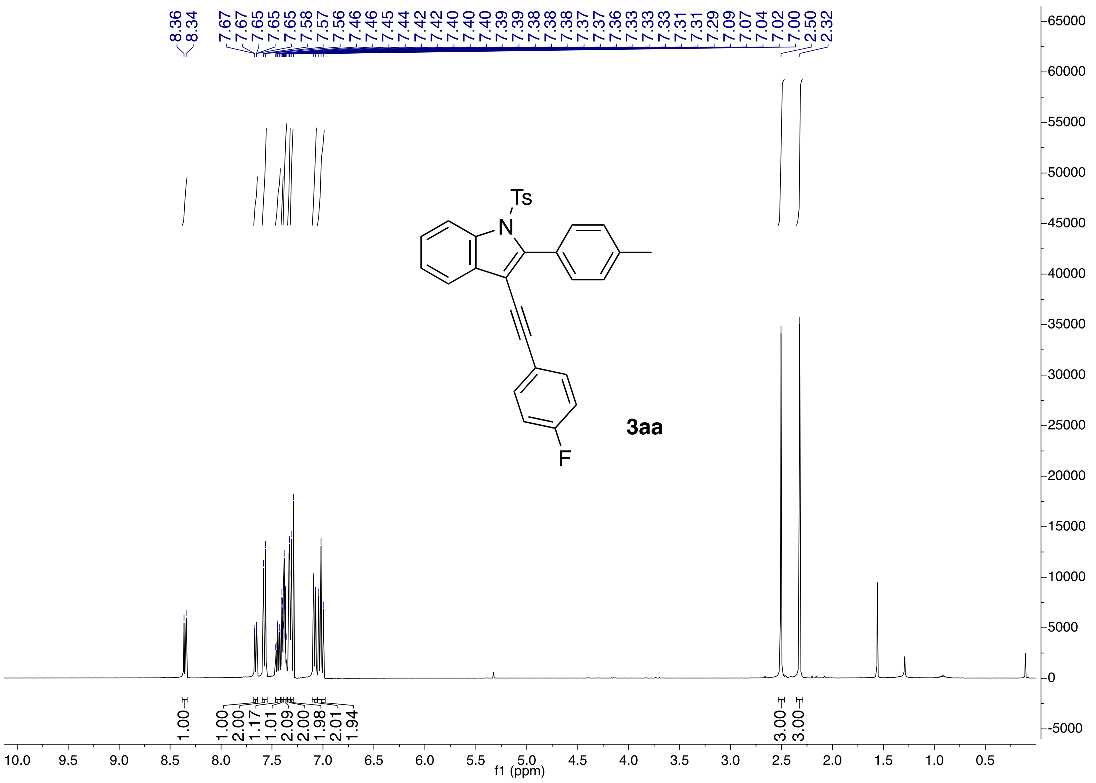

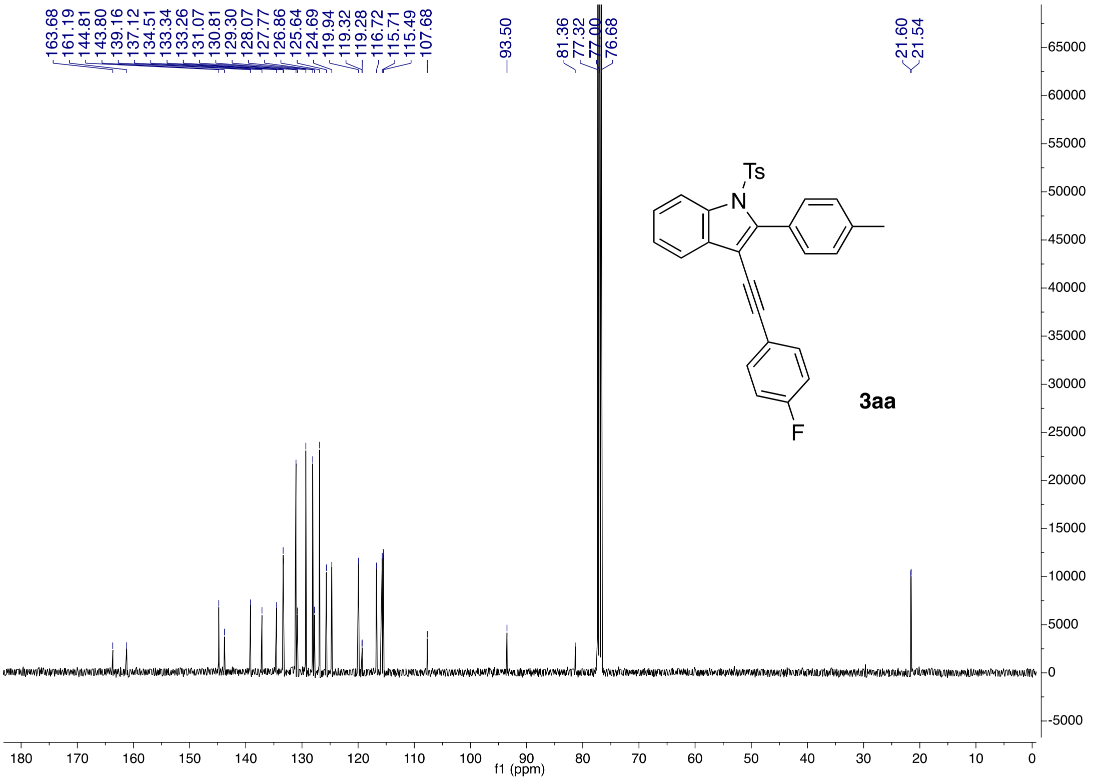

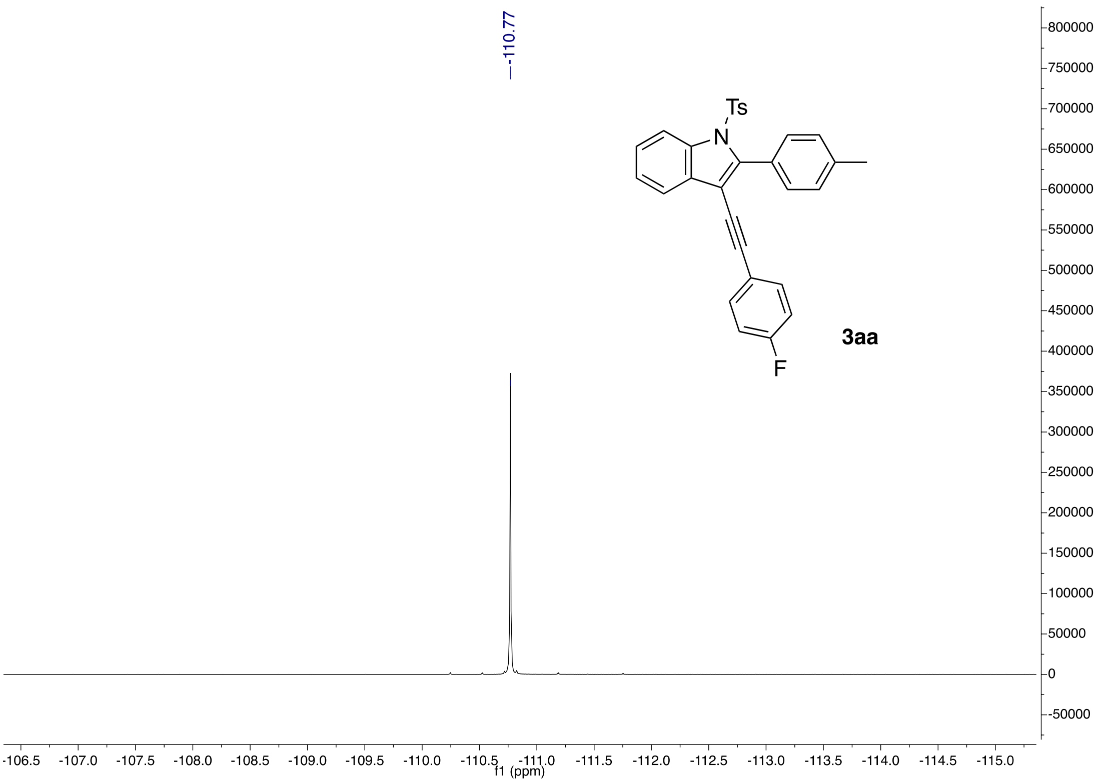

Supplementary Fig. 78 ^{1}H NMR, ^{13}C NMR and ^{19}F NMR spectra of compound **3aa**

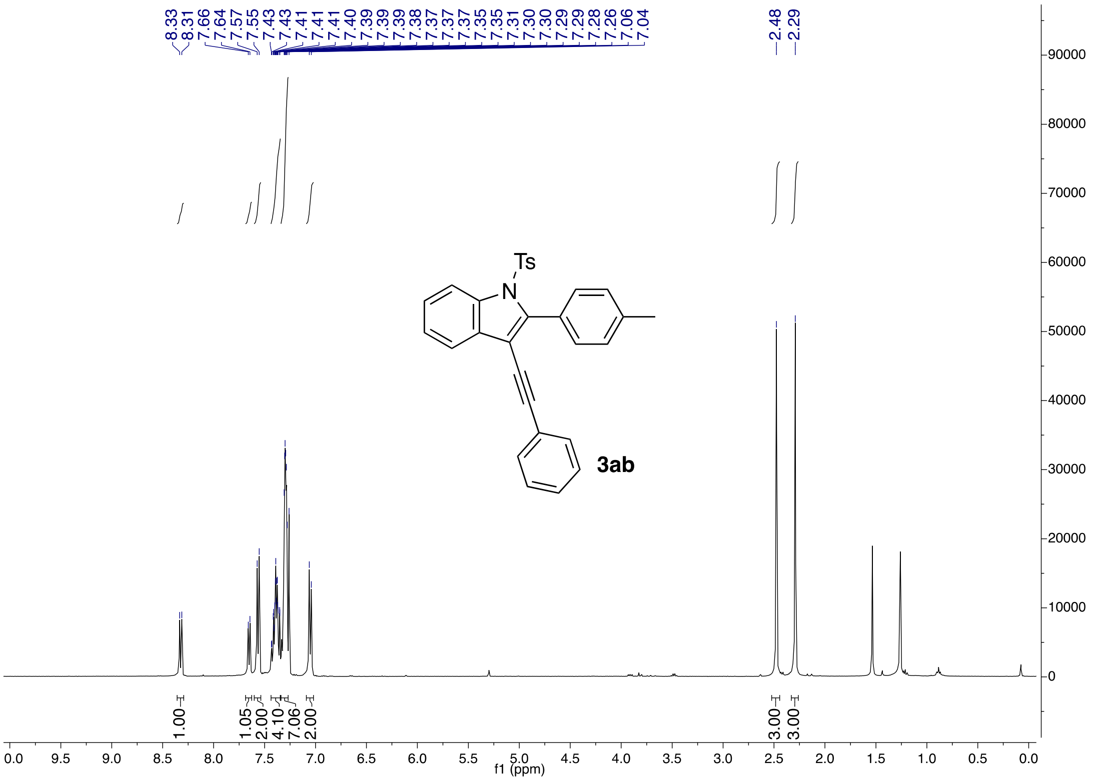

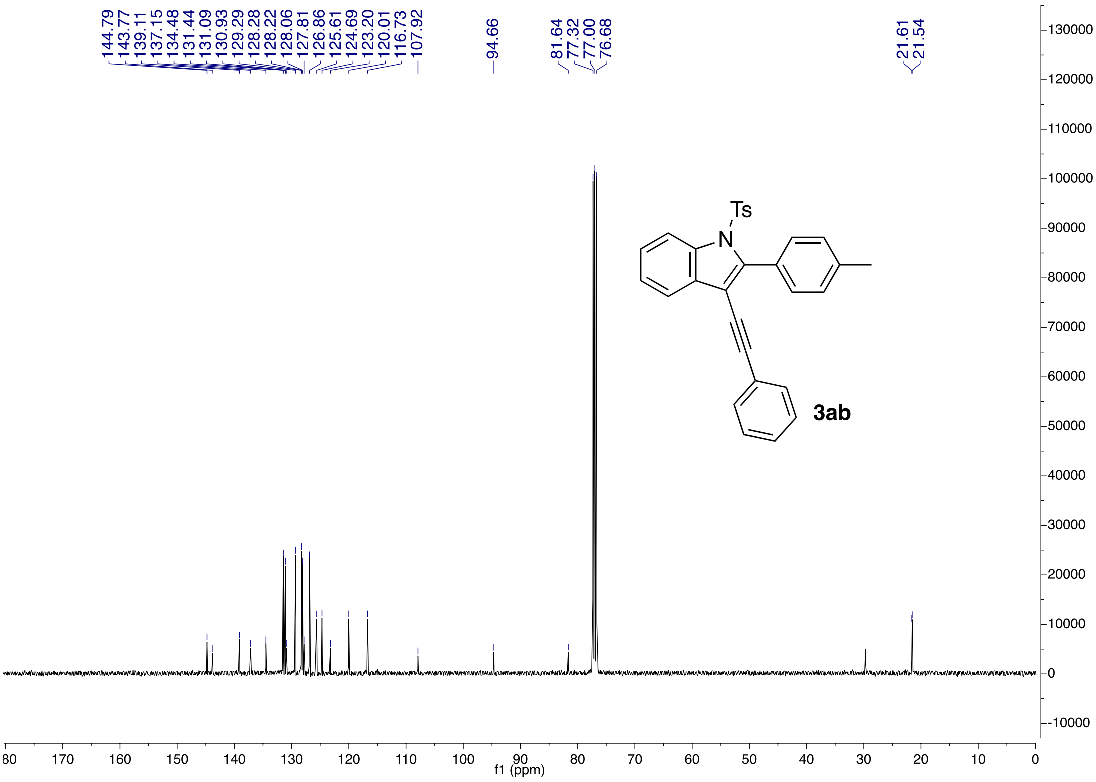

Supplementary Fig. 79 ^{1}H NMR and ^{13}C NMR spectra of compound **3ab**

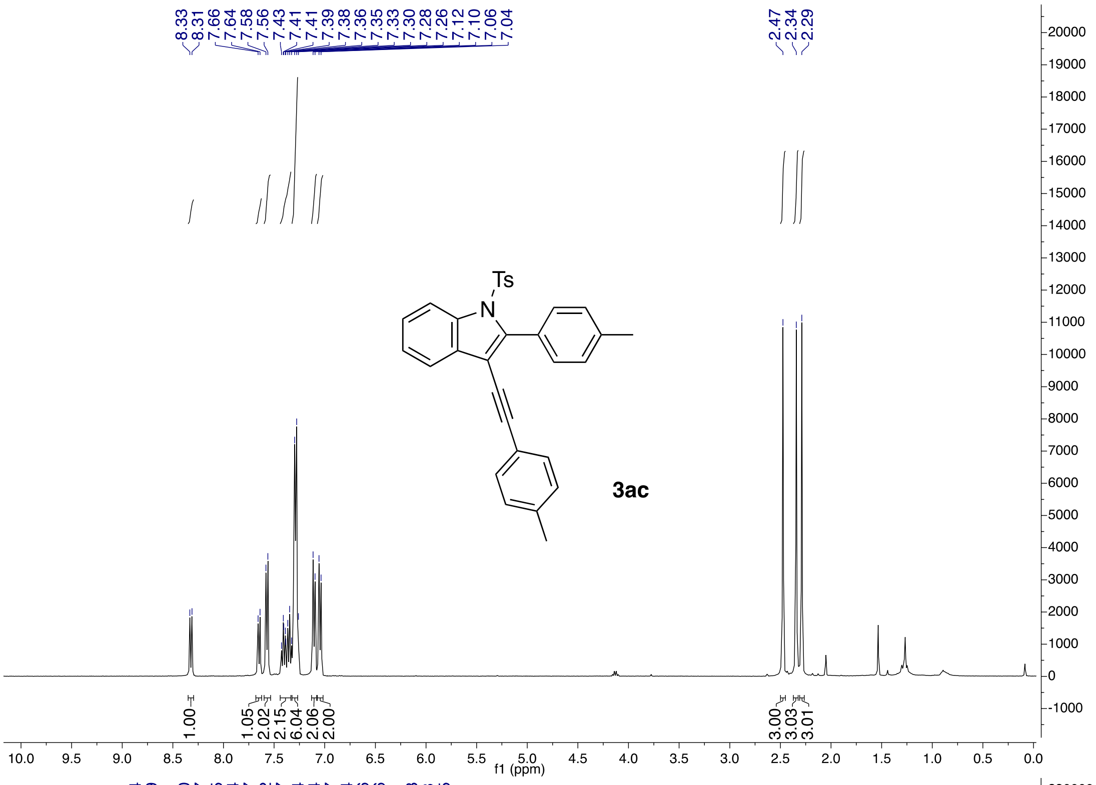

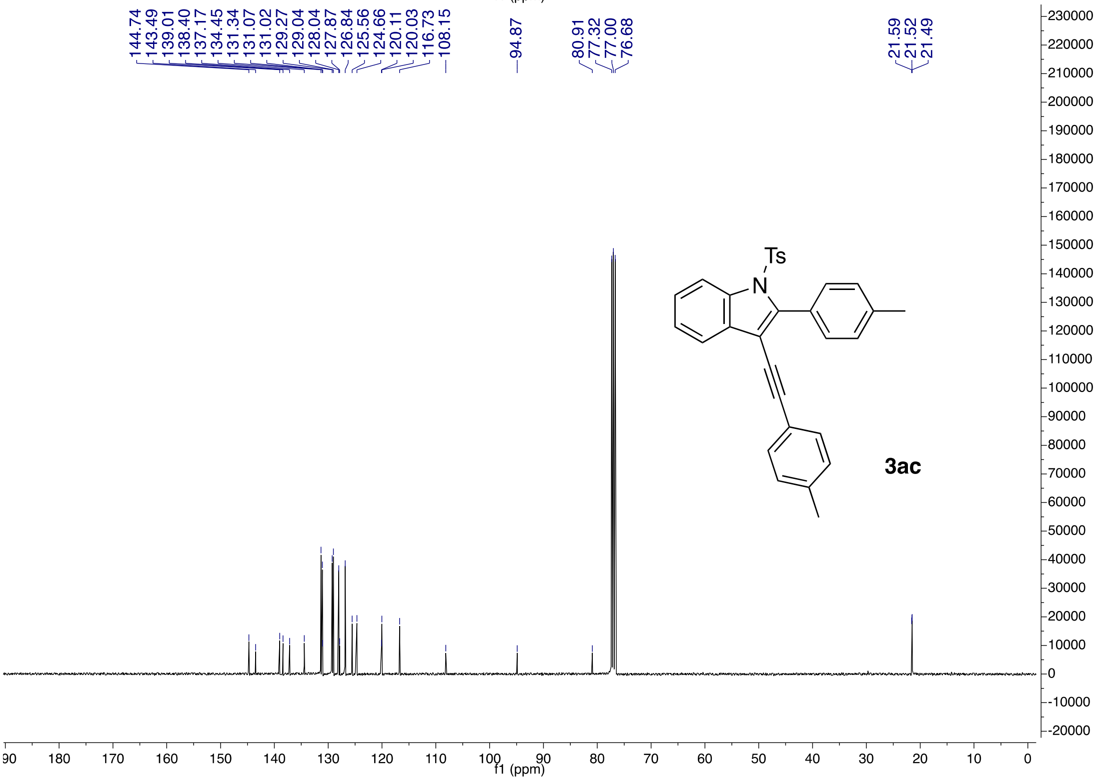

Supplementary Fig. 80 ^{1}H NMR and ^{13}C NMR spectra of compound **3ac**

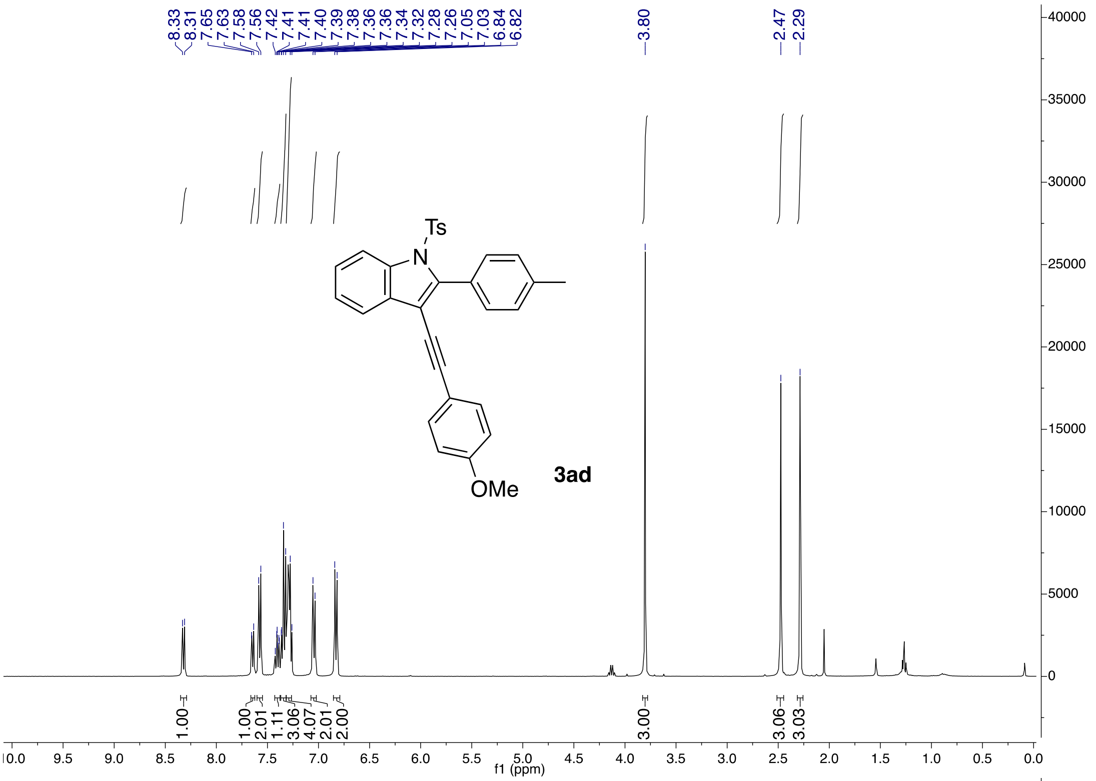

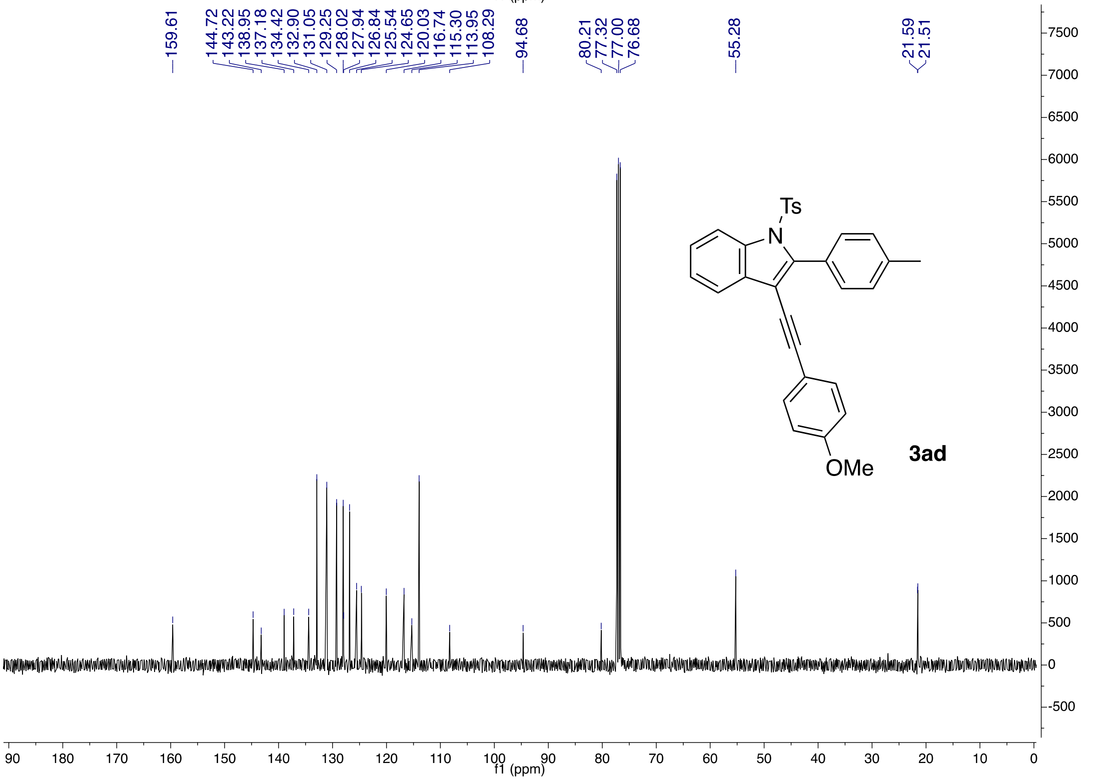

Supplementary Fig. 81 ^{1}H NMR and ^{13}C NMR spectra of compound **3ad**

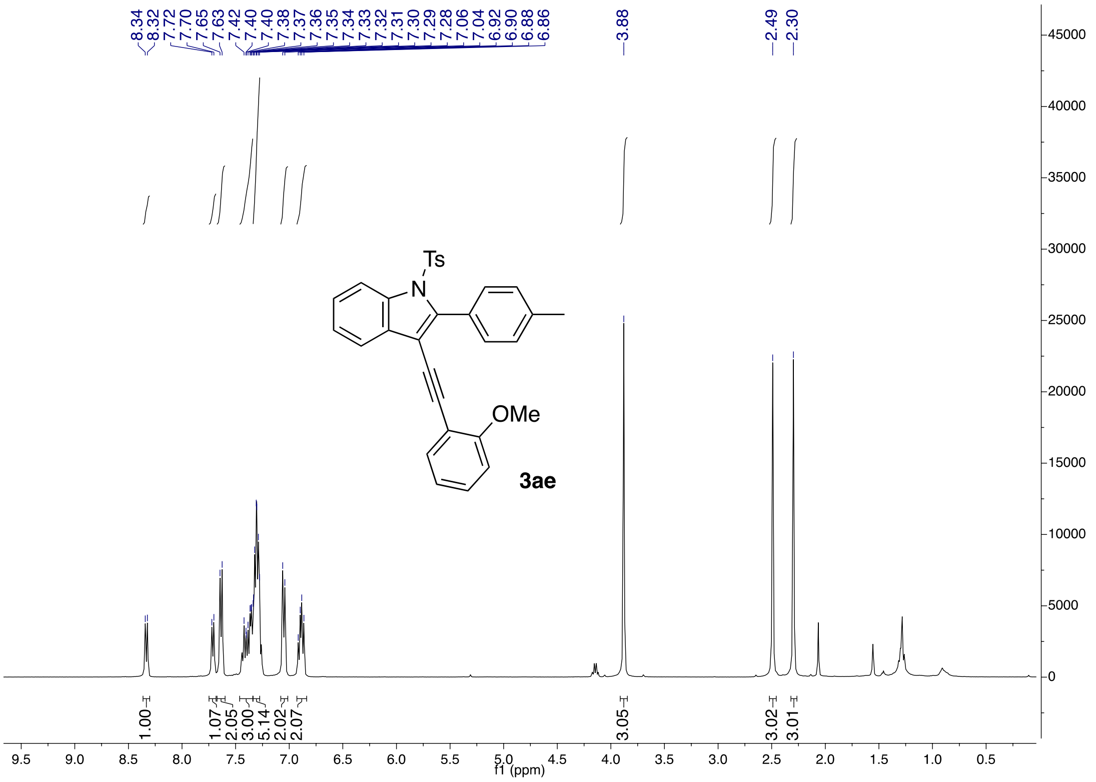

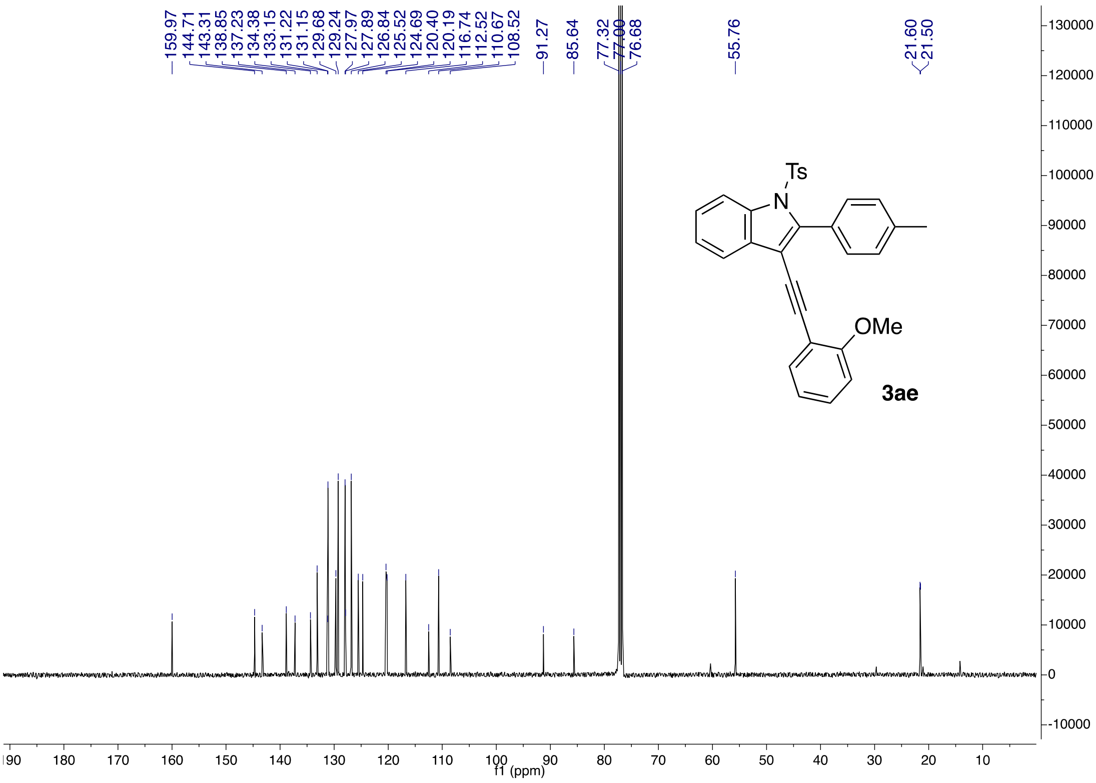

Supplementary Fig. 82 ^{1}H NMR and ^{13}C NMR spectra of compound **3ae**

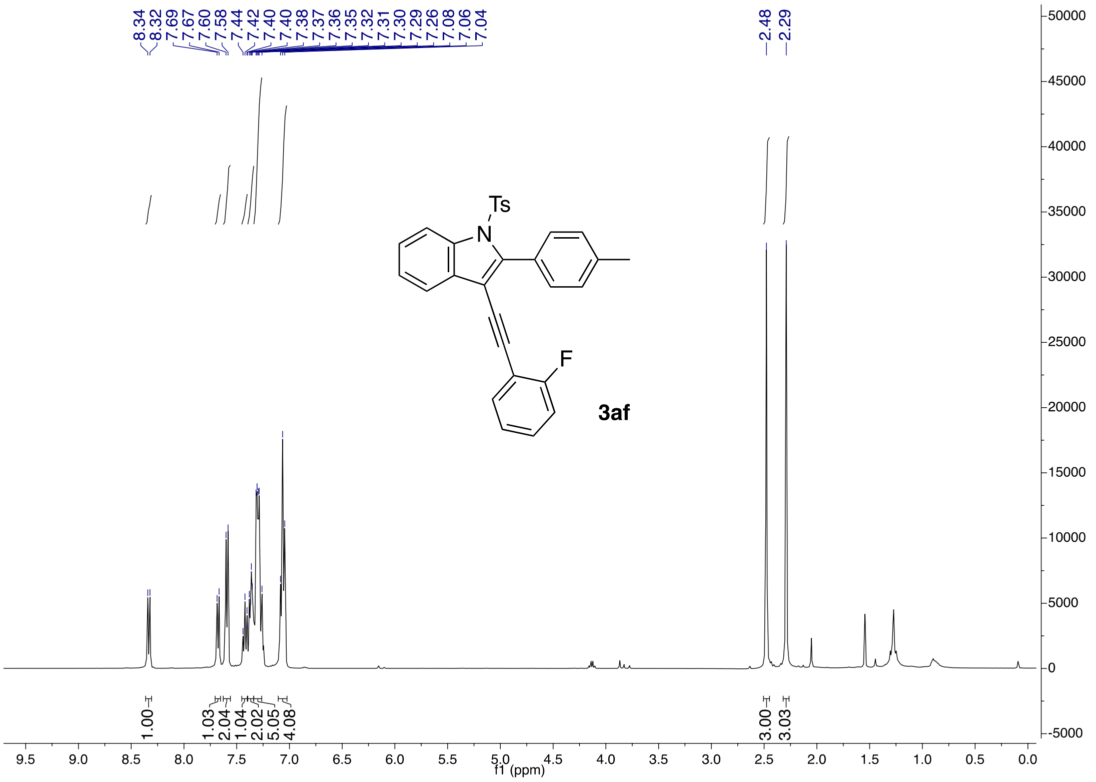
8.34
8.32
7.69
7.67
7.60
7.58
7.44
7.42
7.40
7.38
7.37
7.36
7.35
7.32
7.31
7.30
7.29
7.26
7.08
7.06
7.04
2.48
2.29
1.00
1.03
2.04
1.04
2.02
5.05
4.08
3.00
3.03
Ts
N
F
3af
f1 (ppm)

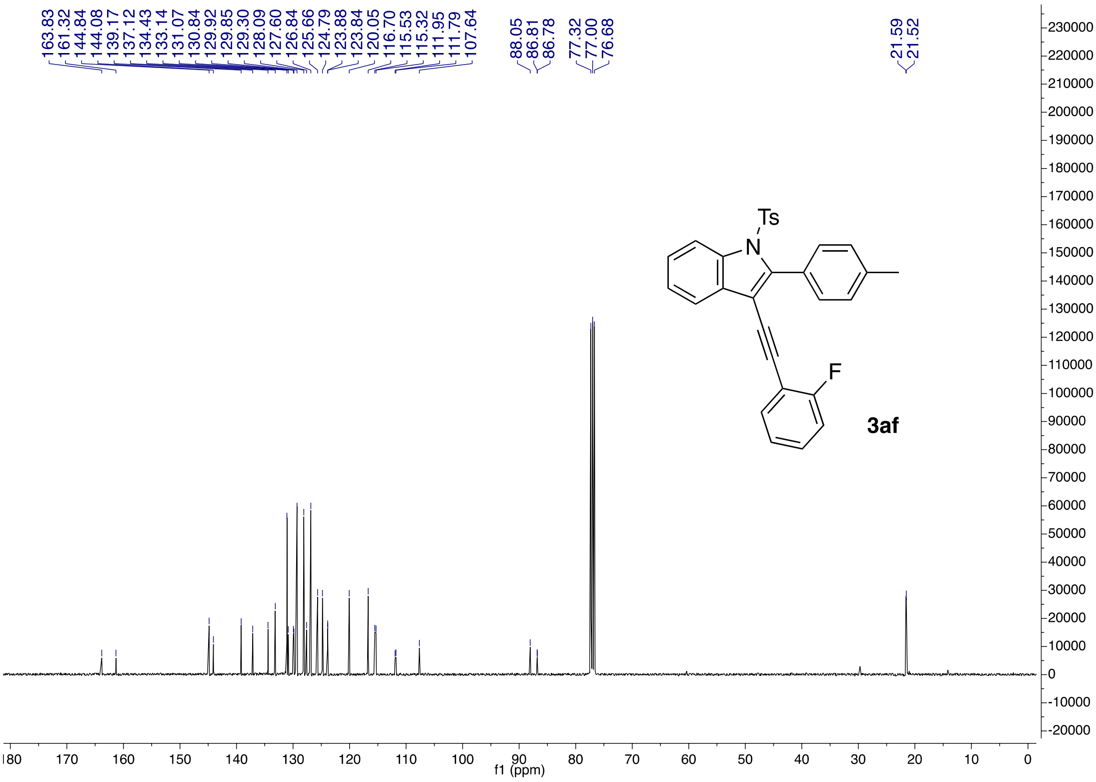
163.83
161.32
144.84
144.08
139.17
137.12
134.43
133.14
131.07
130.84
129.92
129.85
129.30
128.09
127.60
126.84
125.66
124.79
123.88
123.84
120.05
116.70
115.53
115.32
111.95
111.79
107.64
88.05
86.81
86.78
77.32
77.00
76.68
21.59
21.52
Ts
N
F
3af
f1 (ppm)

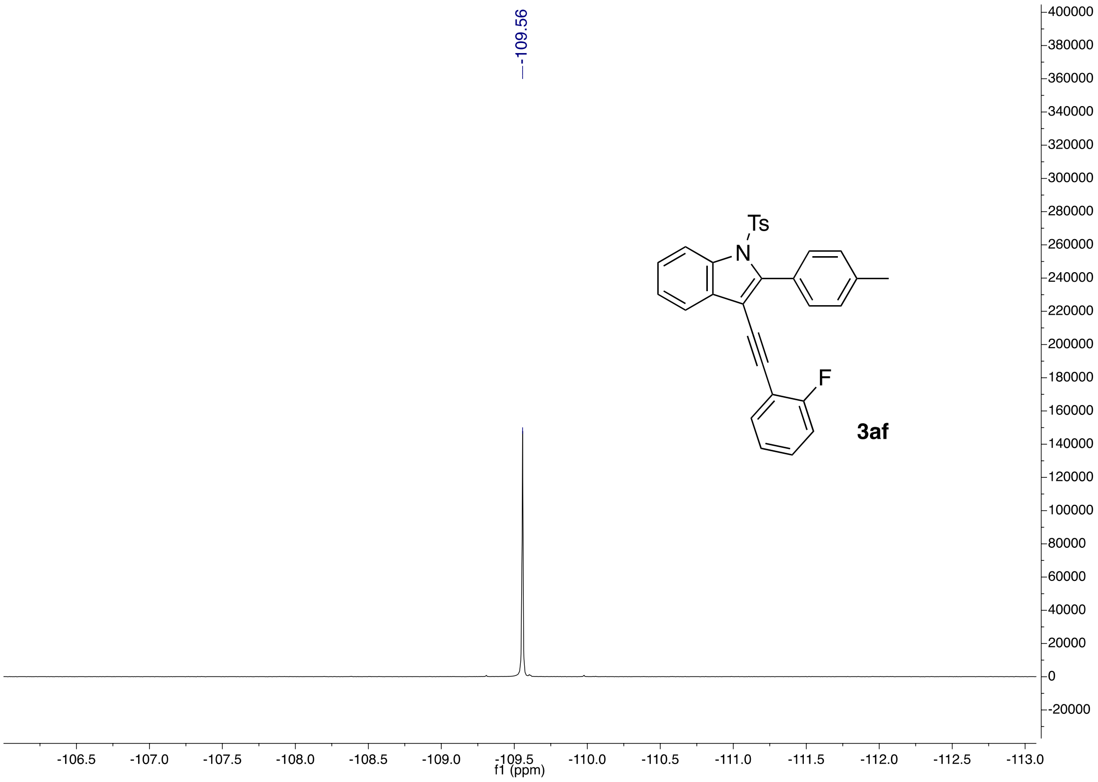

Supplementary Fig. 83 ^{1}H NMR, ^{13}C NMR and ^{19}F NMR spectra of compound **3af**

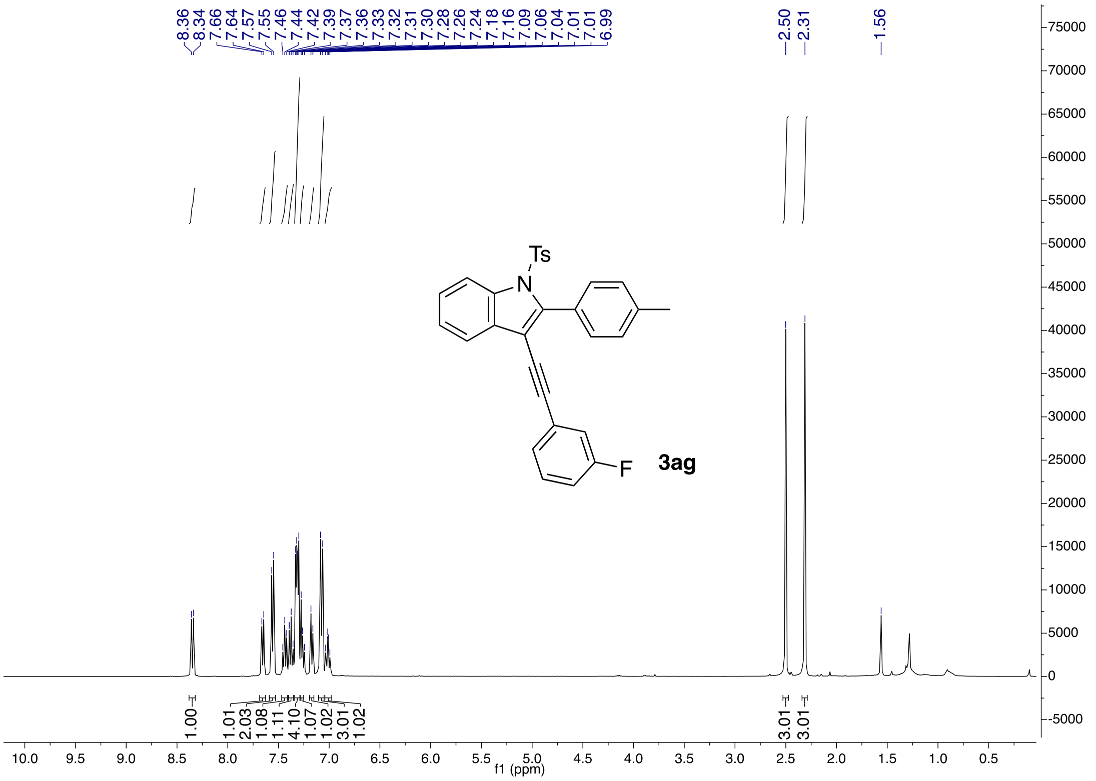
8.36
8.34
7.66
7.64
7.57
7.55
7.46
7.44
7.42
7.39
7.37
7.36
7.33
7.32
7.31
7.30
7.28
7.26
7.24
7.18
7.16
7.09
7.06
7.04
7.01
7.01
6.99
2.50
2.31
1.56
Ts
N
F
3ag
1.00
1.01
2.03
1.08
1.11
4.10
1.07
1.02
3.01
1.02
3.01
3.01
f1 (ppm)

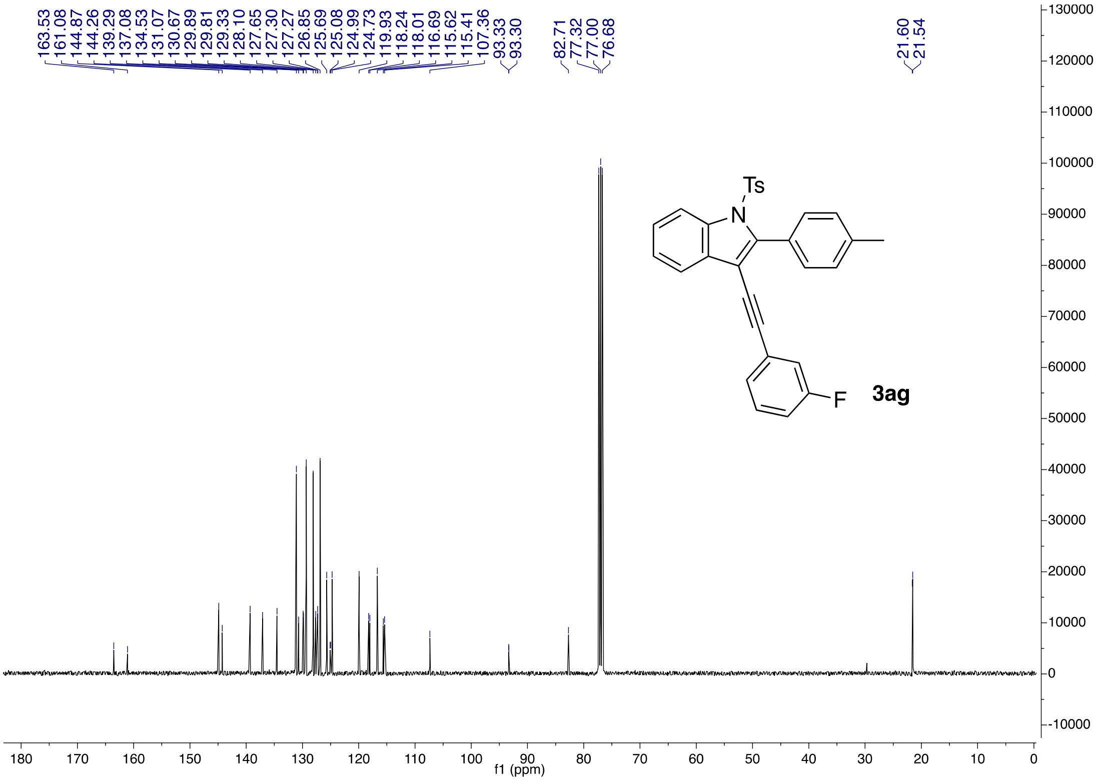
163.53
161.08
144.87
144.26
139.29
137.08
134.53
131.07
130.67
129.89
129.81
129.33
128.10
127.65
127.30
127.27
126.85
125.69
125.08
124.99
124.73
119.93
118.24
118.01
116.69
115.62
115.41
107.36
93.33
93.30
82.71
77.32
77.00
76.68
21.60
21.54
Ts
N
F
3ag
f1 (ppm)

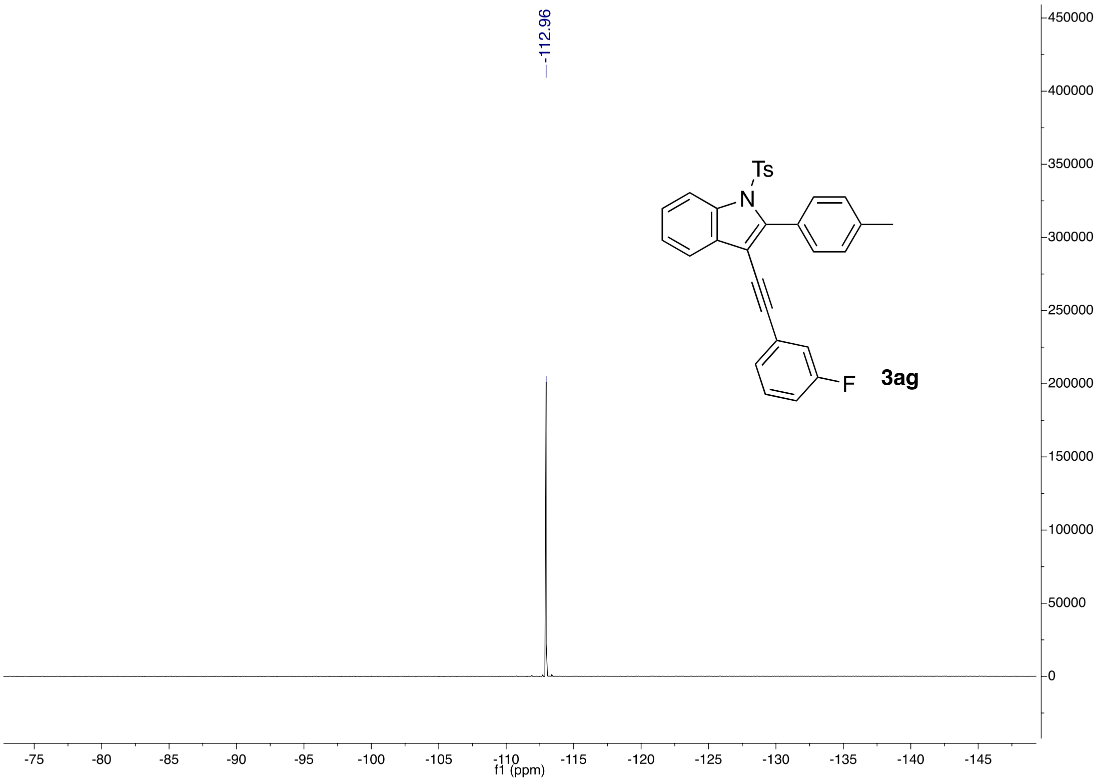

Supplementary Fig. 84 ^{1}H NMR, ^{13}C NMR and ^{19}F NMR spectra of compound **3ag**

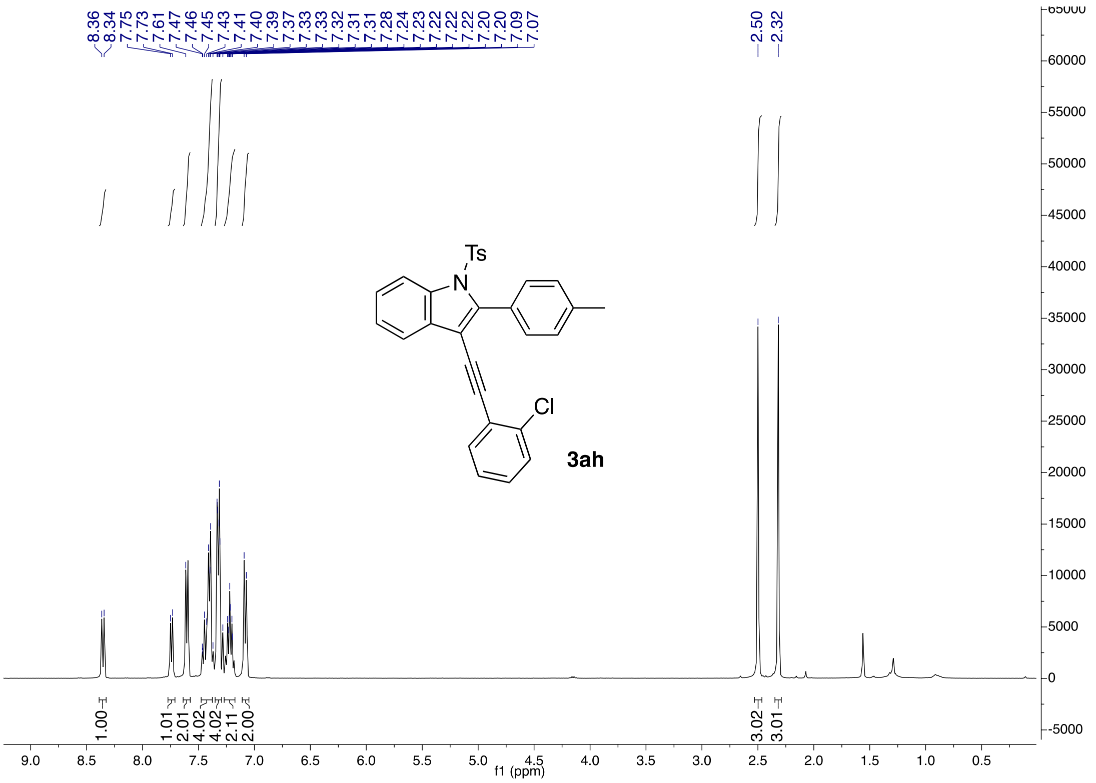

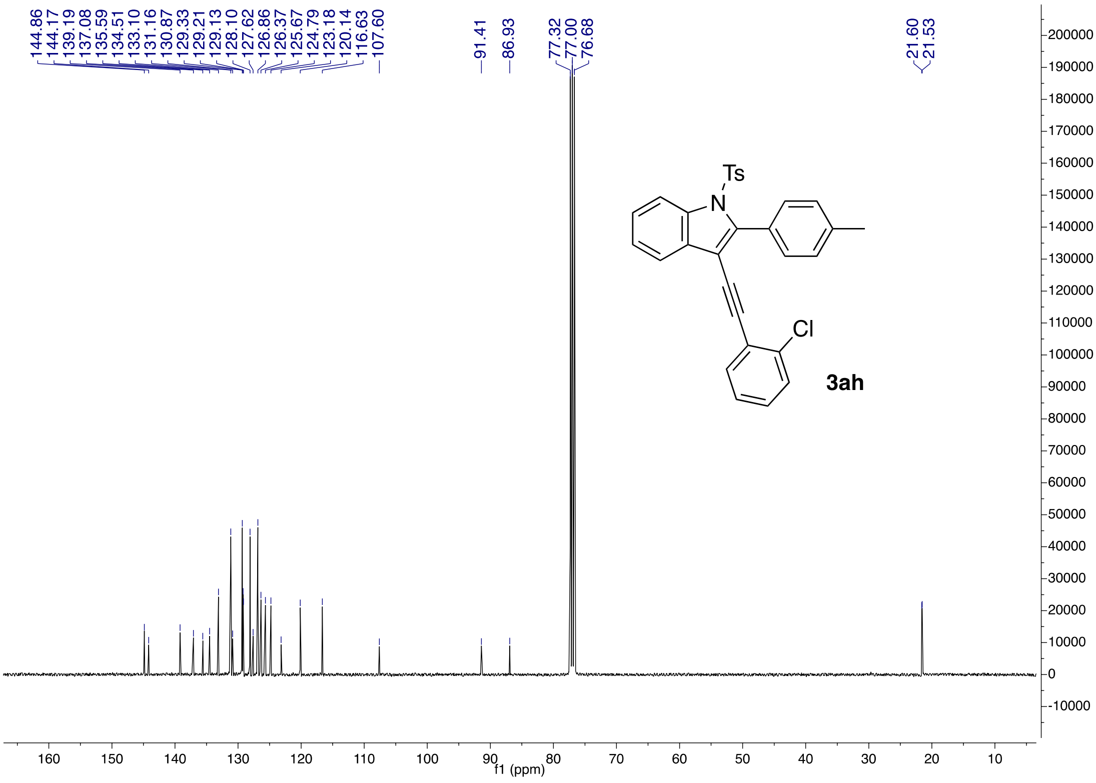

Supplementary Fig. 85 ^{1}H NMR and ^{13}C NMR spectra of compound **3ah**

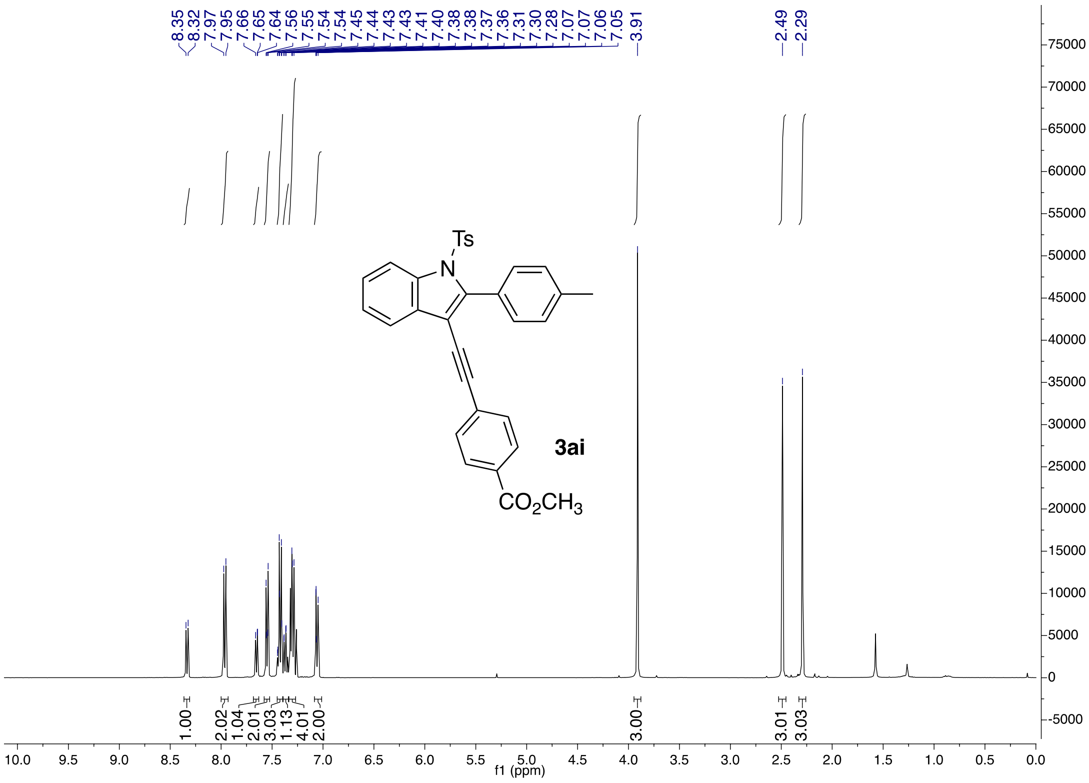

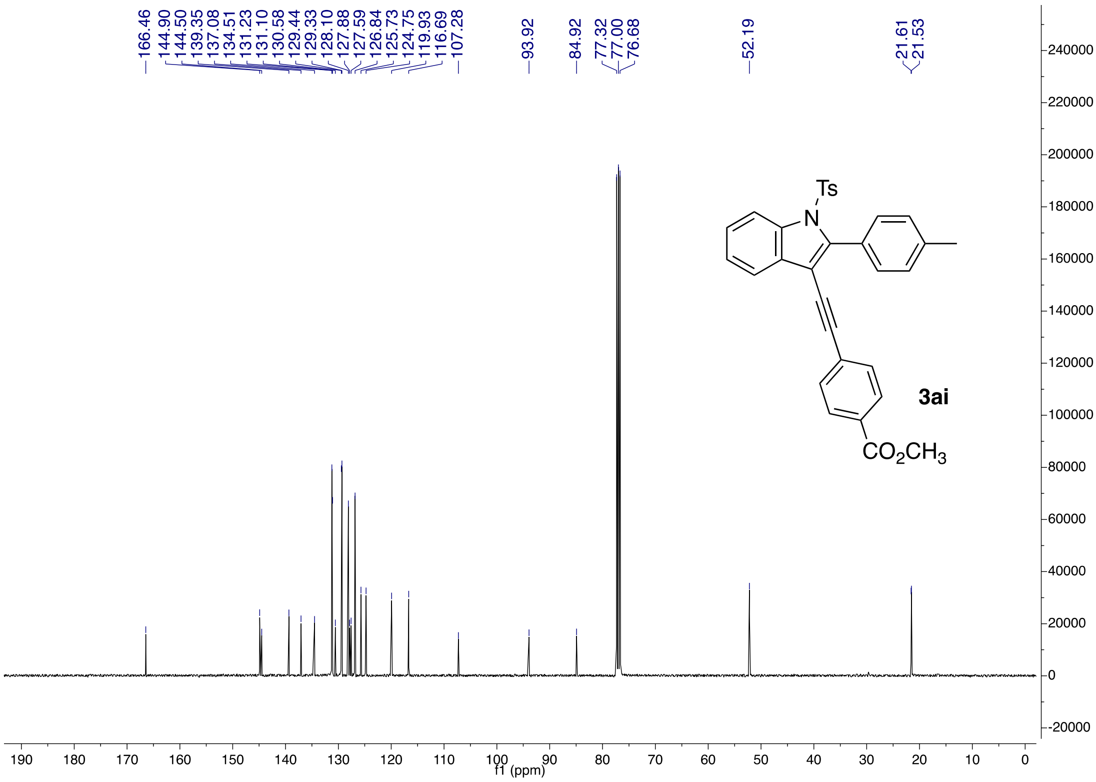

Supplementary Fig. 86 ^{1}H NMR and ^{13}C NMR spectra of compound **3ai**

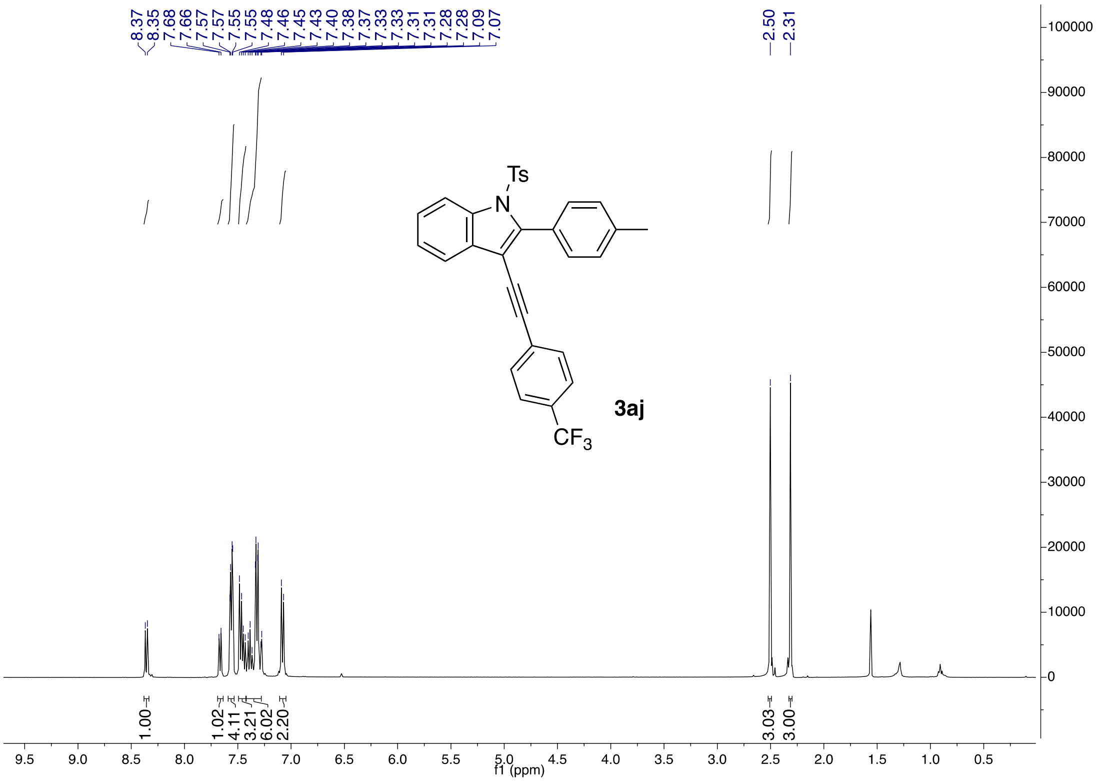

8.37
8.35
7.68
7.66
7.57
7.57
7.55
7.55
7.48
7.46
7.45
7.43
7.40
7.38
7.37
7.33
7.33
7.31
7.31
7.28
7.09
7.07
2.50
2.31
1.00
1.02
4.11
3.21
6.02
2.20
3.03
3.00
Ts
N
3aj
CF3
f1 (ppm)

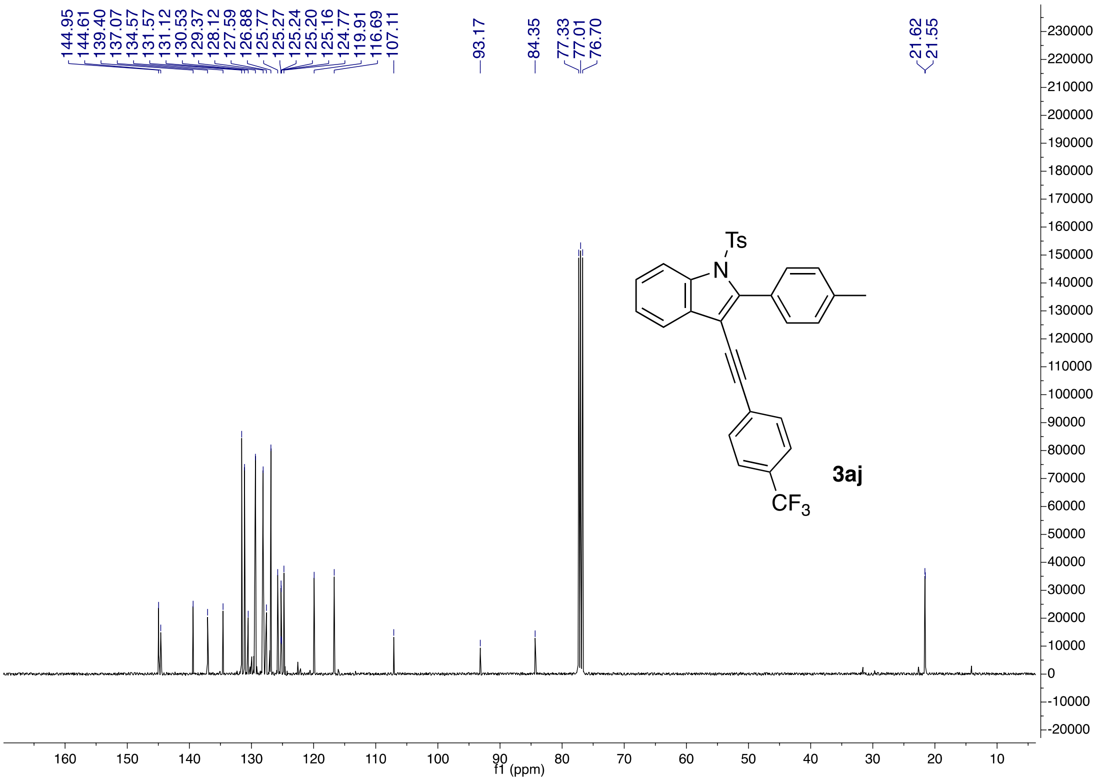

144.95
144.61
139.40
137.07
134.57
131.57
131.12
130.53
129.37
128.12
127.59
126.88
125.77
125.27
125.24
125.20
125.16
124.77
119.91
116.69
107.11
93.17
84.35
77.33
77.01
76.70
21.62
21.55
Ts
N
3aj
CF3
f1 (ppm)

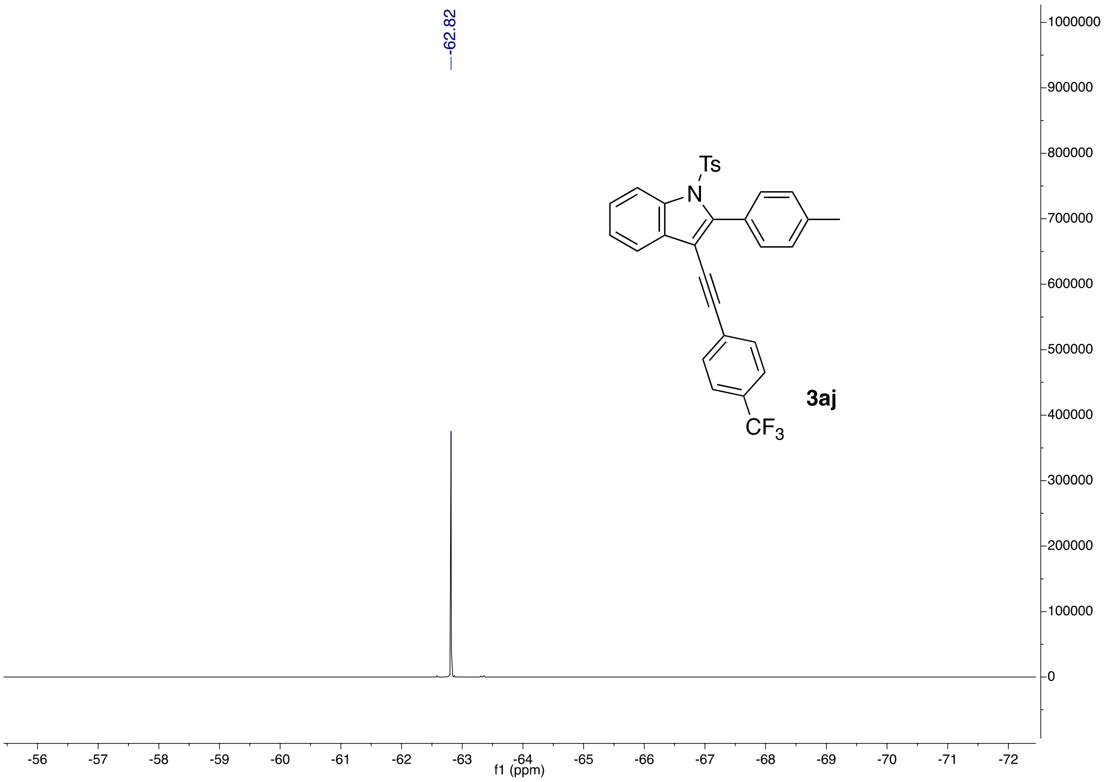

Supplementary Fig. 87 ^{1}H NMR, ^{13}C NMR and ^{19}F NMR spectra of compound **3aj**

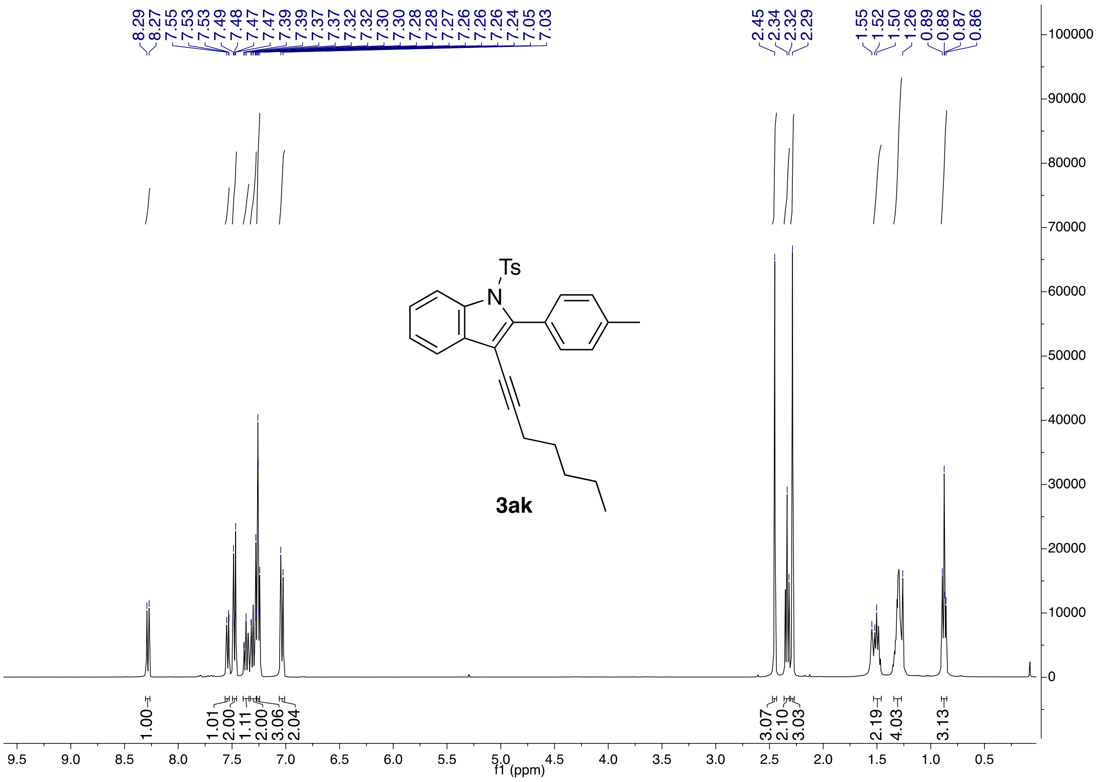

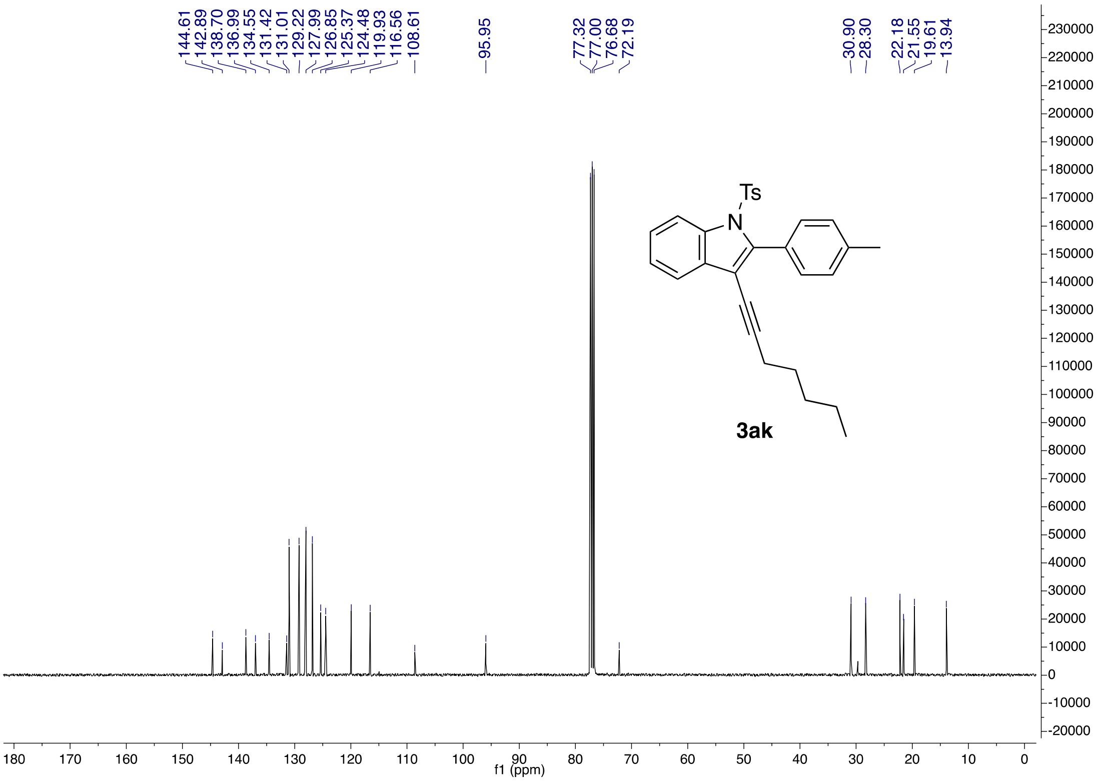

Supplementary Fig. 88 ^{1}H NMR and ^{13}C NMR spectra of compound **3ak**

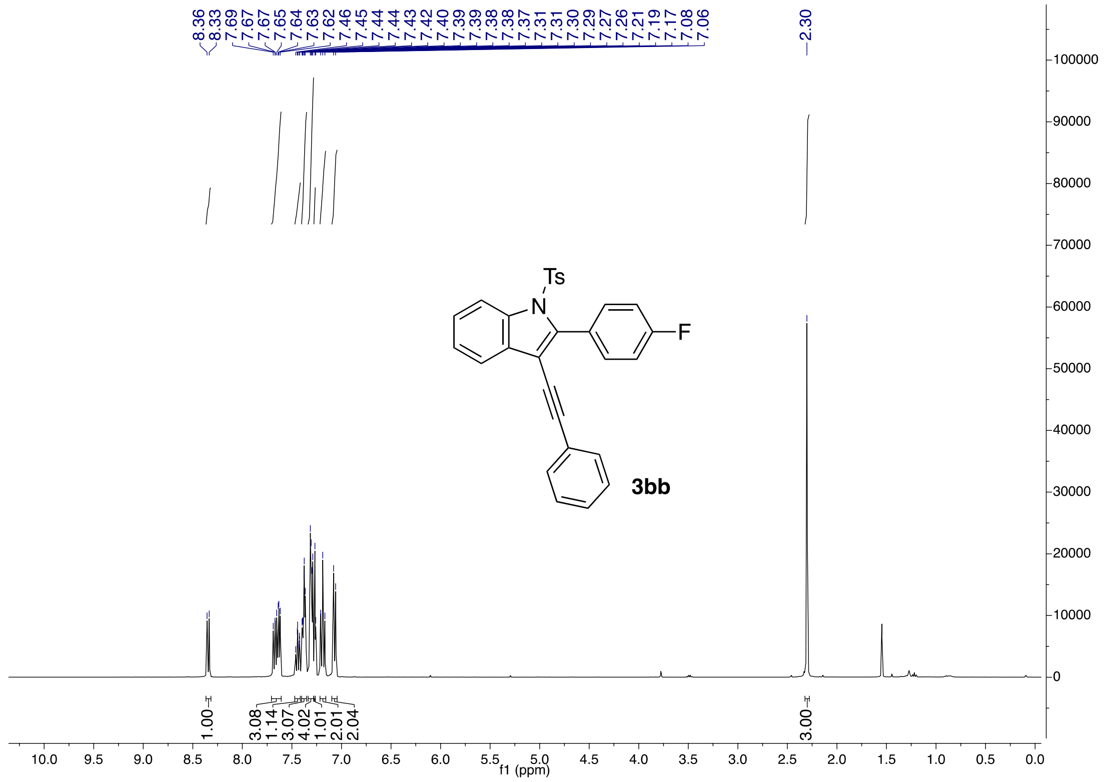

8.36
8.33
7.69
7.67
7.65
7.64
7.63
7.62
7.46
7.45
7.44
7.44
7.43
7.42
7.40
7.39
7.39
7.38
7.38
7.37
7.31
7.31
7.30
7.29
7.27
7.26
7.21
7.19
7.17
7.08
7.06
2.30
Ts
N
F
3bb
1.00
3.08
1.14
3.07
4.02
1.01
2.01
2.04
3.00
f1 (ppm)

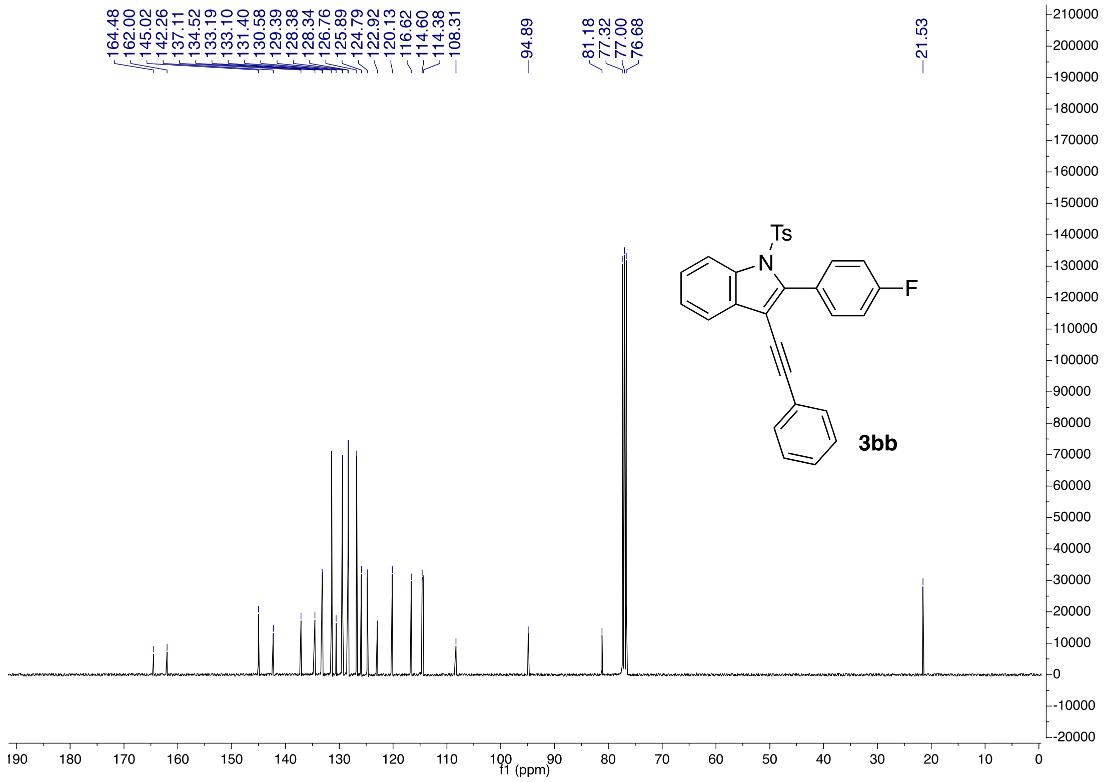

164.48
162.00
145.02
142.26
137.11
134.52
133.19
133.10
131.40
130.58
129.39
128.38
128.34
126.76
125.89
124.79
122.92
120.13
116.62
114.60
114.38
108.31
94.89
81.18
77.32
77.00
76.68
21.53
Ts
N
F
3bb
f1 (ppm)

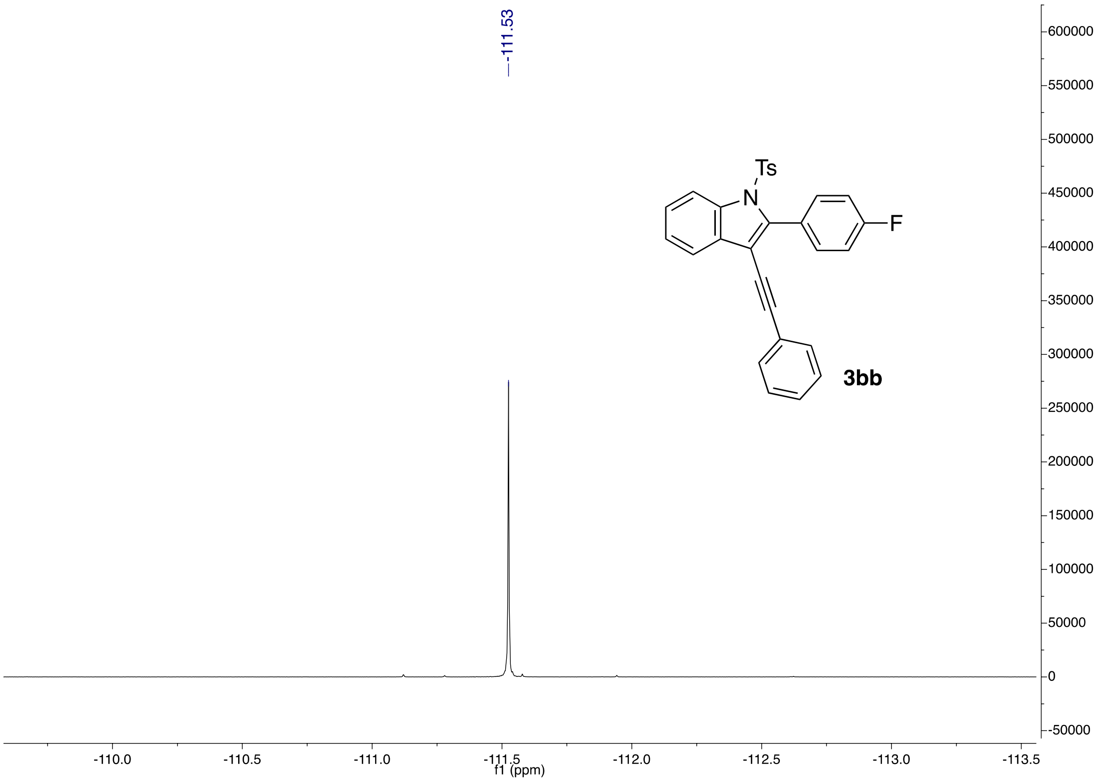

Supplementary Fig. 89 ^{1}H NMR, ^{13}C NMR and ^{19}F NMR spectra of compound **3bb**

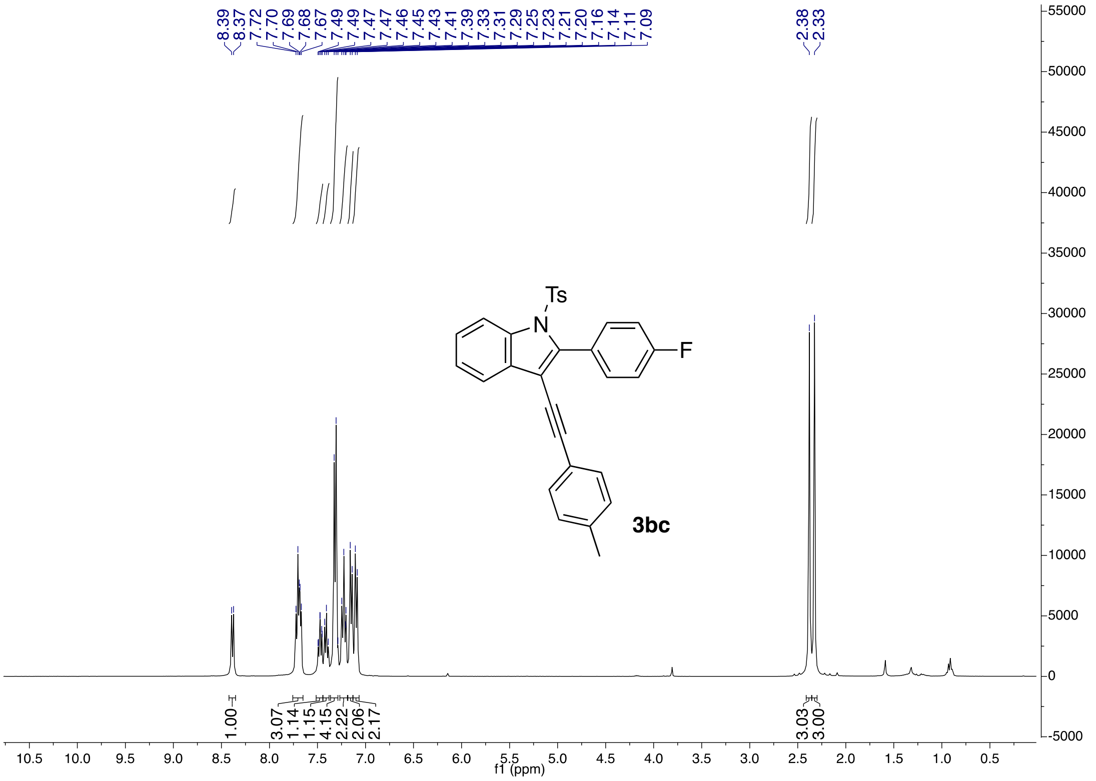
8.39
8.37
7.72
7.70
7.69
7.68
7.67
7.49
7.47
7.46
7.45
7.43
7.41
7.39
7.33
7.31
7.29
7.25
7.23
7.21
7.20
7.16
7.14
7.11
7.09
2.38
2.33
Ts
N
F
3bc
1.00
3.07
1.14
1.15
4.15
2.22
2.06
2.17
3.03
3.00
f1 (ppm)

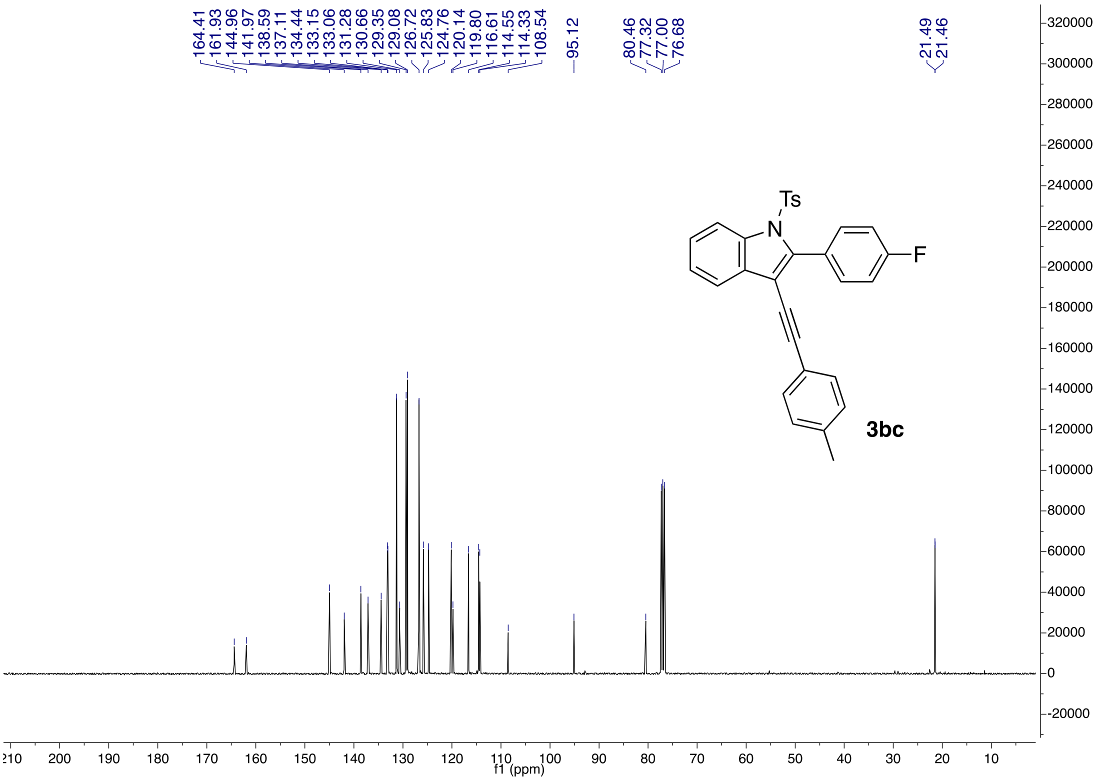
164.41
161.93
144.96
141.97
138.59
137.11
134.44
133.15
133.06
131.28
130.66
129.35
129.08
126.72
125.83
124.76
120.14
119.80
116.61
114.55
114.33
108.54
95.12
80.46
77.32
77.00
76.68
21.49
21.46
Ts
N
F
3bc
f1 (ppm)

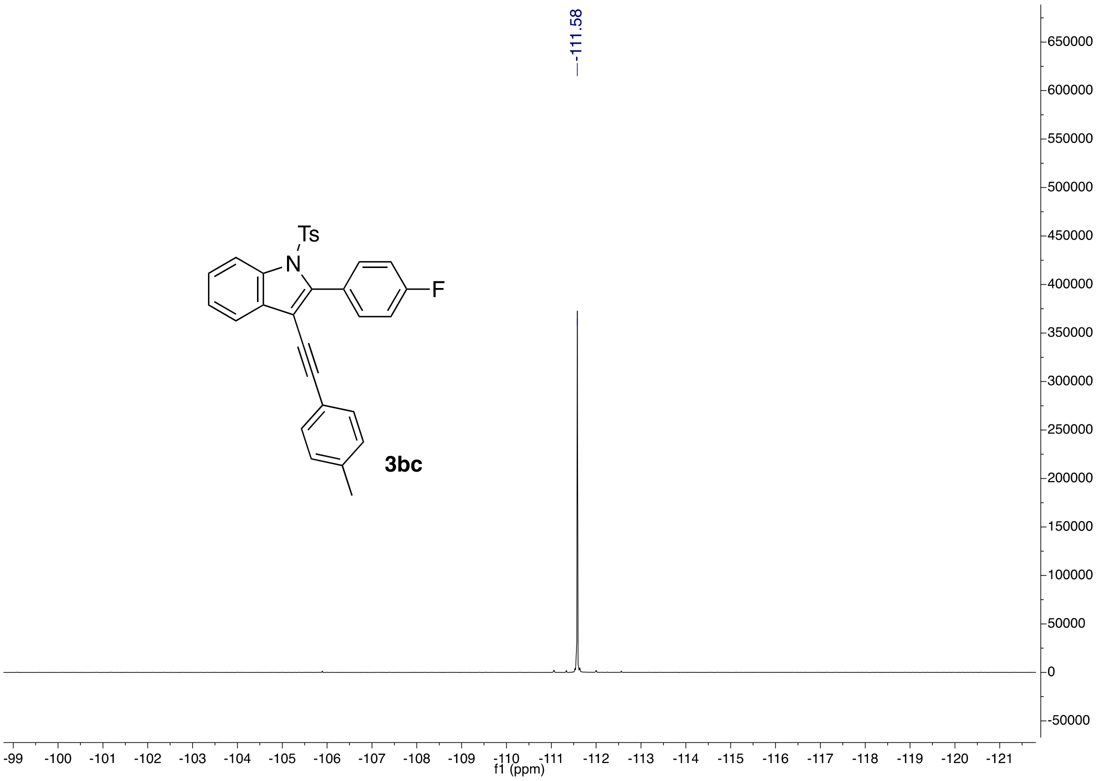

Supplementary Fig. 90 ^{1}H NMR, ^{13}C NMR and ^{19}F NMR spectra of compound **3bc**

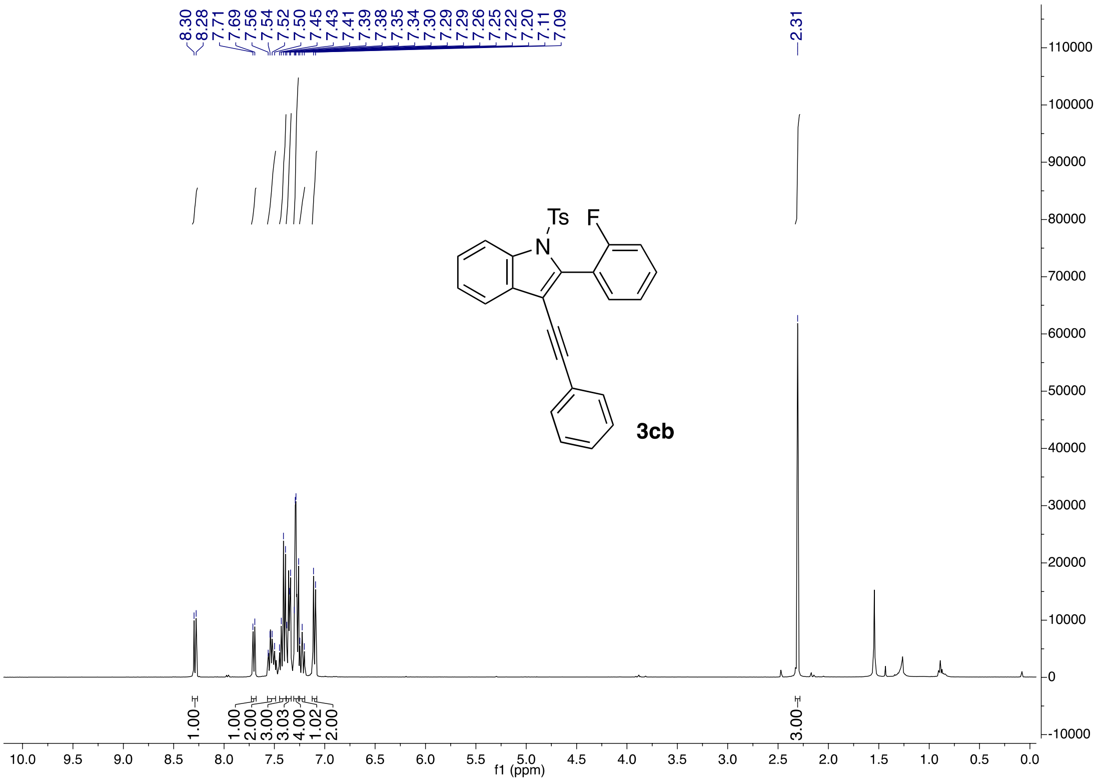

8.30
8.28
7.71
7.69
7.56
7.54
7.52
7.50
7.45
7.43
7.41
7.39
7.38
7.35
7.34
7.30
7.29
7.29
7.26
7.25
7.22
7.20
7.11
7.09
2.31
Ts
N
F
3cb
1.00
1.00
2.00
3.00
3.03
4.00
1.02
2.00
3.00
f1 (ppm)

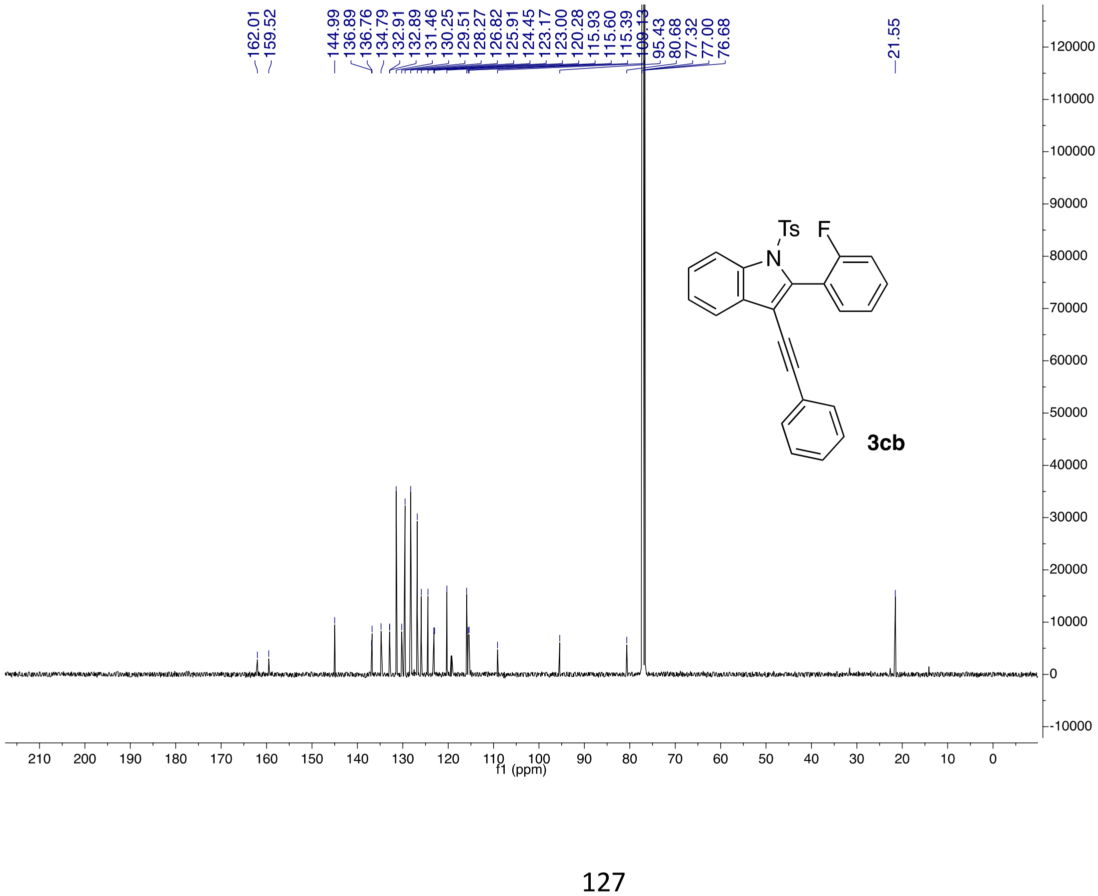

162.01
159.52
144.99
136.89
136.76
134.79
132.91
132.89
131.46
130.25
129.51
128.27
126.82
125.91
124.45
123.17
123.00
120.28
115.93
115.60
115.39
95.43
80.68
77.32
77.00
76.68
21.55
Ts
N
F
3cb
f1 (ppm)

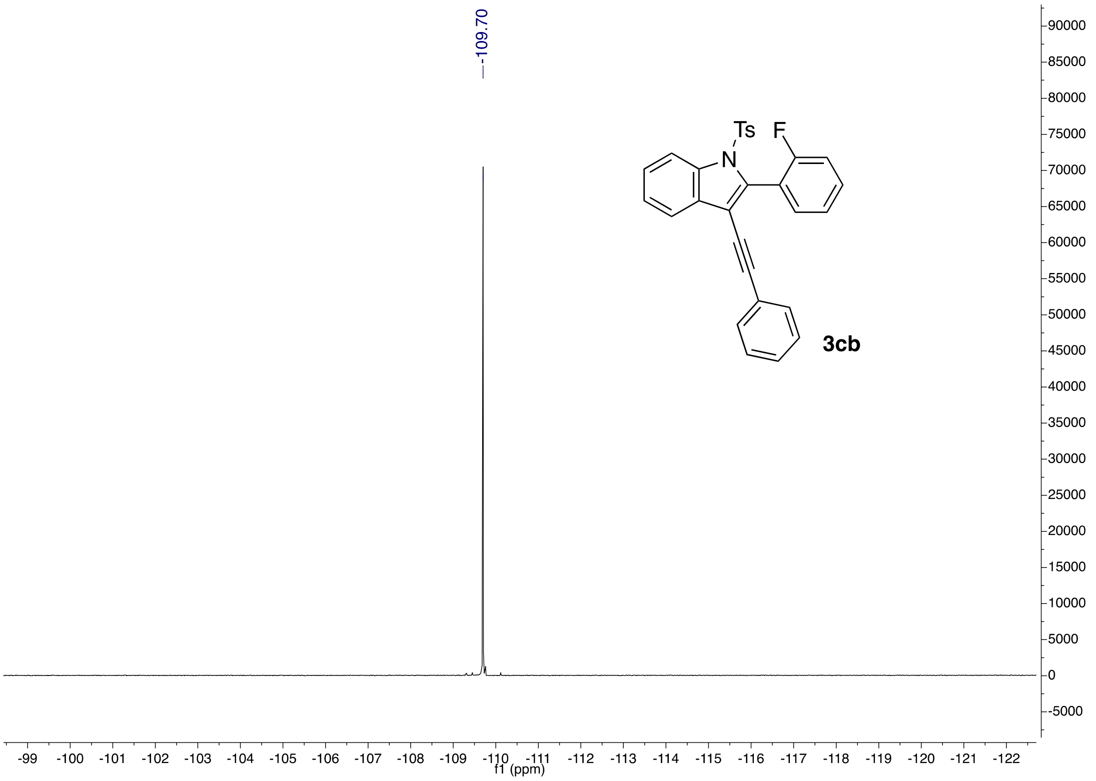

Supplementary Fig. 91 ^{1}H NMR, ^{13}C NMR and ^{19}F NMR spectra of compound **3cb**

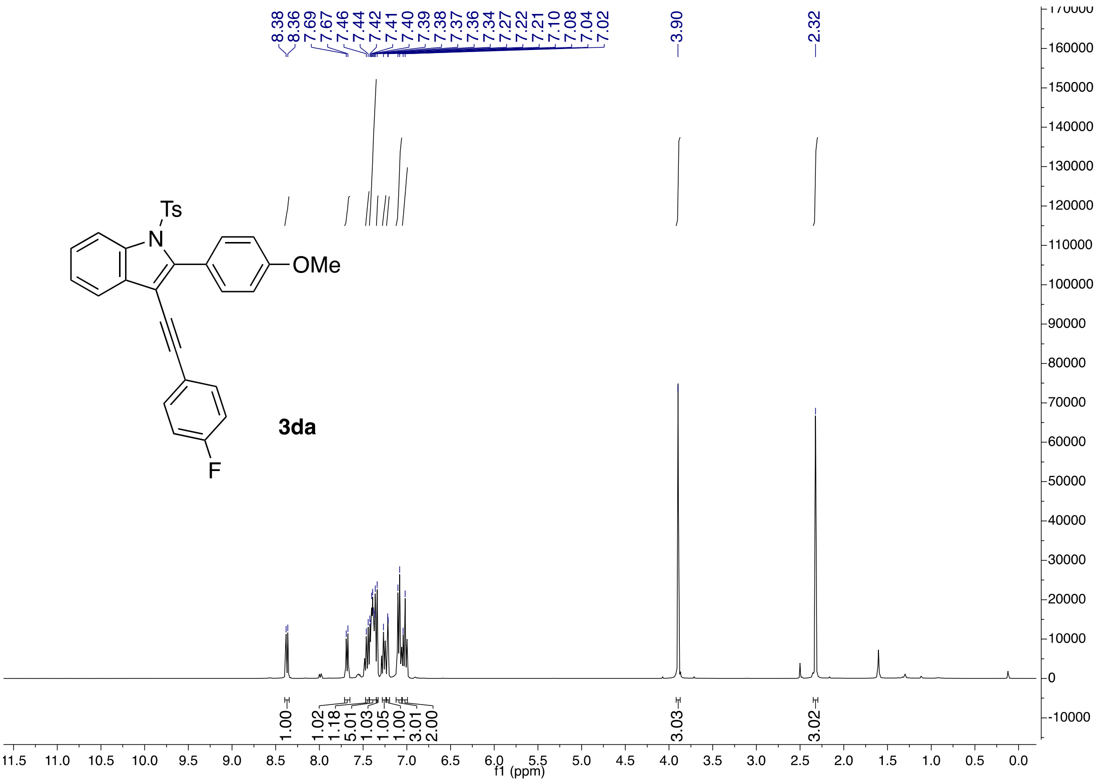
8.38
8.36
7.69
7.67
7.46
7.44
7.42
7.41
7.40
7.39
7.38
7.37
7.36
7.34
7.27
7.22
7.21
7.10
7.08
7.04
7.02
3.90
2.32
Ts
N
OMe
F
3da
1.00
1.02
1.18
5.01
1.03
1.05
1.00
3.01
2.00
3.03
3.02
f1 (ppm)

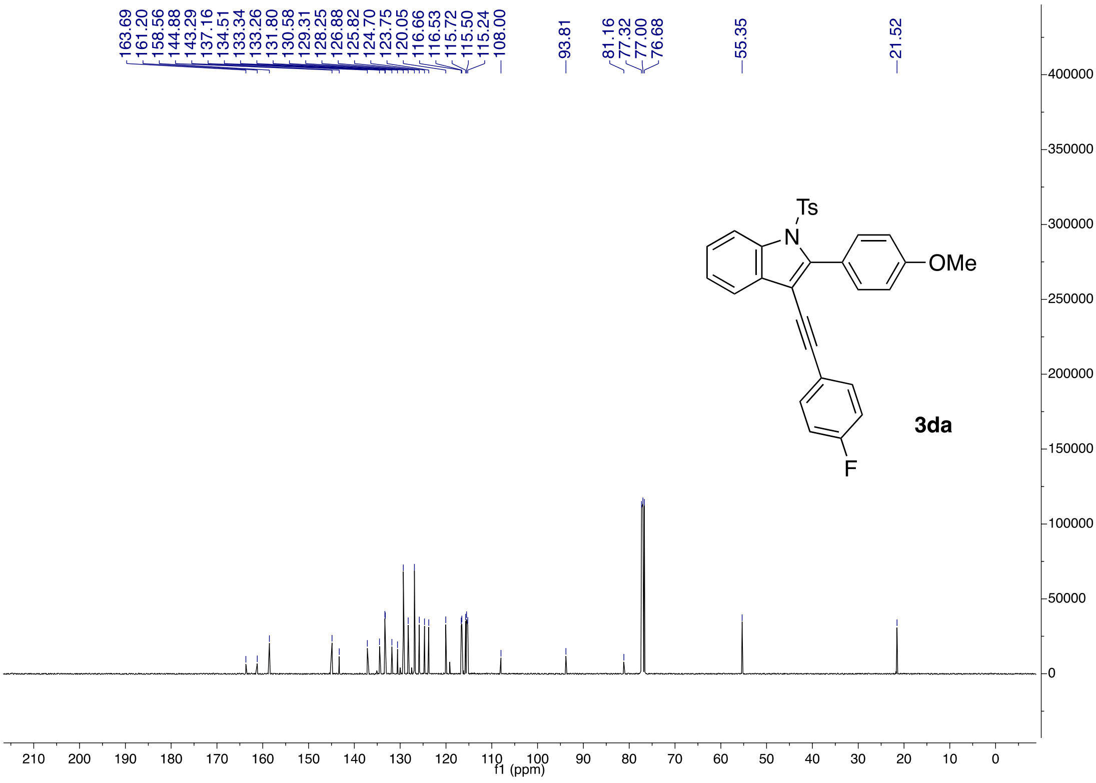
163.69
161.20
158.56
144.88
143.29
137.16
134.51
133.34
133.26
131.80
130.58
129.31
128.25
126.88
125.82
124.70
123.75
120.05
116.66
116.53
115.72
115.50
115.24
108.00
93.81
81.16
77.32
77.00
76.68
55.35
21.52
Ts
N
OMe
F
3da
f1 (ppm)

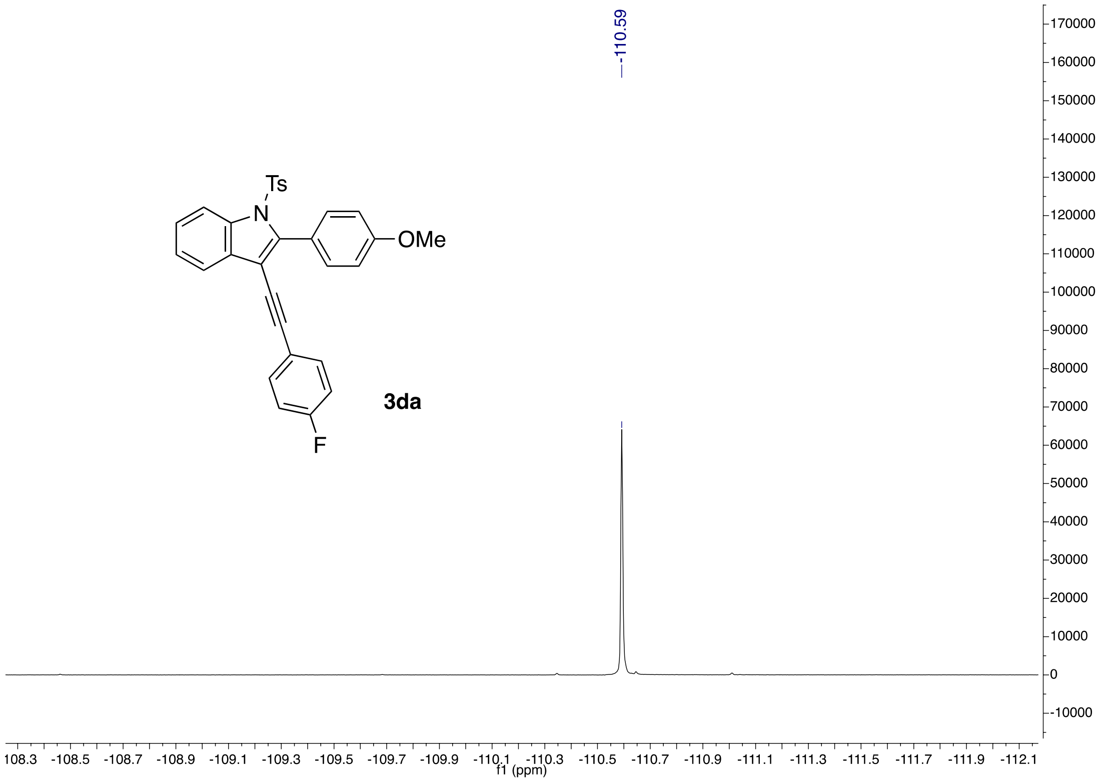

Supplementary Fig. 92 ^{1}H NMR, ^{13}C NMR and ^{19}F NMR spectra of compound **3da**

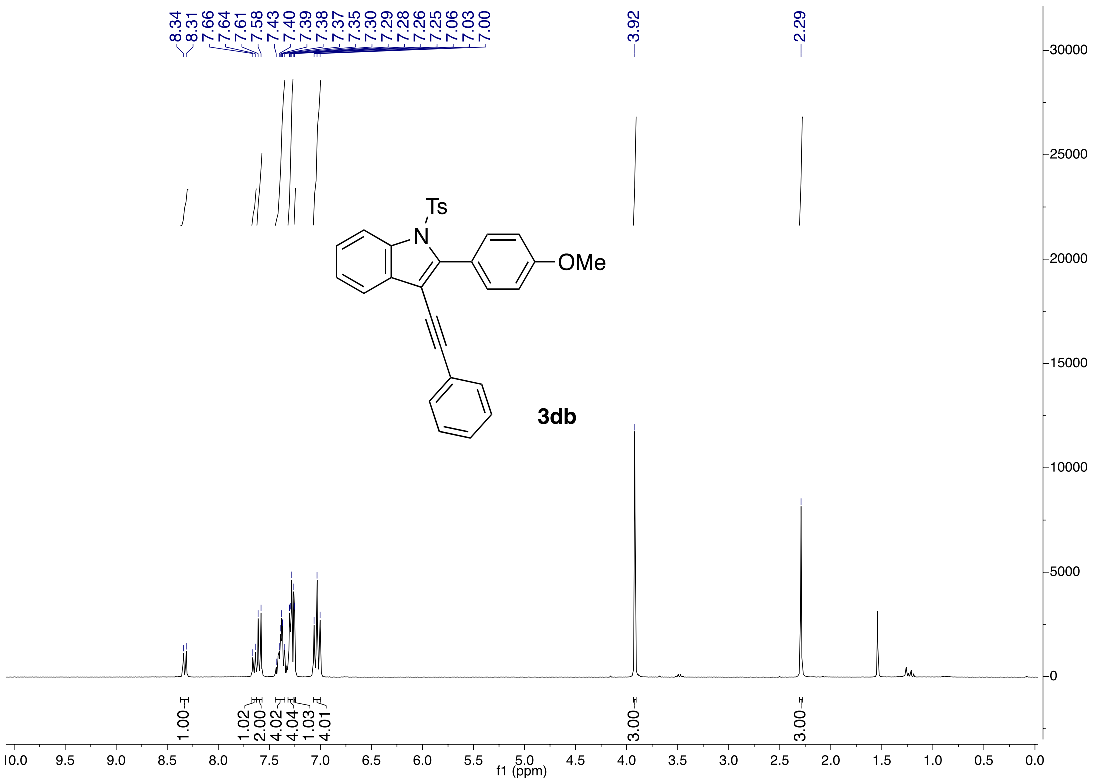

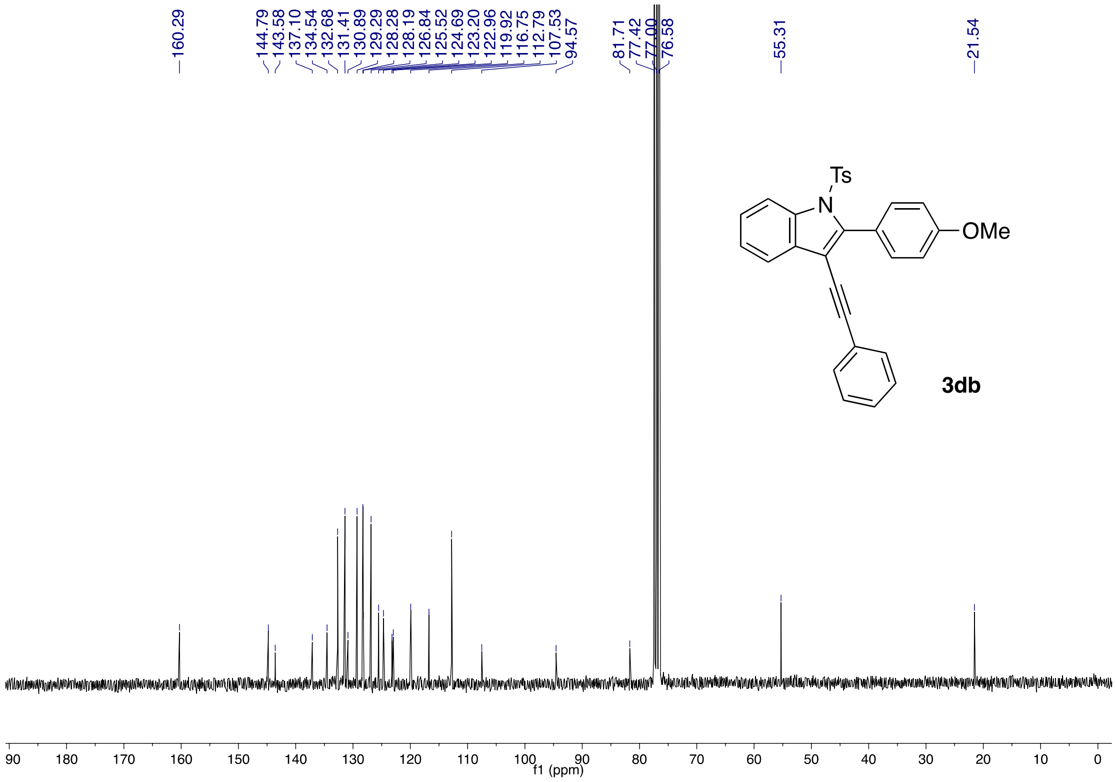

Supplementary Fig. 93 ^{1}H NMR and ^{13}C NMR spectra of compound **3db**

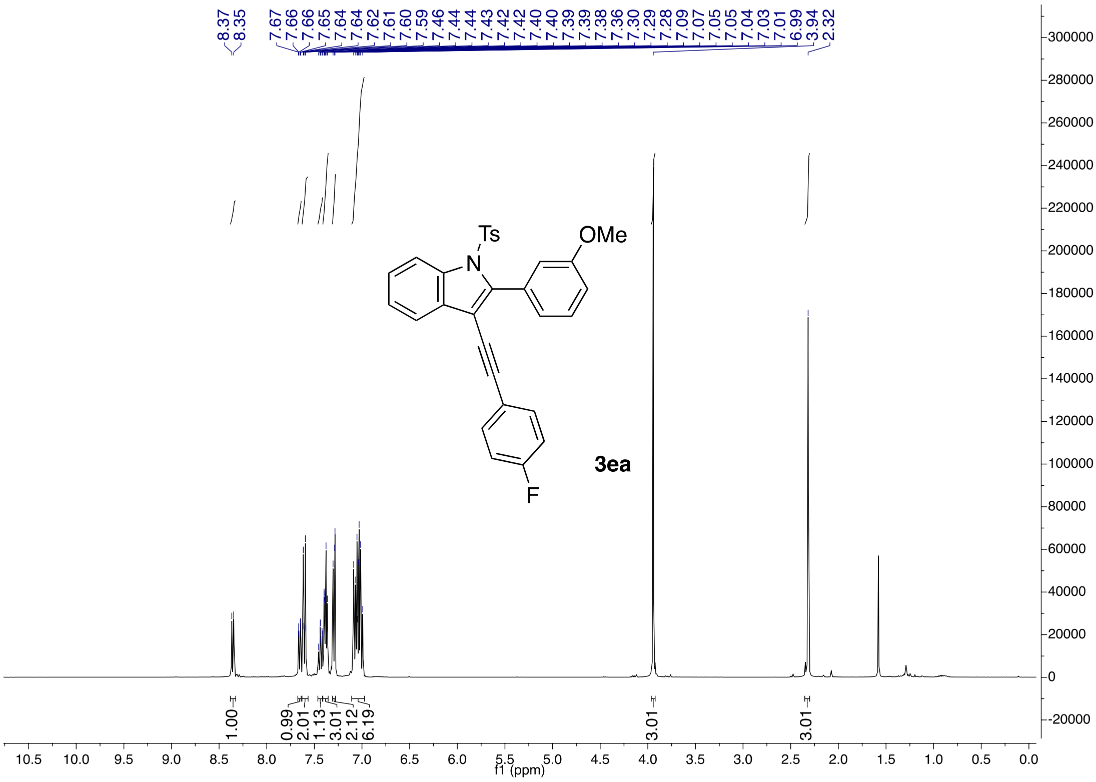

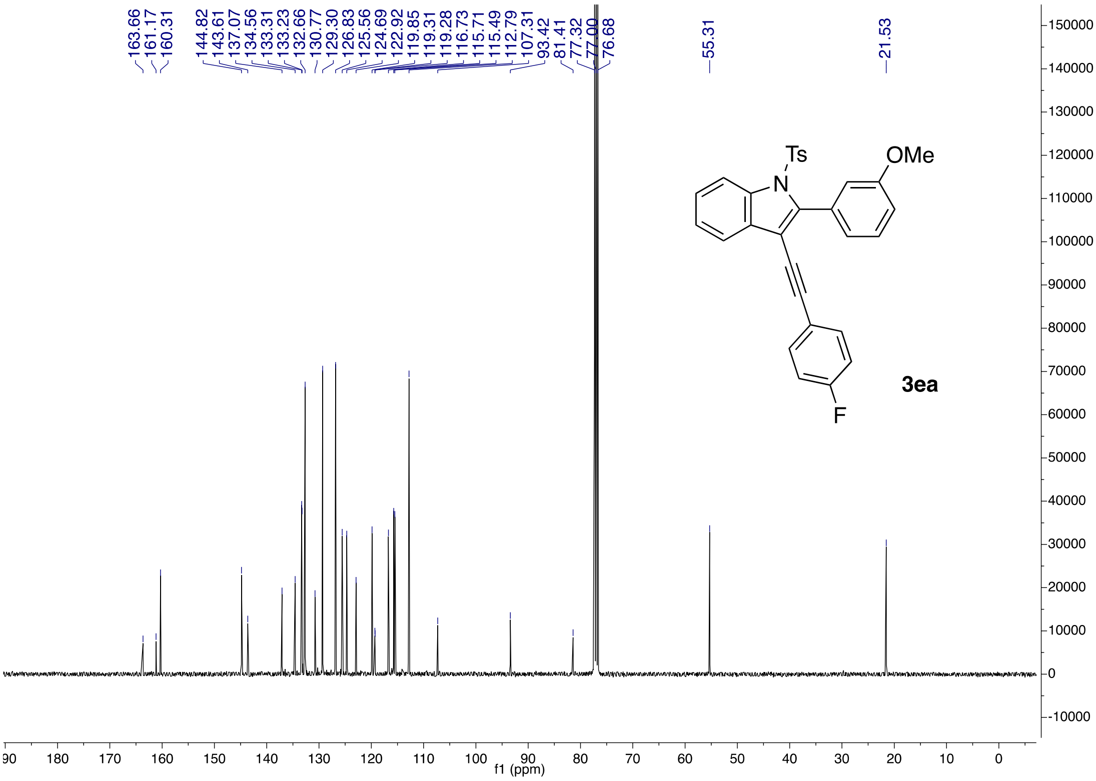

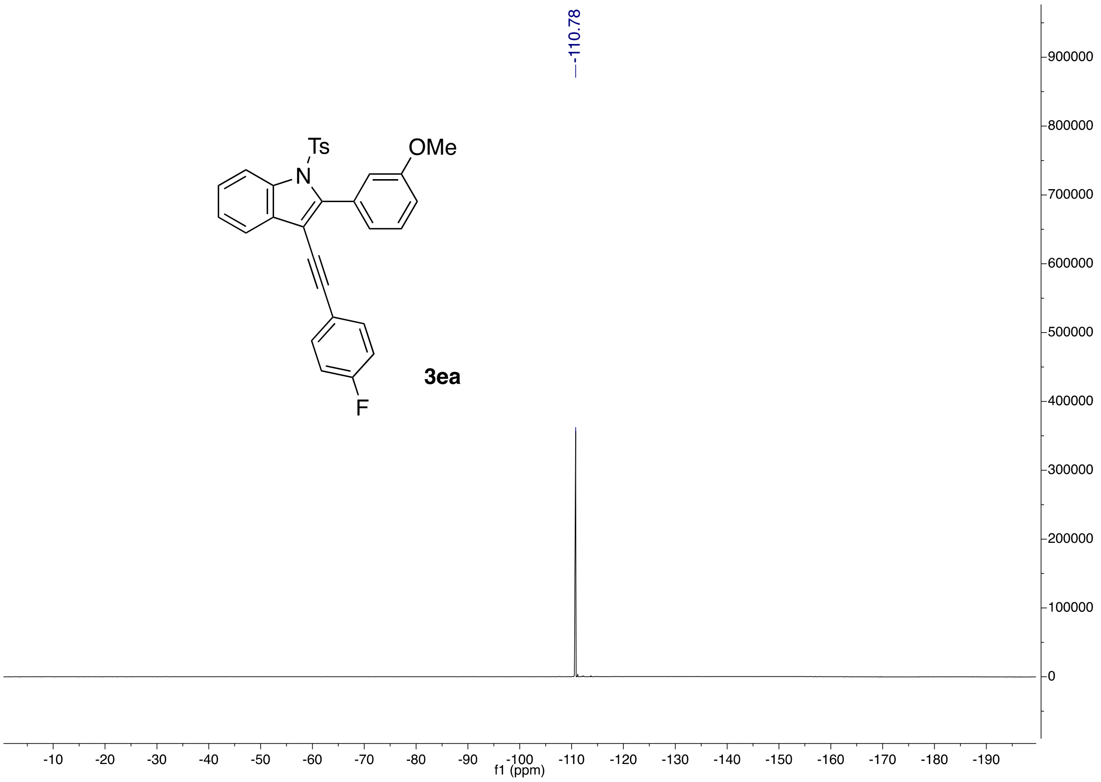

Supplementary Fig. 94 ^{1}H NMR, ^{13}C NMR and ^{19}F NMR spectra of compound **3ea**

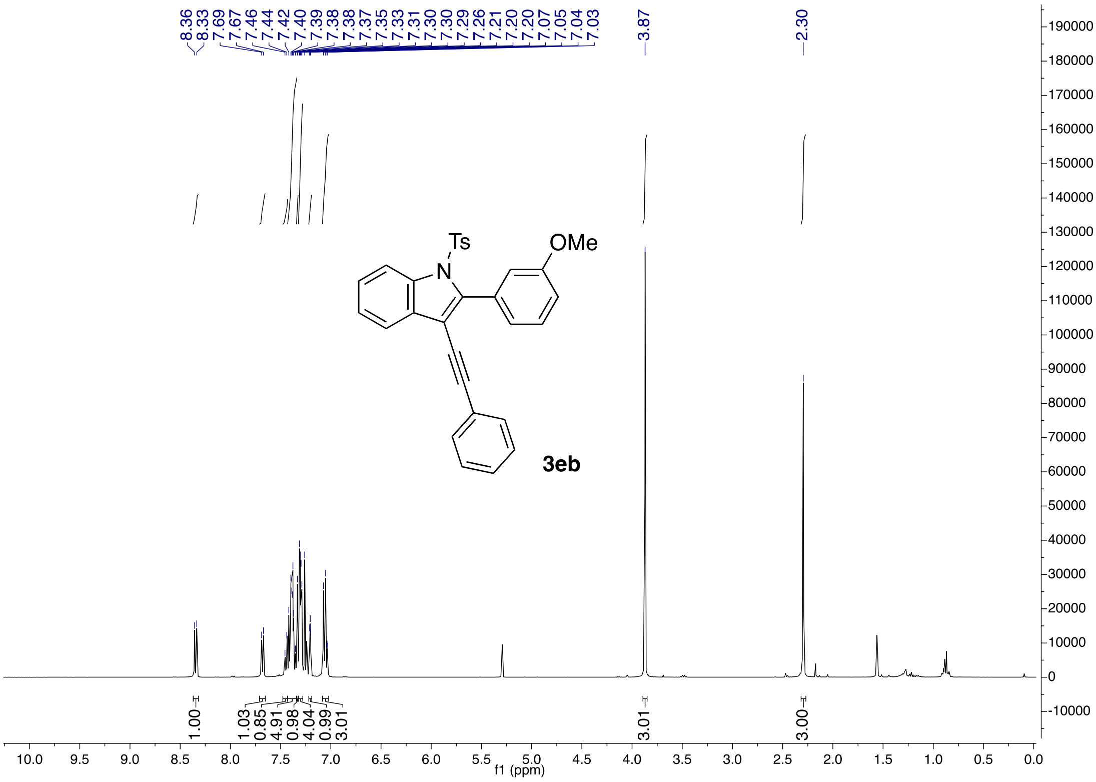

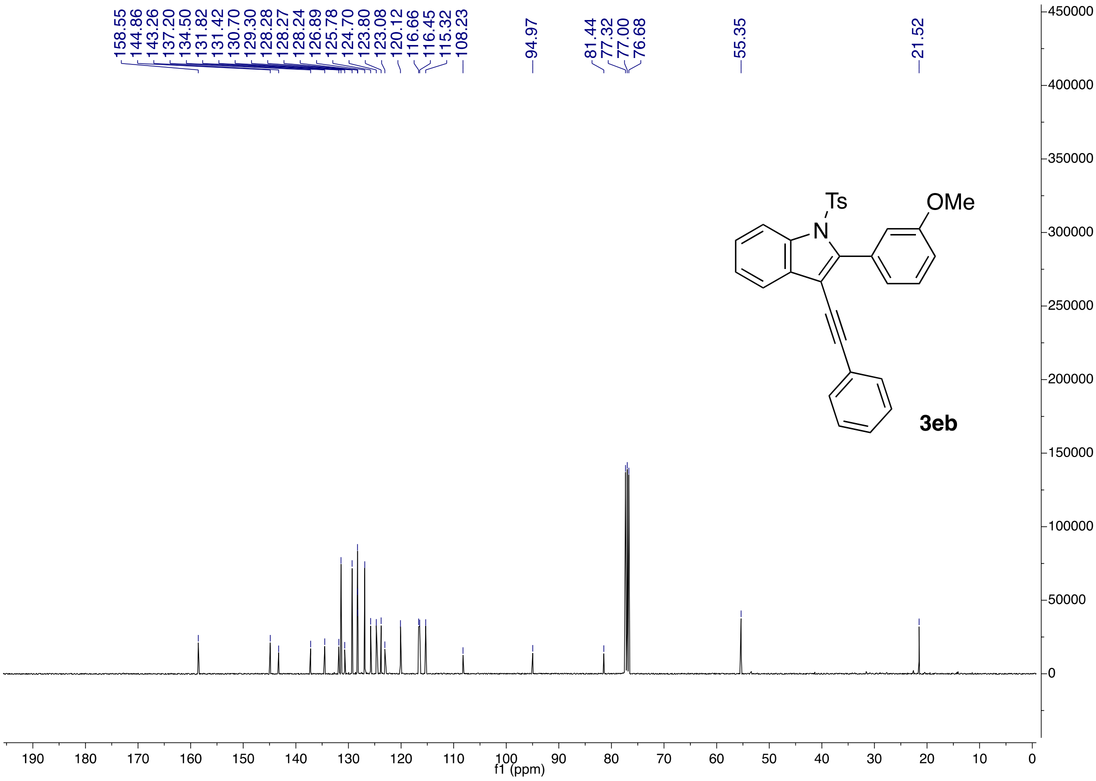

Supplementary Fig. 95 ^{1}H NMR and ^{13}C NMR spectra of compound **3eb**

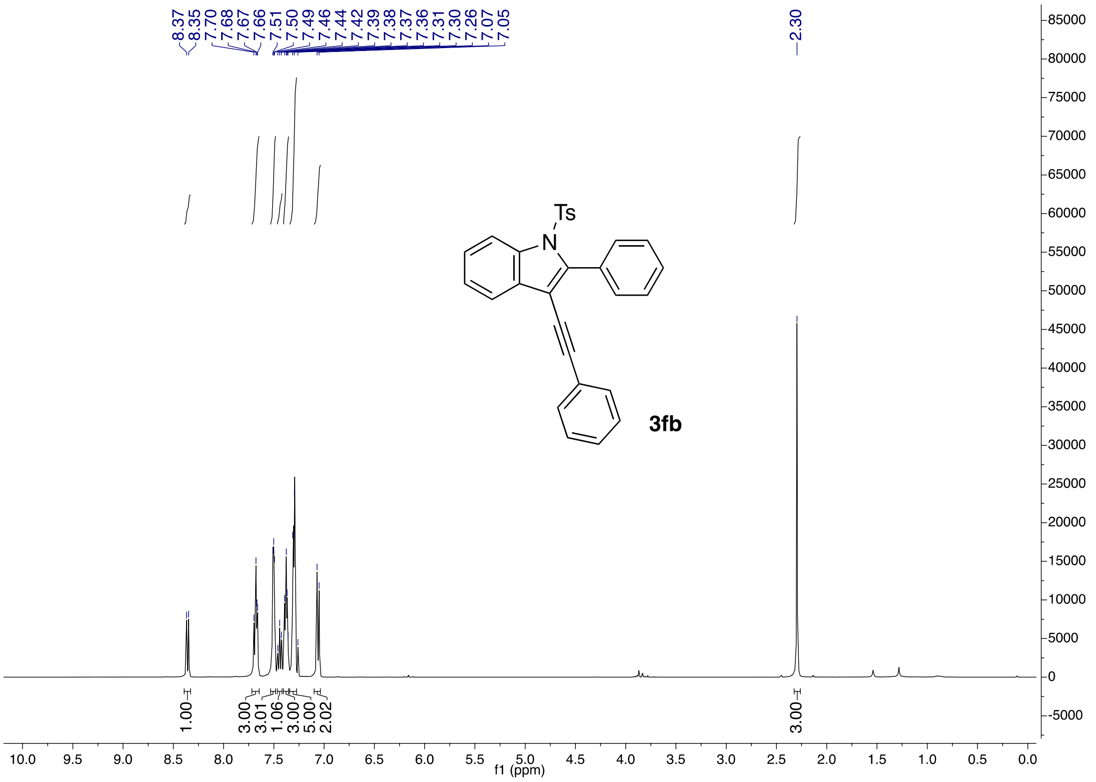

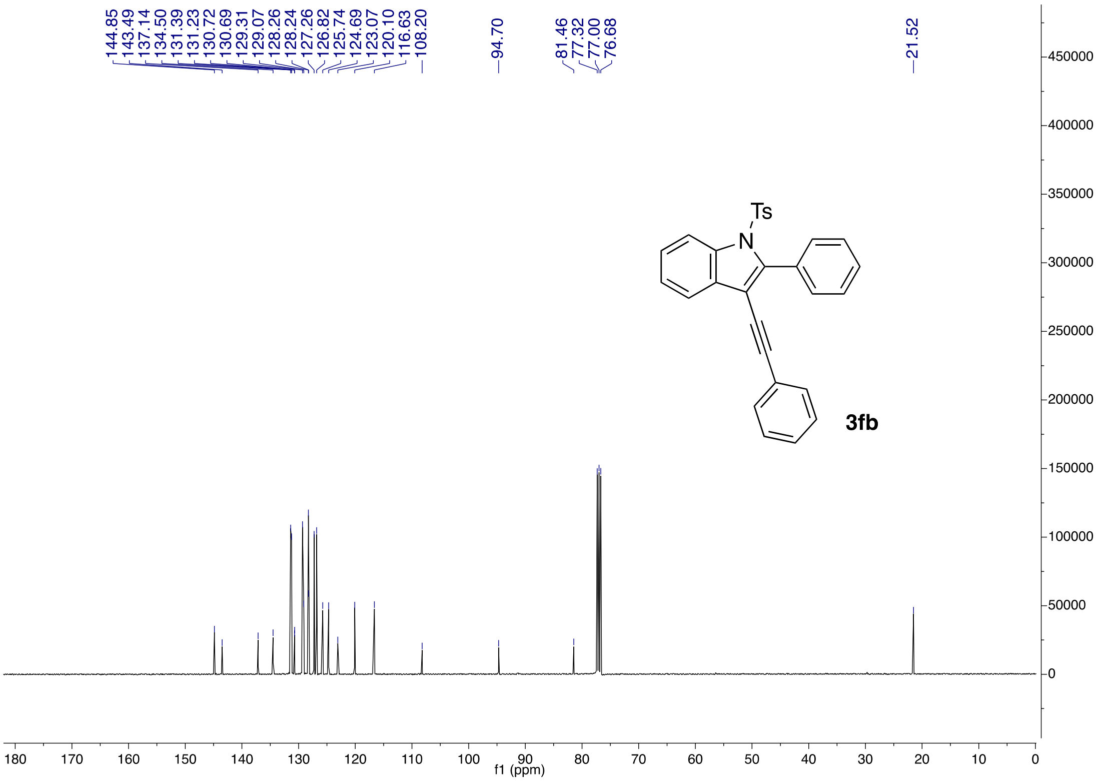

Supplementary Fig. 96 ^{1}H NMR and ^{13}C NMR spectra of compound **3fb**

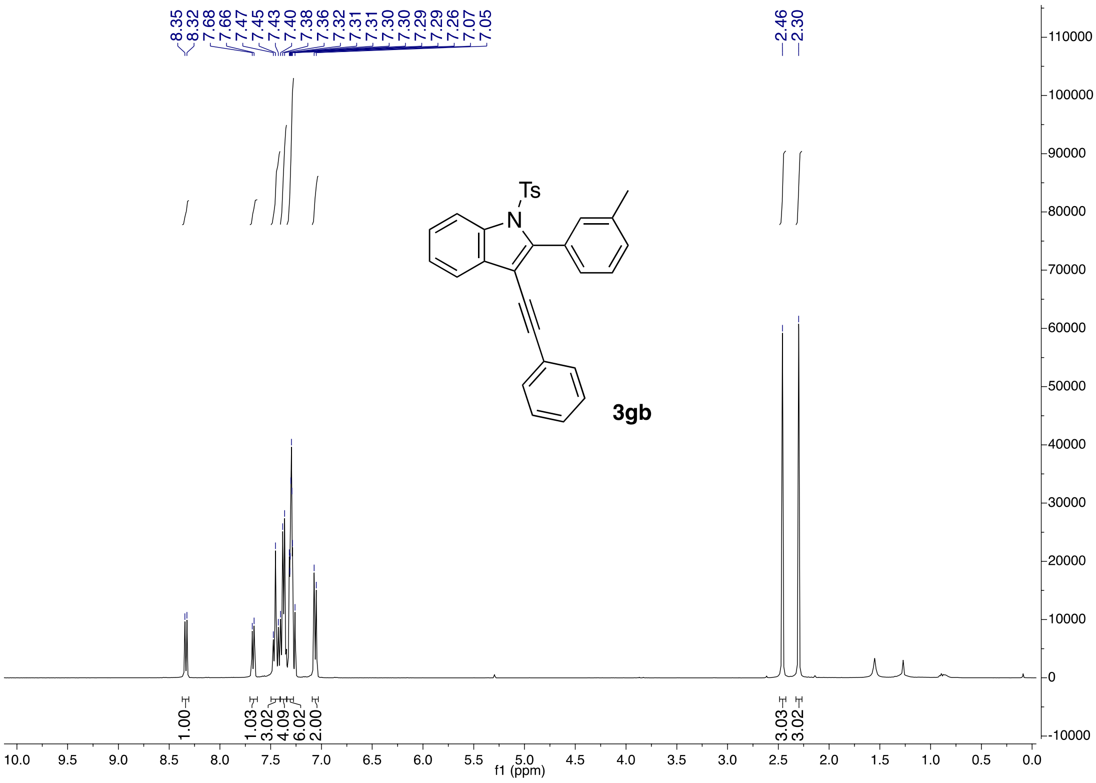

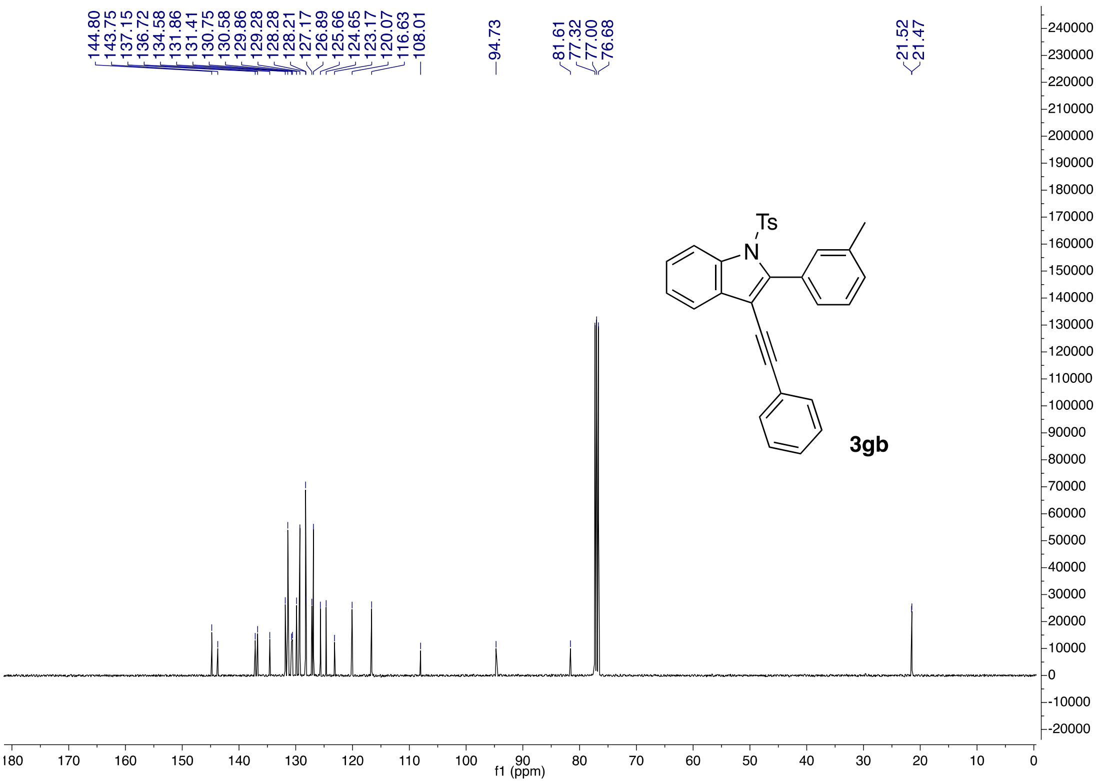

Supplementary Fig. 97 ^{1}H NMR and ^{13}C NMR spectra of compound **3gb**

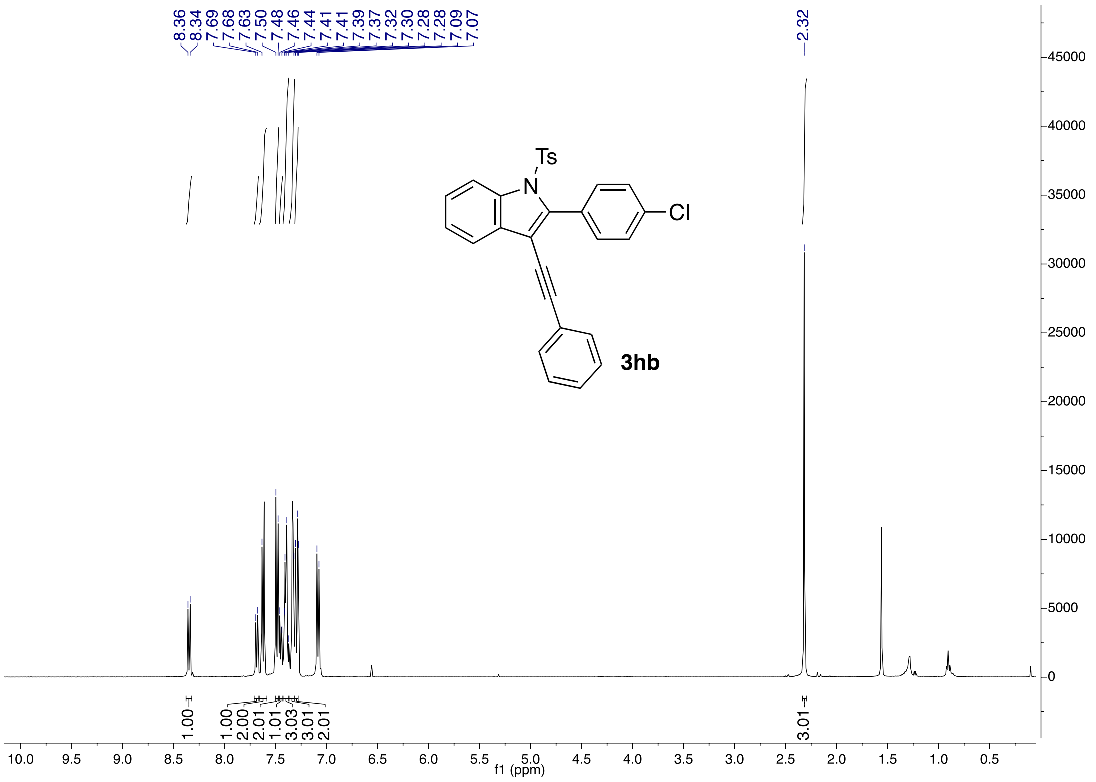

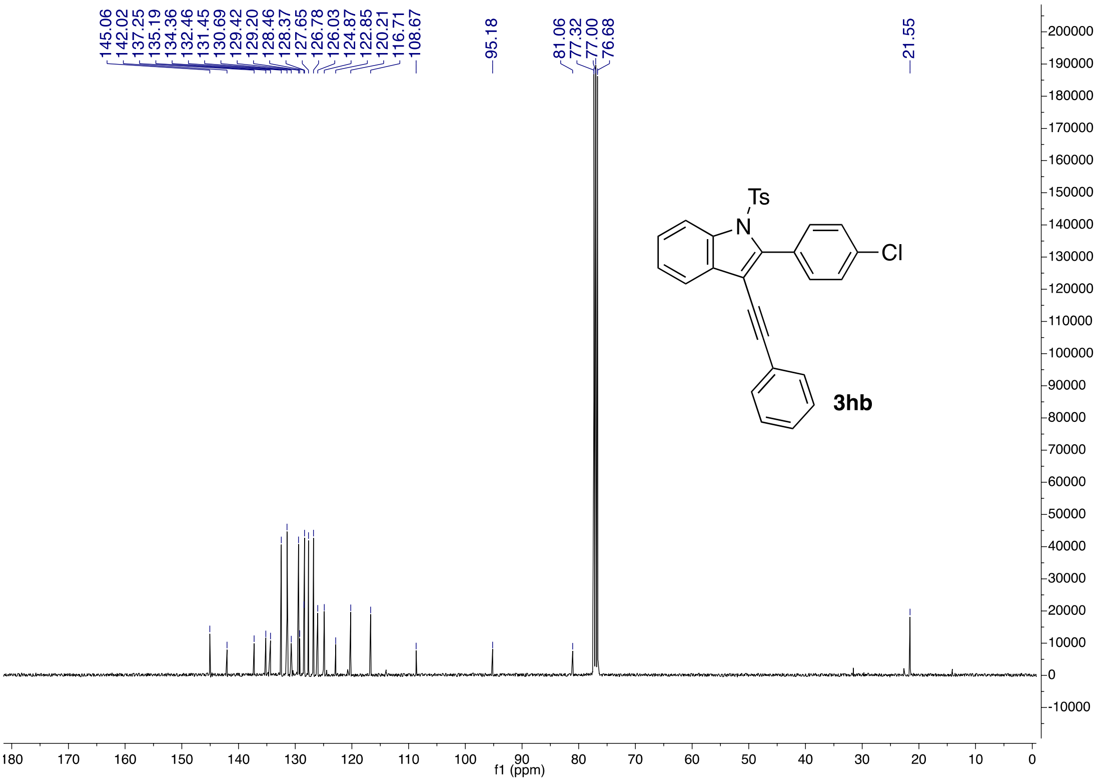

Supplementary Fig. 98 ^{1}H NMR and ^{13}C NMR spectra of compound **3hb**

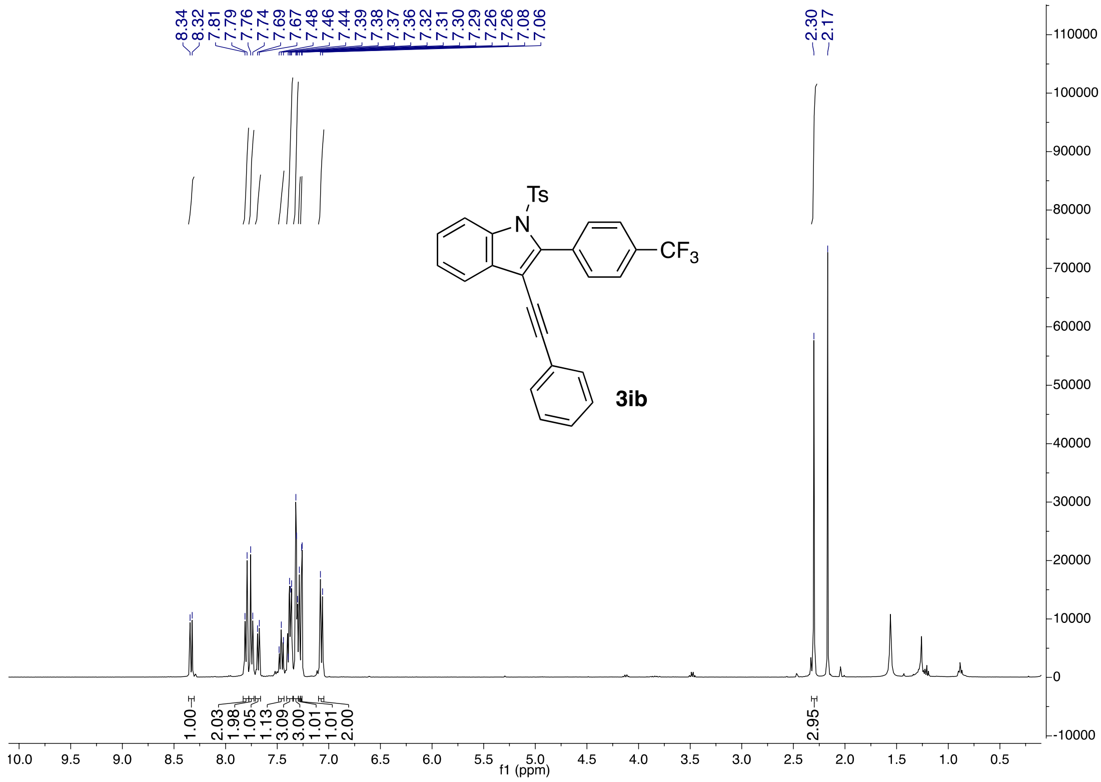
3ib
Ts
N
CF3
f1 (ppm)

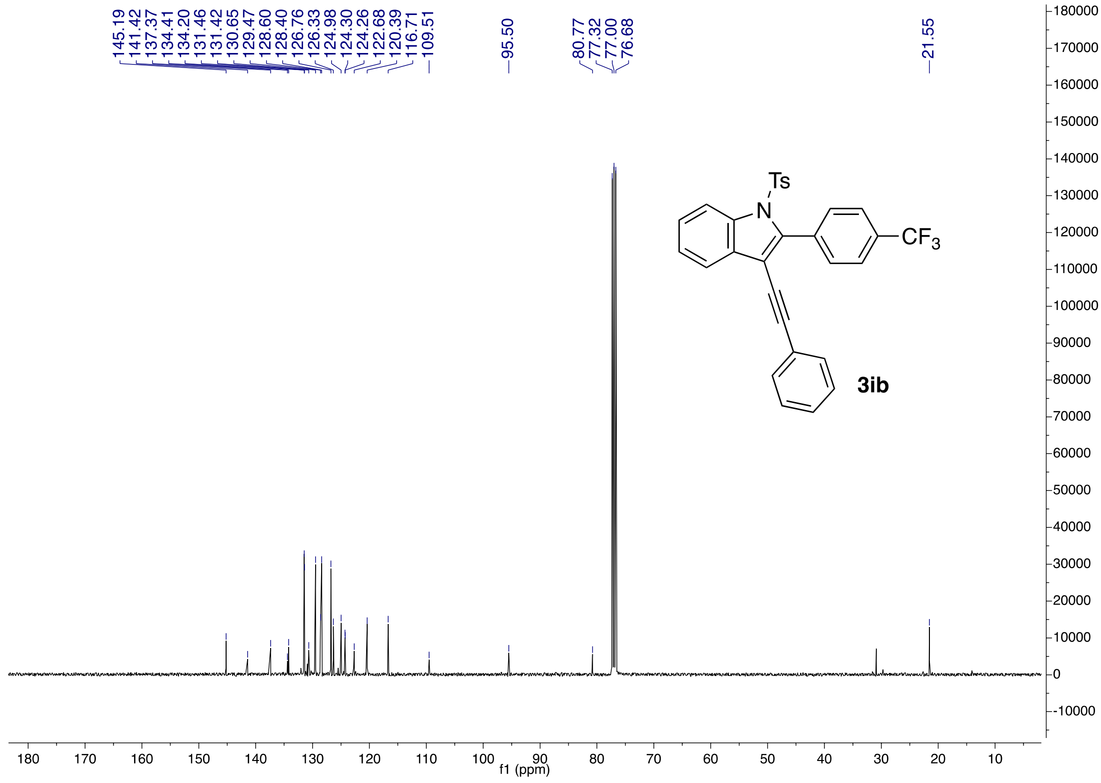
3ib
Ts
N
CF3
f1 (ppm)

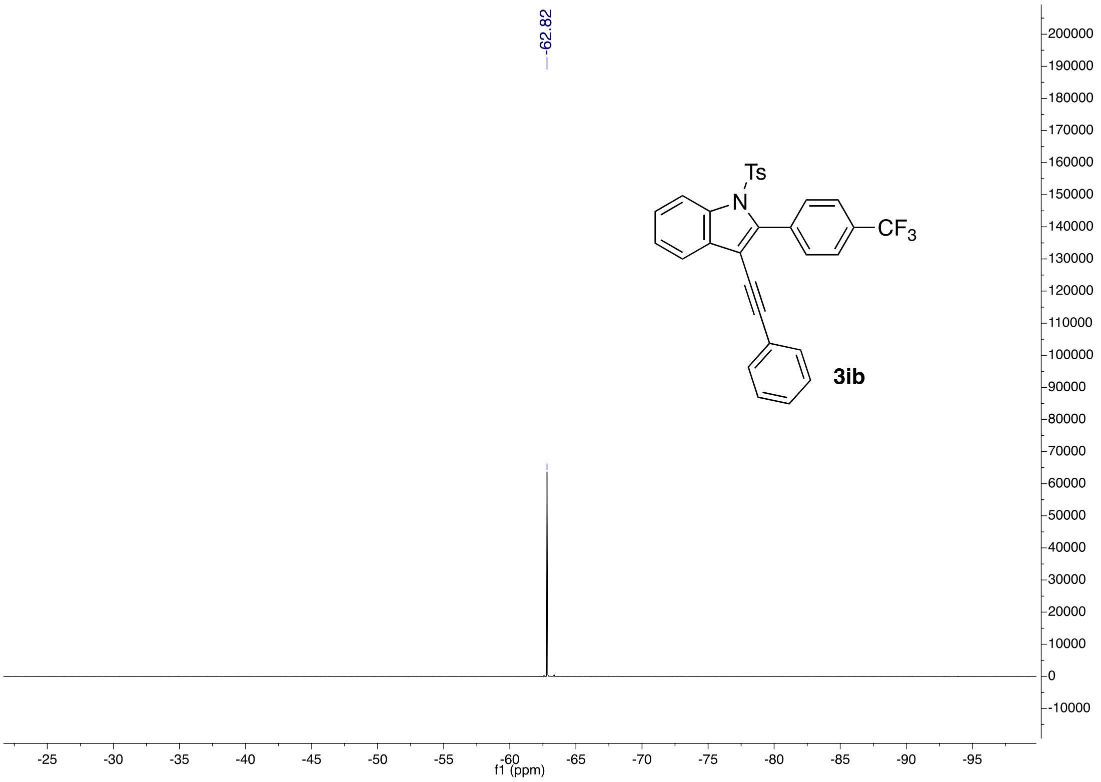

Supplementary Fig. 99 ^{1}H NMR, ^{13}C NMR and ^{19}F NMR spectra of compound **3ib**

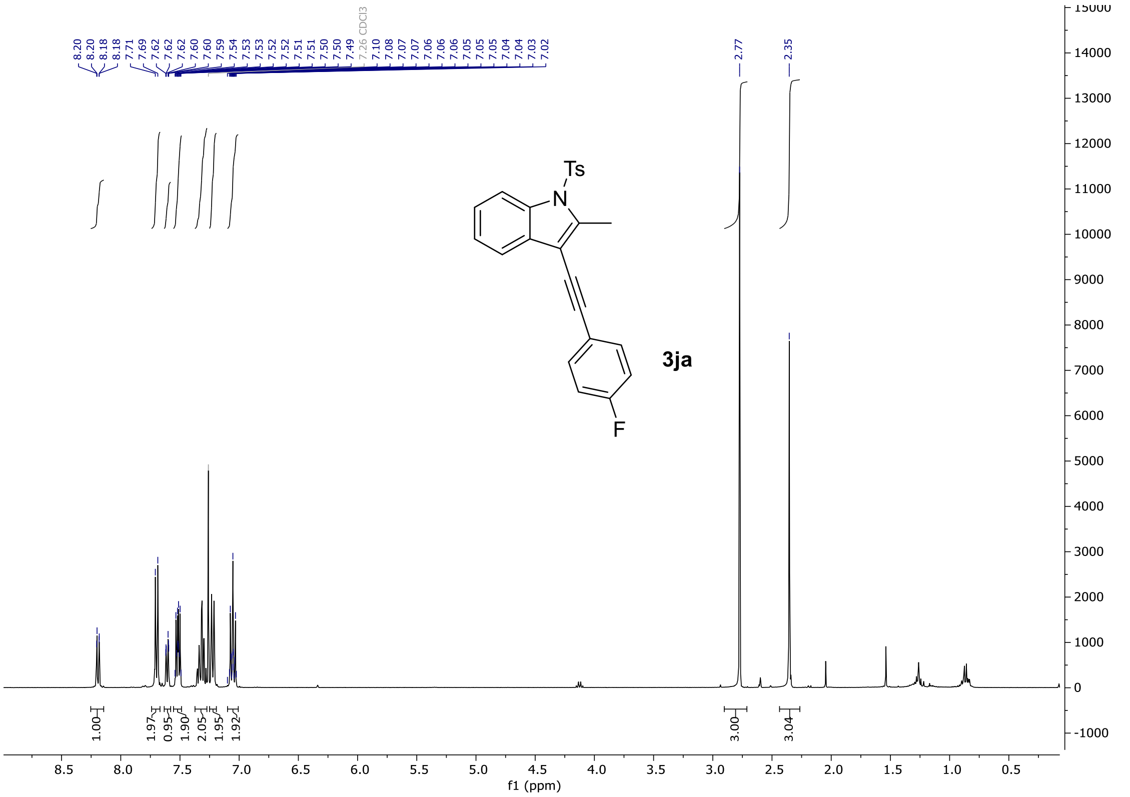

8.20
8.20
8.18
8.18
7.71
7.69
7.62
7.62
7.60
7.60
7.59
7.54
7.53
7.53
7.52
7.52
7.51
7.51
7.50
7.50
7.49
7.26 CDCl3
7.10
7.08
7.07
7.07
7.06
7.06
7.06
7.05
7.05
7.05
7.04
7.04
7.03
7.02
2.77
2.35
Ts
N
F
3ja
1.00
1.97
0.95
1.90
2.05
1.95
1.92
3.00
3.04
f1 (ppm)

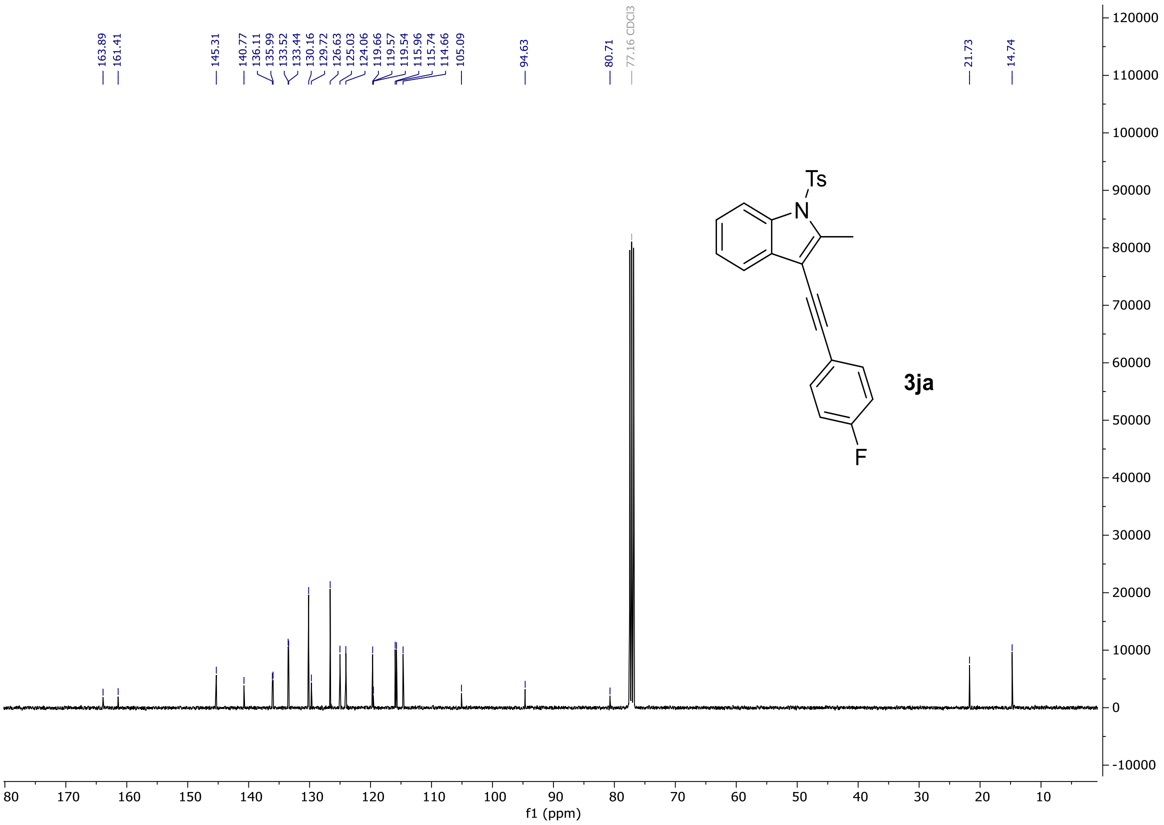

163.89
161.41
145.31
140.77
136.11
135.99
133.52
133.44
130.16
129.72
126.63
125.03
124.06
119.66
119.57
119.54
115.96
115.74
114.66
105.09
94.63
80.71
77.16 CDCl3
21.73
14.74
Ts
N
F
3ja
f1 (ppm)

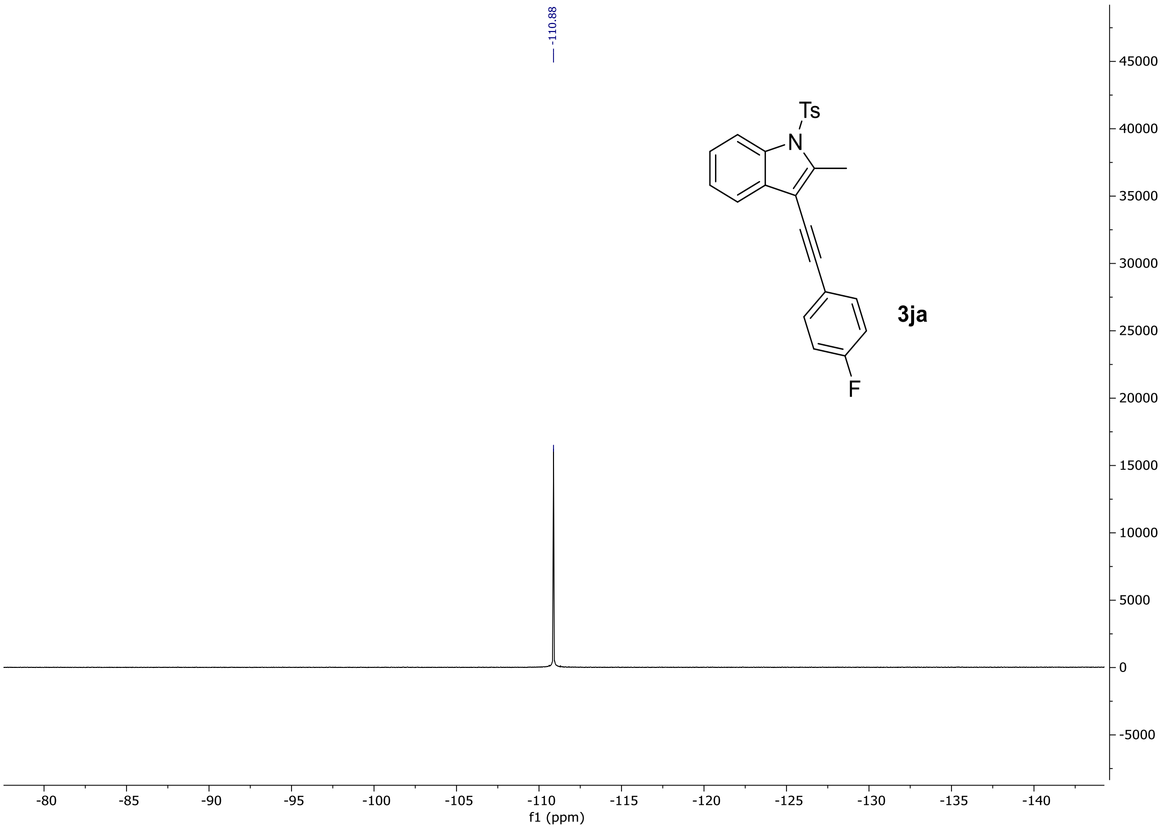

Supplementary Fig. 100 ^{1}H NMR, ^{13}C NMR and ^{19}F NMR spectra of compound **3ja**

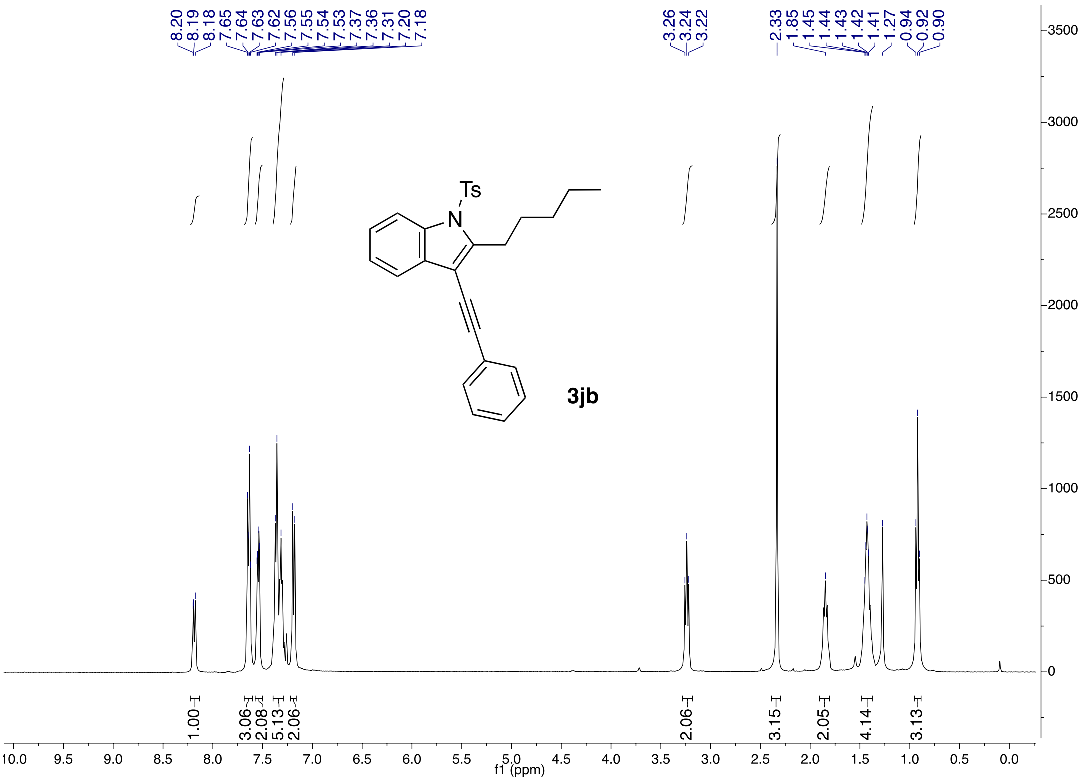

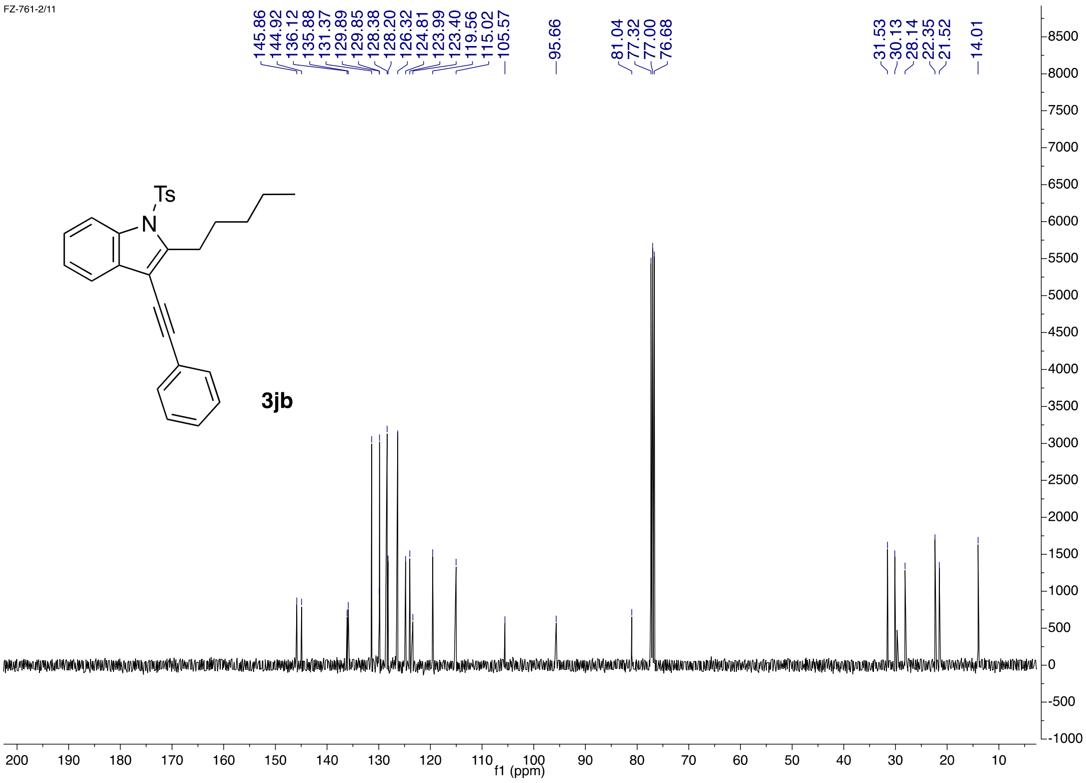

Supplementary Fig. 101 ^{1}H NMR and ^{13}C NMR spectra of compound **3jb**

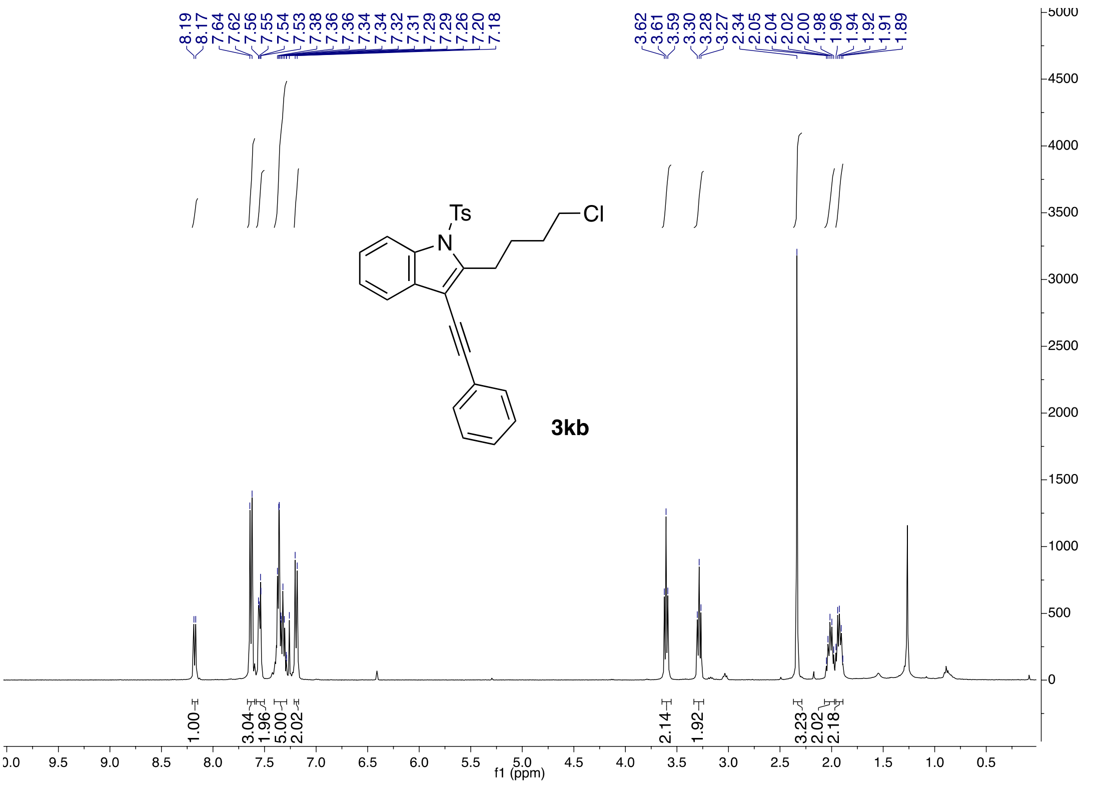

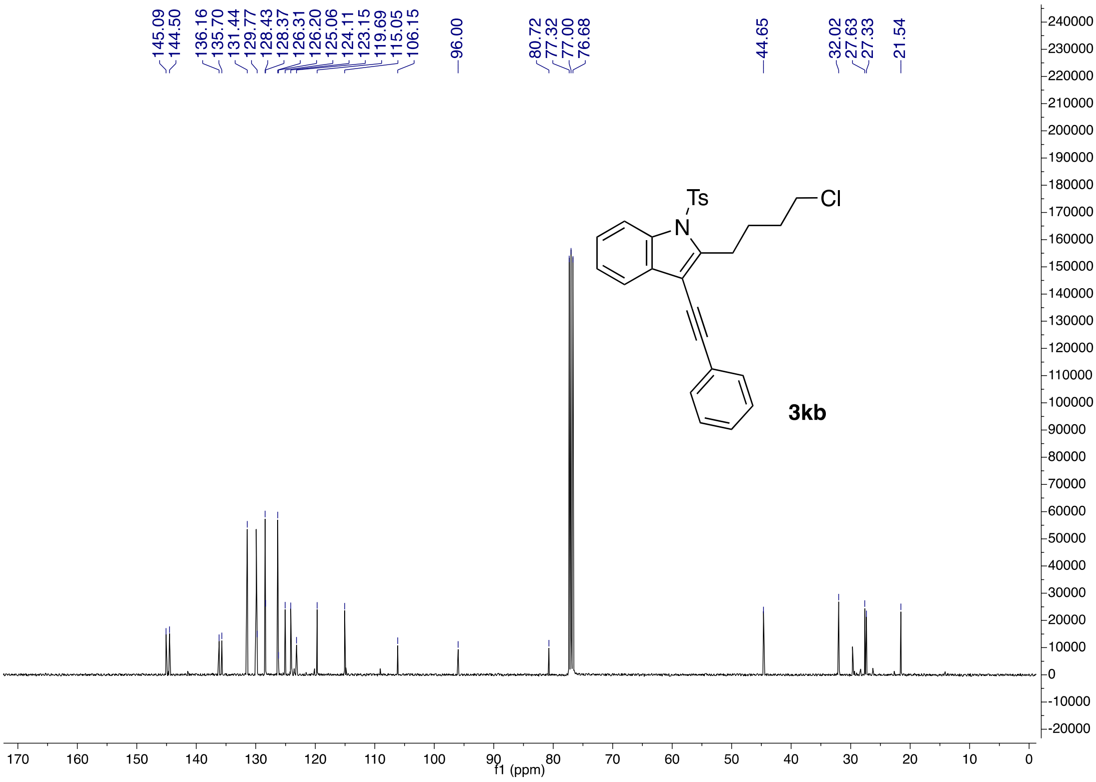

Supplementary Fig. 102 ^{1}H NMR and ^{13}C NMR spectra of compound **3kb**

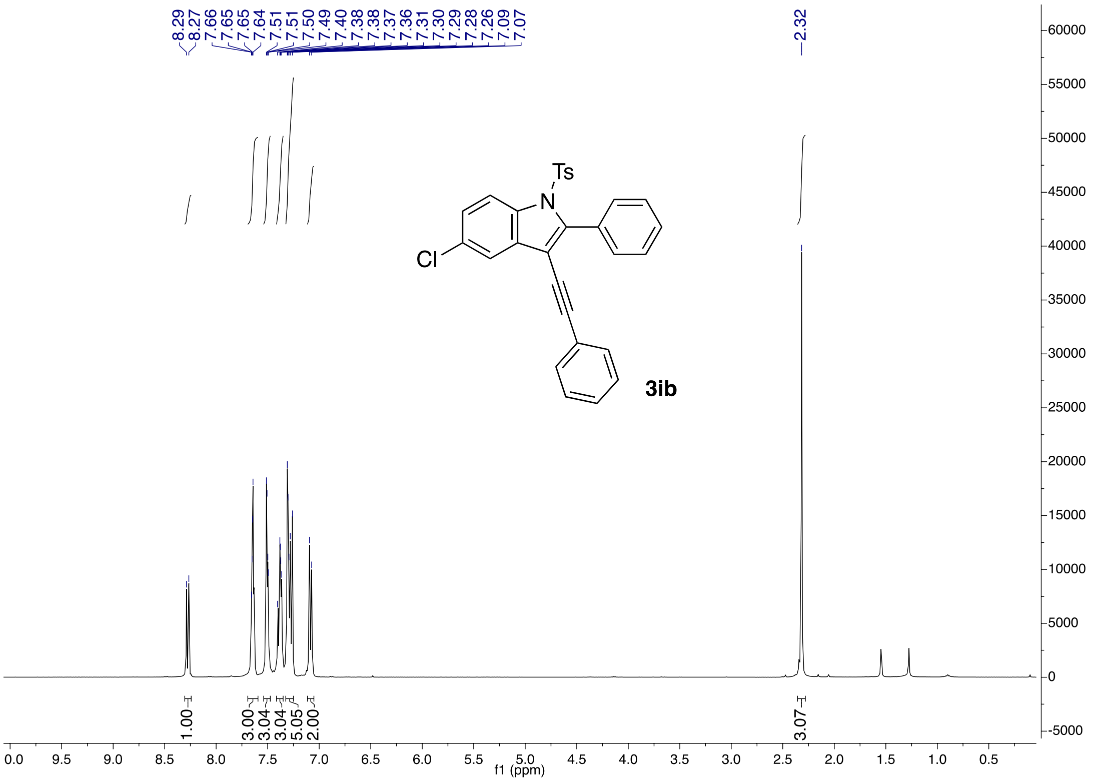

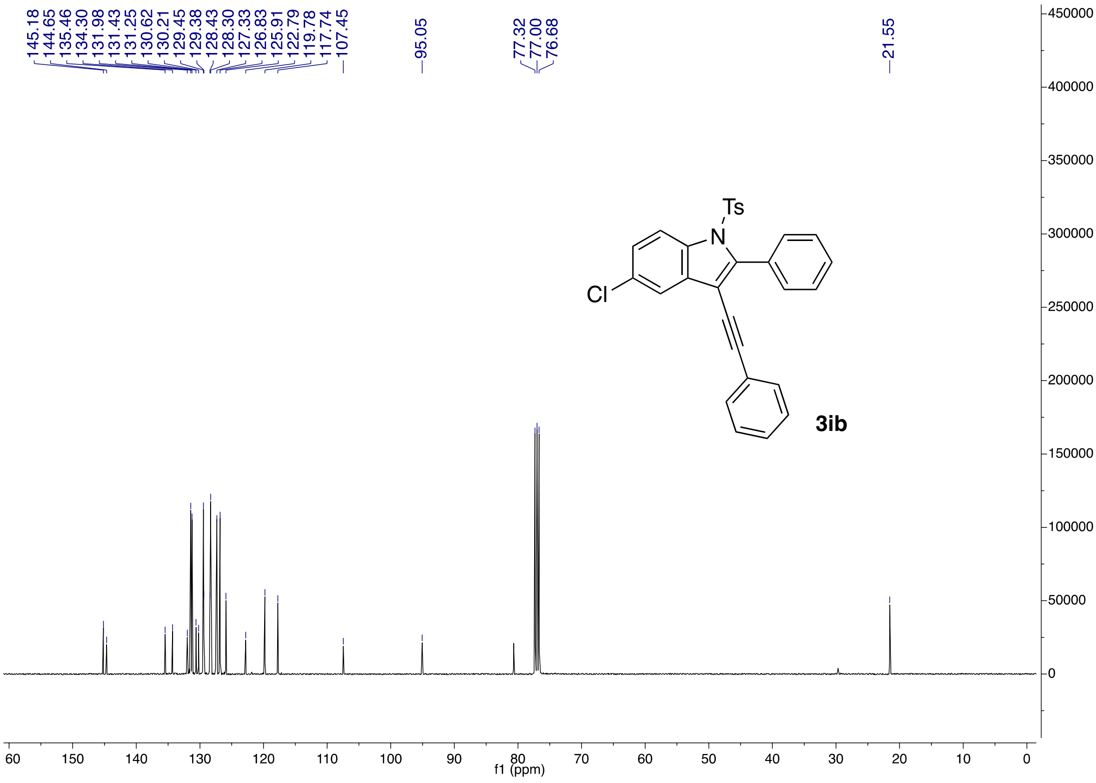

Supplementary Fig. 103 ^{1}H NMR and ^{13}C NMR spectra of compound **3ib**

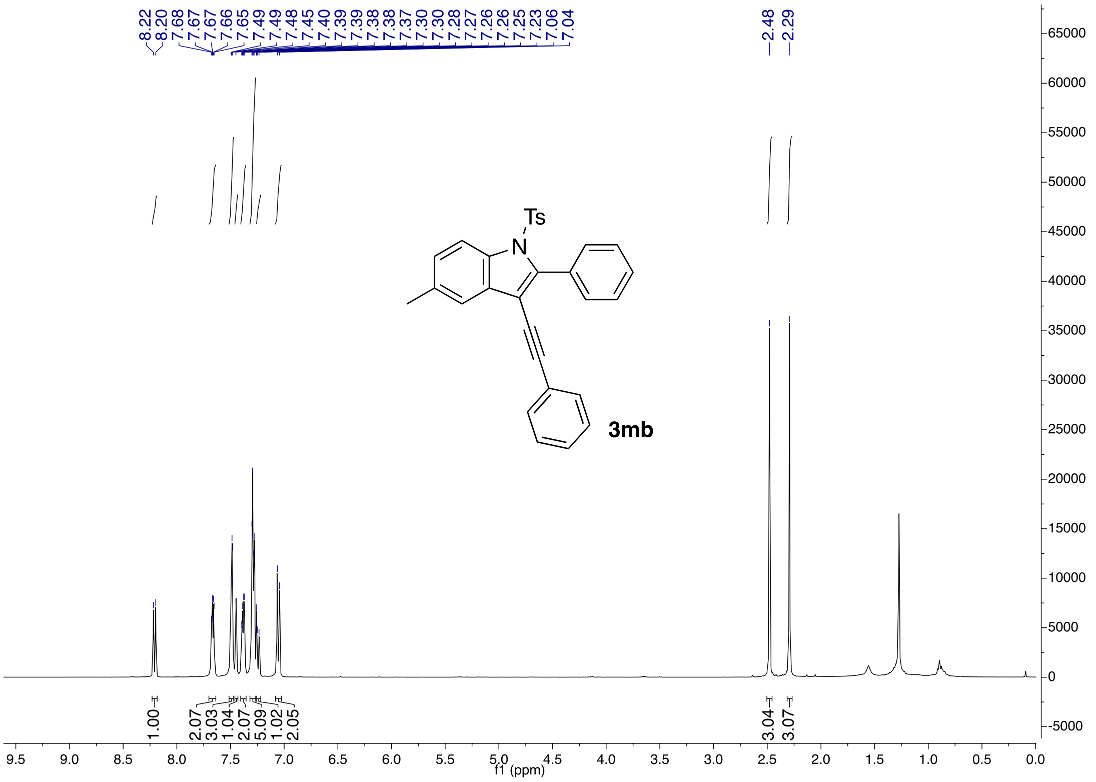

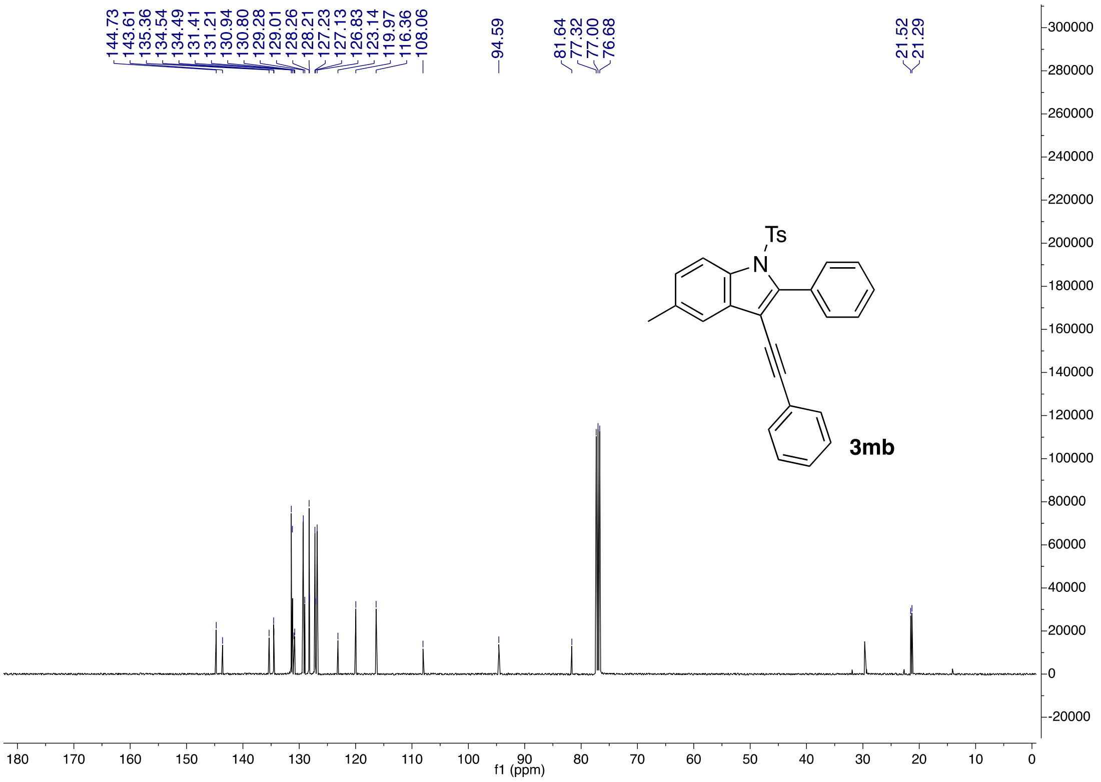

Supplementary Fig. 104 ^{1}H NMR and ^{13}C NMR spectra of compound **3mb**

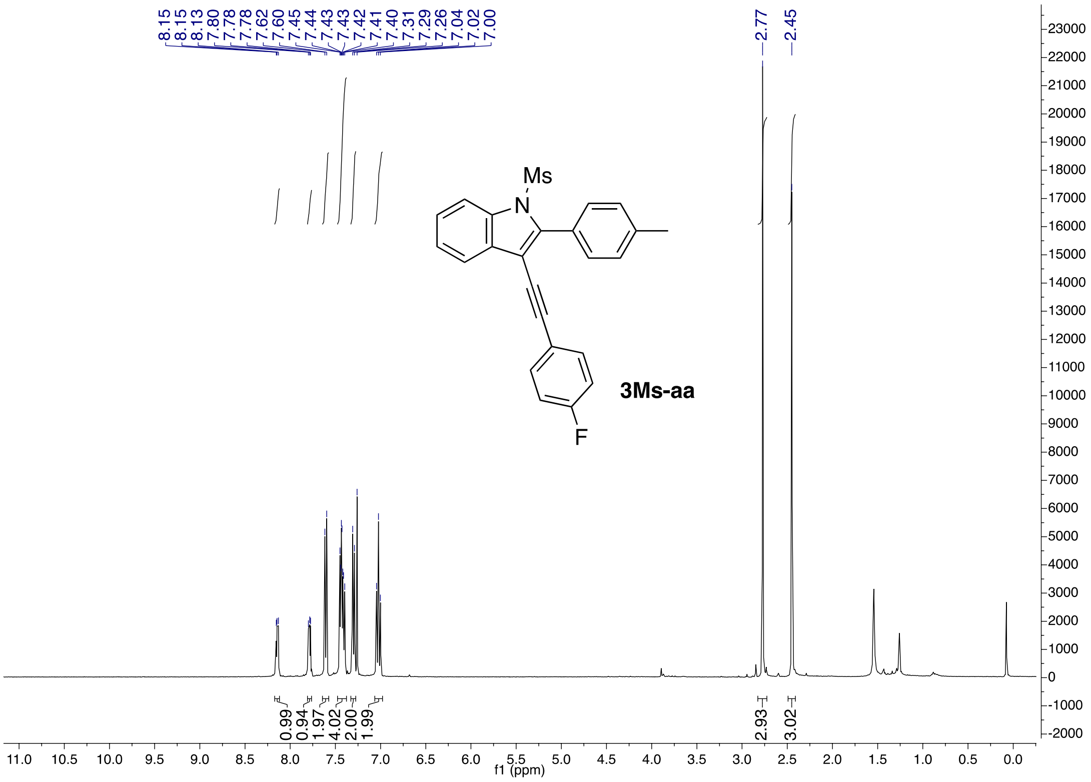
8.15
8.15
8.13
7.80
7.78
7.62
7.60
7.45
7.44
7.43
7.43
7.42
7.41
7.40
7.31
7.29
7.26
7.04
7.02
7.00
2.77
2.45
Ms
N
F
3Ms-aa
0.99
0.94
1.97
4.02
2.00
1.99
2.93
3.02
f1 (ppm)

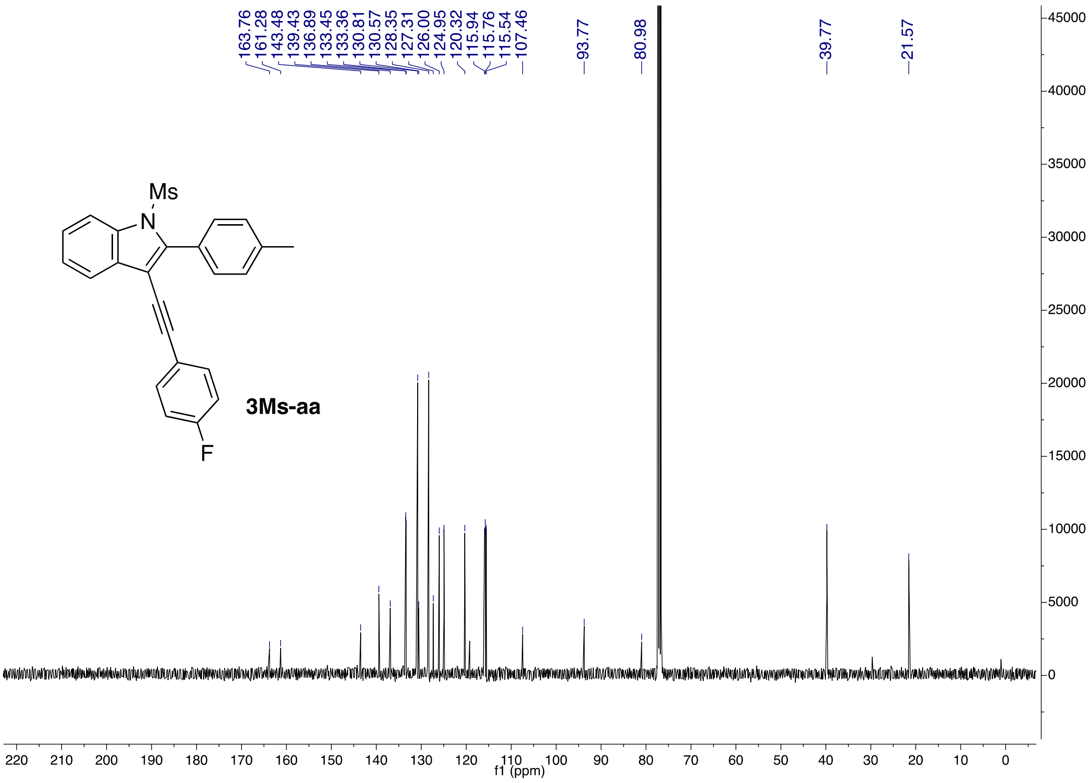
163.76
161.28
143.48
139.43
136.89
133.45
133.36
130.81
130.57
128.35
127.31
126.00
124.95
120.32
115.94
115.76
115.54
107.46
93.77
80.98
39.77
21.57
Ms
N
F
3Ms-aa
f1 (ppm)

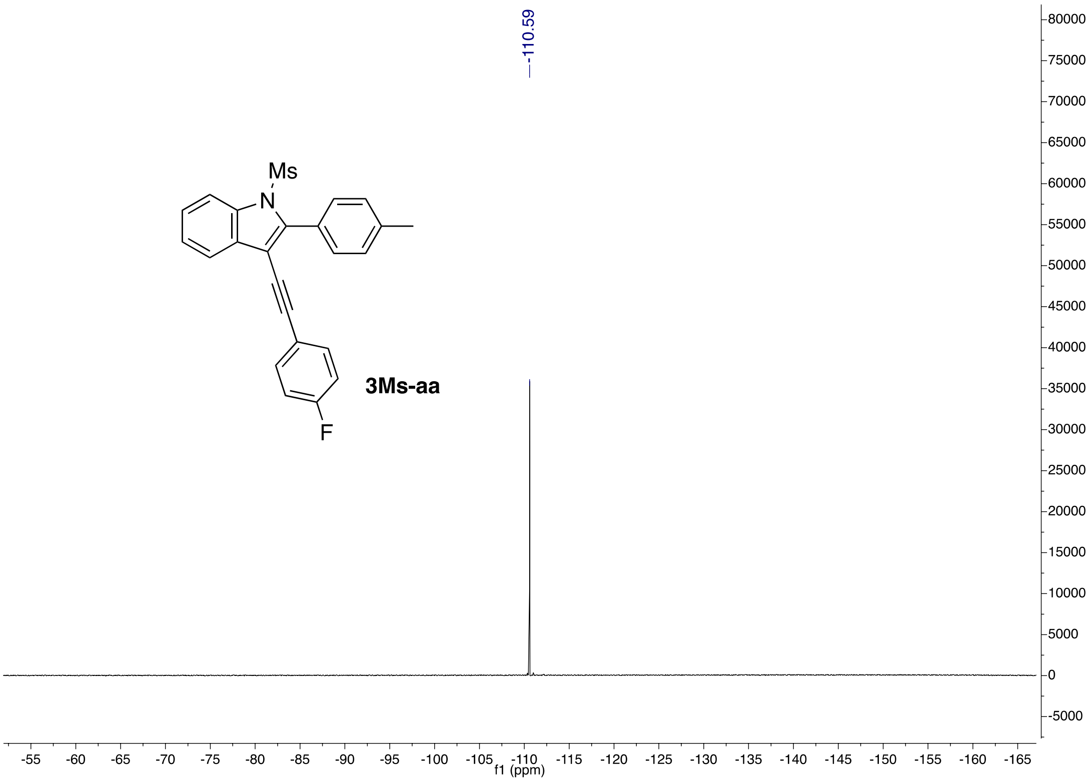

Supplementary Fig. 105 ^{1}H NMR, ^{13}C NMR and ^{19}F NMR spectra of compound **3Ms-aa**

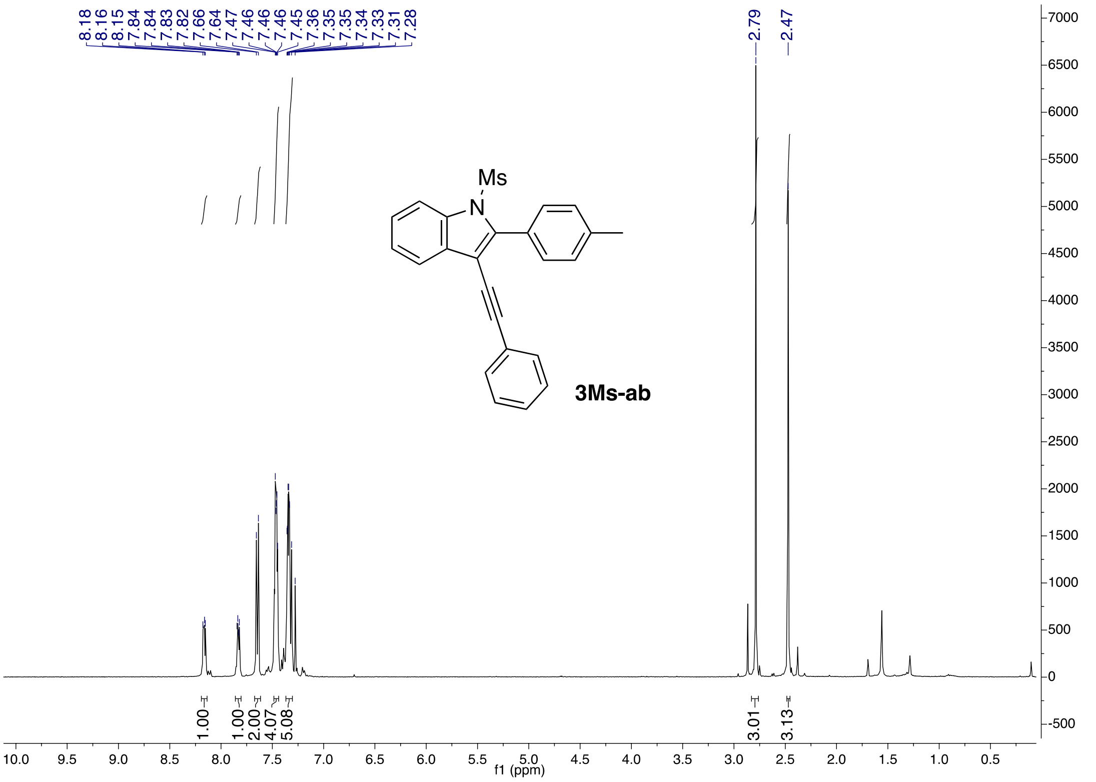

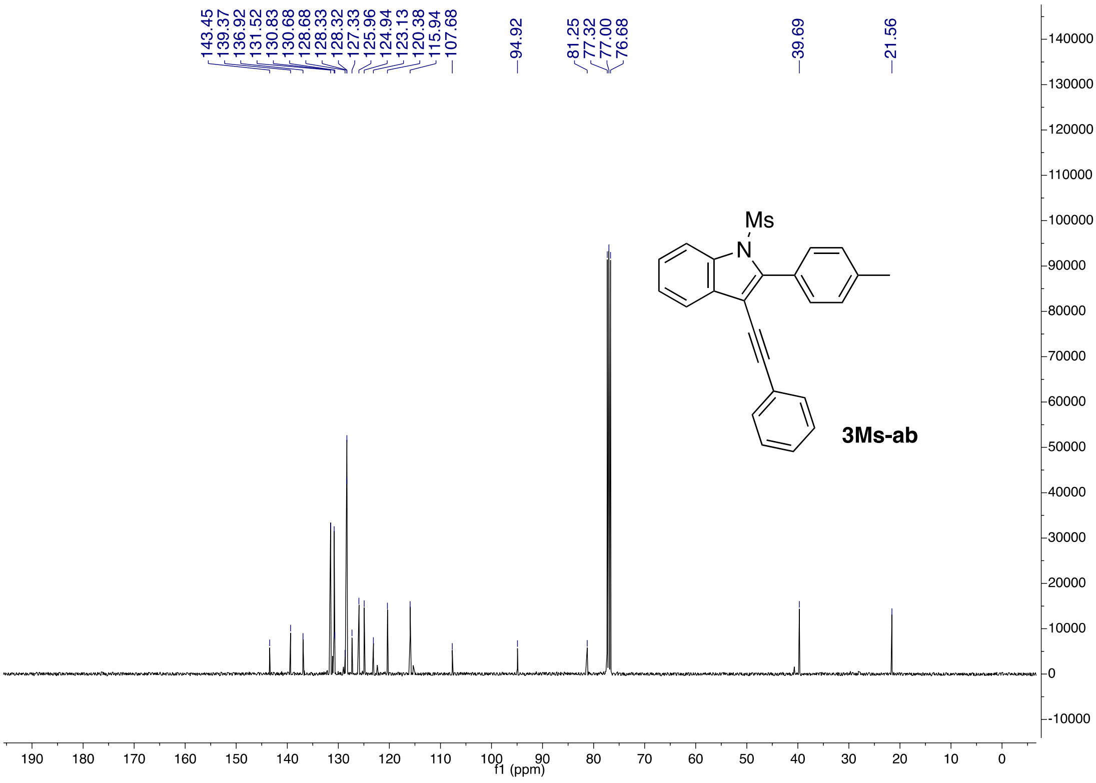

Supplementary Fig. 106 ^{1}H NMR and ^{13}C NMR spectra of compound **3Ms-ab**

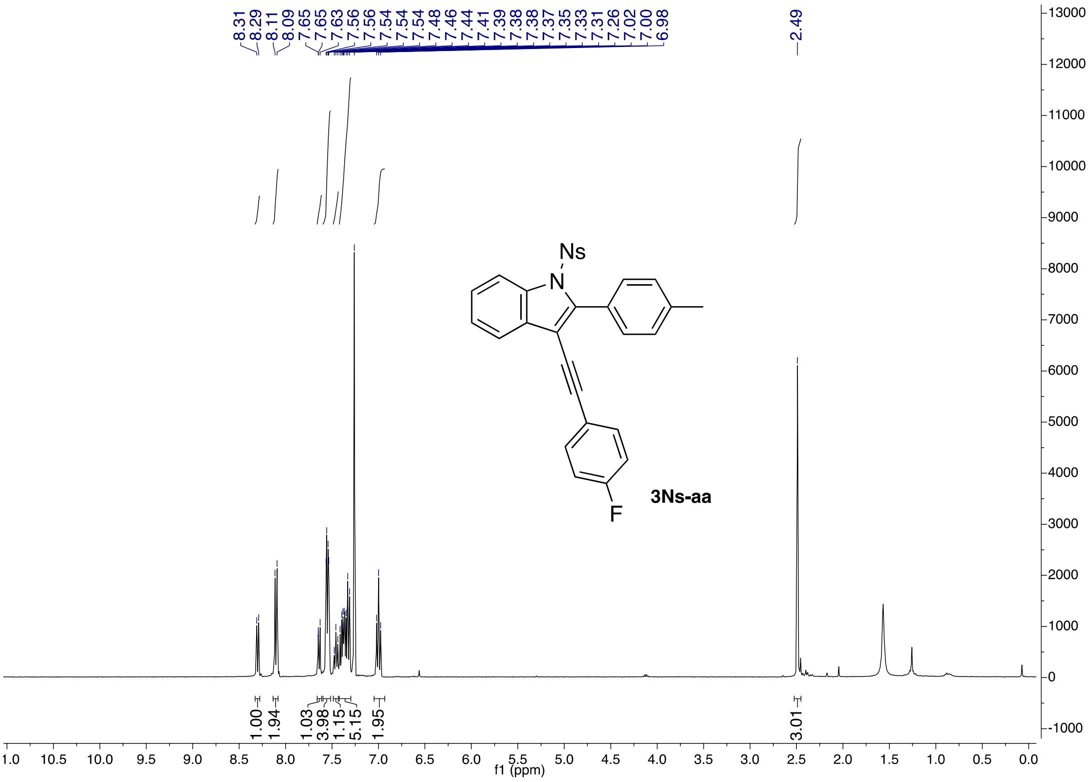
8.31
8.29
8.11
8.09
7.65
7.65
7.63
7.56
7.56
7.54
7.54
7.54
7.48
7.46
7.44
7.41
7.39
7.38
7.38
7.37
7.35
7.33
7.31
7.26
7.02
7.00
6.98
2.49
1.00
1.94
1.03
3.98
1.15
5.15
1.95
3.01
Ns
N
F
f1 (ppm)

3Ns-aa

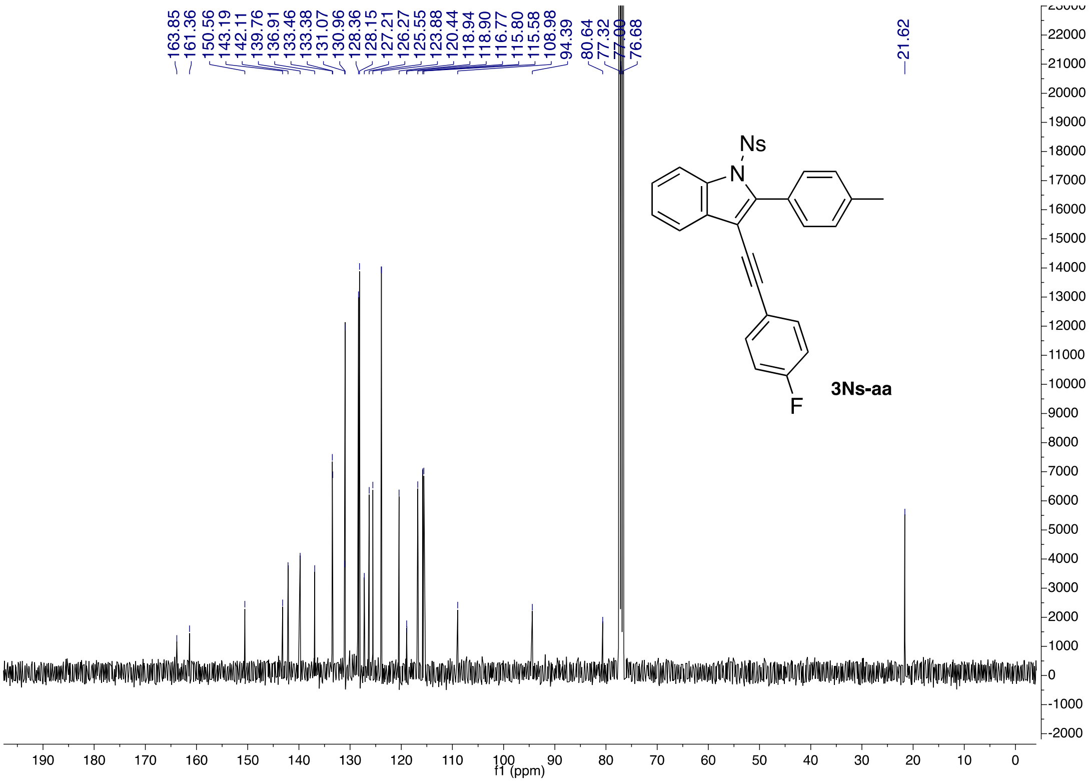
163.85
161.36
150.56
143.19
142.11
139.76
136.91
133.46
133.38
131.07
130.96
128.36
128.15
127.21
126.27
125.55
123.88
120.44
118.94
118.90
116.77
115.80
115.58
108.98
94.39
80.64
77.32
77.00
76.68
21.62
Ns
N
F
f1 (ppm)

3Ns-aa

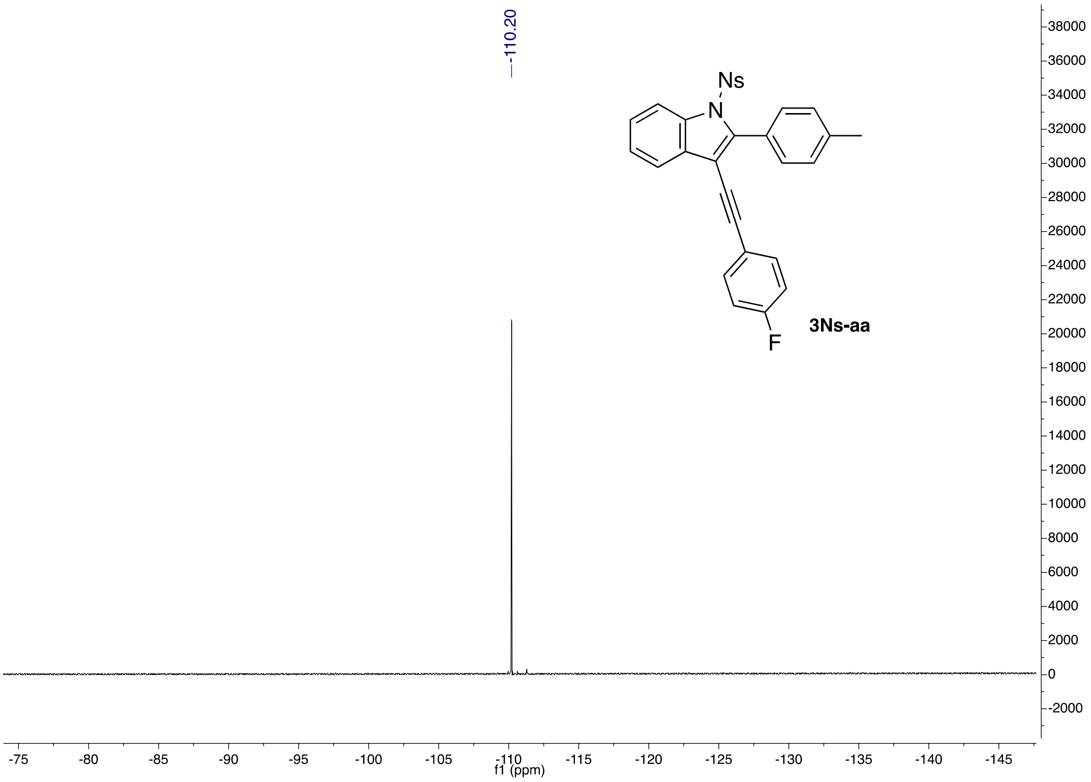

Supplementary Fig. 107 ^{1}H NMR, ^{13}C NMR and ^{19}F NMR spectra of compound **3Ns-aa**

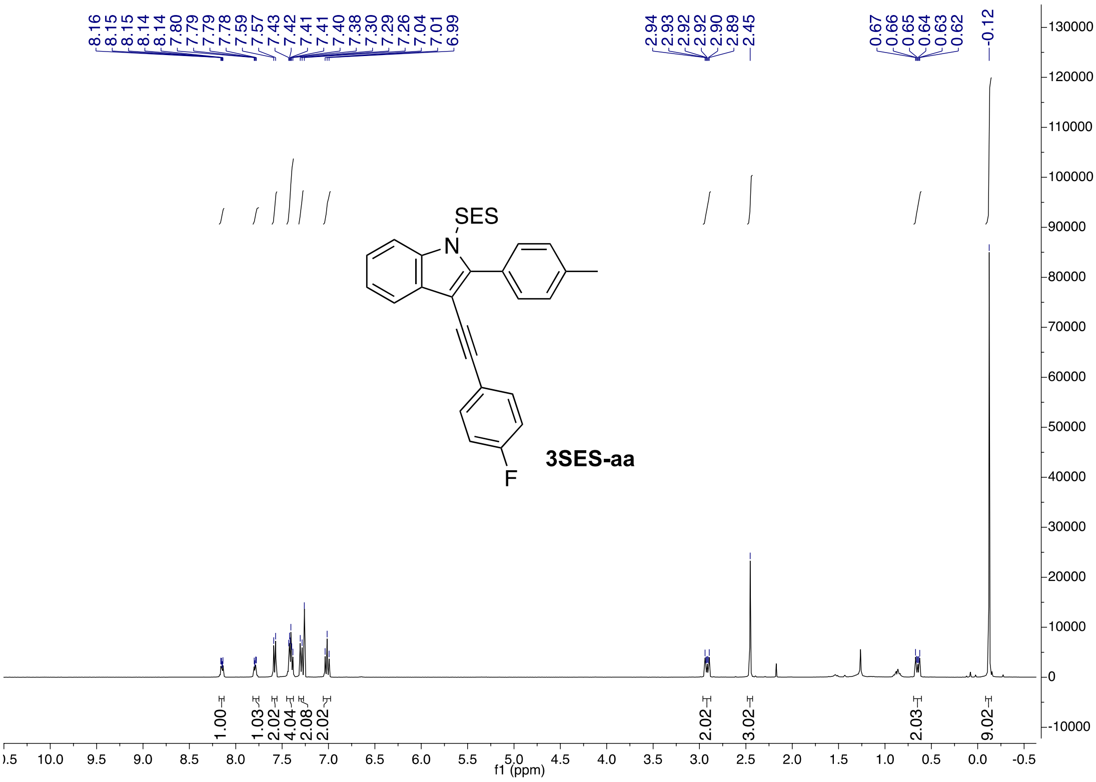
8.16
8.15
8.15
8.14
7.80
7.79
7.79
7.78
7.59
7.57
7.43
7.42
7.41
7.41
7.40
7.38
7.30
7.29
7.26
7.04
7.01
6.99
2.94
2.93
2.92
2.92
2.90
2.89
2.45
0.67
0.66
0.65
0.64
0.63
0.62
0.12
SES
N
F
3SES-aa
1.00
1.03
2.02
4.04
2.08
2.02
2.02
3.02
2.03
9.02
f1 (ppm)

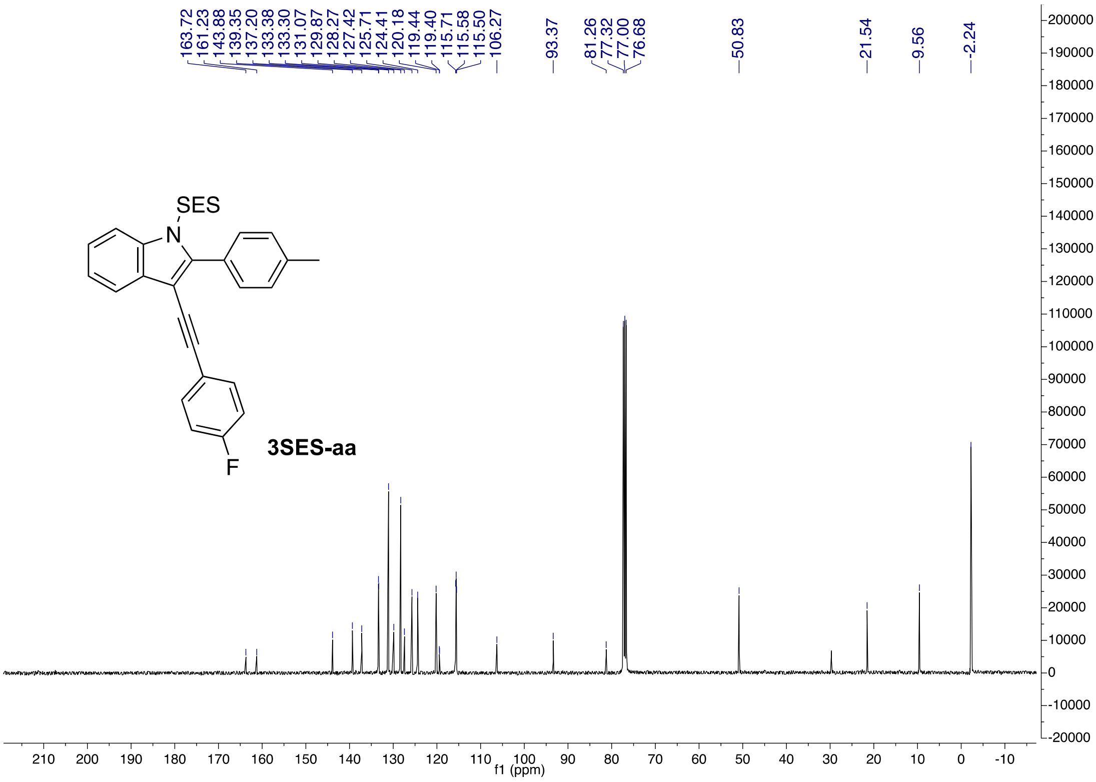
163.72
161.23
143.88
139.35
137.20
133.38
133.30
131.07
129.87
128.27
127.42
125.71
124.41
120.18
119.44
119.40
115.71
115.58
115.50
106.27
93.37
81.26
77.32
77.00
76.68
50.83
21.54
9.56
-2.24
SES
N
F
3SES-aa
f1 (ppm)

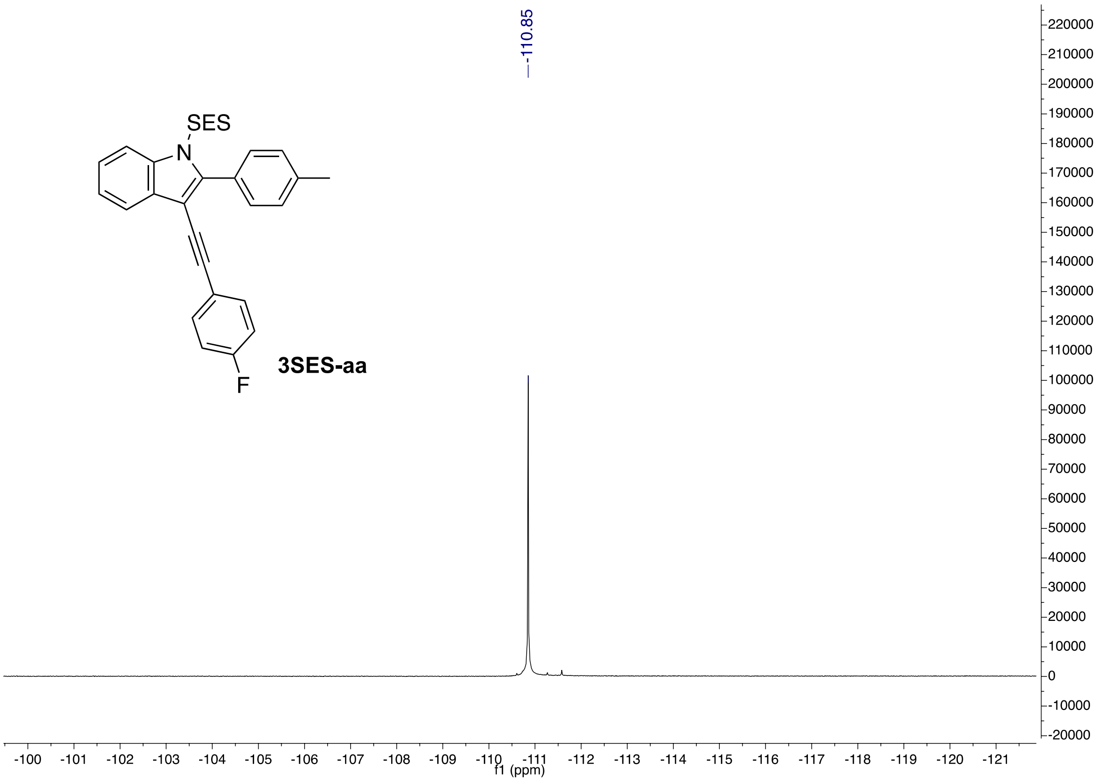

Supplementary Fig. 108 ^{1}H NMR, ^{13}C NMR and ^{19}F NMR spectra of compound **3SES-aa**

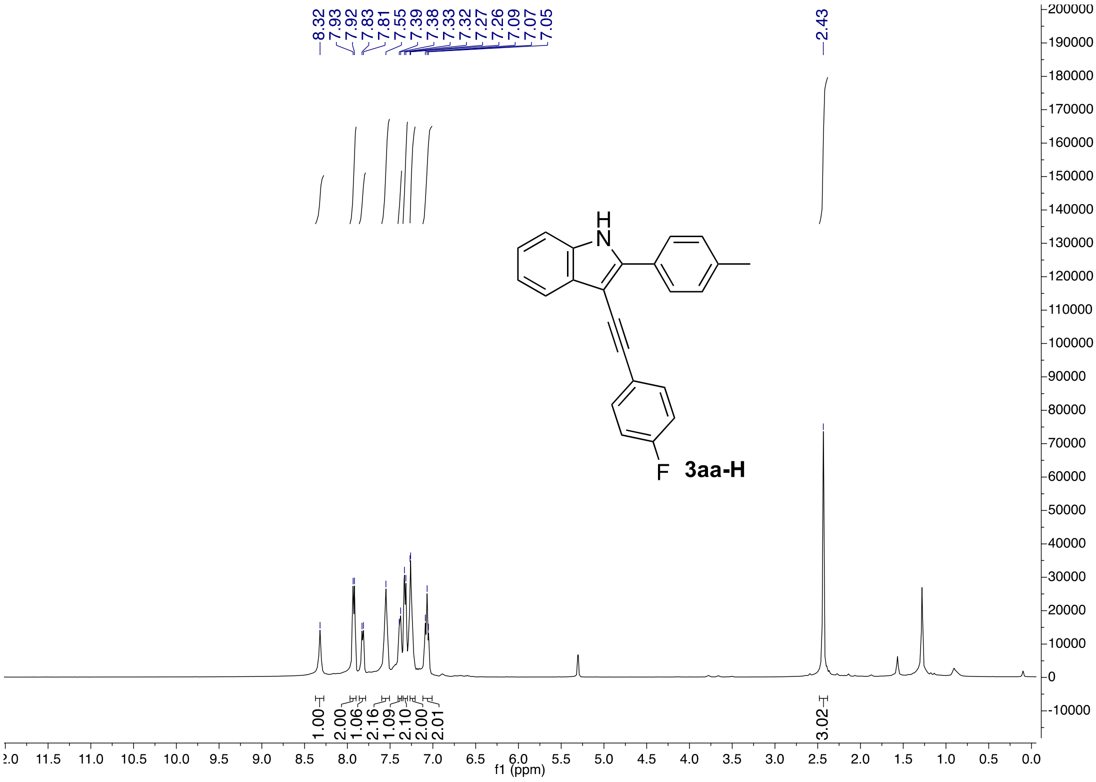
8.32
7.93
7.92
7.83
7.81
7.55
7.39
7.38
7.33
7.32
7.27
7.26
7.09
7.07
7.05
2.43
1.00
2.00
1.06
2.16
1.09
2.10
2.00
2.01
3.02
3aa-H
f1 (ppm)

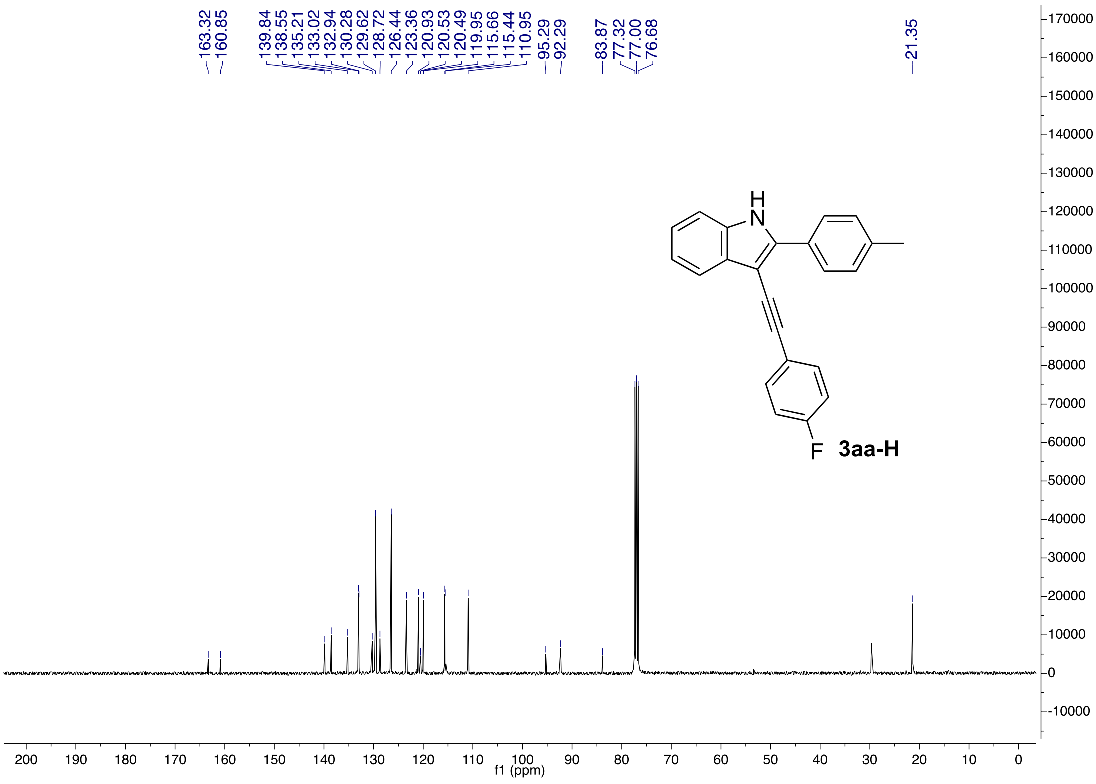
163.32
160.85
139.84
138.55
135.21
133.02
132.94
130.28
129.62
128.72
126.44
123.36
120.93
120.53
120.49
119.95
115.66
115.44
110.95
95.29
92.29
83.87
77.32
77.00
76.68
21.35
3aa-H
f1 (ppm)

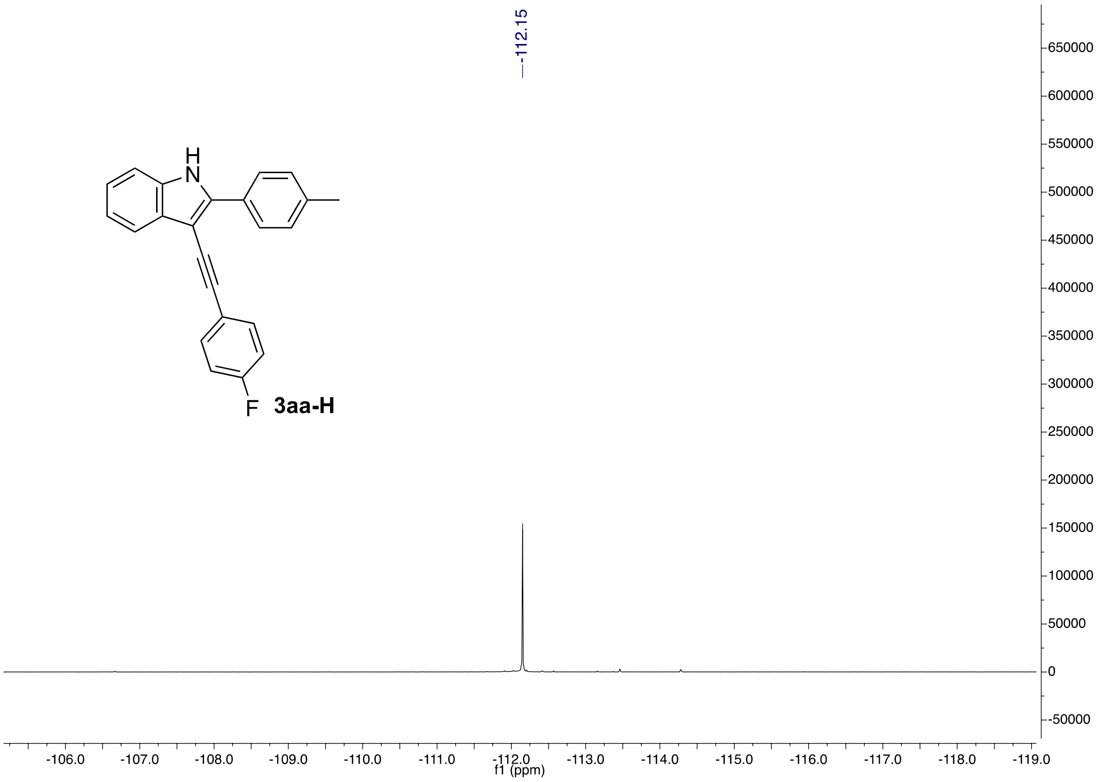

Supplementary Fig. 109 ^{1}H NMR, ^{13}C NMR and ^{19}F NMR spectra of compound **3aa-H**

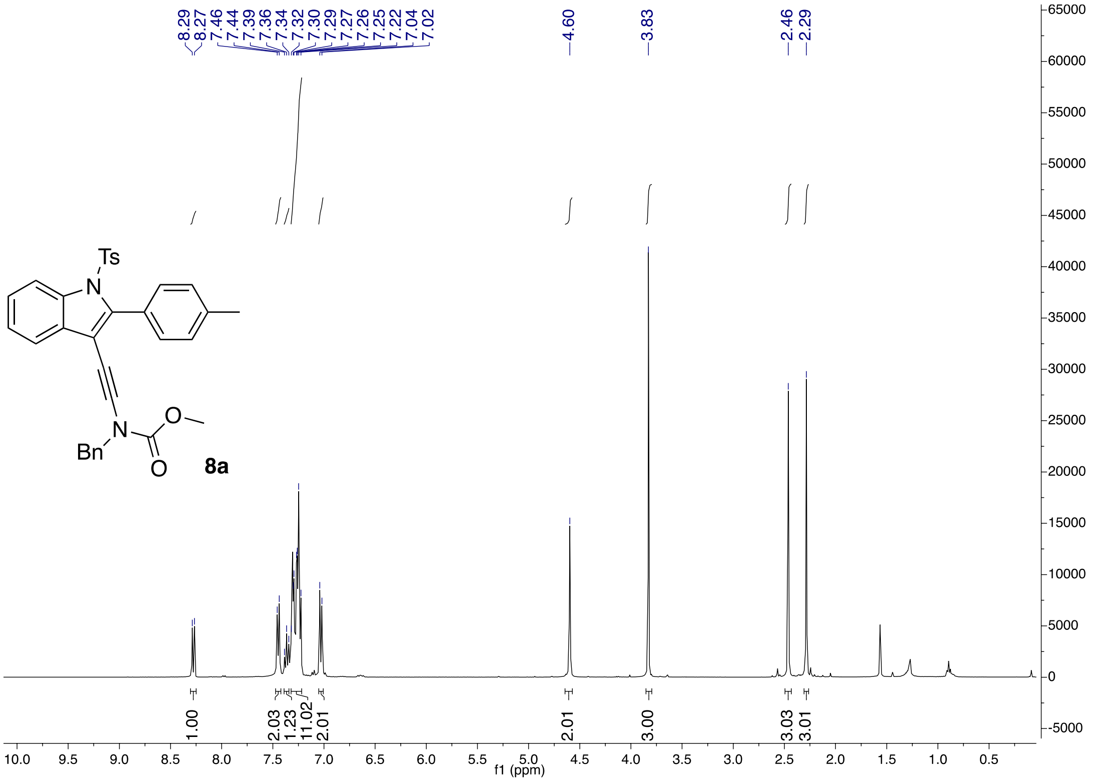

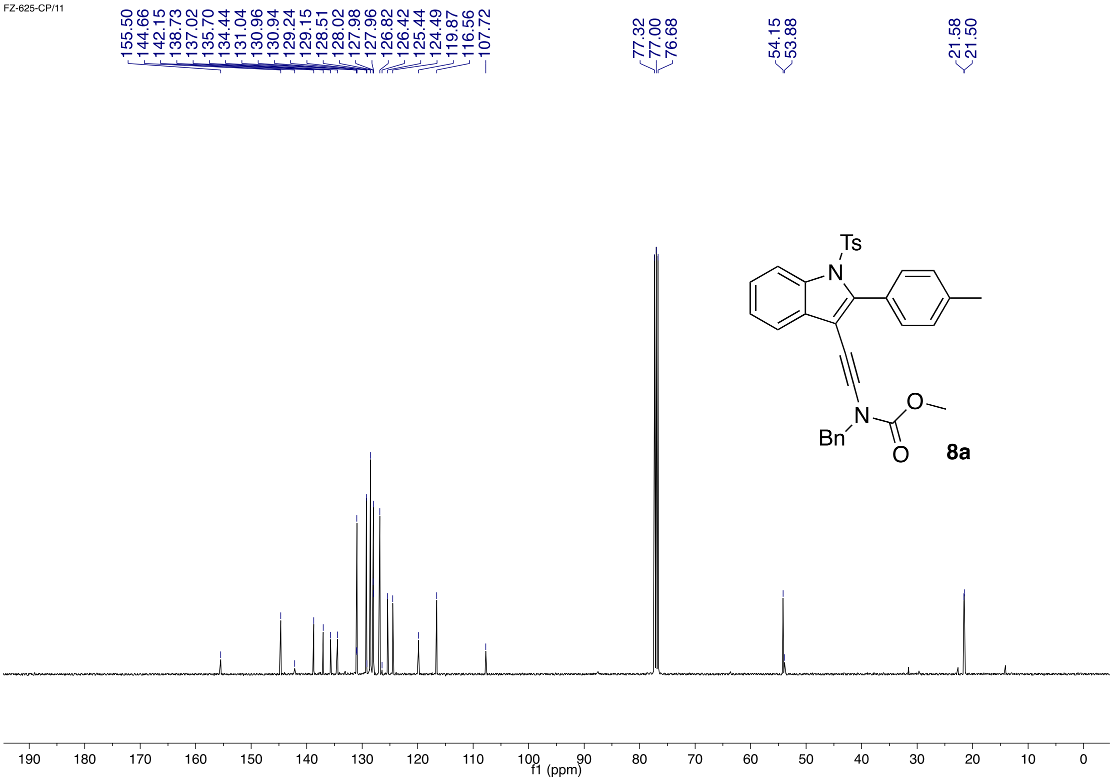

Supplementary Fig. 110 ^{1}H NMR and ^{13}C NMR spectra of compound **8a**

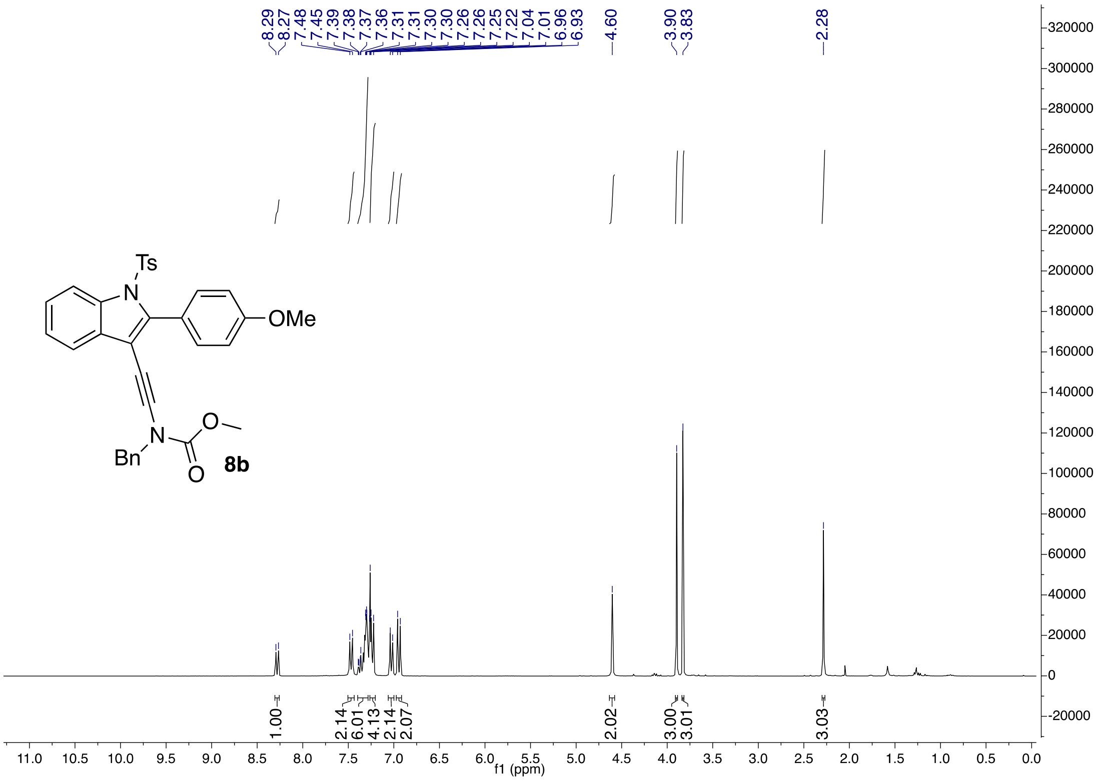

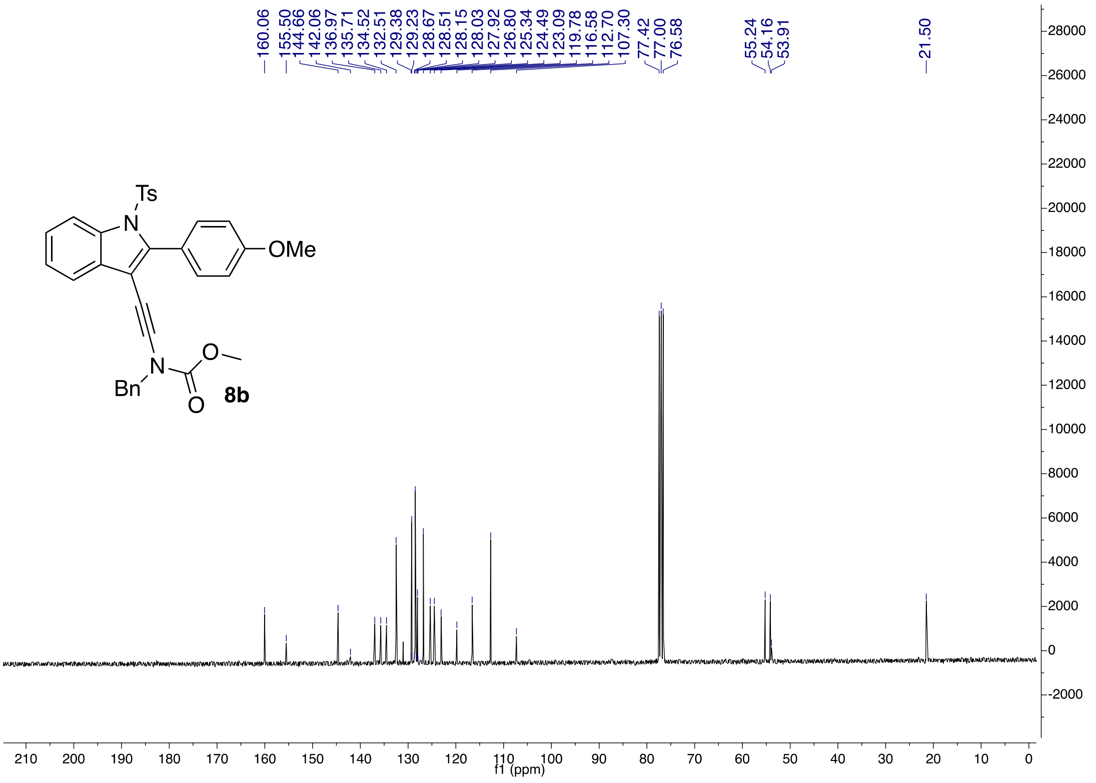

Supplementary Fig. 111 ^{1}H NMR and ^{13}C NMR spectra of compound **8b**

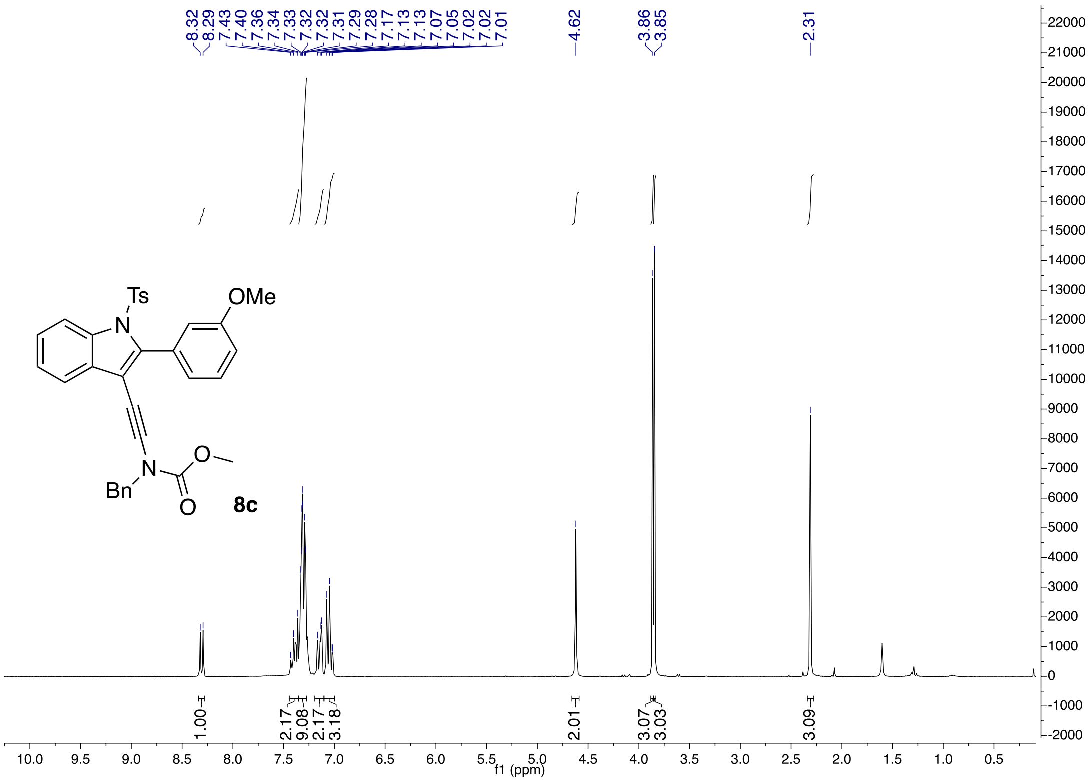

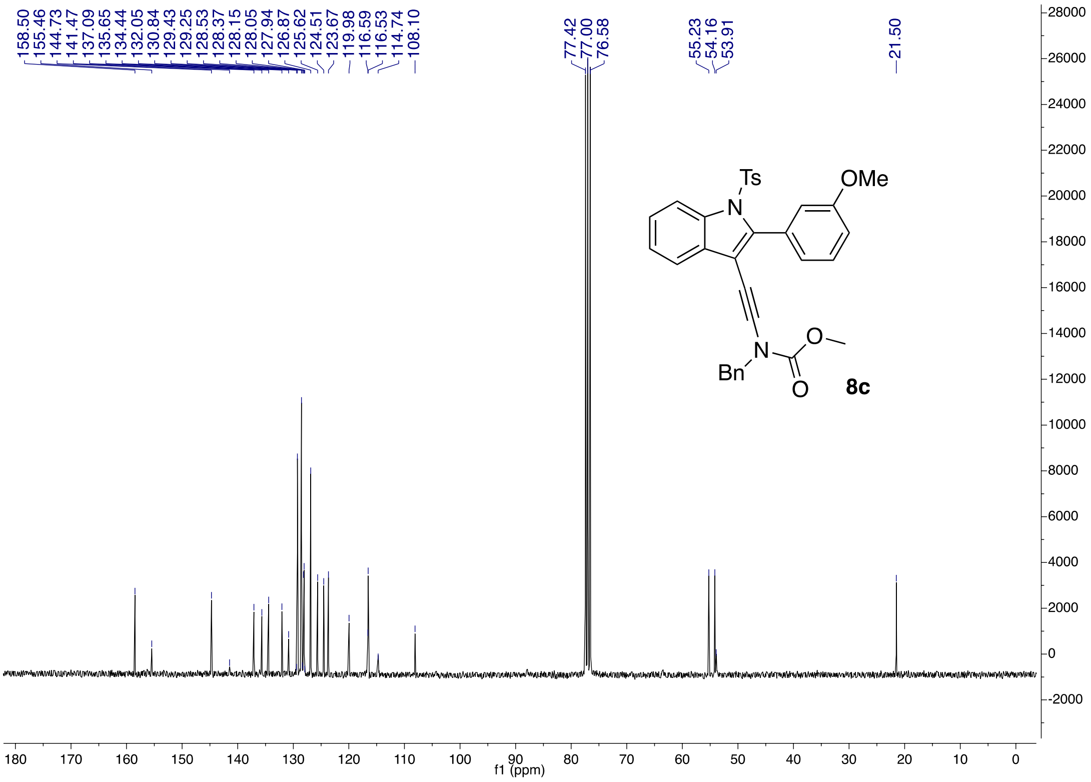

Supplementary Fig. 112 ^{1}H NMR and ^{13}C NMR spectra of compound **8c**

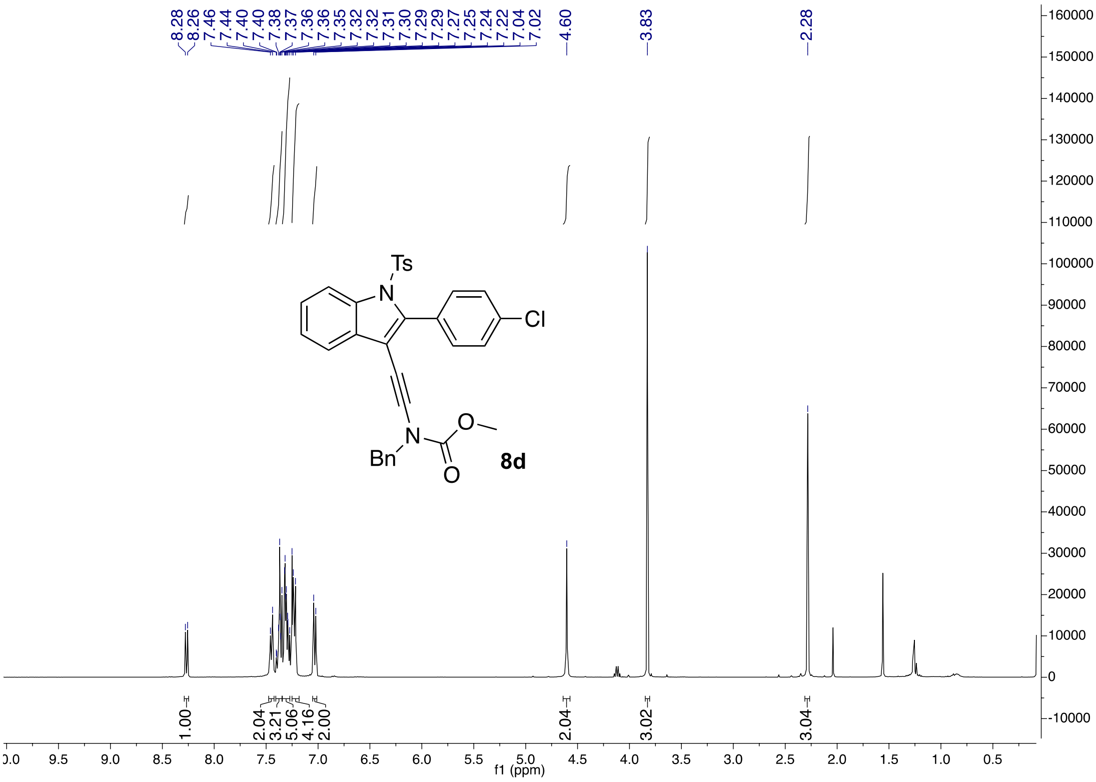

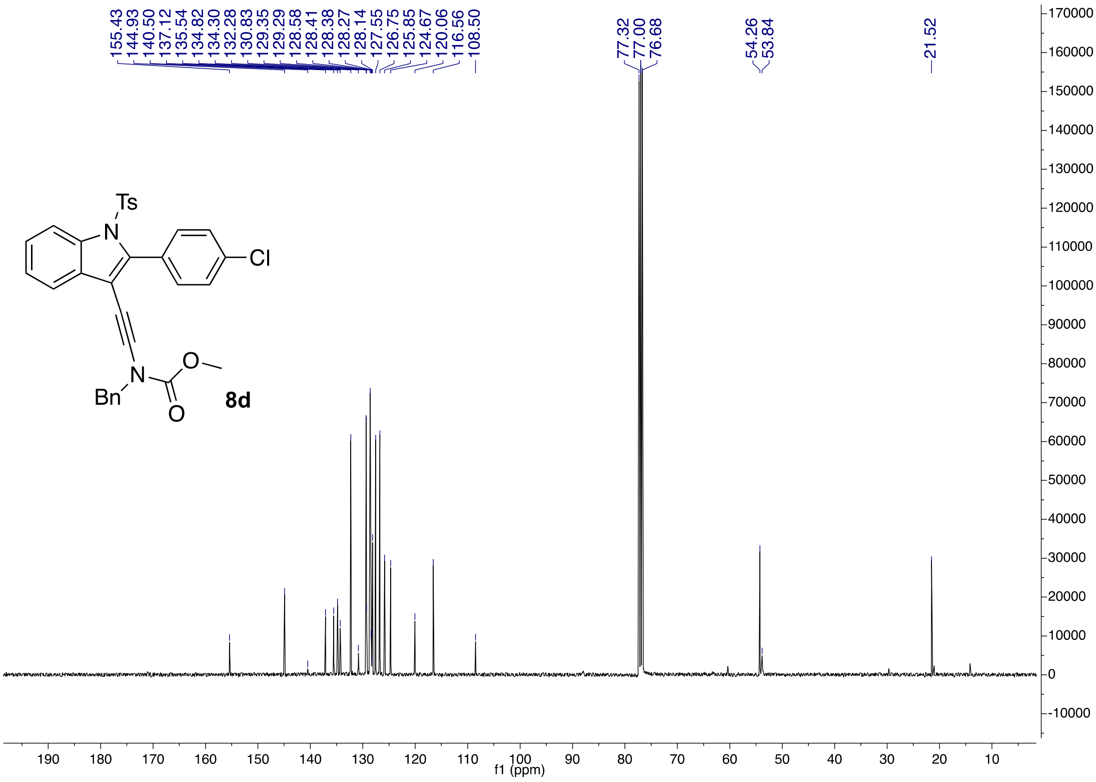

Supplementary Fig. 113 ^{1}H NMR and ^{13}C NMR spectra of compound **8d**

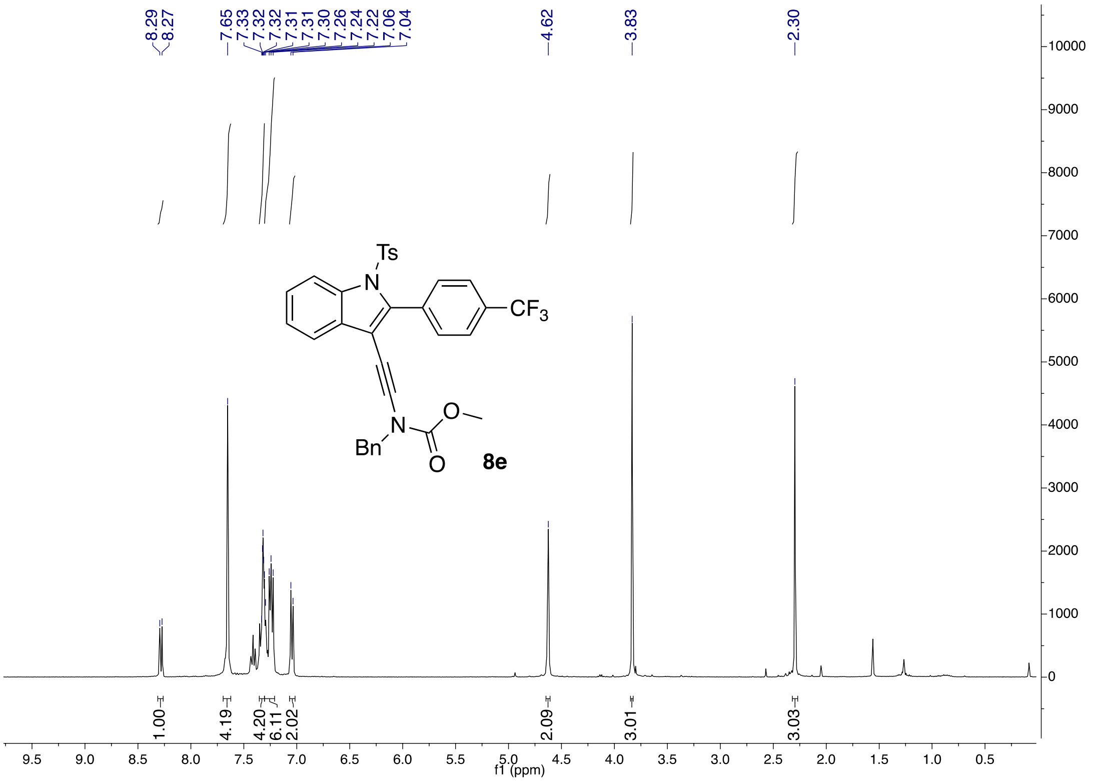
8e
Ts
N
CF3
O
Bn
f1 (ppm)

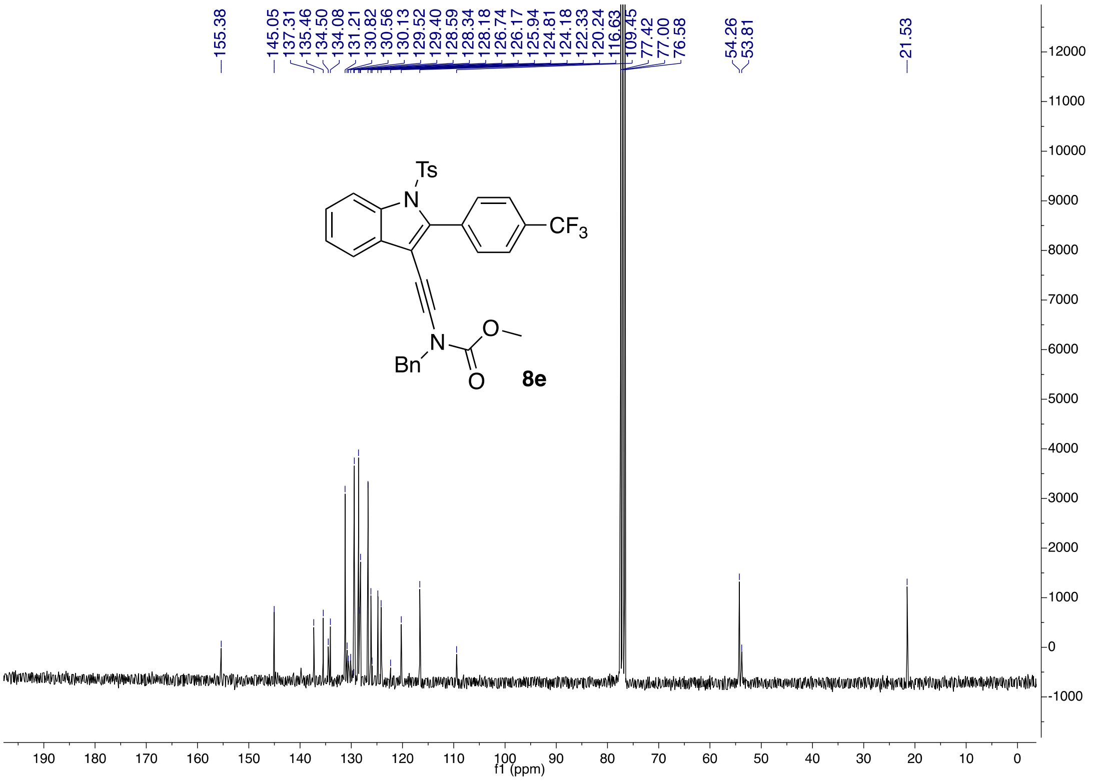
8e
Ts
N
CF3
O
Bn
f1 (ppm)

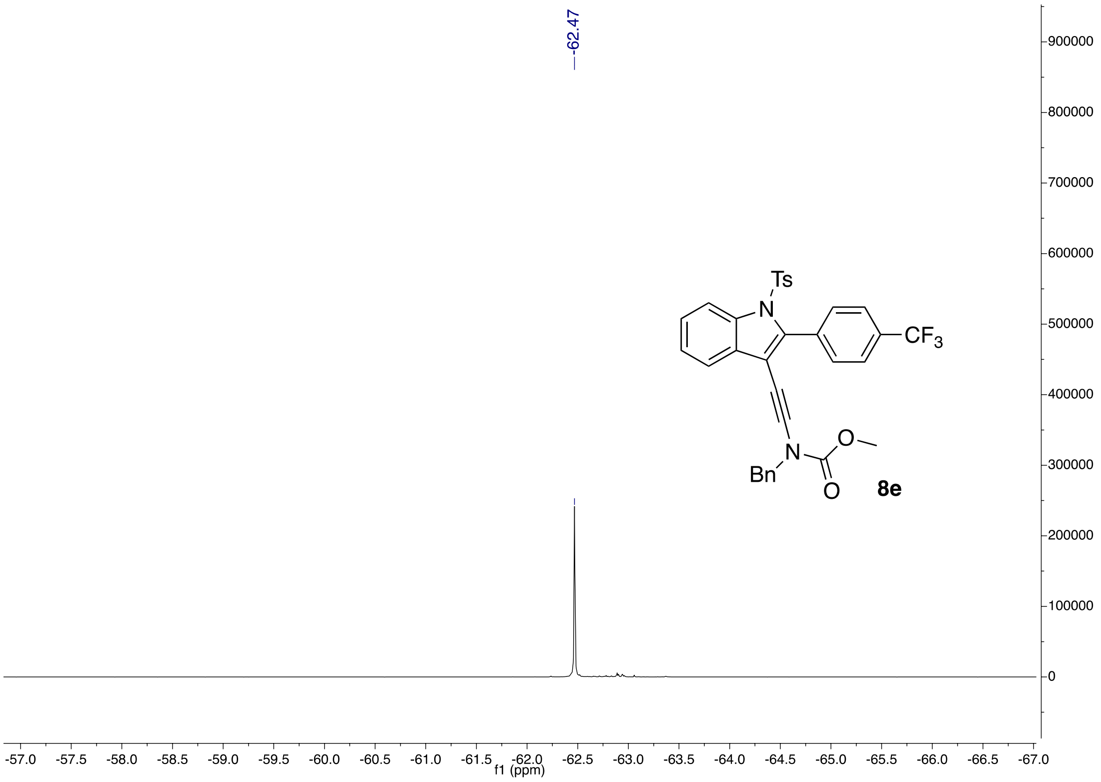

Supplementary Fig. 114 ^{1}H NMR, ^{13}C NMR and ^{19}F NMR spectra of compound **8e**

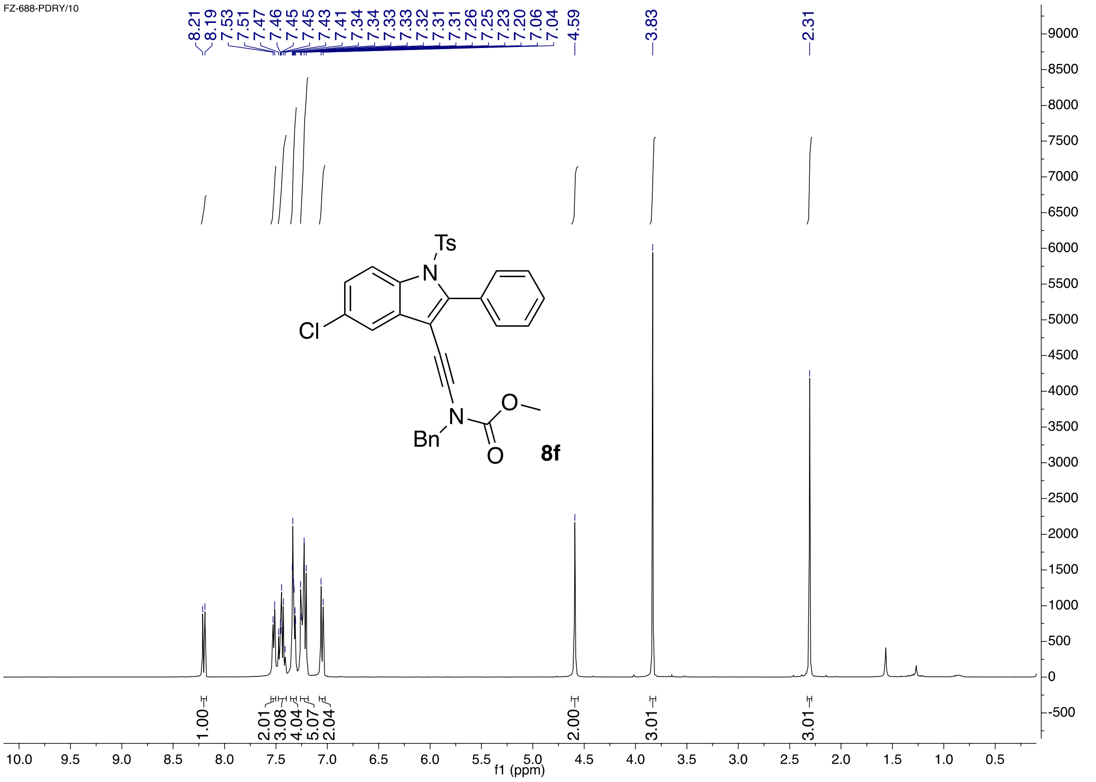

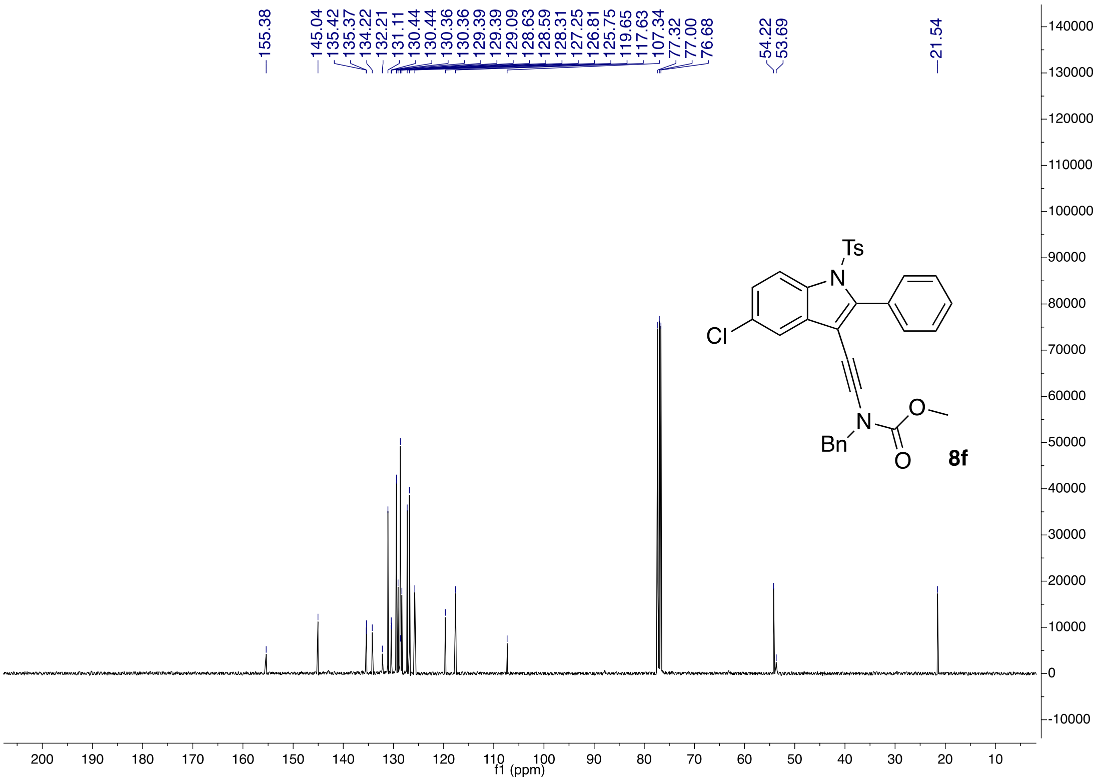

Supplementary Fig. 115 ^{1}H NMR and ^{13}C NMR spectra of compound **8f**

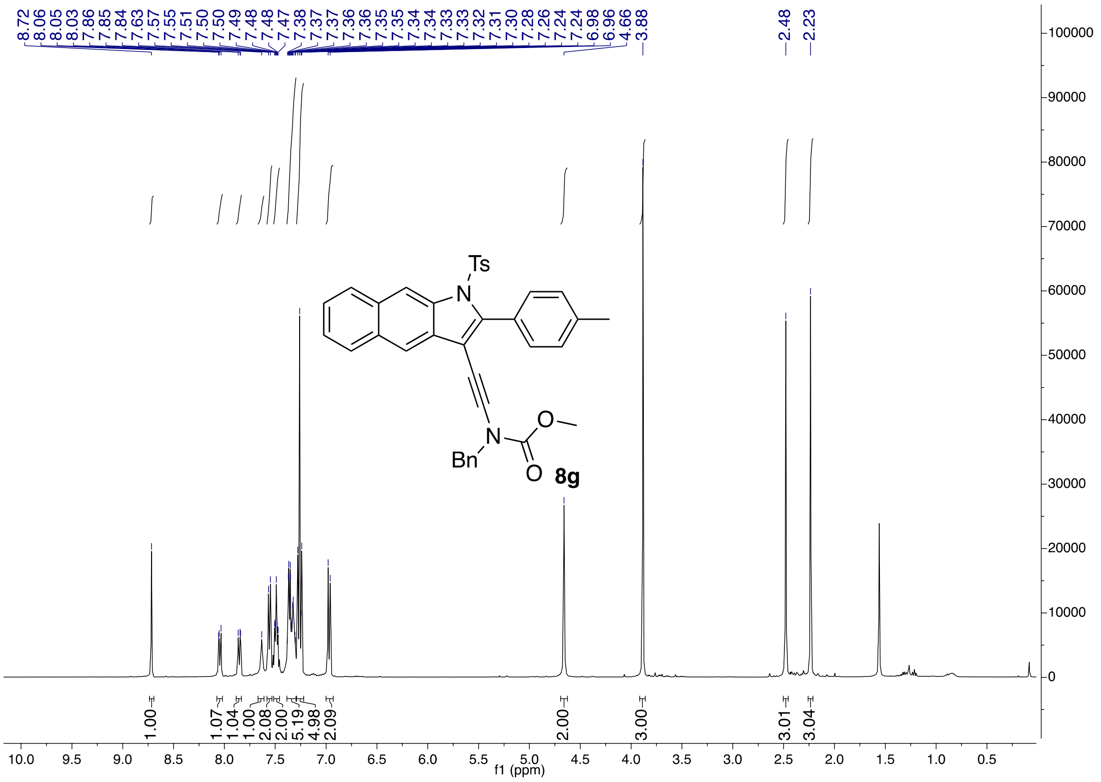

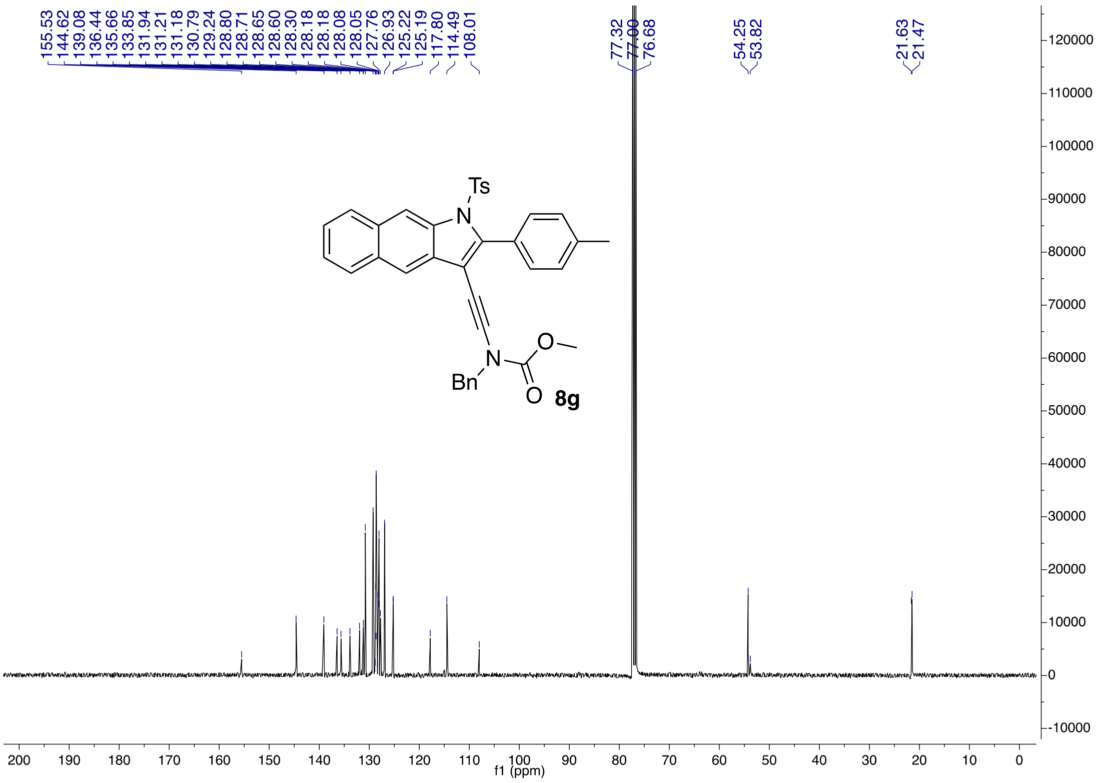

Supplementary Fig. 116 ^{1}H NMR and ^{13}C NMR spectra of compound **8g**

- Post-functionalization

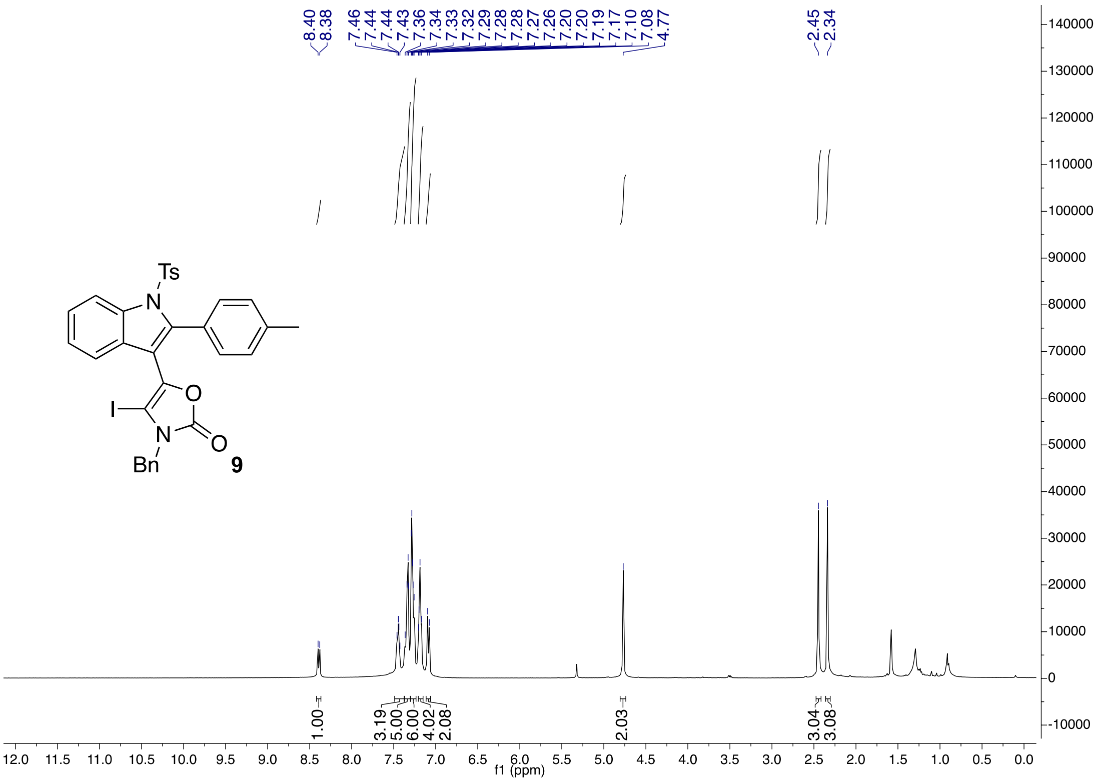

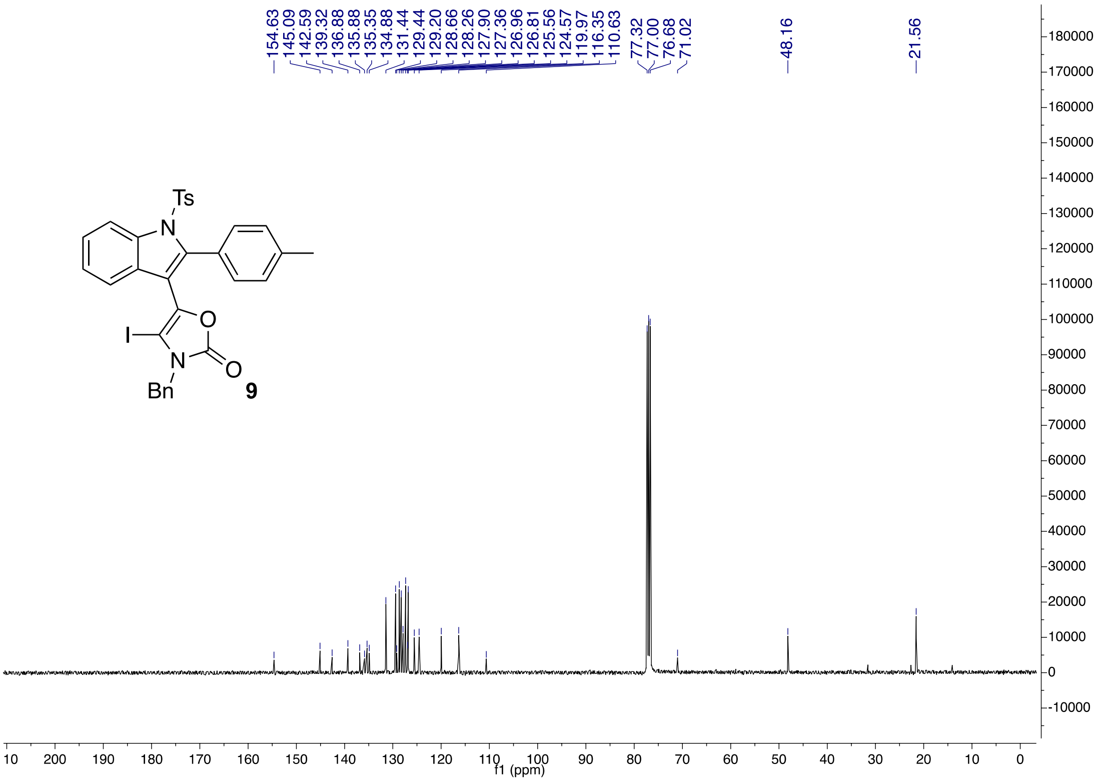

Supplementary Fig. 117 ^{1}H NMR and ^{13}C NMR spectra of compound **9**

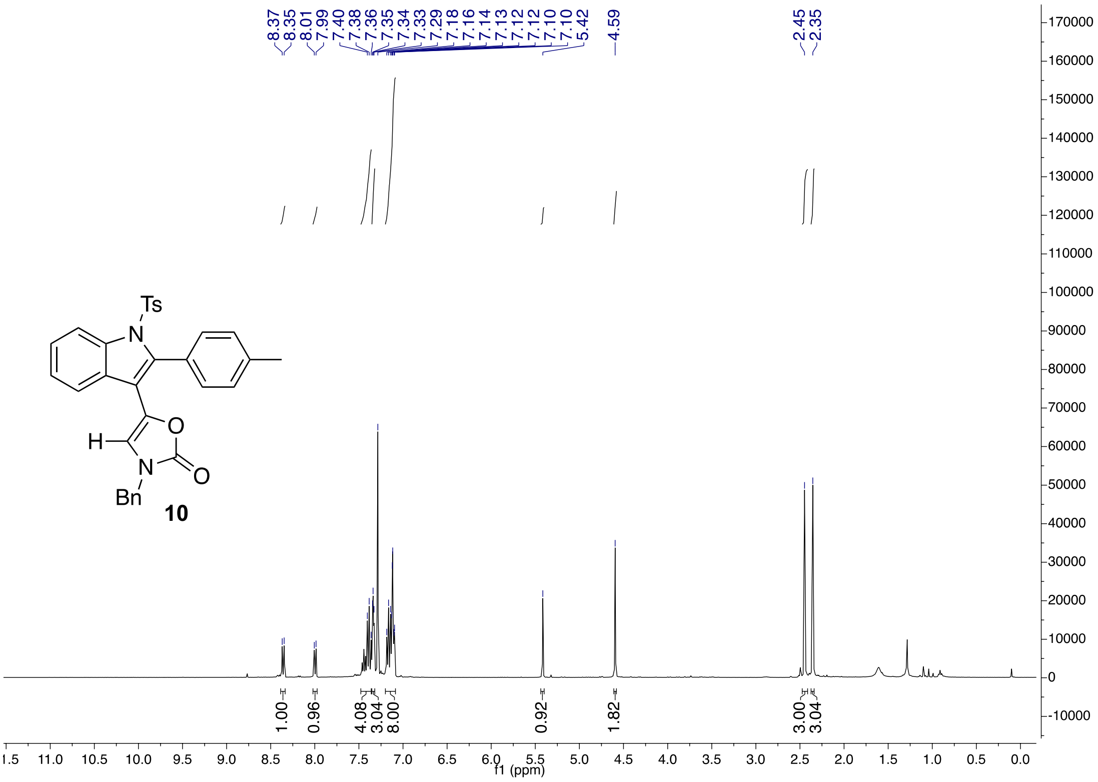

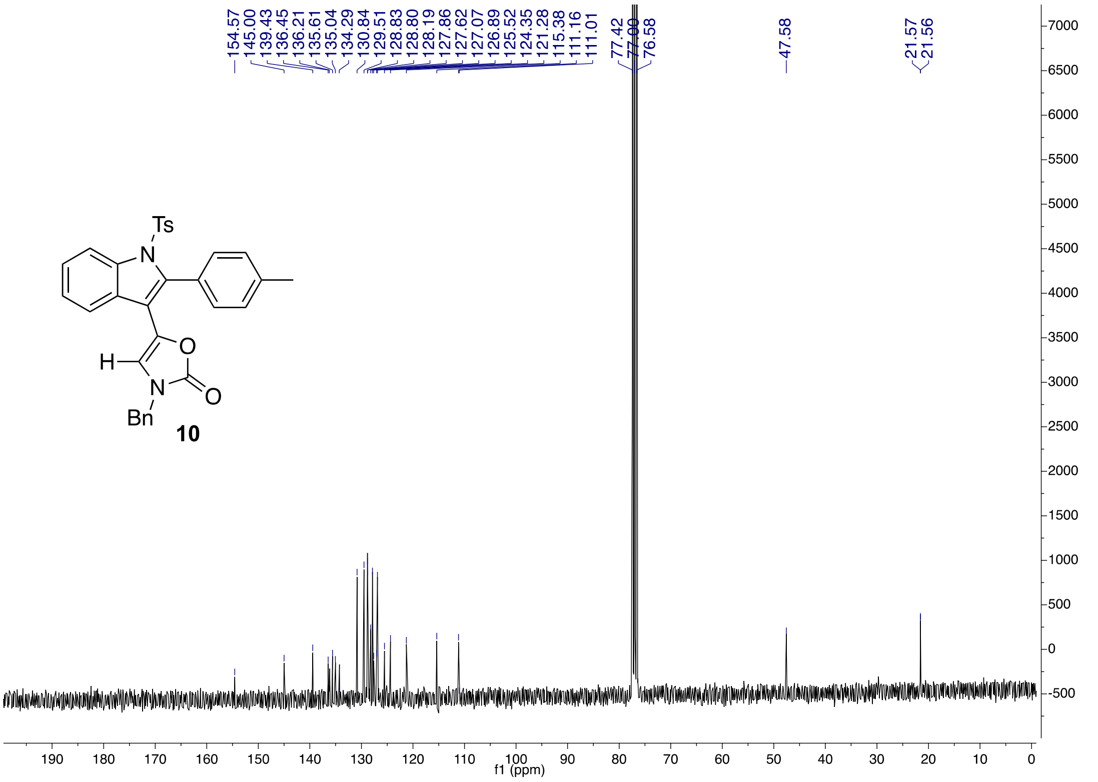

Supplementary Fig. 118 ^{1}H NMR and ^{13}C NMR spectra of compound **10**

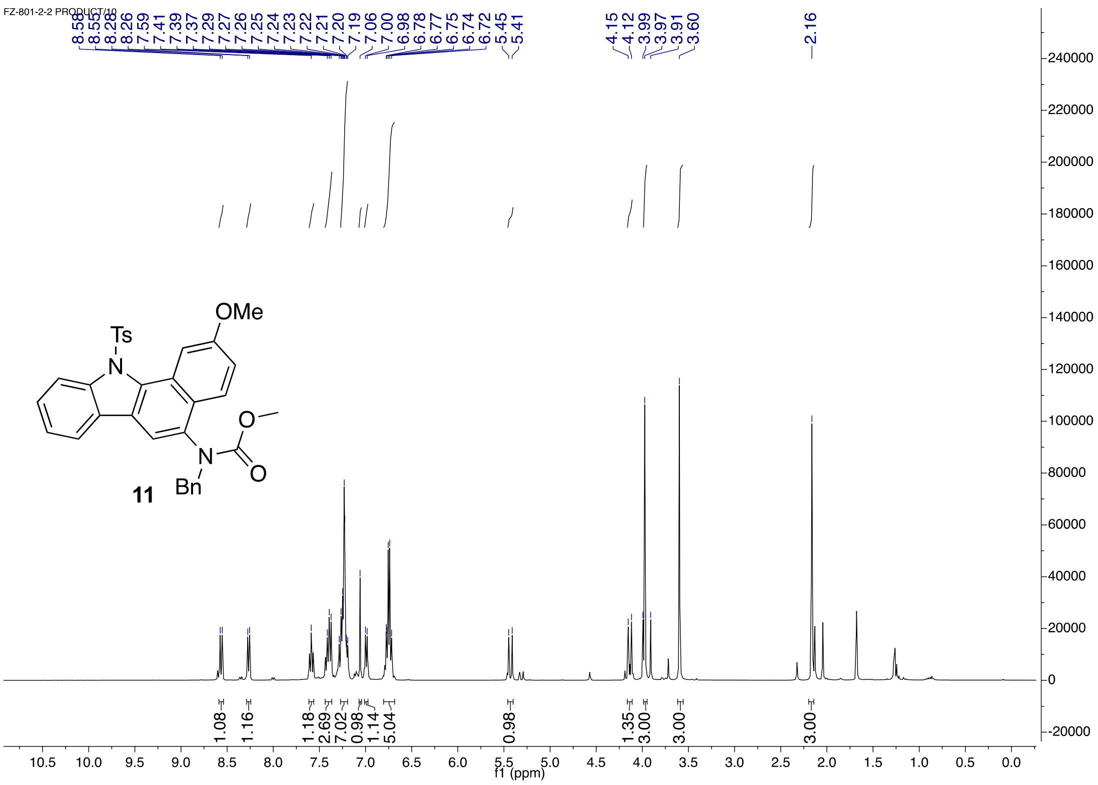

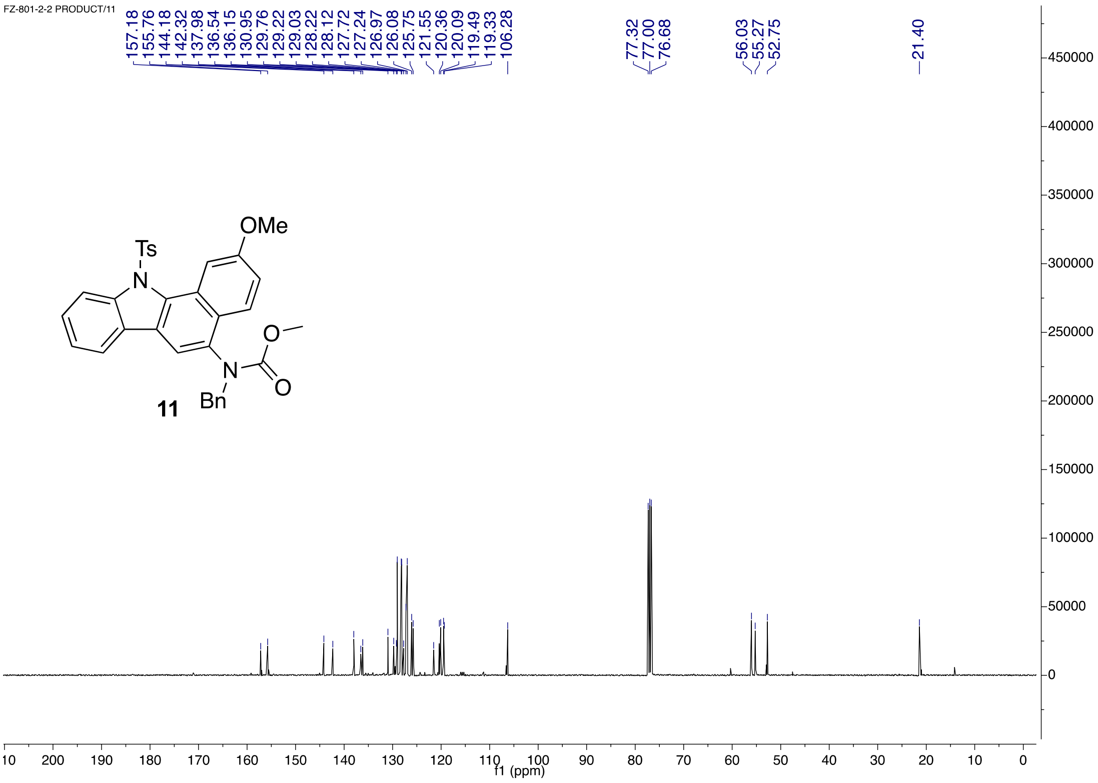

Supplementary Fig. 119 ^{1}H NMR and ^{13}C NMR spectra of compound **11**

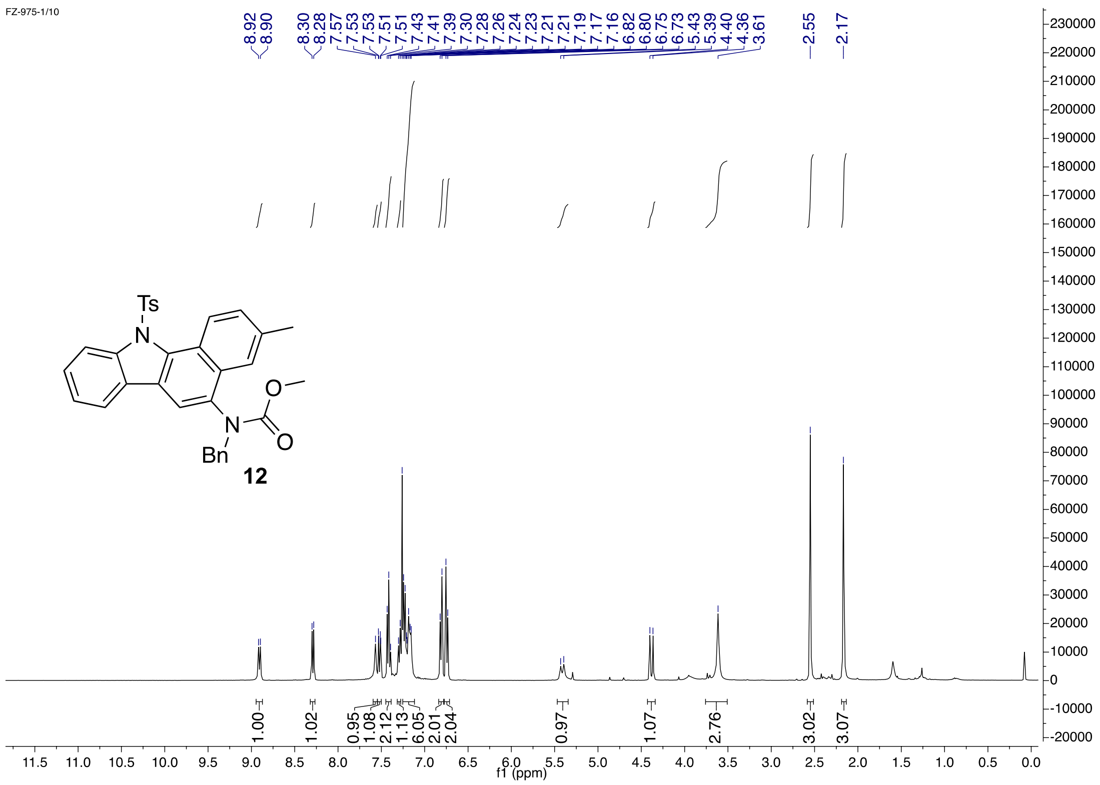

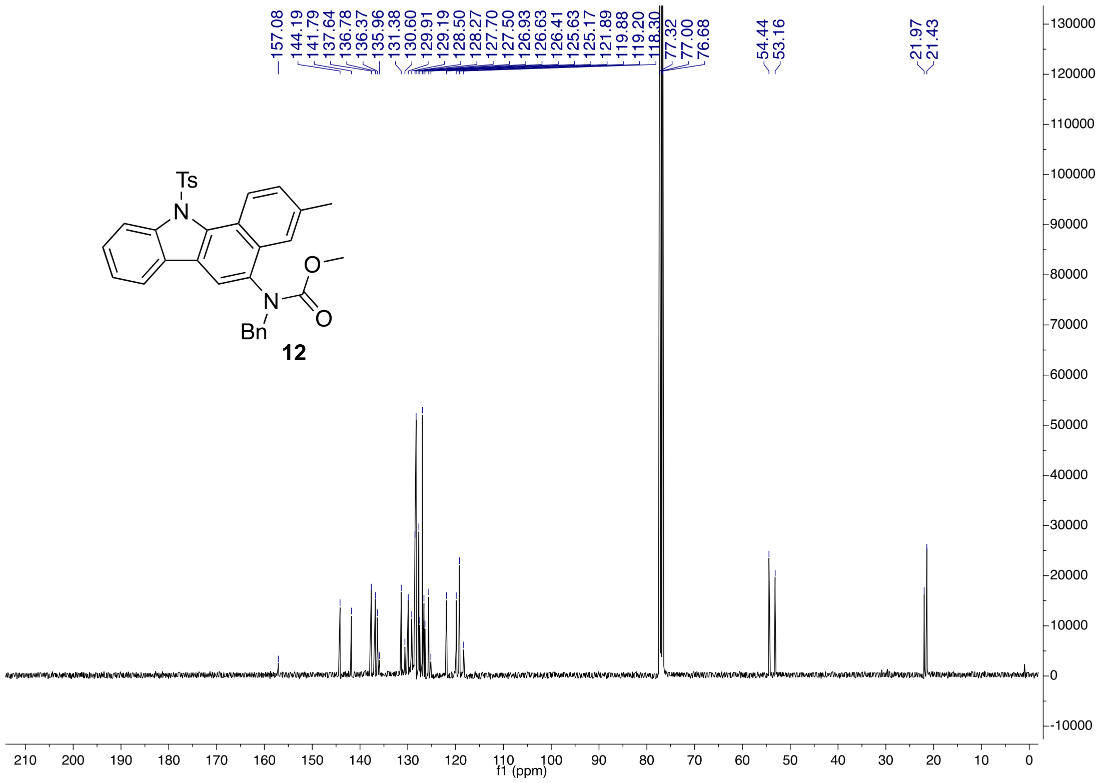

Supplementary Fig. 120 ^{1}H NMR and ^{13}C NMR spectra of compound **12**

- Side products

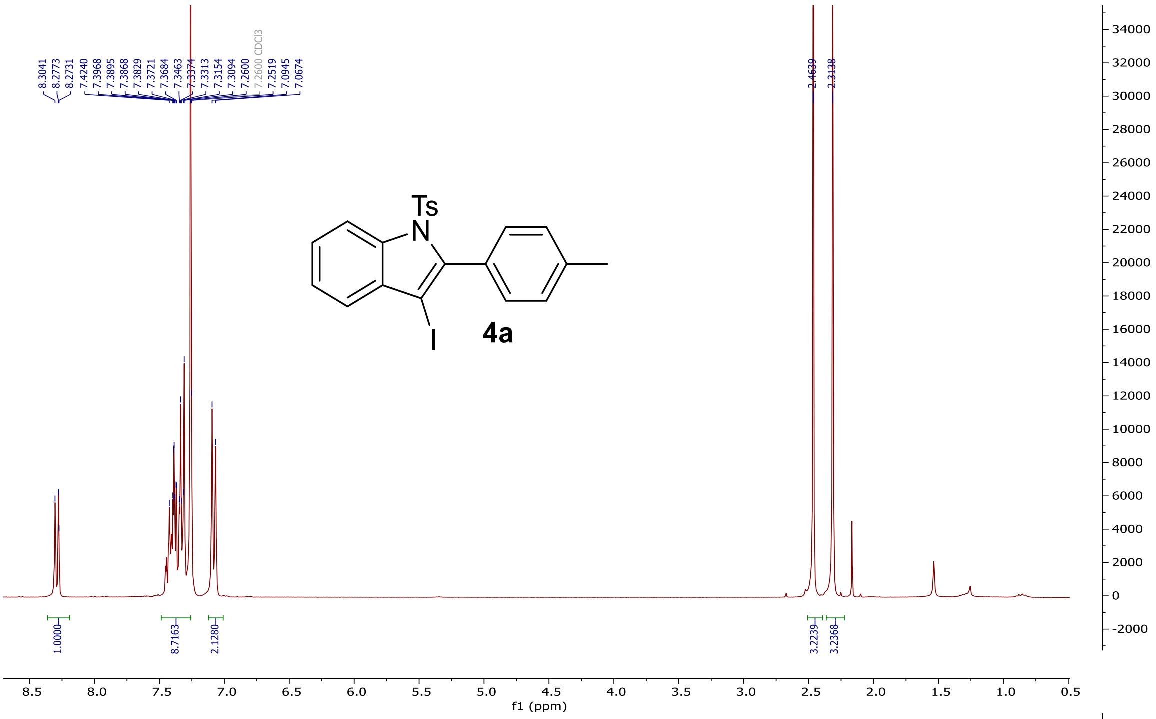

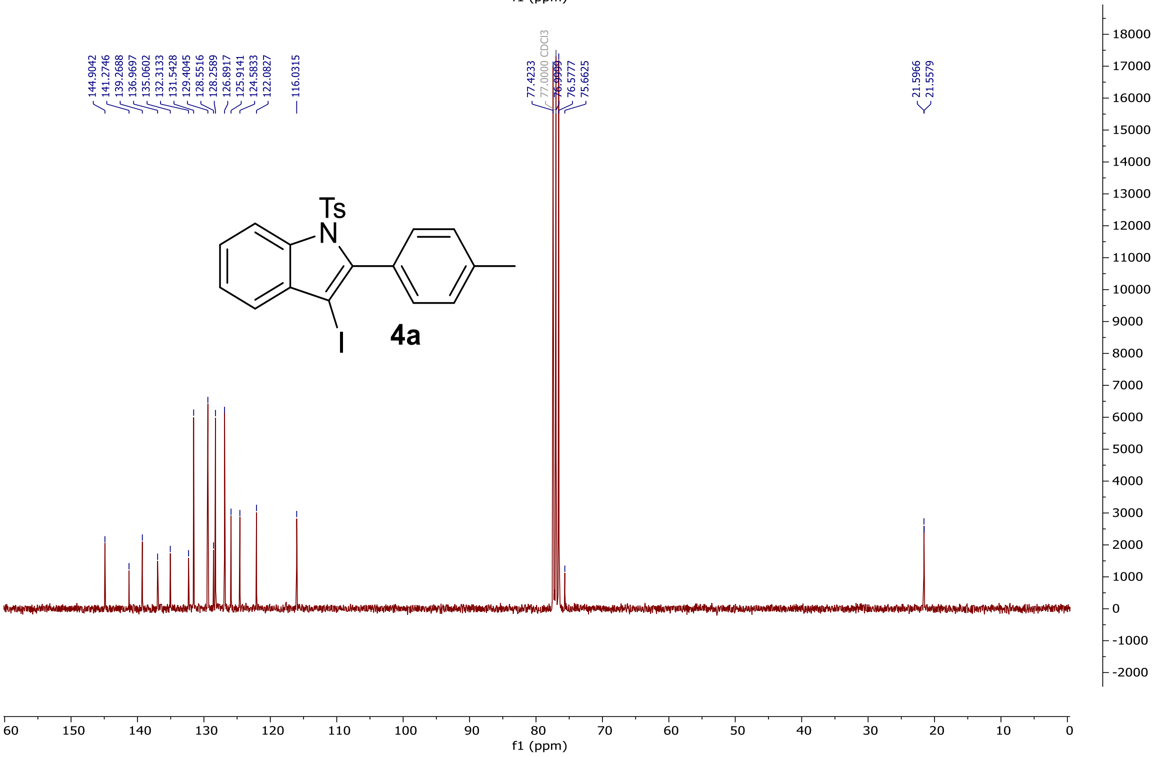

Supplementary Fig. 121 ^{1}H NMR and ^{13}C NMR spectra of compound **4a**

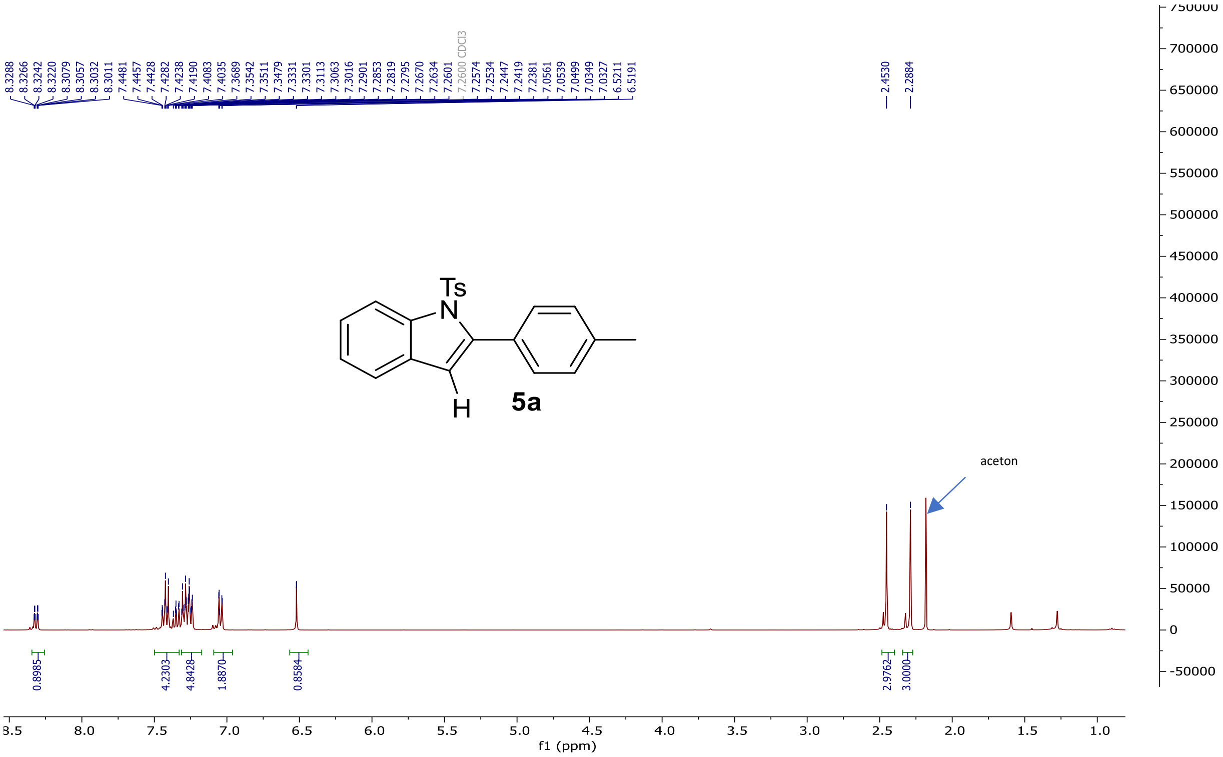

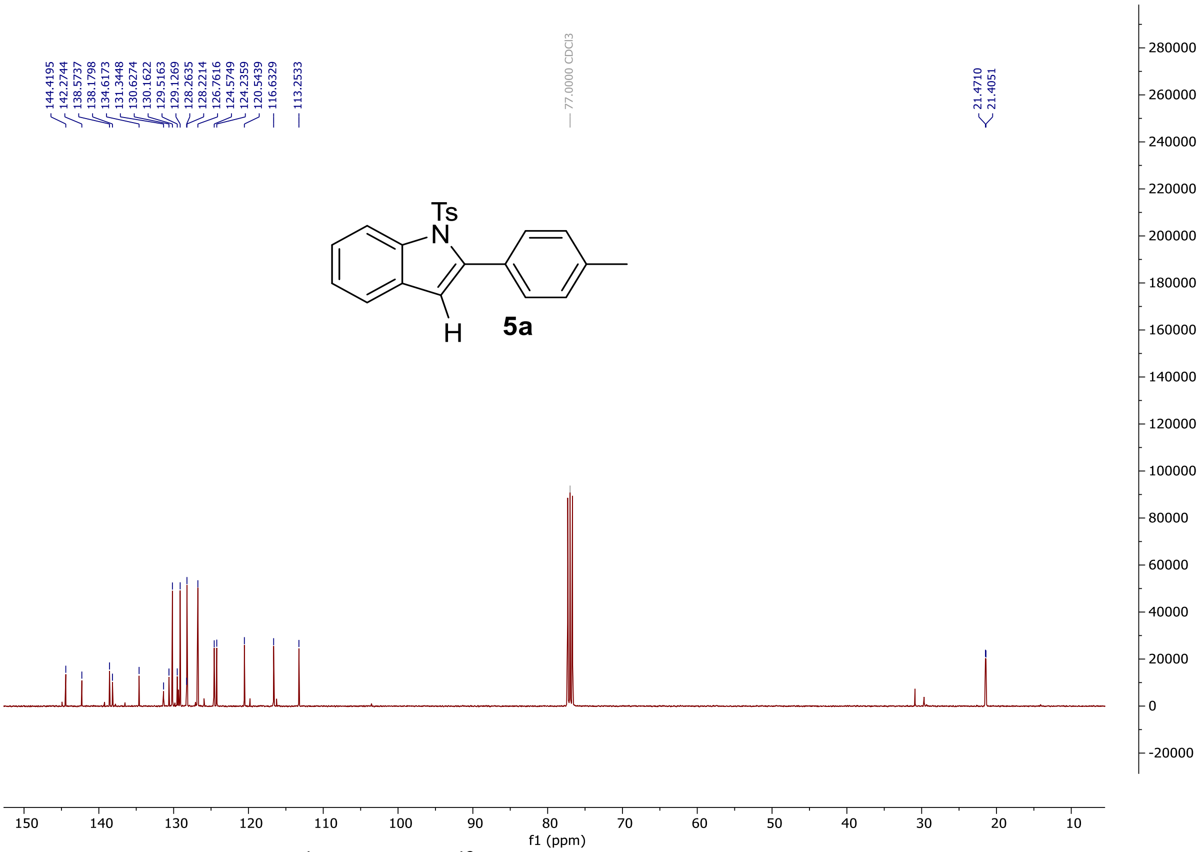

Supplementary Fig. 122 ^{1}H NMR and ^{13}C NMR spectra of compound **5a**

4. X-Ray crystal structure determinations

For compound **8b-monoclinic**, a single crystal was selected, mounted onto a cryoloop and transferred into a cold nitrogen gas stream. Intensity data were collected with a Bruker Kappa APEX-II CCD diffractometer using a micro-focused Cu-Kα radiation (λ = 1.54178 Å). Data collection was performed at 200K, with the Bruker APEXIII suite. Unit-cell parameters determination, integration and data reduction were carried out with SAINT program. SADABS was used for scaling and absorption correction. The structure was solved with SHELXT[39] and refined by full-matrix least-squares methods with SHELXL[40] using Olex2 software package[41]. All non-hydrogen atoms were refined anisotropically.
For the other compounds **3aa**, **6**, **10**, **12**, **8b-triclinic**, **1a-K**, a single crystal of each compound was selected, mounted onto a cryoloop and transferred into a cold nitrogen gas stream. Intensity data were collected with a Bruker Kappa APEX-II CCD diffractometer using a graphite-monochromated Mo-Kα radiation (λ = 0.71073 Å). Data collections were performed at 200K with the Bruker APEXIII suite. Unit-cell parameters determinations, integrations and data reductions were carried out with SAINT program. SADABS was used for scaling and absorption corrections. The structures were solved with SHELXT[40] and refined by full-matrix least-squares methods with SHELXL[40] using Olex2 software package[41] (**3aa**, **12**, **8b** and **1a-K**) or WinGX suite[42] (**6** and **10**). All non-hydrogen atoms were refined anisotropically.

These structures were deposited at the Cambridge Crystallographic Data Centre with numbers CCDC 2090314 to 2090317 and 2090321 to 2090323, and can be obtained free of charge via www.ccdc.cam.ac.uk.

Supplementary Table 5 Crystallographic data for compounds **1a, 3aa, 6 and 11**

	**1a**	**3aa**	**6**	**11**
CCDC deposit number	**2150836**	**2090317**	**2090321**	**2090322**
Empirical formula[a]	$C_{22}H_{19}NO_2S$	$C_{30}H_{22}FNO_2S$	$C_{43}H_{30}NO_2F_9PSAu$	$C_{32}H_{26}N_2O_4S$
F.W. [g mol^{-1}]	361.44	479.54	1023.67	534.61
Crystal system	Triclinic	Monoclinic	Monoclinic	Monoclinic
Space group	*P-1*	*C2/c*	$P2_1/n$	*C2/c*
a [Å]	9.8180(4)	35.056(2)	11.6947(8)	21.1818(5)
b [Å]	10.4210(4)	7.9873(5)	17.3981(10)	8.6122(2)
c [Å]	11.1983(5)	18.5380(11)	20.6279(14)	28.8077(7)
α [°]	93.874(2)	90	90	90
β [°]	107.437(2)	110.312(3)	103.444(3)	91.187(2)
γ [°]	117.201(2)	90	90	90
V [Å^3]	943.53(7)	4868.0(5)	4082.1(5)	5254.0(2)
Z	2	8	4	8
T [K]	199.98	200(1)	200(1)	200(1)
λ [Å]	1.54178	0.71073	0.71073	0.71073
ρ_{calc} [g cm^{-3}]	1.272	1.309	1.666	1.352
μ [mm^{-1}]	1.641 ($Cu_{K\alpha}$)	0.169 ($Mo_{K\alpha}$)	3.773 ($Mo_{K\alpha}$)	0.165 ($Mo_{K\alpha}$)
F(000)	380.0	2000.0	2008.0	2240.0
Measured reflections	18829	43674	48522	42020
Unique reflections	3339	7551	10138	8020
R_{int}	0.0367	0.0328	0.0231	0.0237
R_{sigma}	0.0221	0.0230	0.0191	0.0175
Reflections I>2σ(I)	2939	5843	8502	6701
Parameters	241	318	525	354
Restraints	1	0	0	0
R_1^b [I>2σ(I)]	0.0359	0.0411	0.0293	0.0510
wR_2^c [I>2σ(I)]	0.0980	0.1089	0.0684	0.1314
R_1^b [all data]	0.0414	0.0577	0.0394	0.0622
wR_2^c [all data]	0.1016	0.1196	0.0732	0.1405
GOF	1.071	1.031	1.022	1.094
Largest residuals [eÅ^3]	-0.44 ; 0.21	-0.35 ; 0.34	-0.67 ; 1.13	-0.25 ; 0.43

[a] Including solvate molecules. [b] $R_1 = \sum \left||F_o| - |F_c|\right| / \sum |F_o|$ [c] $wR_2 = \left[\sum w\left(F_o^2 - F_c^2\right)^2 / \sum w\left(F_o^2\right)^2\right]^{1/2}$

Supplementary Table 6 Crystallographic data for compounds **13**, **8b** and **1a-K**.

	**13**	**8b-triclinic**	**8b-monoclinic**	**1a-K**
CCDC deposit number	**2090314**	**2090316**	**2090315**	**2090323**
Empirical formula[a]	$C_{33}H_{28}N_2O_4S$	$C_{33}H_{28}N_2O_5S$, 0.15(CH_2Cl_2)	$C_{33}H_{28}N_2O_5S$, 0.2(CH_2Cl_2)	$C_{22}H_{18}KNO_2S$
F.W. [g mol^{-1}]	548.63	577.37	581.62	399.53
Crystal system	Triclinic	Triclinic	Monoclinic	Monoclinic
Space group	*P-1*	*P-1*	*C2/c*	$P2_1/n$
a [Å]	11.9616(19)	12.4643(15)	75.043(3)	14.8979(9)
b [Å]	11.9670(19)	13.9395(17)	9.9216(3)	5.3706(3)
c [Å]	12.2619(19)	19.312(2)	24.6860(8)	24.5080(19)
α [°]	100.869(3)	75.518(3)	90	90
β [°]	108.109(2)	87.281(3)	101.413(2)	99.677(5)
γ [°]	116.245(3)	77.399(3)	90	90
V [Å^3]	1382.6(4)	3170.5(7)	18016.5(10)	1933.0(2)
Z	2	4	24	4
T [K]	200(1)	200(1)	200(1)	200(1)
λ [Å]	0.71073	0.71073	1.54178	0.71073
ρ_{calc} [g cm^{-3}]	1.318	1.210	1.287	1.373
μ [mm^{-1}]	0.159 ($Mo_{K\alpha}$)	0.169 ($Mo_{K\alpha}$)	1.643 ($Cu_{K\alpha}$)	0.400 ($Mo_{K\alpha}$)
F(000)	576.0	1209.0	7306.0	832.0
Measured reflections	30735	65102	105985	14954
Unique reflections	6835	11385	15883	3506
R_{int}	0.0591	0.0632	0.0378	0.1176
R_{sigma}	0.0521	0.0521	0.0241	0.1086
Reflections I>2σ(I)	4634	7508	13673	1988
Parameters	363	901	1327	282
Restraints	0	286	441	0
R_1[b] [I>2σ(I)]	0.0464	0.0922	0.0478	0.0544
wR_2[c] [I>2σ(I)]	0.1018	0.2517	0.1320	0.0895
R_1[b] [all data]	0.0834	0.1356	0.0555	0.1260
wR_2[c] [all data]	0.1176	0.2790	0.1382	0.1087
GOF	1.015	1.116	1.038	0.996
Largest residuals [eÅ^3]	-0.43 ; 0.28	-0.33 ; 0.89	-0.34 ; 0.77	-0.32 ; 0.27

[a] Including solvate molecules. [b] $R_1 = \sum \left||F_o| - |F_c|\right| / \sum |F_o|$ [c] $wR_2 = \left[\sum w\left(F_o^2 - F_c^2\right)^2 / \sum w\left(F_o^2\right)^2 \right]^{1/2}$

X-RAY CRYSTAL STRUCTURES

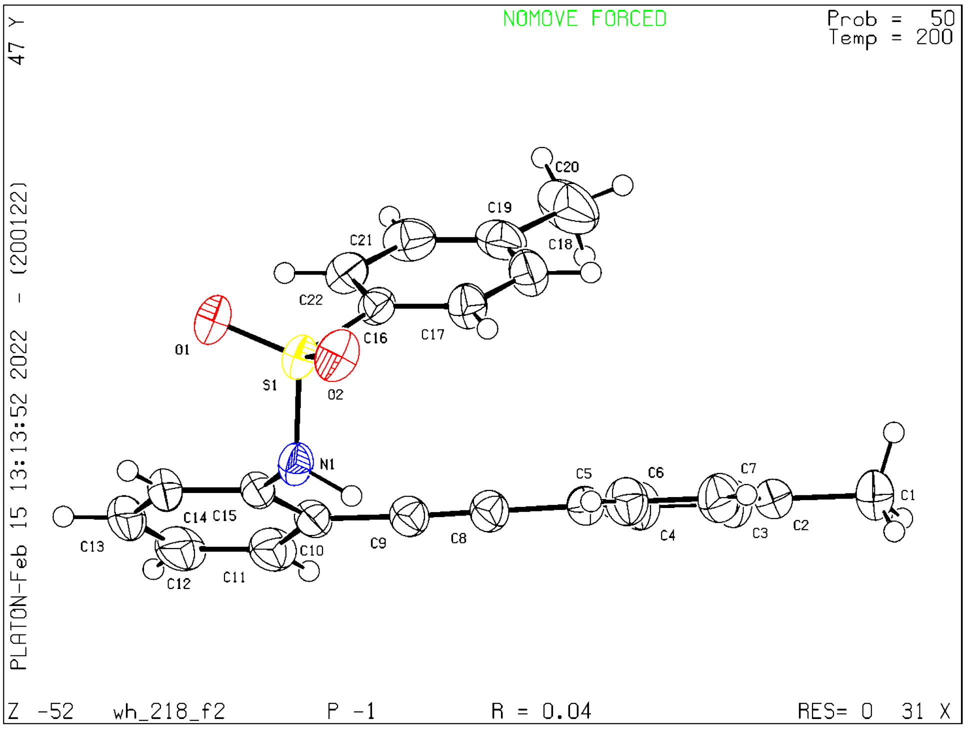

Supplementary Fig. 123 Crystal structure of **1a**

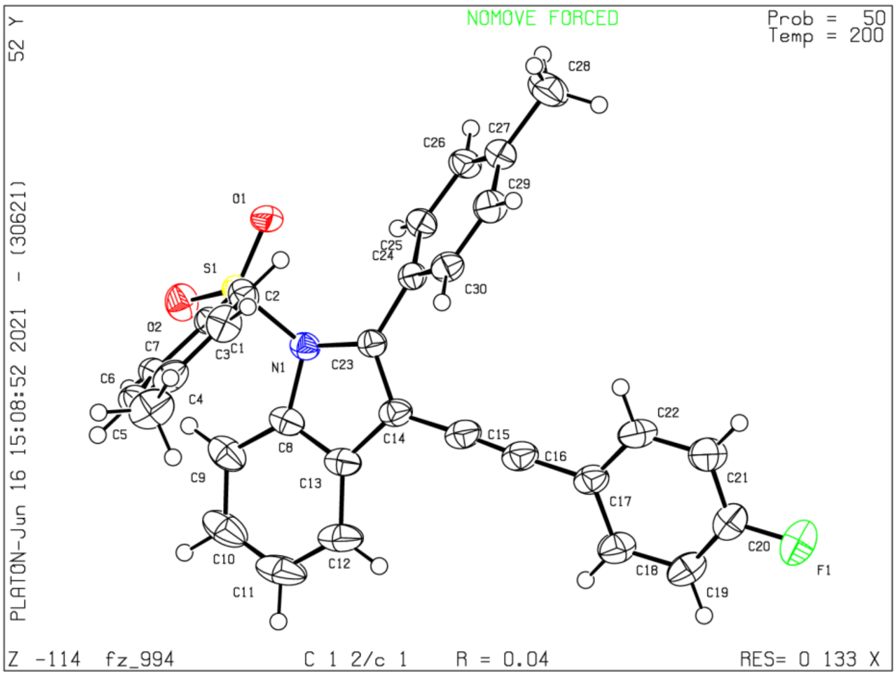

Supplementary Fig. 124 Crystal structure of **3aa**

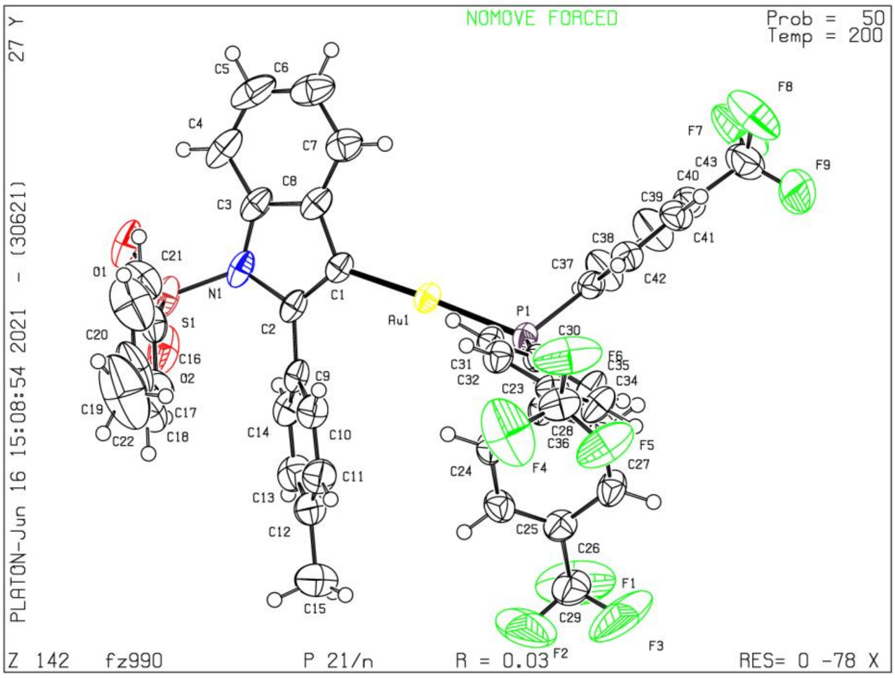

Supplementary Fig. 125 Crystal structure of **6**

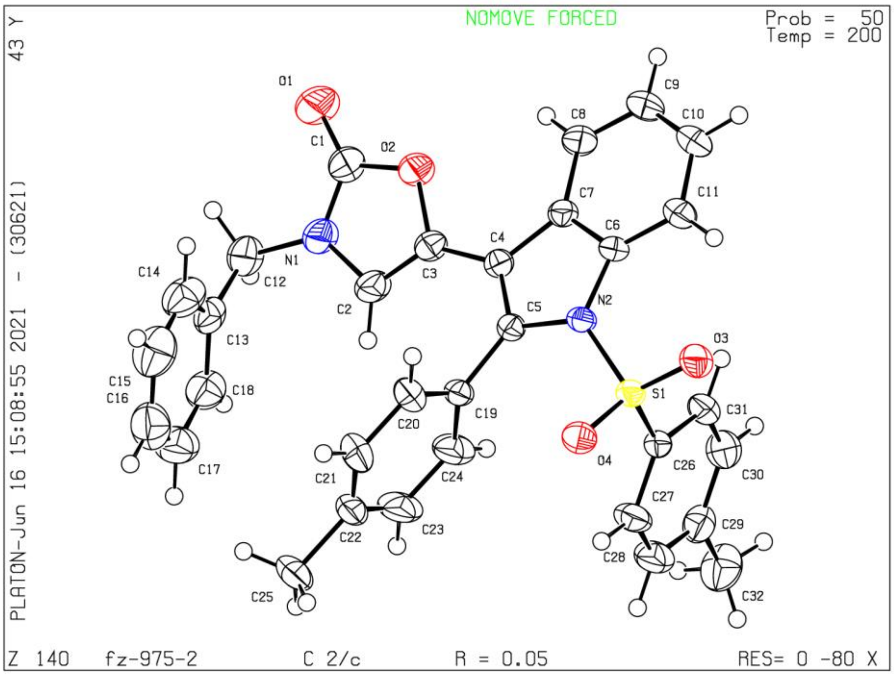

Supplementary Fig. 126 Crystal structure of **10**

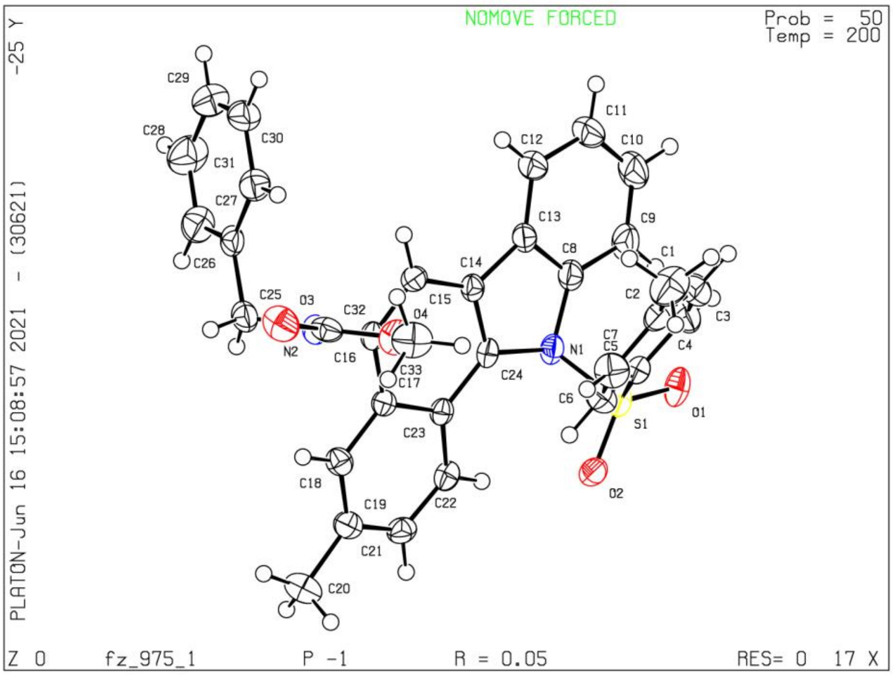

Supplementary Fig. 127 Crystal structure of **12**

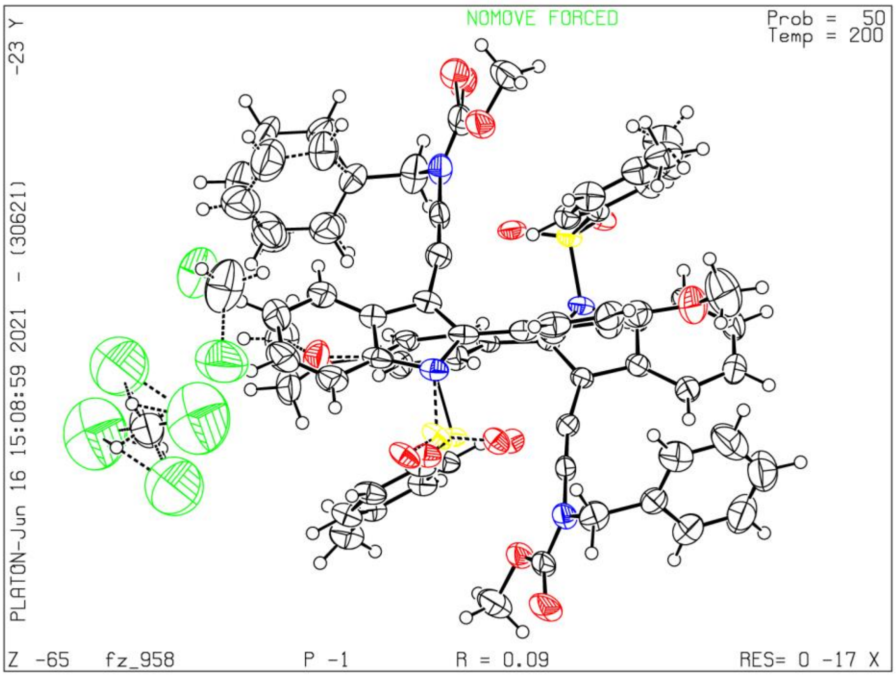

Supplementary Fig. 128 Crystal structure of **8b**-triclinic

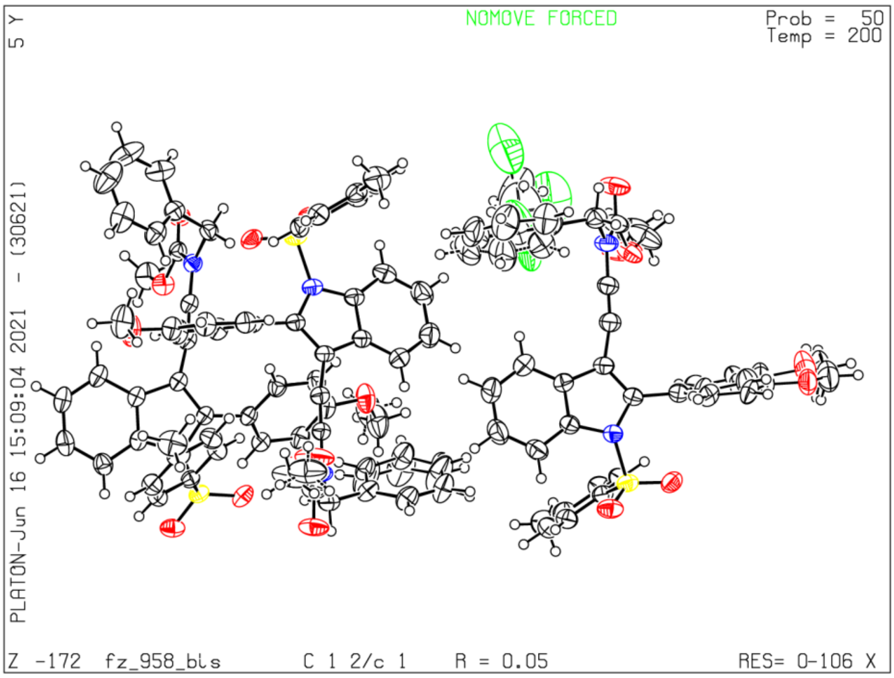

Supplementary Fig. 129 Crystal structure of **8b**-monoclinic

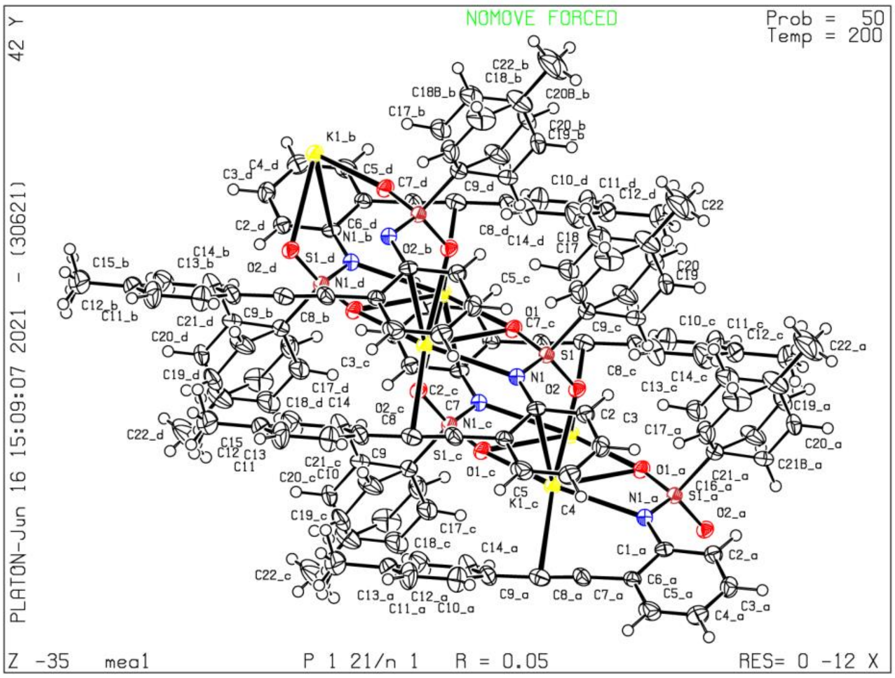

Supplementary Fig. 130 Crystal structure of **1a-K**

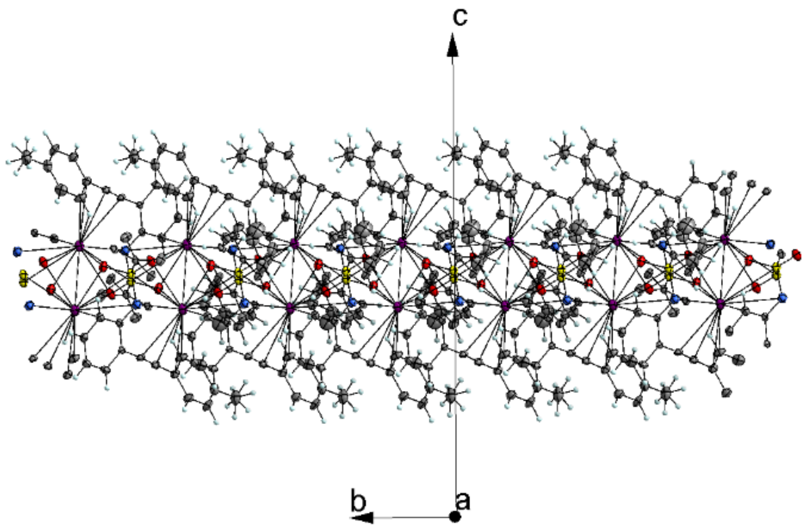

Supplementary Fig. 131 Crystal structure of **1a-K**. This structure forms a linear polymer along the b-axis.

III. Supplementary References

1. Ishay, M. A., Lu, Z. & Yoon, T. P. [2+2] Cycloadditions by oxidative visible light photocatalysis. *J. Am. Chem. Soc.* **132**, 8572-8574 (2010).
2. Singh, A., Teegardin, K., Kelly, M., Prasad, K. S., Krishnan, S. & Weaver, J. D. Facile synthesis and complete characterization of homoleptic and heteroleptic cyclometalated Iridium(III) complexes for photocatalysis. *J. Organomet. Chem*. **776**, 51-59 (2015).
3. Xia, Z., Corcé, V., Zhao, F., Przybylski, C., Espagne, A., Jullien, L. Le Saux, T., Gimbert, Y., Dossmann, H. , Mouriès-Mansuy, V. , Ollivier, C. & Fensterbank, L. Photosensitized oxidative addition to gold(I) enables alkynylative cyclization of o-alkylnylphenols with iodoalkynes. *Nature Chem.* **11**, 797-805 (2019).
4. Thordarson, P. Determining association constants from titration experiments in supramolecular chemistry. *Chem. Soc. Rev.* **40**, 1305-1323 (2011).
5. Thordarson, P. & Brynn Hibbert, D. The death of the Job plot, transparency, open science and online tools, uncertainty estimation methods and other developments in supramolecular chemistry data analysis. *Chem. Commun.* **52**, 12792-12805 (2016).
6. http://supramolecular.org (Online software used for bind fitting experiment)
7. Pike, S. J.; Hunter, C. A.; Brammer, L. & Perutz, R. N. Benchmarking of halogen bond strength in solution with nickel fluorides: bromine versus iodine and perfluoroaryl versus perfluoroalkyl donors. *Chem. Eur. J*. **25**, 9237-9241 (2019).
8. Dumele, O.; Wu, D.; Trapp, N.; Goroff, N. & Diederich, F. Halogen bonding of (iodoethynyl)benzene derivatives in solution. *Org. Lett.* **16**, 4722-4725 (2014).
9. Huggenberger, C. & Fischer, H. Absoption spectra and termin-ation kinetics of transient free radicals in solution observed by UV/VIS spectroscopy with modulated excitation. *Helv. Chim. Acta* **64**, 338-353 (1981).
10. Valeur, B. Molecular Fluoresence. Principles and Applications Ch. 6 (Wiley-VCH, Weinheim, 2002)
11. Alberty, R. A. & Hammes, G. G. Application of the theory of diffusion-controlled reactions to enzyme kinetics. *J. Phys. Chem.* **62**, 154-159 (1958).
12. Rae, M., Fedorov, A. & Berberan-Santos, M., N. Fluorescence quenching with exponential distance dependence: Application to the external heavy-atom effect. *J. Chem. Phys*. **119**, 2223-2231 (2003).
13. Frisch, M. J., Trucks, G. W., Schlegel, H. B., Scuseria, G. E., Robb, M. A., Cheeseman, J. R., Scalmani, G., Barone, V., Mennucci, B., Petersson, G. A., Nakatsuji, H., Caricato, M., Li, X., Hratchian, H. P., Izmaylov, A. F., Bloino, J., Zheng, G., Sonnenberg, J. L., Hada, M., Ehara, M., Toyota, K., Fukuda, R., Hasegawa, J., Ishida, M., Nakajima, T., Honda, Y., Kitao, O., Nakai, H., Vreven, T., Montgomery, Jr., J. A.; Peralta, J. E., Ogliaro, F., Bearpark, M., Heyd, J. J., Brothers, E., Kudin, K. N., Staroverov, V. N., Kobayashi, R., Normand, J., Raghavachari, K., Rendell, A., Burant, J. C., Iyengar, S. S., Tomasi, J., Cossi, M., Rega, N., Millam, J. M., Klene, M., Knox, J. E., Cross, J. B., Bakken, V., Adamo, C., Jaramillo, J., Gomperts, R., Stratmann, R. E., Yazyev, O., Austin, A. J., Cammi, R., Pomelli, C., Ochterski, J. W., Martin, R. L., Morokuma, K., Zakrzewski, V. G., Voth, G. A., Salvador, P., Dannenberg, J. J., Dapprich, S., Daniels, A. D., Farkas, O., Foresman, J. B., Ortiz, J. V., Cioslowski, J. & Fox, D. J. Gaussian 09, Revision C.1 Gaussian, Inc., Wallingford CT, 2009.
14. a) Weigend, F. & Ahlrichs, R. Balanced basis sets of split valence, triple zeta valence and quadruple zeta valence quality for H to Rn: Design and assessment of accuracy. *Phys. Chem. Chem. Phys.* **7**, 3297-3305 (2005). b) Andrae, D., Häußermann, U., Dolg, M., Stoll, H. & Preuß, H. Energy-adjusted *ab initio* pseudopotentials for the second and third row transition elements. *Theor. Chim. Acta* **77**, 123-141 (1990). c) Peterson, K. A., Figgen, D., Goll, E., Stoll, H. & Dolg, M. Systematically convergent basis sets with relativistic pseudopotentials. II. Small-core pseudopotentials and

correlation consistent basis sets for the post-*d* group 16–18 elements. *J. Chem. Phys.* **119**, 11113-11123 (2003).

15. Tomasi, J., Mennucci, B. & Cammi, R. Quantum mechanical continuum solvation models. *Chem. Rev.* **105**, 2999-3094 (2005).
16. a) Pritchard, B. P., Altarawy, D., Didier, B., Gibsom, T. D. & Windus, T. L. New basis set exchange: An open, up-to-date resource for the molecular sciences community *J. Chem. Inf. Model.* **59**, 4814-4820 (2019). b) Feller, D. The role of databases in support of computational chemistry calculations. *J. Comput. Chem.* **17**, 1571-1586 (1996). c) Schuchardt, K. L., Didier, B. T., Elsethagen, T., Sun, L., Gurumoorthi, V., Chase, J., Li, J. & Windus, T. L. Basis set exchange: a community database for computational sciences. *J. Chem. Inf. Model.* **47**, 1045-1052 (2007). d) https://www.basissetexchange.org/.
17. a) Kitaura, K. & Morokuma, K. A new energy decomposition scheme for molecular interactions within the Hartree-Fock approximation. *Int. J. Quantum Chem.* **10**, 325-340 (1976). b) Zhao, L., von Hopffgarten, M. Andrada, D. M. & Frenking, G. Energy decomposition analysis *WIRES Comput. Mol. Sci.* **8**, e1345 (2018).
18. te Velde, G., Bickelhaupt, F. M., Baerends, E. J., Fonseca Guerra, C., van Gisbergen, S. J. A., Snijders, J. G. & Ziegler, T. Chemistry with ADF. *J. Comput. Chem.* **22**, 931-967 (2001).
19. a) Becke, A. D. Density-functional exchange-energy approximation with correct asymptotic behaviour. *Phys. Rev. A* **38**, 3098-3100 (1988). b) Grimme, S., Antony, J., Ehrlich, S. & Krieg, H. A consistent and accurate *ab initio* parametrization of density functional dispersion correction (DFT-D) for the 94 elements H-Pu. *J. Chem. Phys.* **132**, 154104 (2010). c) Dunning, T. H. Gaussian basis functions for use in molecular calculations. III. Contraction of (10s6p) atomic basis sets for the first-row atoms. *J. Chem. Phys.* **55**, 716-723 (1971). d) Faas, S., Snijders, J. G., van Lenthe, J. H., van Lenthe, E. & Baerends, E. J. The ZORA formalism applied to the Dirac-Fock equation. *Chem. Phys. Lett.* **246**, 632-640 (1995).
20. Mitoraj, M. P., Michalak, A., Ziegler, T. A combined charge and energy decomposition scheme for bond analysis. *J. Chem. Theory Comput.* **5**, 962 (2009).
21. Chemcraft - graphical software for visualization of quantum chemistry computations. https://www.chemcraftprog.com.
22. a) Cavallo, G., Metrangolo, P., Milani, R., Pilati, T., Priimagi, A., Resnati, G. & Terraneo G. The halogen bond. *Chem. Rev.* **116**, 2478-2601 (2016). b) Politzer, P., Murray, J. S. & Clark, T. Halogen bonding and other σ-hole interactions: a perspective. *Phys. Chem. Chem. Phys.* **15**, 11178-11189, (2013).
23. Bader, R. F. W., Carroll, M. T., Cheeseman, J. R. & Chang, C. Properties of atoms in molecules: atomic volumes. *J. Am. Chem. Soc.* **109**, 7968-7979 (1987).
24. Stejskal, E. O. & Tanner, J. E. Spin Diffusion Measurements: Spin Echoes in the Presence of a Time-Dependent Field Gradient. *J. Chem. Phys.* **42**, 288-292 (1965).
25. Wu, D., Chen, A. & Johnson Jr, C. S. An improved diffusion-ordered spectroscopy experiment incorporating bipolar-gradient pulses. *J. Magn. Reson. A* **115**, 260-264 (1995).
26. a) Hansen, S. Translational friction coefficients for cylinders of arbitrary axial ratios estimated by Monte Carlo simulation. *J. Chem. Phys.* **121**, 9111-9115 (2004). b) Allouche, L., Marquis, L. A. & Lehn, J.-M. Discrimination of metallosupramolecular architectures in solution by using diffusion ordered spectroscopy (DOSY) experiments : double-stranded helicates of different lenghs. *Chem. Eur. J.* **12**, 7520-7525 (2006).
27. Waldeck, A. R. P., Kuchel, W. A., Lennon, J. & Capman, B. E. NMR Diffusion measurements to characterise membrane transport and solute binding. *Prog. Nucl. Magn. Reson. Spectrosc.* **30**, 39-68 (1997).

28. Han, X. & Lu, X. Cationic Pd(II)-catalyzed tandem reaction of 2-arylethynylanilines and aldehydes: An efficient synthesis of substituted 3-hydroxymethyl indoles. *Org. Lett.* **12**, 3336-3339 (2010).
29. Li, Y.-L., Li, J., Yu, S.-N., Wang, J.-B., Yu, Y.-M. & Deng, J. A concise approach for the synthesis of 3-iodoindoles and 3-iodobenzo[b]furans via Ph_3P-catalyzed iodocyclization. *Tetrahedron* **71**, 8271-8277 (2015).
30. Liu, J., Xie, X., Liu, Y. Silver-catalyzed cascade cyclization–stannylation of *o*-alkynylaniline derivatives with 2-tributylstannylfuran: an efficient synthesis of (3-indolyl)stannanes. *Chem. Commun.* **49**, 11794-11796 (2013).
31. Ye, Y., Cheung, K. P. S., He, L. & Tsui, G. C. Domino cyclization/trifluoromethylation of 2-alkynylanilines using fluoroform-derived $CuCF_3$: synthesis of 3-(trifluoromethyl)indoles. *Org. Chem. Front.* **5**, 1511-1515 (2018).
32. Swamy, N. K., Yazici, A. & Pyne, S. G. Copper-mediated cyclization-halogenation and cyclization-cyanation reactions of β-hydroxyalkynes and o-alkynylphenols and anilines. *J. Org. Chem.* **75**, 3412-3419 (2010).
33. Takaesu, N. A., Ohta, E., Zakharov, L. N., Johnson, D. W. & Haley, M. M. Synthesis and properties of naphtho[2,3-*e*]-1,2-azaphosphorine 2-oxides: PN-anthracene analogues. *Organometallics* **36**, 2491-2493 (2017).
34. Zhao, Y., Jin, J. & Chan, P. W. H. Gold catalyzed photoredox C1-alkynylation of *N*-alkyl-1,2,3,4-tetrahydroisoquinolines by 1-bromoalkynes with UVA LED light. *Adv. Synth. Catal.* **361**, 1313-1321 (2019).
35. Iqbal, N., Iqbal, N., Han, S. S. & Cho, E. Synthesis of fluoroalkylated alkynes *via* visible-light photocatalysis. *J. Org. Biomol. Chem.* **17**, 1758-1762 (2019).
36. Reddy, K. R., Venkateshwar, M., Maheswari, C. U. & Kumar, P. S. Mild and efficient oxy-iodination of alkynes and phenols with potassium iodide and *tert*-butyl hydroperoxide. *Tetrahedron Lett.* **51**, 2170-2173 (2010).
37. Lehnherr, D., Alzola, J. M., Lobkovsky, E. B. & Dichtel, W. R. Regioselective synthesis of polyheterohalogenated naphthalenes via the benzannulation of haloalkynes. *Chem. Eur. J.* **21**, 18122-18127 (2015).
38. Wang, Y. & Danheiser, R. L. Synthesis of 2-iodoynamides and regioselective [2+2] cycloadditions with ketene.*Tetrahedron Lett.* **52**, 2111-2114 (2011).
39. Palatinus, L. & Chapuis, G. SUPERFLIP - a computer program for the solution of crystal structures by charge flipping in arbitrary dimensions. *J. Appl. Crystallogr.* **40**, 786-790 (2007).
40. Sheldrick, G. M. Crystal structure refinement with SHELXL. *Acta Cryst.* **C71**, 3-8 (2015).
41. Dolomanov, O. V., Bourhis, L. J., Gildea, R. J., Howard, J. A. K. & Puschmann, H. *OLEX2*: a complete structure solution, refinement and analysis program. *J. Appl. Crystallogr.* **42**, 339-341 (2009).
42. Farrugia, L. J. *WinGX* suite for small-molecule single-crystal crystallography. *J. Appl. Crystallogr.* **32**, 837-838 (1999).
43. Li, Y.-L., Li, J., Yu, S.-N., Wang, J.-B., Yu, Y.-M. & Deng, J. A concise approach for the synthesis of 3-iodoindoles and 3-iodobenzo[b]furans via Ph_3P-catalyzed iodocyclization. *Tetrahedron* **71**, 8271-8277 (2015).
44. Jang, Y. H. & Youn, S. W. Metal-free C–H amination for indole synthesis. *Org. Lett.* **16**, 3720–3723 (2014).
45. Maaliki, C., Chevalier, Y., Thiery, E. & Thibonnet, J. Palladium and copper catalyzed Sonogashira decarboxylative coupling of aryl iodides and alkynyl carboxylic acids. *Tetrahedron Lett.* **57**, 3358–3362 (2016).
46. Nissen, F. & Detert, H. Total Synthesis of lavendamycin by a [2+2+2] cycloaddition. *Eur J. Org. Chem.* 2845–2853 (2011).
