# Supplementary material for: Reactant-induced photoactivation of in situ generated organogold intermediates leading to alkynylated indoles via Csp2-Csp cross-coupling
Source: Nat Commun. 2022 Apr 28;13:2295. doi: 10.1038/s41467-022-29982-2 (PMC9051093; doi:10.1038/s41467-022-29982-2)
Supplement: Supplementary file 2 — Description of Additional Supplementary Files [file 41467_2022_29982_MOESM2_ESM.pdf]

Description of Additional Supplementary files

File name : Supplementary Data 1

Description : Supplementary Table 3: Cartesian coordinates of the optimized structures of the compounds involved in this work.
